# A Catalytic Asymmetric Hydrolactonization

**DOI:** 10.1021/jacs.3c01404

**Published:** 2023-04-12

**Authors:** Rajat Maji, Santanu Ghosh, Oleg Grossmann, Pinglu Zhang, Markus Leutzsch, Nobuya Tsuji, Benjamin List

**Affiliations:** † Max-Planck-Institut für Kohlenforschung, Kaiser-Wilhelm-Platz 1, 45470 Mülheim an der Ruhr, Germany; ‡ Institute for Chemical Reaction Design and Discovery (WPI-ICRedd), Hokkaido University, Sapporo 001-0021, Japan

## Abstract

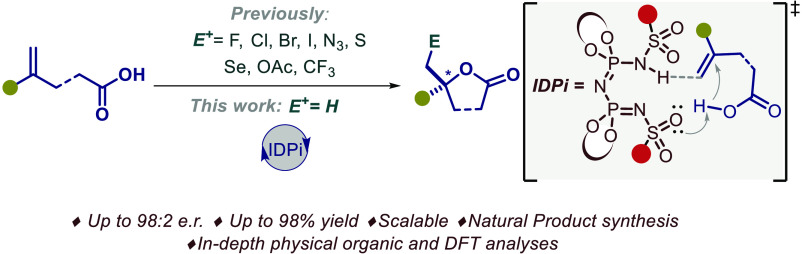

Despite recent advancements in the development of catalytic asymmetric electrophile induced lactonization reactions of olefinic carboxylic acids, the archetypical hydrolactonization has long remained an unsolved and well-recognized challenge. Here, we report the realization of a catalytic asymmetric hydrolactonization using a confined imidodiphosphorimidate (IDPi) Brønsted acid catalyst. The method is operationally simple, scalable, and compatible with a wide variety of substrates. Its potential is showcased with concise syntheses of the sesquiterpenes (−)-boivinianin A and (+)-gossonorol. Through in-depth physicochemical and DFT analyses, we derive a nuanced picture of the mechanism and enantioselectivity of this reaction.

With an estimated one-third of all drugs and natural products featuring lactones,^1^ the development of lactonization strategies continues to garner considerable attention.^2^ Especially, lactones that are formally derived from stereogenic, methyl-substituted tertiary alcohols, for simplicity called “tertiary lactones” here, exemplify biological significance.^3^ These structures are privileged not only due to their presence in a vast array of natural products and drugs (Figure 1a) but also as valuable synthetic intermediates for elaboration into numerous bioactives. A catalytic asymmetric hydrolactonization of unsaturated carboxylic acids would arguably provide an elegant and straightforward solution to this problem. However, contemporary asymmetric electrophilic lactonization methods typically introduce an electrophile other than the proton, i.e. a halide and related functional groups (Figure 1b).^4^ While a reductive replacement of such a group can be realized, a direct hydrolactonization procedure would evidently provide the desired motifs in a more atom- and step-economical manner (Figure 1c). We now report a general catalytic asymmetric hydrolactonization of readily available γ,δ-unsaturated carboxylic acids using a confined imidodiphosphorimidate (IDPi) catalyst.

**Figure 1:**
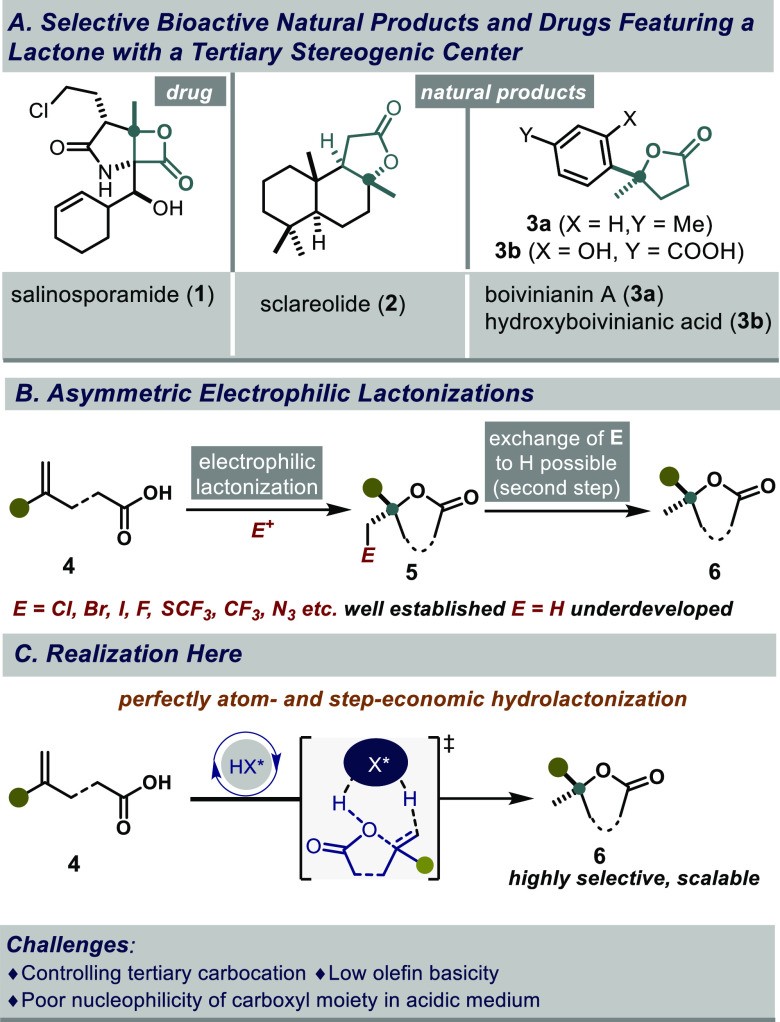
(a) Bioactives containing lactones with methyl-substituted stereocenters. (b) Current strategy and limitations. (c) Our design.

Since their discovery in 1883,^5^ electrophilic lactonizations have continually served as workhorse organic transformations, and numerous such variants have been developed in recent years.^6^ Despite significant effort, however, the catalytic asymmetric hydrolactonization still remains in its infancy.^7^ A phosphonium salt and an iridium complex have previously been utilized, albeit with limited substrate scope.^8,9^ Constructing enantiomerically enriched tertiary lactones upon direct hydrofunctionalization remains a challenging problem. Recognizing these limitations and inspired by our recent success in activating unbiased olefins toward hydrofunctionlizations,^10^ we sought to design a suitable chiral organocatalyst capable of imparting stereocontrol on the process. Two critical factors were central to the design of such a catalyst: first, enabling substrate reactivity via activating the alkene;^11^ and second, providing an environment conducive to facial discrimination during cyclization, rendering the process stereoselective.^12^

In earlier studies, we established that fine-tuning different substituents of IDPi catalysts can significantly enhance their acidity, enabling protonation of unactivated olefins.^10^ Additionally, these catalysts contain small enzyme-like substrate-binding cavities that provide tunable microenvironments, enabling controlled stereodifferentiation.^13^ We therefore envisioned that IDPi catalysts possess all the requisite characteristics to position them for successful application in the proposed transformation.

With the foregoing mechanistic blueprint in hand, we set out to investigate the desired transformation using γ,δ-unsaturated acid **4a** as substrate. Consistent with our initial hypothesis, moderately acidic and unconfined Brønsted acids were ineffective in promoting the desired transformation (see the Supporting Information (SI), Table S1). We surmised that the optimized conditions for our recently disclosed catalytic enantioselective hydroalkoxylation might be an appropriate starting point for further investigation.^11a^ However, to our disappointment, all efforts with the previous optimal catalyst **7a** proved futile (Table 1, entry 1), with no observable reaction. We reasoned that the moderate acidity of IDPi **7a**—featuring aryl sulfonyl groups—might be insufficient to trigger transformation of olefin **4a**, and upon turning to the more acidic triflyl-based catalyst **7b**, we were indeed pleased to find reactivity, albeit with modest conversion and enantioselectivity (Table 1, entry 2). Changing the solvent from cyclohexane (CyH) to CHCl_3_ significantly improved conversion and even allowed us to reduce the temperature to boost selectivity (Table 1, entries 2, 3, and 8). Encouraged by these results, we decided to fine-tune the acidity and active site topography of the catalyst by judicially varying 3,3′-positions of BINOLs and inner core sulfonyl groups. Screening of a number of 3,3′-substituents showed that 4-^*t*^Bu-C_6_H_4_ performed best (Table 1, entries 4–8). When altering the inner core sulfonyl groups, a significant increase in enantioselectivity was observed upon switching from triflyl (**7b**) to the pentafluorophenylsulfonyl group (**7g**; Table 1, entries 8, 9). Similarly, catalyst **7h**, with an even bulkier perfluoronaphthalene-2-sulfonyl group, gave excellent enantioselectivity (Table 1, entry 10), which was further increased by lowering temperature and concentration (Table 1, entries 11, 12) leading to an e.r. of 96.5:3.5. Comprehensive solvent screening (see SI) revealed that both CHCl_3_ and toluene could lead to high stereoselectivity, while the conversion was generally better in CHCl_3_.

**Table 1:**
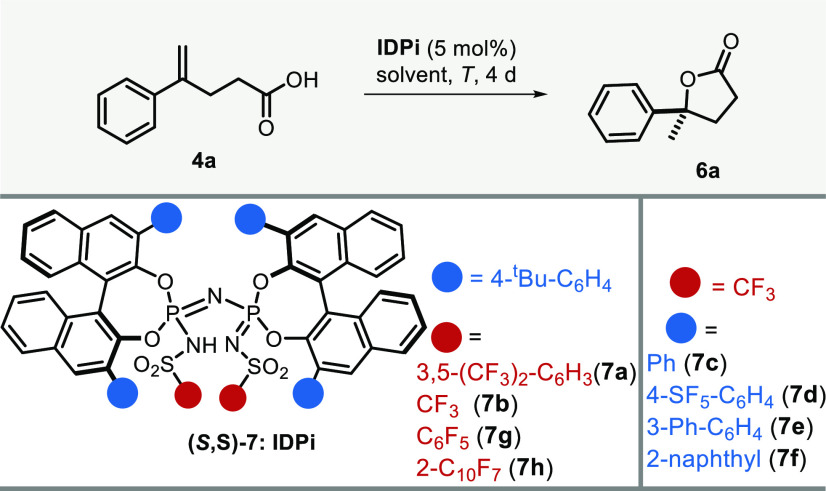
Reaction Development^a^

Entry	IDPi	*T* (°C)	Solvent	Conv. (%)^b^	e.r.^c^
1	**7a**	60	CyH	0	–
2	**7b**	60	CyH	45	67:33
3	**7b**	60	CHCl_3_	>95	68.32
4^d^	**7c**	40	CHCl_3_	>95	50.5:49.5
5^d^	**7d**	40	CHCl_3_	>95	51.5:48.5
6^d^	**7e**	40	CHCl_3_	>95	53:47
7^d^	**7f**	40	CHCl_3_	>95	55:45
8^d^	**7b**	40	CHCl_3_	>95	75.5:24.5
9^d^	**7g**	40	CHCl_3_	>95	93:7
10^d^	**7h**	40	CHCl_3_	>95	95:5
11	**7h**	40	CHCl_3_	>95	95.5:4.5
12^e^	**7h**	20	CHCl_3_	87	96.5:3.5

a Unless otherwise noted, reactions used 0.05 mmol of substrate at 0.2 M concentration.

b Conversion to **6a** was determined by ^1^H NMR with anisole as internal standard.

c Enantiomeric ratio (e.r.) of **6a** was determined by chiral HPLC.

d At 0.5 M concentration.

e For 5 d.

Having optimized the reaction conditions, we next evaluated the substrate scope (Figure 2a). A broad range of aromatic groups was well tolerated, with varied substitution patterns and electronic properties, affording tertiary lactones **6a**–**m** in good to excellent yields with consistently high enantioselectivity. Remarkably, catalyst **7h** was proficient in handling sterically demanding *ortho*-substituted aromatics (**6l**, **6m**) at slightly elevated temperature. To test robustness, operational simplicity, and practicality of our method, conversion of olefin **4j** to lactone **6j** was performed on a gram scale without any deterioration in enantioselectivity, recovering the catalyst (95%) by column chromatography. Substrates bearing polyaromatic (**4n**) and heterocyclic (**4o**) groups also proved compatible, leading to the desired lactones **6n** and **6o** with high yield and enantioselectivity. An initial attempt to extend this methodology to the synthesis of six-membered tertiary lactones (**6p**) was also successful.

**Figure 2:**
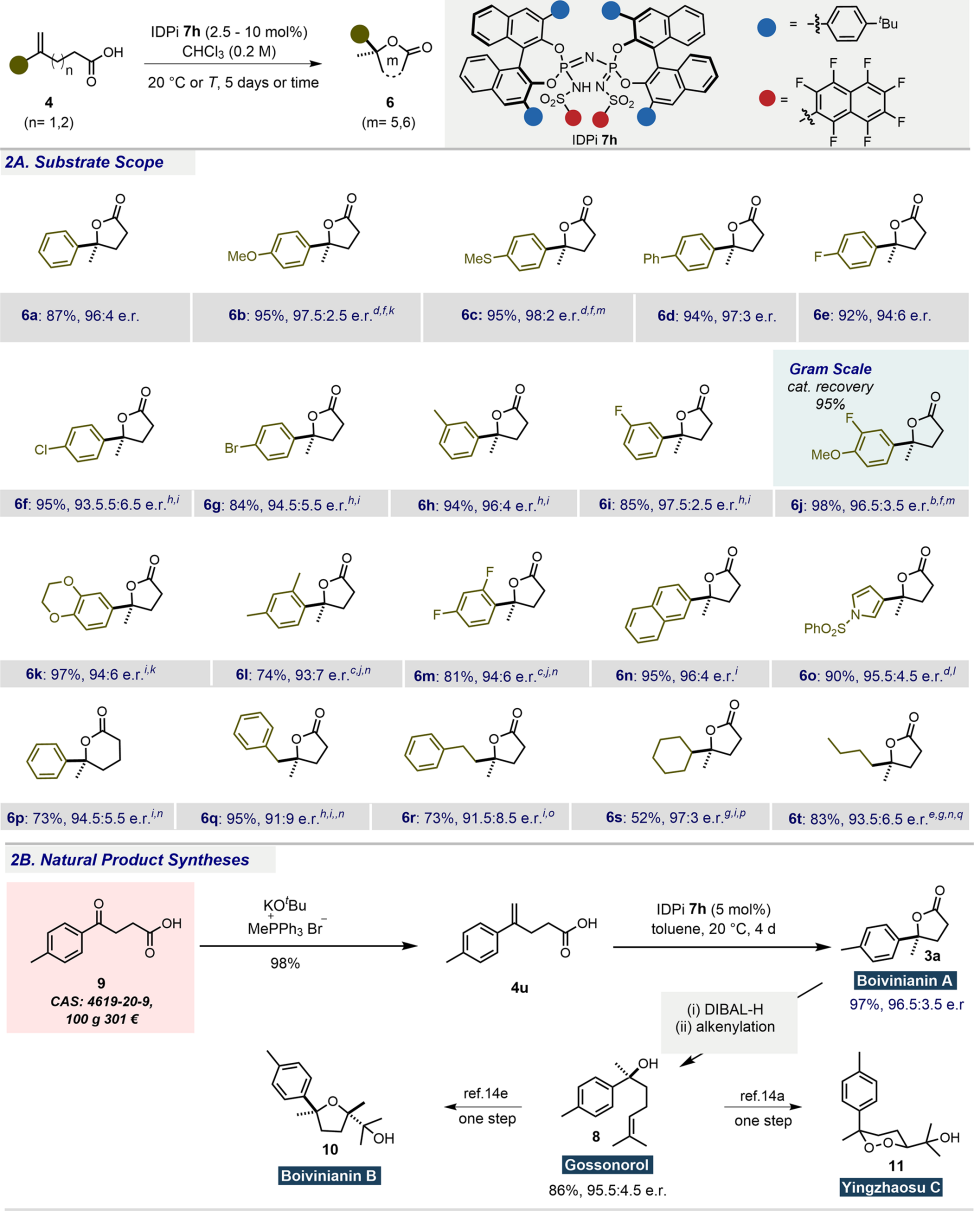
(A) Scope of hydrolactonization, showing isolated yields. Enantiomeric ratios (e.r.) were measured by HPLC, and absolute configurations were determined by literature comparison. ^*a*^Unless otherwise stated, reactions were carried out with substrate **4** (0.10 mmol) and IDPi **7h** (5 mol %) in CHCl_3_ (0.2 M) at 20 °C for 5 days. ^*b*^IDPi **7h** (2.5 mol %). ^*c*^IDPi **7h** (10 mol %). ^*d*^CHCl_3_ (0.1 M). ^*e*^Cyclohexane (0.1 M). ^*f*^0 °C. ^*g*^10 °C. ^*h*^40 °C. ^*i*^Toluene (0.2 M). ^*j*^Toluene (0.5 M). ^*k*^2 days. ^*l*^3 days. ^*m*^4 days. ^*n*^8 days. ^*o*^14 days. ^*p*^17 days. ^*q*^IDPi **7g** (10 mol %) was used. (B) Synthesis of natural products (see SI for exact procedures).

Next, we tested our catalyst with aliphatic substrates and were pleased to observe excellent reactivity and enantioselectivity for benzyl- and phenethyl-substituted lactones (**6q**, **6r**). Even completely unbiased aliphatic substrates with cyclohexyl (**4s**) and butyl (**4t**) groups furnished the corresponding lactones **6s** and **6t**, in good to moderate yield and with excellent enantioselectivity. However, these conditions were not compatible with higher (tri- and tetra-substituted) olefin substrates, highlighting a present limitation of this new methodology.

The utility of our approach is illustrated by an efficient route to the antioxidant (−)-boivinianin A **3a** (Figure 2b), which was accessed directly in excellent yield and with high enantioselectivity by subjecting γ-alkenoic acid **4u** to our optimized conditions. Subsequent one-pot functionalization of this natural product completed a concise total synthesis of the sesquiterpene (−)-gossonorol **8** in 86% yield. Notably, the stereoselective synthesis of both natural products using our method was achieved in three steps from inexpensive carboxylic acid **9**, thereby representing a more direct and economically viable alternative to existing routes.^14^ Moreover, (−)-gossonorol has found utility as a synthetic precursor to other natural products including boivinianin B **10** and the antimalarial compound yingzhaosu C **11** (Figure 2b),^14a,14e^ further emphasizing the potential of the described hydrolactonization methodology.

Keen to understand the mechanism of our catalytic hydrolactonization, we performed a series of kinetic and spectroscopic studies. The reaction orders of catalyst **7g** and substrate **4u** were investigated using variable time normalization analysis (VTNA) of concentration profiles obtained from ^1^H NMR at 50 °C. The normalized curves overlapped best when assuming first-order dependence on both catalyst and substrate (Figure 3A1). The steady-state approximation agreed with NMR analysis over the reaction course, suggesting that the free catalyst is the resting state (Figure S16). To elucidate the nature of the transition state, we performed a Hammett analysis with different *para*-substituents of aryl substituted γ-alkenoic acids (Figure 3A2). Plotting log(*k*_X_/*k*_H_) against σ_*p*_^+^ revealed a negative linear correlation. The nature of the observed linear free-energy relationship (ρ^+^ = −2.6 ± 0.2) indicates significant positive charge accumulation at the reaction center during the rate-determining step. Eyring analysis of the decay rate of **4u** over the temperature range from 30 to 60 °C allowed us to determine the activation enthalpy Δ*H*^‡^ (15.5 ± 0.3 kcal mol^–1^), activation entropy Δ*S*^‡^ (−20.7 ± 1.0 cal mol^–1^K^–1^), and free energy Δ*G*^‡^ (22.0 ± 0.5 kcal mol^–1^) of the reaction (Figure 3A3).

**Figure 3:**
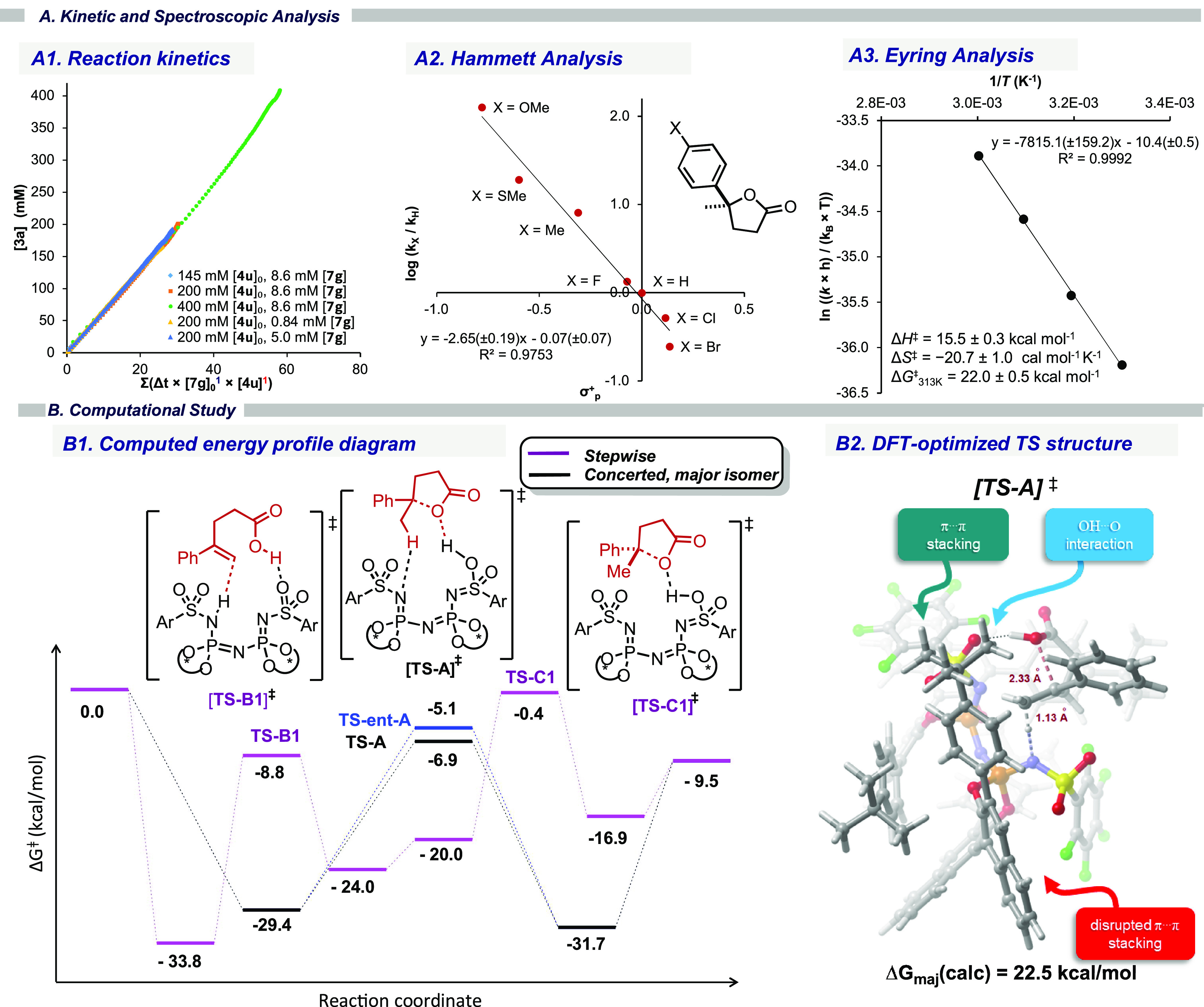
(A) Mechanistic study of the hydrolactonization reaction using catalyst **7g** and substrate **4u**. (A1) The overall reaction orders of the individual components were determined using VTNA. (A2) The nature of the reaction intermediate using a Hammett study. (A3) The thermodynamic parameters using catalyst **7g** and substrate **4u** determined by Eyring analysis. (B) Computational analysis of the hydrolactonization reaction. (B1) Computed free energy profile of asymmetric hydrolactonization at the [B3LYP-D3/def2-TZVP+C-PCM-(chloroform)//PBE-D3/def2-SVP+C-PCM-(chloroform)] level at 313.15 K. (B2) TS structure leading to the major enantiomer of **6a**.

For further molecular-level insight into these processes, we undertook density functional theory (DFT) calculations using substrate **4a** and IDPi catalyst **7g**. Previous studies on electrophilic lactonizations have suggested two distinct mechanistic pathways: (i) stepwise protonation and subsequent stereoselective cyclization,^15^ or (ii) concerted stereoselective cyclization.^16^ Comprehensive analysis of both putative reaction pathways revealed that this reaction follows an asynchronous concerted mechanism (Figure 3B1, also Figure S19) where alkene protonation (C–H: 1.18 Å) precedes ring closure (C–O: 2.33 Å; Figure 3B2).^17^ This marked asynchronicity leads to a net accumulation of positive charge at the electrophilic carbon center (+0.26*e*), consistent with the Hammett study, and the computed energetic span is in qualitative agreement with that of the experiment.^18^ Furthermore, the hydrogen bond established early in molecular recognition between the substrate carboxylic acid group and the sulfone moiety of the catalyst (OH···O interaction) is conserved along the entire reaction pathway. We next looked to rationalize the stereochemical outcome of the transformation, noting that the DFT-computed selectivity of 94.5:5.5 (*ΔΔG*^‡^ = 1.81 kcal mol^–1^) between the two stereodetermining TS structures (Figure S13) was in excellent agreement with experimental observations (93:7; *ΔΔG*^‡^ = 1.7 kcal mol^–1^). To trace its origins, we employed distortion/interaction analysis,^19^ which considers the energy penalty for distortion of the substrate into the transition state conformation as the key driver behind selectivity.^20^ Finally, we challenged our computational model to rationalize the substrate scope limitation in the presence of tri- and tetra-substituted olefins (see Figure S6 in the SI). Based on the computed TS structure, the methylene group of the substrate protrudes into the binding pocket to accept a proton from the sulfonamide group of the catalyst. This pocket is just adequate to accommodate a methylene group but not any higher-substituted alkene (Figure S21) resulting in enzyme-like substrate specificity observed in this case.

To summarize, we have described an organocatalytic asymmetric hydrolactonization that efficiently generates enantiomerically enriched tertiary lactones. Using a confined IDPi catalyst, we realized this transformation under mild conditions for a diverse array of substrates with good to excellent enantioselectivities, while suppressing competing alkene isomerization. The utility of our method was further demonstrated by concise total syntheses of (−)-boivinianin A (**3a**) and (+)-gossonorol (**8**), further establishing formal syntheses of boivinianin B (**10**) and yingzhaosu C (**11**). Experimental and computational analyses deconstructed the reaction mechanism and its underlying stereoselectivity. The new methodology fills a long-standing gap in the asymmetric electrophilic lactonization literature and recapitulates many features of enzymatic catalysis. We believe that the principles underlying the present study will enable a leap forward in designing other elusive alkene hydrofunctionalizations.

## Supporting Information Available

Experimental, computational, and spectroscopic details (PDF)

## References

[ref1] aKang E. J.; Lee E. Total Synthesis of Oxacyclic Macrodiolide Natural Products. Chem. Rev. 2005, 105 (12), 4348–4378. 10.1021/cr040629a. 16351047

[ref2] aSeitz M.; Reiser O. Synthetic Approaches Towards Structurally Diverse γ-Butyrolactone Natural-Product-Like Compounds. Curr. Opin. Chem. Biol. 2005, 9 (3), 285–292. 10.1016/j.cbpa.2005.03.005. 15939330

[ref3] aJiang M.; Wu Z.; Guo H.; Liu L.; Chen S. A Review of Terpenes from Marine-Derived Fungi: 2015–2019. Mar. Drugs 2020, 18 (6), 321 10.3390/md18060321. 32570903 PMC7345631

[ref4] aDenmark S. E.; Kuester W. E.; Burk M. T. Catalytic, Asymmetric Halofunctionalization of Alkenes—A Critical Perspective. Angew. Chem., Int. Ed. 2012, 51 (44), 10938–10953. 10.1002/anie.201204347. PMC3529098 23011853

[ref5] Dowle M. D.; Davies D. I. Synthesis and synthetic utility of halolactones. Chem. Soc. Rev. 1979, 8 (2), 171–197. 10.1039/cs9790800171.

[ref6] aWhitehead D. C.; Yousefi R.; Jaganathan A.; Borhan B. An Organocatalytic Asymmetric Chlorolactonization. J. Am. Chem. Soc. 2010, 132 (10), 3298–3300. 10.1021/ja100502f. 20170118 PMC2883568

[ref7] aUyanik M.; Ishibashi H.; Ishihara K.; Yamamoto H. Biomimetic Synthesis of Acid-Sensitive (−)-Caparrapi Oxide and (+)-8-Epicaparrapi Oxide Induced by Artificial Cyclases. Org. Lett. 2005, 7 (8), 1601–1604. 10.1021/ol050295r. 15816762

[ref8] Sakuma M.; Sakakura A.; Ishihara K. Kinetic Resolution of Racemic Carboxylic Acids through Asymmetric Protolactonization Promoted by Chiral Phosphonous Acid Diester. Org. Lett. 2013, 15 (11), 2838–2841. 10.1021/ol401313d. 23676002

[ref9] aNagamoto M.; Nishimura T. Iridium-catalyzed asymmetric cyclization of alkenoic acids leading to γ-lactones. Chem. Comm 2015, 51 (70), 13466–13469. 10.1039/C5CC05393E. 26216621

[ref10] aTsuji N.; Kennemur J. L.; Buyck T.; Lee S.; Prévost S.; Kaib P. S. J.; Bykov D.; Farès C.; List B. Activation of Olefins via Asymmetric Brønsted Acid Catalysis. Science 2018, 359 (6383), 1501–1505. 10.1126/science.aaq0445. 29599238

[ref11] aAkiyama T.; Mori K. Stronger Brønsted Acids: Recent Progress. Chem. Rev. 2015, 115 (17), 9277–9306. 10.1021/acs.chemrev.5b00041. 26182163

[ref12] Schreyer L.; Properzi R.; List B. IDPi Catalysis. Angew. Chem., Int. Ed. 2019, 58 (37), 12761–12777. 10.1002/anie.201900932. 30840780

[ref13] aAmatov T.; Tsuji N.; Maji R.; Schreyer L.; Zhou H.; Leutzsch M.; List B. Confinement-Controlled, Either syn- or anti-Selective Catalytic Asymmetric Mukaiyama Aldolizations of Propionaldehyde Enolsilanes. J. Am. Chem. Soc. 2021, 143 (36), 14475–14481. 10.1021/jacs.1c07447. 34436899 PMC8447262

[ref14] aBoukouvalas J.; Pouliot R.; Fréchette Y. Concise Synthesis of Yingzhaosu C and epi-Yingzhaosu C by Peroxyl Radical Cyclization. Assignment of Relative Configuration. Tetrahedron Lett. 1995, 36 (24), 4167–4170. 10.1016/0040-4039(95)00714-N.

[ref15] aDenmark S. E.; Burk M. T.; Hoover A. J. On the Absolute Configurational Stability of Bromonium and Chloronium Ions. J. Am. Chem. Soc. 2010, 132 (4), 1232–1233. 10.1021/ja909965h. 20058922

[ref16] Ashtekar K. D.; Vetticatt M.; Yousefi R.; Jackson J. E.; Borhan B. Nucleophile-Assisted Alkene Activation: Olefins Alone Are Often Incompetent. J. Am. Chem. Soc. 2016, 138 (26), 8114–8119. 10.1021/jacs.6b02877. 27284808 PMC5340197

[ref17] Computational study performed in ORCA 4.2 at the B3LYP-D3/def2-TZVP+CPCM-(Chloroform)//PBE-D3/def2-SVP+ CPCM-(Chloroform) level of theory. For additional details, see SI-Table S2.

[ref18] Kozuch S.; Shaik S. How to Conceptualize Catalytic Cycles? The Energetic Span Model. Acc. Chem. Res. 2011, 44 (2), 101–110. 10.1021/ar1000956. 21067215

[ref19] Bickelhaupt F. M.; Houk K. N. Analyzing Reaction Rates with the Distortion/Interaction-Activation Strain Model. Angew. Chem., Int. Ed. 2017, 56 (34), 10070–10086. 10.1002/anie.201701486. PMC5601271 28447369

[ref20] See SI (Table S4) for details.

