# Supplementary material for: A Catalytic Asymmetric Hydrolactonization
Source: J Am Chem Soc. 2023 Apr 12;145(16):8788–93. doi: 10.1021/jacs.3c01404 (PMC10141295; doi:10.1021/jacs.3c01404)
Supplement: Supplementary file 1 — ja3c01404_si_001.pdf [file ja3c01404_si_001.pdf]

A Catalytic Asymmetric Hydrolactonization

Rajat Maji, Santanu Ghosh,† Oleg Grossmann,† Pinglu Zhang, Markus Leutzsch, Nobuya Tsuji, and Benjamin List*

Corresponding Author
*Benjamin List – Max-Planck-Institut für Kohlenforschung, D45470 Mülheim an der Ruhr, Germany; Institute for Chemical Reaction Design and Discovery (WPI-ICRedd), Hokkaido University, Sapporo 001-0021, Japan; orcid.org/0000-0002-9804-599X; list@kofo.mpg.de.

Table of Contents

1. General Information and Instrumentation 3

2. Synthesis and Characterization of Substrates 5

3. Synthesis and Characterization of IDPi Catalysts 6

4. Reaction Development and Optimization 7

5. Organocatalytic Asymmetric Hydrolactonization and its Application 10

6. Determination of the Absolute Configuration 17

7. Kinetic Studies to Investigate the Reaction Mechanism 18

8. Computational Studies 29

8.1 Methods 29

8.2 Results and Discussions 30

9. References 35

10. NMR Spectra of All New Catalyst, Starting Materials and Products 37

11. HPLC & GC Traces of Lactones 69

12. Optimized Cartersian Coordinates from Computational Analysis 93

1. General Information and Instrumentation

Unless otherwise stated, all reactions were magnetically stirred and conducted in oven-dried (80 °C) or flame-dried glassware in anhydrous solvents under Ar, applying standard Schlenk techniques. Solvents and liquid reagents, as well as solutions of solid or liquid reagents were added via syringes, stainless steel or polyethylene cannulas through rubber septa or through a weak Ar counter-flow. Solid reagents were added through a weak Ar counter-flow. Cooling baths were prepared in Dewar vessels, filled with ice/water (0 °C), cooled acetone (> –78 °C) or dry ice/acetone (–78 °C). Heated oil baths were used for reactions requiring elevated temperatures. Solvents were removed under reduced pressure at 40 °C using a rotary evaporator, and unless otherwise stated, the remaining compound was dried in high vacuum (10^{-3} mbar) at ambient temperature. All given yields are isolated yields of chromatographically and NMR spectroscopically pure materials, unless otherwise stated.

Chemicals: Chemicals were purchased from commercial suppliers (including abcr, Acros, Alfa Aesar, Fluorochem, TCI and Sigma-Aldrich) and used without further purification unless otherwise stated. Commercially available boronic acids and keto acids were purchased from Merck, Alfa Aesar and Acros Organics.

Solvents: Solvents (CH_2Cl_2, $CHCl_3$, Et_2O, THF, toluene) were dried by distillation from an appropriate drying agent in the technical department of the Max-Planck-Institut für Kohlenforschung and received in Schlenk flasks under Ar. In addition, more solvents (acetone, benzene, cyclohexane, methylcyclohexane, 1,4-dioxane, DMF, DMSO, EtOAc, EtOH, MeCN, MeOH, MTBE, *n*-hexane, *n*-heptane, *n*-pentane, methylene) were purchased from commercial suppliers and dried over molecular sieves.

Inert Gas: Dry argon was purchased from Air Liquide with > 99.5% purity.

Thin Layer chromatography: Thin-layer chromatography (TLC) was performed using silica gel pre-coated glass plates (SIL G-25, with fluorescent indicator UV254; Macherey-Nagel) and aluminium oxide pre-coated plastic sheets (Polygram AlOx N, 0.2 mm, with fluorescent indicator UV254; Macherey-Nagel), which were visualized by irradiation with UV light (λ = 254 or 366 nm), basic $KMnO_4$, phosphomolybdic acid (PMA) and/or anisaldehyde. Preparative thin-layer chromatography was performed on silica gel pre-coated glass plates SIL G-100, with fluorescent indicator UV254 (Macherey-Nagel).

Column Chromatography: Flash column chromatography (FCC) was carried out using Merck silica gel (60 Å, 230−400 mesh, particle size 0.040−0.063 mm) or an automated flash purification system: BIOTAGE Isolera™ FOUR (pre-packed Sfär Silica HC D columns 10 g and 25 g) using technical grade solvents. Elution was accelerated using compressed air. All reported yields, unless otherwise specified, refer to spectroscopically and chromatographically pure compounds.

Nomenclature: Nomenclature follows the suggestions proposed by the computer program ChemBioDraw (15.0.0.106) of CBD/cambridgesoft.

Nuclear Magnetic Resonance Spectroscopy: ^{1}H, ^{13}C, ^{19}F, ^{31}P Nuclear magnetic resonance (NMR) spectra for characterisation were recorded on a Bruker Avance III 500 or Bruker Avance NEO 600 MHz NMR spectrometer in a suitable deuterated solvent. The solvent employed and respective measuring frequency are indicated for each experiment. ^{1}H and ^{13}C chemical shifts are reported relative to tetramethylsilane (TMS) with two or one digits after the comma. The resonance multiplicity is described as s (singlet), d (doublet), t (triplet), q (quartet), m (multiplet), and bs (broad singlet). All spectra were recorded at 298 K unless otherwise noted, processed with MestReNova 14.1.2 suits of program, and coupling constants are reported as observed. The residual deuterated solvent signal relative to tetramethylsilane (TMS) was used as the internal reference in ^{1}H NMR spectra (e.g. $CDCl_3$ = 7.26 ppm, CD_2Cl_2 = 5.32 ppm). ^{19}F and ^{31}P NMR shifts are reported relative to $CFCl_3$ and H_3PO_4 respectively. Signals

are reported as follows: chemical shift δ in ppm (multiplicity, coupling constant *J* in Hz, number of protons). ^{13}C NMR spectra reported in ppm from tetramethylsilane (TMS) with the solvent resonance as the internal standard (e.g. $CDCl_3$ = 77.2 ppm, CD_2Cl_2 = 53.8 ppm). All X-nuclei were acquired proton decoupled unless otherwise noted.

Kinetic NMR measurements were performed at a Bruker Avance III 300 MHz WB NMR spectrometer equipped with a Bruker 5mm BBFO NMR probe with z-gradient. The temperatures set for the kinetic measurements were calibrated with a 80% Glycol in DMSO-d_6 sample.

Mass Spectrometry: Electron impact (EI) mass spectrometry (MS) was performed on a Finnigan MAT 8200 (70 eV) or MAT 8400 (70 eV) spectrometer. Electrospray ionization (ESI) mass spectrometry was conducted on a Bruker ESQ 3000 spectrometer. High resolution mass spectrometry (HRMS) was performed on a Finnigan MAT 95 (EI) or Bruker APEX III FTMS (7T magnet, ESI). The ionization method and mode of detection employed is indicated for the respective experiment and all masses are reported in atomic units per elementary charge (m/z) with an intensity normalized to the most intense peak.

Specific Rotations: Specific rotations ($[\alpha]_D^t$) were measured with a Rudolph RA Autopol IV Automatic Polarimeter at the indicated temperature with a sodium lamp (sodium D line, λ = 589 nm). Measurements were performed in an acid resistant 1 mL cell (50 mm length) with concentrations (g/(100 mL)) reported in the corresponding solvent.

High Performance Liquid Chromatography: High performance liquid chromatography (HPLC) was performed on Shimadzu LC-20AD liquid chromatograph (SIL-20AC auto sampler, CMB-20A communication bus module, DGU-20A5 degasser, CTO-20AC column oven, SPD-M20A diode array detector), Shimadzu LC-20AB liquid chromatograph (SIL-20ACHT auto sampler, DGU-20A5 degasser, CTO-20AC column oven, SPD-M20A diode array detector), or Shimadzu LC-20AB liquid chromatograph (reversed phase, SIL-20ACHT auto sampler, CTO-20AC column oven, SPD-M20A diode array detector) using Daicel columns with a chiral stationary phase. All solvents used were HPLC-grade solvents purchased from Sigma-Aldrich. The column employed and respective solvent mixture are indicated for each experiment.

Abbreviations: e.r. = enantiomeric ratio, TLC = thin layer chromatography, THF = tetrahydrofuran, MTBE = methyl *tert*-butyl ether, MeCN = acetonitrile, mesitylene = 1,3,5-trimethylbenzene

2. Synthesis and Characterization of Substrates

General Procedures of Substrate (4) Preperation:

All alkenoic acid starting materials were prepared following literature reported procedures[1-4] described in Figure S1 below: All characteristic compound data have been matched with the the previously published data.

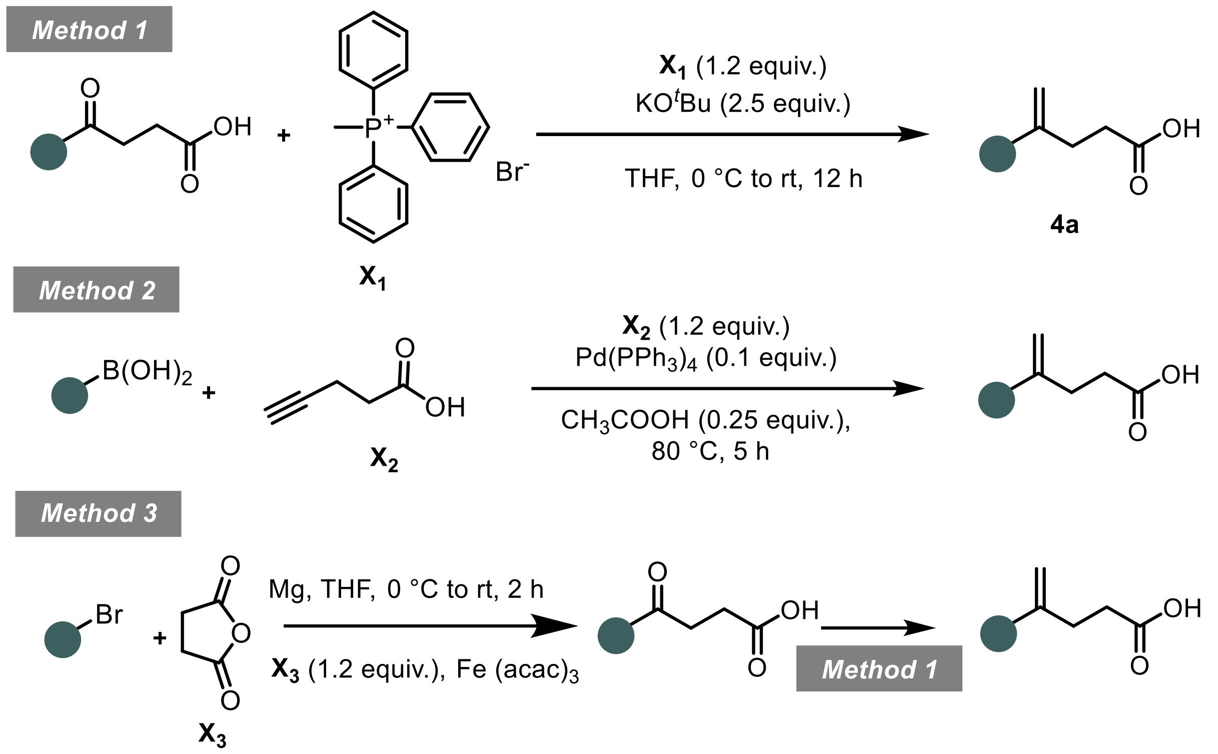

Figure *S1.* Methods to prepare alkenoic acids

Charecterization of* 4-(3-fluoro-4-methoxyphenyl)pent-4-enoic acid (4j)*:

A flame dried schlenk flask under argon was charged with triphenyl phosphoniumiodide (5.13 mg , 14.36 mmol, 1.3 equiv.) in THF (30 mL). The reaction was cooled with ice water and subsequently KO^{t}Bu (3.225 g, 28.73 mmol, 2.6 equiv.) was added portion-wise over 5 mins, and the reaction was left stirring for 30 mins. Thereafter, the corresponding keto acid (commercially available)was added (2500 mg, 11.05 mmol, 1.0 equiv.) to the reaction mixture in 3 portions followed by an additional 10 ml to rise the side of the flask. The mixture was allowed to warm up to r.t. and stirred for 16 h. The organic layer was separated and the aqueous layer was extracted with CH_2Cl_2 (3 × 50 mL). The combined organic phases were washed with sat. $NaHCO_3$ (100 mL) and brine (100 mL) and dried over anhydrous $MgSO_4$. After concentration *in vacuo,* the crude product was purified by biotage column using CH_2Cl_2 and MeOH.

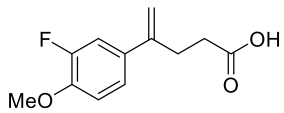

4j

obtained product 2.28 g, 92% yield, white solid.

^{1}H NMR (501 MHz, $CDCl_3$) δ 7.19 – 7.08 (m, 2H), 6.92 (t, *J* = 8.6 Hz, 1H), 5.27 (d, *J* = 0.9 Hz, 1H), 5.07 (q, *J* = 1.2 Hz, 1H), 3.90 (s, 3H), 2.83–2.73 (m, 2H), 2.53 (dd, *J* = 8.6, 6.9 Hz, 2H).

^{13}C NMR (126 MHz, $CDCl_3$) δ 178.8, 153.4, 151.4, 147.4, 145.0, 133.7, 121.9, 114.1, 112.6, 56.5, 32.9, 30.1.

^{19}F NMR (565 MHz, $CDCl_3$): -135.25

HRMS (ESI) (m/z): calculated for $C_{12}H_{13}FO_3Na$ $[M+Na]^+$: 224.0776; found 224.0775

3. Synthesis and Characterization of IDPi Catalysts

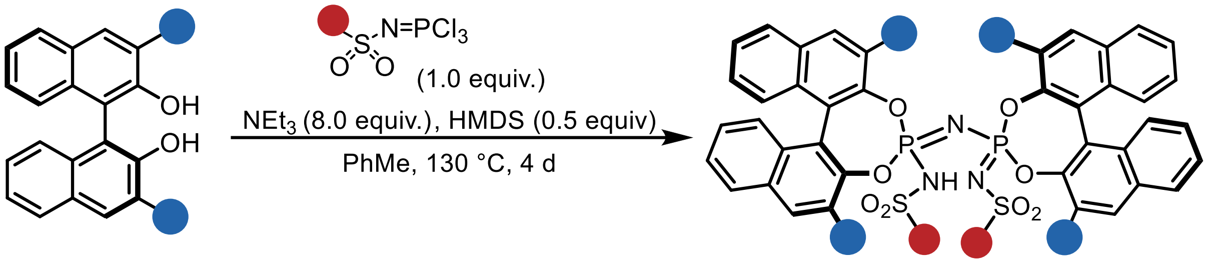

Figure S2: General procedure of IDPi synthesis

((trifluoromethyl)sulfonyl)phosphorimidoyl trichloride and IDPi Catalysts were prepared following the previously reported procedures.[4-8]

General Procedure: IDPi Synthesis

In a flame dried Schlenk equipped with a magnetic stir bar was 3,3'-disubstituted BINOL (1.01 equiv.) dissolved in PhMe (0.25 M) under argon. ((trifluoromethyl)sulfonyl)phosphorimidoyl trichloride (1.00 equiv.) was added and NEt_3 (8 equiv.) was added dropwise to form a heterogeneous mixture. The mixture was stirred at r.t. for 30 min and TLC showed full consumption of diol. Then hexamethyldisilazane (0.50 equiv.) was added and the mixture was stirred at r.t. for 30 mins, before it was heated at 130 °C for 4 d. The reaction mixture was cooled down to r.t., diluted with CH_2Cl_2 and concentrated under reduced pressure directly onto silica. The residue was purified by flash column chromatography on silica gel. The purified product was dissolved in Et_2O and passed through a plug of DOWEX® 50W X8 (hydrogen form, strongly acidic, 50–100 mesh) and concentrated under reduced pressure to afford a white solid.

Note: Before every use the DOWEX resin was pretreated with 3 column volumes each of H_2SO_4 (0.25 M), $H_2O_{dest.}$ *and Et_2O.*

Imidodiphosphorimidic acid IDPi 7g : white solid (yield 52%)

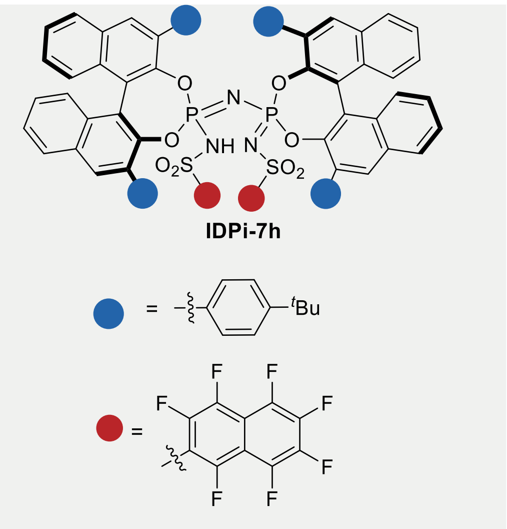

^{1}H NMR (600 MHz, $CDCl_3$) δ 8.2 (ddt, *J* = 8.2, 1.3, 0.6 Hz, 2H), 8.0 (s, 2H), 7.9 (s, 2H), 7.8 (d, *J* = 8.2 Hz, 2H), 7.7 (ddd, *J* = 8.0, 6.8, 1.0 Hz, 2H), 7.5 – 7.5 (m, 8H), 7.4 (ddd, *J* = 8.2, 6.8, 1.2 Hz, 2H), 7.4 (ddd, *J* = 8.1, 6.7, 1.1 Hz, 2H), 7.2 (d, *J* = 8.6 Hz, 2H), 7.1 (d, *J* = 8.4 Hz, 2H), 7.1 (ddd, *J* = 8.3, 6.8, 1.1 Hz, 2H), 6.8 (d, *J* = 8.4 Hz, 4H), 6.5 (d, *J* = 8.5 Hz, 4H), 5.6 (s, 1H), 1.3 (s, 18H), 0.9 (s, 18H).

^{13}C NMR (151 MHz, $CDCl_3$) δ 151.3, 150.3, 149.7 (d, *J* = 273.0 Hz), 143.8 – 143.5 (m), 143.3 (dd, *J* = 261.1, 15.6 Hz), 142.2 (d, *J* = 263.4 Hz), 140.7 (dt, *J* = 260.6, 14.7 Hz), 140.1 (d, *J* = 257.4 Hz), 138.9 (dt, *J* = 258.4, 15.4 Hz), 134.6, 133.3, 132.8, 132.6, 132.0, 131.6, 131.4, 131.3, 131.0, 130.3, 129.5, 129.1, 129.0, 128.0, 127.3, 126.8, 126.7, 126.5, 126.2, 126.2, 126.1, 124.7, 123.2, 122.6, 119.8 (t, *J* = 15.3 Hz), 112.2 – 111.7 (m), 107.4 – 107.0 (m).

^{31}P NMR (243 MHz, $CDCl_3$) δ -6.39

^{19}F NMR (565 MHz, $CDCl_3$) δ -111.41 (dd, *J* = 76.3, 15.2 Hz), -132.08 (d, *J* = 13.7 Hz), -141.24 (dt, *J* = 76.1, 15.0 Hz), -144.25 (dt, *J* = 57.8, 15.6 Hz), -146.62 (dt, *J* = 57.3, 17.5 Hz), -149.81 (t, *J* = 18.1 Hz), -154.51 (t, *J* = 18.0 Hz).

HRMS (ESI) (m/z): calculated for $C_{108}H_{82}N_3O_8S_2F_{14}P_2$ $[M-H]^-$: 1834.4027; found 1834.4018

$[\alpha]_D^{25}$ = +140.6 (*c* 0.33, $CHCl_3$)

Additional HSQC, HMBC, COSY and ROESY analysis has been performed to ascertain connectivity (see spectra in S10)

4. Reaction Development and Optimization

A flame dried 1.5 mL screw-cap vial was charged with 5 mol% catalyst. Dry solvent and alkenoic acids **4a** (0.05 mmol, 1.0 equiv.) and anisole (as internal standard) were added. The solution was stirred under intert condition for 5 days reaction. Thereafter triethyl amine was added. Subsequently, an aliquot of the mixture was taken to determine the yield and conversion by ^{1}H-NMR in $CDCl_3$. E.r. were determined prep. TLC followed by HPLC on a chiral stationary phase.

Table S1. Screening to identify a suitable Chiral Catalyst for asymmetric hydrolactonization

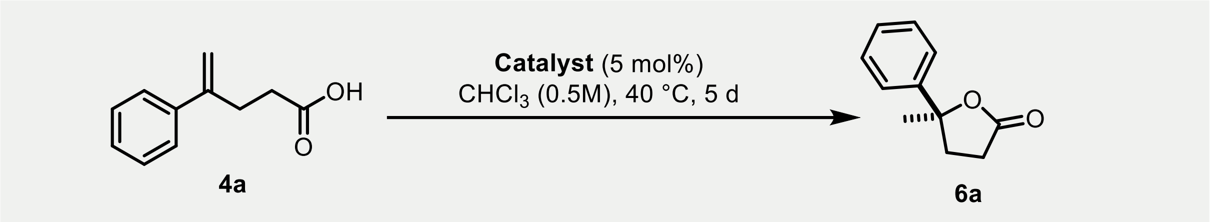

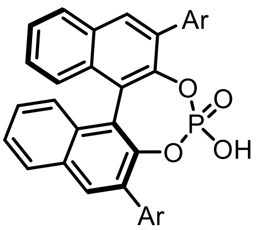

(*S*)-Chiral Phosphoric Acid **(CPA)**
Ar= Ph
no reaction

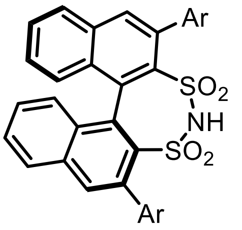

(*S*)-Disulfonimide **(DSI)**
Ar = 3,5-$(CF_3)_2C_6H_3$
25% conversion, e.r. 50.5:49.5

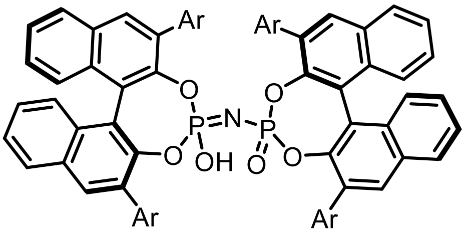

(*S,S*)-Imidodiphosphoric acid **(IDP)**
Ar = Ph
no reaction

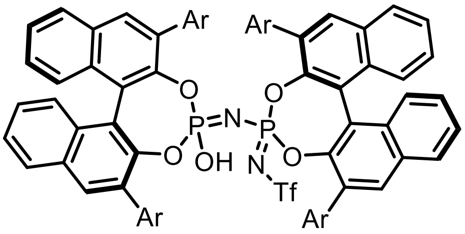

(*S,S*)-Imidodiphosphoric acid **(iIDP)**
Ar = Ph
no reaction

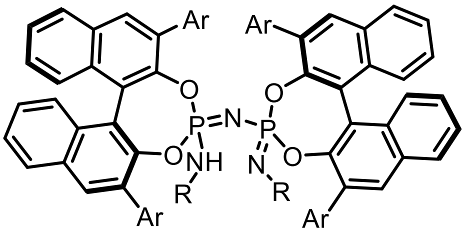

(*S*,S)-7: IDPi
(*S,S*)-Imidodiphosphorimidic acid
Ar = 4-^{t}Bu-C_6H_4, R = Tf
full conversion, e.r. 67:33

Entry	Catalyst	T (°C)	Solvent	Conv. (%)[b]	e.r.[c]
1	**CPA**	60	$CHCl_3$	NR	-
2	**DSI**	60	$CHCl_3$	25	50.5:49.5
3	**IDP**	60	$CHCl_3$	NR	-
4	**iIDP**	60	$CHCl_3$	NR	-
5	**IDPI-7b**	60	$CHCl_3$	>95	68:32

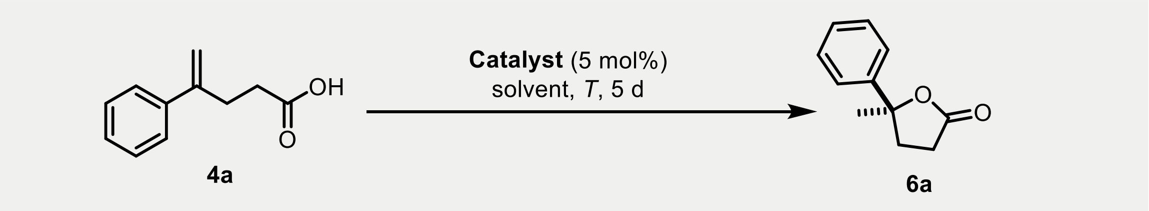

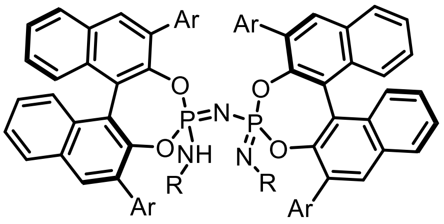

(*S*,S) IDPi 7a
Ar = 4-^{t}Bu-C_6H_4, R = 3,5-$(CF_3)_2C_6H_3$
No reaction @ 60° C

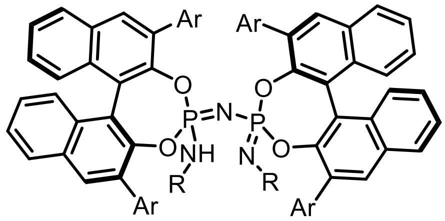

(*S*,S) IDPi 7b
Ar = 4-^{t}Bu-C_6H_4, R = Tf
full conversion @60° C, e.r. 67:33

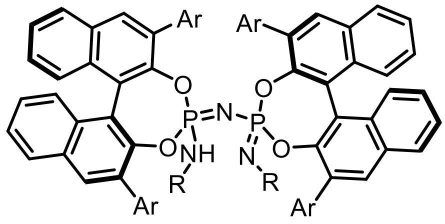

(*S,S*) IDPi 7c

Ar = Ph, R = Tf
full conversion-@40° C, e.r. 50.5:49.5

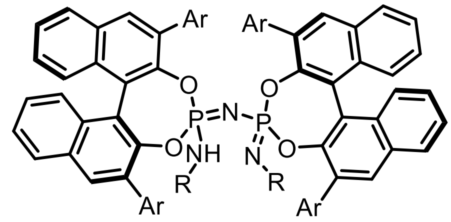

(*S*,S) IDPi 7d
Ar = 4-SF_5-C_6H_4, R = Tf
full conversion-@40° C, e.r. 51.5:48.5

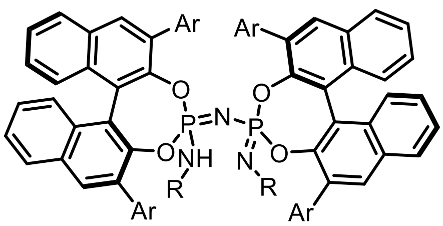

(*S*,S) IDPi 7e
Ar = 3-Ph-C_6H_4, R = Tf
full conversion-@40° C, e.r. 53:47

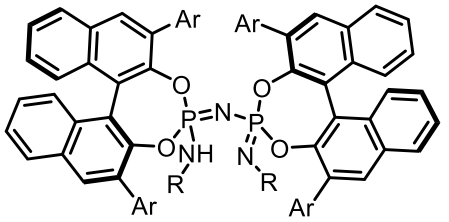

(*S*,S) IDPi 7f
Ar = 2-napthyl, R = Tf
full conversion-@40° C, e.r. 55:45

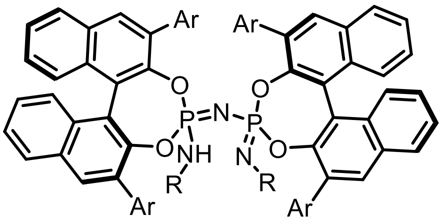

(*S*,S) IDPi 7g

Ar = 4-^{t}Bu-C_6H_4, R = $SO_2C_6F_5$
full conversion-@40° C, e.r. 93:7

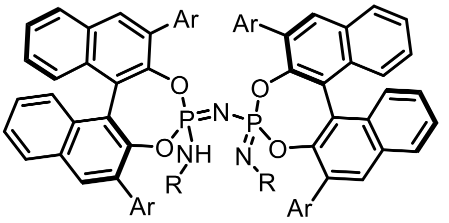

(*S*,S) IDPi 7h
Ar = 4-^{t}Bu-C_6H_4, R = $SO_2C_{10}F_7$
full conversion-@40° C, e.r. 95:5

Figure S3. Screening to Identify suitable IDPi Catalysts for asymmetric hydrolactonization

While performing initial optimization, we undertook a comparative analysis of asymmetric hydro lactonization with our previously reported asymmetric hydroalkoxylation. We noted, in presence of catalyst 7c at 60°C, carboxylic acid 4a is more reactive than analogous alcohol, although the observed selectivity for the asymmetric hydrolactonization has been significantly less compared to the asymmetric hydroalkoxylation (e.r 50.5:49.5 vs 63.5: 36.5).

Table S2. *Solvent Screening for the asymmetric organocatalytic hydrolactonization*

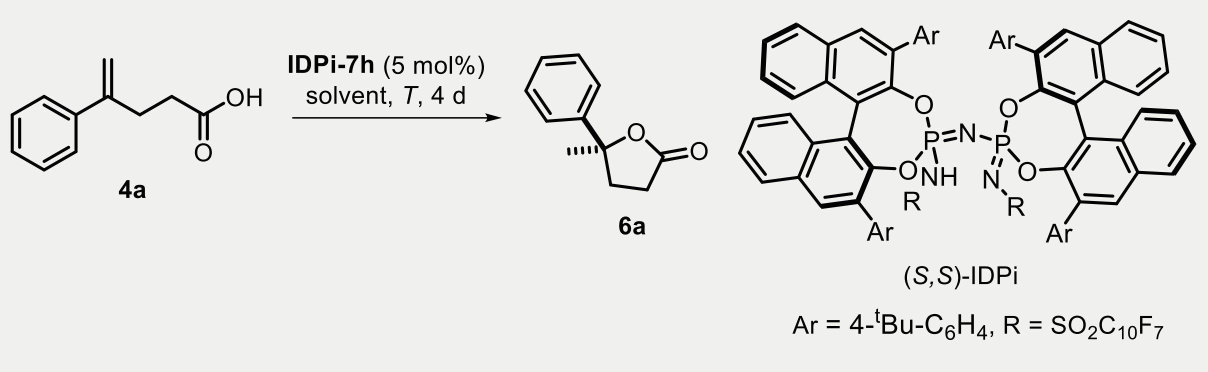

Catalyst	T (°C)	Solvent (0.5M)	Conv. (%)[b]	e.r.[c]
**7h**	40	$CHCl_3$	>95	95:5
**7h**	40	DCM	>95	94:6
**7h**	40	MTBD	>95	78:22
**7h**	40	Diethyl ether	>95	89.5:10.5
**7h**	40	Acetone	>95	73:27
**7h**	40	Cyclohexane	50	75.5:24.5
**7h**	40	m-Xylene	75	93:7
**7h**	40	Toulene	75	95:5
**7h**	40	Benzene	75	93.5:6.5

5. Organocatalytic Asymmetric Hydrolactonization and its Application

General Procedure for the Enantioselective hydrolactonization reaction:

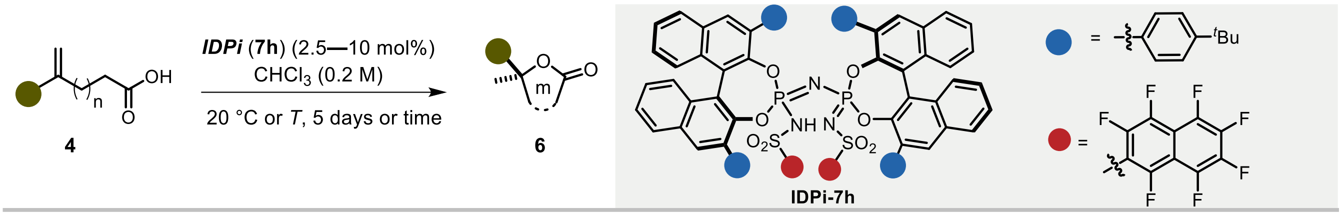

***Figure S4*: General procedure of enantioselective hydrolactonization**

A 5 mL flame dried screw-cap vial, equipped with a magnetic stirring bar, was charged with **IDPi-7h**. Dry solvent (0.2 M) and acid **4** (0.1 mmol, 1.0 equiv.) were added. The reaction was stirred for 1– 8 days at ambient temperature before adding *wet* triethylamine (0.5 equiv.). The mixture was diluted with water (10 mL), extracted with CH_2Cl_2(8mL × 3), dried (MgSO4), and concentrated in vacuo. The residue was purified by flash column chromatography (Hexanes/EtOAc 20:1) to yield the corresponding chiral lactone.

Racemate synthesis: The racemic product was synthesized at 60 °C for 24 h by using acidic Amberlyst as the catalyst (instead of **IDPi**)in $CHCl_3$.

Charecterization of enantiopure products:

(*S*)-5-methyl-5-phenyldihydrofuran-2(3H)-one (6a)

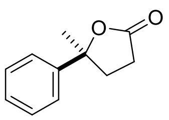

0.1 mmol s.m. with 5 mol% cat **7g** dissolved in 0.2 M $CHCl_3$ at 20 °C for 5 d obtain 15.3 mg, 87% yield, colorless solid, e.r. 96:4.

^{1}H NMR (501 MHz, $CDCl_3$) δ 7.30 – 7.13 (m, 5H), 2.55 – 2.47 (m, 1H), 2.44 – 2.34 (m, 2H), 2.34 – 2.24 (m, 1H), 1.61 (s, 3H).

^{13}C NMR (126 MHz, $CDCl_3$) δ 176.6, 144.5, 128.8, 127.8, 124.3, 87.1, 36.3, 29.6, 29.1.

HRMS (ESI) (m/z): calculated for $C_{11}H_{12}O_2$ $[M+Na]^+$:199.073110; found : 199.072949

The enantiomeric ratio was measured by HPLC analysis using following parameters: Daicel Chiralcel IC-3 column: *n*Hept : *i*PrOH = 95:5, flow rate 1.0 mL/ min, t_{major} = 16.7 min, t_{minor} = 18.7 min

$[\alpha]_D^{25}$ = –22.69 (*c* 0.29, $CHCl_3$)

(*S*)-5-(4-methoxyphenyl)-5-methyldihydrofuran-2(3H)-one (6b)

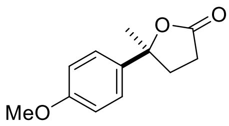

0.1 mmol s.m. with 5 mol% cat **7g** dissolved in 0.1 M $CHCl_3$ at 0 °C for 2 d, obtain 19.69 mg, 96% yield, colorless liquid, e.r. 97.5:2.5.

^{1}H NMR (501 MHz, CD_2Cl_2) δ 7.32 – 7.23 (m, 2H), 6.93 – 6.84 (m, 2H), 3.78 (s, 3H), 2.66 – 2.53 (m, 1H), 2.50 – 2.40 (m, 2H), 2.40 – 2.32 (m, 1H), 1.66 (s, 3H).

^{13}C NMR (126 MHz, CD_2Cl_2) δ 176.6, 159.4, 136.9, 125.8, 114.2, 87.1, 55.6, 36.4, 29.5, 29.4.

HRMS (ESI) (m/z): calculated for $C_{12}H_{14}O_3$ $[M+Na]^+$: 229.0843; found : 229.0845

The enantiomeric ratio was measured by HPLC analysis using following parameters: Daicel Chiralcel IC-3 column: *n*Hept : *i*PrOH = 90:10, flow rate 1.0 mL/ min, t_{major} = 18.1 min, t_{minor} = 21.3 min

$[\alpha]_D^{25}$ = –58.5 (*c* 0.24, $CHCl_3$)

(*S*)-5-methyl-5-(4-(methylthio)phenyl)dihydrofuran-2(3H)-one (6c)

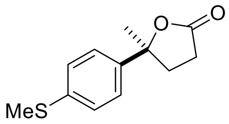

0.1 mmol s.m. with 5 mol% cat **7g** dissolved in 0.1 M $CHCl_3$ at 0 °C for 3 d, obtain 21.4 mg, 95% yield, colorless solid, e.r. 98:2

^{1}H NMR (501 MHz, $CDCl_3$) δ 7.32 – 7.19 (m, 4H), 2.65 – 2.56 (m, 1H), 2.50 (dd, *J* = 8.8, 7.9 Hz, 1H), 2.46 (s, 3H), 2.45 – 2.40 (m, 1H), 2.40 – 2.33 (m, 1H), 1.68 (s, 3H).

^{13}C NMR (126 MHz, $CDCl_3$) δ 176.6, 141.3, 138.3, 126.8, 124.9, 86.9, 36.3, 29.5, 29.2, 15.9

The enantiomeric ratio was measured by HPLC analysis using following parameters: Daicel Chiralcel IC-3 column: *n*Hept : *i*PrOH = 90:10, flow rate 1.0 mL/ min, t_{major} = 17.4 min, t_{minor} = 20.7 min

HRMS (ESI) (m/z): calculated for $C_{12}H_{14}O_2S$ $[M]^+$: 222.0715; found : 222.0821
[α]$_D^{25}$ = –34.7 (*c* 0.46, $CHCl_3$)

(*S*)-5-([1,1'-biphenyl]-4-yl)-5-methyldihydrofuran-2(3H)-one (6d)

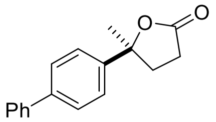

0.1 mmol s.m. with 5 mol% cat **7g** dissolved in 0.2 M $CHCl_3$ at 20 °C for 5 d, obtain 23.17 mg, 94% yield, colorless solid, e.r. 97:3.
^{1}H NMR (501 MHz, $CDCl_3$) δ 7.67–7.55 (m, 4H), 7.50–7.41 (m, 4H), 7.39–7.31 (m, 1H), 2.70–2.61 (m, 1H), 2.61–2.50 (m, 2H), 2.50–2.39 (m, 1H), 1.77 (s, 3H).
^{13}C NMR (126 MHz, $CDCl_3$) δ 176.6, 143.4, 140.8, 140.6, 129.0, 127.6, 127.5, 127.2, 124.8, 87.0, 36.4, 29.6, 29.4.
HRMS (ESI) (m/z): calculated for $C_{17}H_{16}O_2$ $[M]^+$: 252.1146; found : 252.1144
The enantiomeric ratio was measured by HPLC analysis using following parameters: Daicel Chiralcel IC-3 column: *n*Hept : *i*PrOH = 95:5, flow rate 1.0 mL/ min, t_{major} = 23.9 min, t_{minor} = 28.5 min
[α]$_D^{25}$ = –9.96 (*c* 0.16, $CHCl_3$)

(*S*)-5-(4-fluorophenyl)-5-methyldihydrofuran-2(3H)-one (6e)

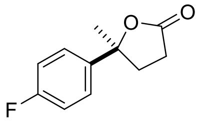

0.1 mmol s.m. with 5 mol% cat **7g** dissolved in 0.2 M $CHCl_3$ at 20 °C for 5 d, obtain 17.86 mg, 92% yield, colorless solid, e.r. 94:6
^{1}H NMR (501 MHz, $CDCl_3$) δ 7.40–7.31 (m, 2H), 7.10–7.00 (m, 2H), 2.70–2.59 (m, 1H), 2.54–2.32 (m, 3H), 1.70 (s, 3H).
^{13}C NMR (126 MHz, $CDCl_3$) δ 176.4, 162.2 (d, *J* = 246.4 Hz), 140.3(d, *J* = 3.2 Hz), 126.0(d, *J* = 8.4 Hz), 115.7(d, *J* = 21.7 Hz), 86.7, 36.3, 29.6, 29.0.
^{19}F NMR (565 MHz, $CDCl_3$):-114.69
HRMS (ESI) (m/z): calculated for $C_{11}H_{11}O_2FNa$ $[M+Na]^+$: 217.06358; found : 217.06352
The enantiomeric ratio was measured by HPLC analysis using following parameters: Daicel Chiralcel IC-3 column: *n*Hept : *i*PrOH = 95:5, flow rate 1.0 mL/ min, t_{major} = 16.3 min, t_{minor} = 18.4 min
[α]$_D^{25}$ = –30.56 (*c* 0.38, $CHCl_3$)

(*S*)-5-(4-chlorophenyl)-5-methyldihydrofuran-2(3H)-one (6f)
0.1 mmol s.m. with 5 mol% cat **7g** dissolved in 0.2 M Toluene at 40 °C for 5 d, obtain 20.01 mg, 95% yield, colorless liquid, e.r. 93.5:6.5.

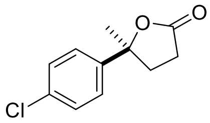

^{1}H NMR (501 MHz, $CDCl_3$) δ 7.34 – 7.30 (m, 4H), 2.66 – 2.61 (m, 1H), 2.55 – 2.36 (m, 3H), 1.70 (s, 3H).
^{13}C NMR (126 MHz, $CDCl_3$) δ 176.2, 143.0, 133.7, 128.9, 125.8, 86.5, 36.2, 29.5, 29.0.
HRMS (ESI) (m/z): calculated for $C_{11}H_{11}O_2Cl$ $[M]^+$: 210.0448; found : 210.044208
The enantiomeric ratio was measured by HPLC analysis using following parameters: Daicel Chiralcel IC-3 column: *n*Hept : *i*PrOH = 95:5, flow rate 1.0 mL/ min, t_{major} = 15.4 min, t_{minor} = 18.1 min
[α]$_D^{25}$ = –34.2 (*c* 0.26, $CHCl_3$)

(*S*)-5-(4-bromophenyl)-5-methyldihydrofuran-2(3H)-one (**6g**)

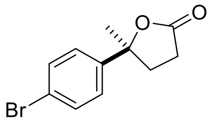

0.1 mmol s.m. with 5 mol% cat **7g** dissolved in 0.2 M Toluene at 40 °C for 5 d, obtain 21.42 mg, 84% yield (at 40 °C), colorless liquid, e.r. 94.5:5.5.
^{1}H NMR (501 MHz, $CDCl_3$) δ 7.49 – 7.40 (m, 2H), 7.24 – 7.12 (m, 2H), 2.61– 2.55 (dtd, *J* = 10.0, 7.9, 7.3, 4.9 Hz, 1H), 2.50 – 2.27 (m, 3H), 1.68 – 1.61 (m, 3H).
^{13}C NMR (126 MHz, $CDCl_3$) δ 176.2, 143.6, 131.9, 126.1, 121.8, 86.6, 36.2, 29.4, 29.0.
HRMS (ESI) (m/z): calculated for $C_{11}H_{11}O_2Br$ $[M]^+$: 253.9936; found : 253.9937
The enantiomeric ratio was measured by HPLC analysis using following parameters: Daicel Chiralcel IC-3 column: *n*Hept : *i*PrOH = 95:5, flow rate 1.0 mL/ min, t_{major} = 16.0 min, t_{minor} = 19.1 min
[α]$_D^{25}$ = –23.8 (*c* 0.21, $CHCl_3$)

(*S*)-5-methyl-5-(m-tolyl)dihydrofuran-2(3H)-one (6h)

0.1 mmol s.m. with 5 mol% cat **7g** dissolved in 0.2 M Toluene at 40 °C for 5 d, obtain 17.88 mg, 94% yield, colorless liquid, e.r. 96:4

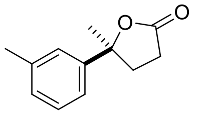

^{1}H NMR (501 MHz, $CDCl_3$) δ 7.26 (t, *J* = 7.6 Hz, 1H), 7.19 (d, *J* = 1.8 Hz, 1H), 7.17 – 7.07 (m, 2H), 2.70–2.58 (m, 1H), 2.56–2.45 (m, 2H), 2.45–2.38 (m, 1H), 2.36 (s, 3H), 1.71 (s, 3H).

^{13}C NMR (126 MHz, $CDCl_3$) δ 176.8, 144.5, 138.5, 128.7, 124.9, 121.3, 87.2, 36.4, 29.7, 29.2, 21.7.

HRMS (ESI) (m/z): calculated for $C_{12}H_{14}O_2Na$ $[M+Na]^+$: 213.0889 found : 213.088599

The enantiomeric ratio was measured by HPLC analysis using following parameters: Daicel Chiralcel IC-3 column: *n*Hept : *i*PrOH = 95:5, flow rate 1.0 mL/ min, t_{major} = 15.6 min, t_{minor} = 17.5 min

$[\alpha]_D^{25}$ = +32.85 (*c* 0.14, $CHCl_3$)

(*S*)-5-(3-fluorophenyl)-5-methyldihydrofuran-2(3H)-one (6i)

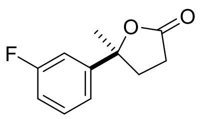

0.1 mmol s.m. with 5 mol% cat **7g** dissolved in 0.2 M Toluene at 40 °C for 5 d, obtain 16.11 mg, 85% yield, colorless liquid, e.r. 97.5:2.5.

^{1}H NMR (501 MHz, CD_2Cl_2) δ 7.37 (td, *J* = 8.0, 6.0 Hz, 1H), 7.20 – 6.96 (m, 3H), 2.66 – 2.56 (m, 1H), 2.53 – 2.35 (m, 3H), 1.69 (s, 3H).

^{13}C NMR (126 MHz, CD_2Cl_2) δ 176.2, 162.8 (d, *J* = 245.8 Hz), 147.8 (d, *J* = 6.9 Hz),130.7 (d, *J* = 8.4 Hz),120.3 (d, *J* = 3.1 Hz),114.7 (d, *J* = 21.2 Hz), 111.8 (d, *J* = 23.0 Hz), 86.5(d, *J* = 2.2 Hz),, 36.3, 29.3, 29.1.

^{19}F NMR (565 MHz, CD_2Cl_2): -113.17

HRMS (ESI) (m/z): calculated for $C_{11}H_{11}O_4FNa$ $[M+Na]^+$: 217.063460; found : 217.063528

The enantiomeric ratio was measured by HPLC analysis using following parameters: Daicel Chiralcel IC-3 column: *n*Hept : *i*PrOH = 95:5, flow rate 1.0 mL/ min, t_{major} = 14.4 min, t_{minor} = 16.7 min

$[\alpha]_D^{25}$ = +18.46 (*c* 0.13, $CHCl_3$)

(*S*)-5-(3-fluoro-4-methoxyphenyl)-5-methyldihydrofuran-2(3H)-one (6j)

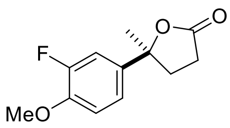

0.11 mmol, s.m. 22.4 mg, using 2.5 mol% of catalyst for 4 days, obtain 21.97 mg, 98% yield, white solid, e.r. 96.5:3.5.

0.10 mmol, s.m. 21.8 mg, using 5 mol% of catalyst for 2 days, obtain 21.52 mg, 97% yield, white solid, e.r. 96.5:3.5.

(also performed in gram scale with 2.5 mol% catalyst loading and catalyst recovered 95% by column chromatography and reused after acidification)

^{1}H NMR (501 MHz, $CDCl_3$) δ 7.14 – 7.05 (m, 2H), 6.94 (t, *J* = 8.6 Hz, 1H), 3.89 (s, 3H), 2.63 (ddd, *J* = 17.0, 9.1, 5.6 Hz, 1H), 2.54 – 2.34 (m, 3H), 1.68 (s, 3H).

^{13}C NMR (126 MHz, $CDCl_3$) δ 176.4, 152.4 (d, *J* = 245.6 Hz), 147.1(d, *J* = 7.8 Hz), 137.6(d, *J* = 8.4 Hz), 120.1(d, *J* = 3.7 Hz),113.7 (d, *J* = 20.8 Hz),112.8 (d, *J* = 24.3 Hz), 86.4 (d, *J* = 3.8 Hz), 56.5 (d, *J* = 12.4 Hz), 36.3, 29.5, 29.1.

^{19}F NMR (565 MHz, $CDCl_3$) -133.93

HRMS (ESI) (m/z): calculated for $C_{12}H_{13}O_3F$ $[M]^+$:224.0849 ; found : 224.084323

The enantiomeric ratio was measured by HPLC analysis using following parameters: Daicel Chiralcel IC-3 column: *n*Hept : *i*PrOH = 90:10, flow rate 1.0 mL/ min, t_{major} = 20.2 min, t_{minor} = 27.9 min

$[\alpha]_D^{25}$ = –37.9 (*c* 0.153, $CHCl_3$)

(*S*)-5-(2,3-dihydrobenzo[b][1,4]dioxin-6-yl)-5-methyldihydrofuran-2(3H)-one (6k)

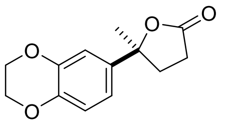

0.1 mmol s.m. with 5 mol% cat **7g** dissolved in 0.2 M Toluene at 40 °C for 2 d, obtain 24.9 mg, 97% yield, colorless solid, e.r. 94:6.

^{1}H NMR (501 MHz, $CDCl_3$) δ 6.88 (d, *J* = 2.0 Hz, 1H), 6.84 – 6.81 (m, 2H), 4.25 (s, 4H), 2.60 (ddd, *J* = 16.2, 9.2, 5.0 Hz, 1H), 2.55 – 2.39 (m, 2H), 2.36 – 2.32 (m, 1H), 1.67 (s, 3H).

^{13}C NMR (126 MHz, $CDCl_3$) δ 176.6, 143.5, 143.1, 137.7, 117.4, 117.3, 113.6, 86.8, 64.5, 36.3, 29.5, 29.2.
HRMS (ESI) (m/z): calculated for $C_{13}H_{14}O_4$ $[M]^+$: 234.0887; found : 234.0890
The enantiomeric ratio was measured by HPLC analysis using following parameters: Daicel Chiralcel IC-3 column: *n*Hept : *i*PrOH = 85:15, flow rate 0.7mL/ min, t_{major} = 28.3 min, t_{minor} = 35.8 min
$[\alpha]_D^{25}$ = –41.6 (*c* 0.26, $CHCl_3$)

(*S*)-5-(2,4-dimethylphenyl)-5-methyldihydrofuran-2(3H)-one (6l)

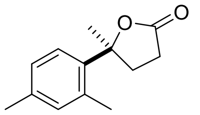

0.1 mmol s.m. with 10 mol% cat **7g** dissolved in 0.5 M Toluene at 40 °C for 8 d, obtain 15.11 mg, 74% yield, colorless solid, e.r. 93:7.
^{1}H NMR (501 MHz, CD_2Cl_2) δ 7.30 (d, *J* = 8.0 Hz, 1H), 7.06 – 6.95 (m, 2H), 2.69 – 2.60 (m, 1H), 2.59 – 2.52 (m, 1H), 2.51 – 2.45 (m, 2H), 2.40 (s, 3H), 2.29 (s, 3H), 1.70 (s, 3H).
^{13}C NMR (126 MHz, CD_2Cl_2) δ 176.3, 139.7, 137.7, 134.1, 133.5, 126.8, 125.0, 88.1, 35.4, 29.1, 28.0, 21.5, 20.8.
HRMS (ESI) (m/z): calculated for $C_{13}H_{16}O_2$ $[M]^+$: 204.1150; found : 204.11448
The enantiomeric ratio was measured by HPLC analysis using following parameters: Daicel Chiralcel IC-3 column: *n*Hept : *i*PrOH = 95:5, flow rate 1.0 mL/ min, t_{major} = 17.9 min, t_{minor} = 20.7 min
$[\alpha]_D^{25}$ = +9.2 (*c* 0.24, $CHCl_3$)

(*S*)-5-(2,4-difluorophenyl)-5-methyldihydrofuran-2(3H)-one (6m)

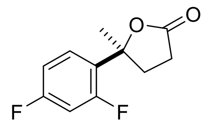

0.1 mmol s.m. with 10 mol% cat **7g** dissolved in 0.5 M Toluene at 40 °C for 8 d, obtain 17.18 mg, 81% yield, colorless liquid, e.r. 94:6.
^{1}H NMR (501 MHz, CD_2Cl_2) δ 7.47 (td, *J* = 8.9, 6.4 Hz, 1H), 6.98 – 6.75 (m, 2H), 2.68 – 2.59 (m, 1H), 2.55 – 2.43 (m, 3H), 1.72 (d, *J* = 1.1 Hz, 3H).
^{13}C NMR (126 MHz, CD_2Cl_2) δ 176.1,162.5 (dd, *J* = 248.4, 12.3 Hz),158.9 (dd, *J* = 246.5, 10.4 Hz),, 128.2 (dd, *J* =13.6, 7.4) Hz), 127.5 (dd, *J* = 9.7, 5.5 Hz),111.5 (dd, *J* = 20.7, 3.6 Hz),104.9 (t, *J* = 26.0 Hz), 84.8 (d, *J* = 3.2 Hz), 35.1 (d, *J* = 4.2 Hz), 28.9, 27.6 (d, *J* = 2.9 Hz).
^{19}F NMR (565 MHz, CD_2Cl_2) -110.29 and -111.93
HRMS (ESI) (m/z): calculated for $C_{11}H_{10}O_2F_2$ $[M+H]^+$: 213.072540; found : 213.072162
The enantiomeric ratio was measured by HPLC analysis using following parameters: Daicel Chiralcel IC-3 column: *n*Hept : *i*PrOH = 95:5, flow rate 1.0 mL/ min, t_{major} = 10.6 min, t_{minor} = 11.4 min
$[\alpha]_D^{25}$ = –52.9 (*c* 0.234, $CHCl_3$)

(*S*)-5-methyl-5-(naphthalen-2-yl)dihydrofuran-2(3H)-one (6n)

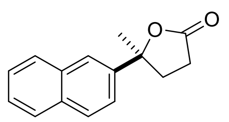

0.1 mmol s.m. with 5 mol% cat **7g** dissolved in 0.2 M Toluene at 20 °C for 5 d, obtain 21.49 mg, 95% yield, white solid, e.r. 96:4.
^{1}H NMR (501 MHz, $CDCl_3$) δ 7.96–7.77 (m, 4H), 7.57–7.47 (m, 2H), 7.44 (dd, *J* = 8.6, 1.9 Hz, 1H), 2.74 – 2.40 (m, 4H), 1.81 (s, 3H).
^{13}C NMR (126 MHz, $CDCl_3$) δ 176.7, 141.6, 133.1, 132.7, 128.8, 128.3, 127.7, 126.7, 126.5, 122.8, 122.6, 87.2, 36.2, 29.5, 29.1.
HRMS (ESI) (m/z): calculated for $C_{15}H_{14}O_2$ $[M]^+$: 226.0994; found : 226.098830
The enantiomeric ratio was measured by HPLC analysis using following parameters: Daicel Chiralcel IC-3 column: *n*Hept : *i*PrOH = 95:5, flow rate 1.0 mL/ min, t_{major} = 19.4 min, t_{minor} = 25.2 min
$[\alpha]_D^{25}$ = –19.4 (*c* 0.12, $CHCl_3$)

(*S*)-5-methyl-5-(1-(phenylsulfonyl)-1H-pyrrol-3-yl)dihydrofuran-2(3H)-one (6o)

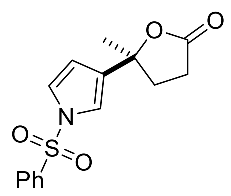

0.11 mmol s.m. with 5 mol% cat **7g** dissolved in 0.1 M $CHCl_3$ at 20 °C for 3 d, obtain 27.5 mg, 90% yield, colorless liq, e.r. 95.5:4.5.
^{1}H NMR (501 MHz, CD_2Cl_2) δ 7.95–7.85 (m, 2H), 7.66 (t, *J* = 1.2 Hz, 1H), 7.58–7.51 (m, 2H), 7.16 (dd, *J* = 3.3, 2.3 Hz, 1H), 7.12 (t, *J* = 2.0 Hz, 1H), 6.28 (dd, *J* = 3.3, 1.7 Hz, 1H), 2.66–2.43 (m, 2H), 2.34 (ddd, *J* = 12.8, 9.0, 6.2 Hz, 2H), 2.23 (ddd, *J* = 12.8, 9.3, 7.8 Hz, 2H), 1.63 (s, 3H).

^{13}C NMR (126 MHz, CD_2Cl_2) δ 176.4, 139.1, 134.6, 133.5, 130.0, 127.3, 122.2, 116.7, 111.7, 83.7, 35.7, 29.4, 28.2.
HRMS (ESI) (m/z): calculated for $C_{15}H_{15}O_4NS$ $[M]^+$: 305.0716; found : 305.0719.
The enantiomeric ratio was measured by HPLC analysis using following parameters: Daicel Chiralpak IC-3 column: *n*Hept:*i*PrOH = 70:30, flow rate 1.0 mL/ min, t_{major} = 18.5 min, t_{minor} = 24.1 min.
$[\alpha]_D^{20}$ = +18.5 (*c* 0.4, CH_2Cl_2)

(*S*)-6-methyl-6-phenyltetrahydro-2H-pyran-2-one (6p)

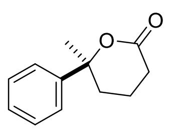

0.1 mmol s.m. with 5 mol% cat **7g** dissolved in 0.5 M Toluene at 20 °C for 8 d, obtain 13.88 mg, 73% yield, colorless liquid, e.r. 94.5:5.5.
^{1}H NMR (501 MHz, $CDCl_3$) δ 7.35–7.23 (m, 4H), 7.23–7.18 (m, 1H), 2.46–2.31 (m, 2H), 2.25 (dt, *J* = 14.3, 4.8 Hz, 1H), 1.93 (ddd, *J* = 14.7, 11.4, 4.3 Hz, 1H), 1.74–1.69 (m, 1H), 1.61 (s, 3H), 1.56–1.44 (m, 1H).
^{13}C NMR (126 MHz, $CDCl_3$) δ 171.7, 144.7, 131.9, 128.8, 127.5, 126.1, 124.5, 85.5, 34.4, 31.4, 29.2, 16.7.
HRMS (ESI) (m/z): calculated for $C_{12}H_{14}O_2$ $[M]^+$: 190.0994; found : 190.0988
The enantiomeric ratio was measured by HPLC analysis using following parameters: Daicel Chiralcel IC-3 column: *n*Hept : *i*PrOH = 97:3, flow rate 1.0 mL/ min, t_{major} = 21.6 min, t_{minor} = 26.3 min
$[\alpha]_D^{25}$ = -33.9 (*c* 0.45, $CHCl_3$)

(*S*)-5-benzyl-5-methyldihydrofuran-2(3H)-one (6q)

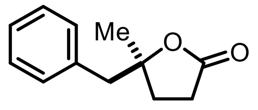

Prepared according to the general procedure in 0.1 mmol scale, using toluene as solvent, and at 40 °C for 8 d, purified by flash column chromatography using 20-30% Et_2O in hexane as eluents, The desired product was obtained as colorless oil (18 mg, 95% yield, **91:9 e.r.**)
^{1}H NMR (501 MHz, $CDCl_3$) δ 7.33 – 7.27 (m, 3H), 7.25 – 7.23 (m, 2H), 3.02 (d, *J* = 13.9 Hz, 1H), 2.88 (d, *J* = 14.0 Hz, 1H), 2.44 (ddd, *J* = 17.6, 10.0, 6.1 Hz, 1H), 2.20 (ddd, *J* = 12.6, 9.8, 6.1 Hz, 1H), 2.09–2.02 (m, 1H), 1.98–1.92 (m, , 1H), 1.44 (s, 3H);
^{13}C NMR (126 MHz, $CDCl_3$) δ 176.9, 135.8, 130.6, 128.6, 127.2, 86.4, 47.0, 32.3, 29.4, 27.2;
HRMS (GC-EI) (m/z) calculated for $C_{12}H_{14}O_2$ $[M]^+$: 190.098830, found: 190.099070.
$[\alpha]_D^{25}$ = 36.38 (*c* 0.11, $CHCl_3$)

(*R*)-5-methyl-5-phenethyldihydrofuran-2(3H)-one (6r)

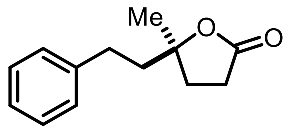

Prepared according to the general procedure (5 mol% cat **7g**) in 0.1 mmol scale, using toluene as solvent, and at 20 °C for 14 d, purified by flash column chromatography using 20-30% Et_2O in hexane as eluents, The desired product was obtained as colorless oil (30 mg, 73% yield, **91.5:8.5 e.r.**)
^{1}H NMR (501 MHz, $CDCl_3$) ^{1}H NMR (501 MHz, $CDCl_3$) δ 7.32 – 7.21 (m, 2H), 7.23 – 7.18 (m, 3H), 2.77 – 2.68 (m, 2H), 2.68 – 2.59 (m, 2H), 2.18–2.12 (m, 1H), 2.08 – 2.02 (m, 1H), 2.01 – 1.93 (m, 2H), 1.47 (s, 3H);
^{13}C NMR (126 MHz, $CDCl_3$) δ 176.9, 141.4, 128.7, 128.4, 126.3, 86.5, 43.1, 33.3, 30.4, 29.2, 25.8;
HRMS (GC-EI) (m/z) calculated for $C_{13}H_{16}O_2$ $[M]^+$: 204.114480, found: 204.114420
$[\alpha]_D^{25}$ = -11.7 (*c* 0.27, $CHCl_3$)

(*S*)-5-cyclohexyl-5-methyldihydrofuran-2(3H)-one (6s)

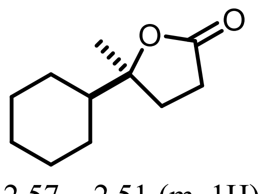

Prepared according to the general procedure in 0.2 mmol scale, using toluene (0.2 M) as solvent and at 10 °C for 17 d, purified by flash column chromatography using 15-25% Et2O in hexane as eluents, The desired product was obtained as colorless oil (19 mg, 52% yield, **97:3 e.r.**);
^{1}H NMR (501 MHz, $CDCl_3$) ^{1}H NMR (501 MHz, $CDCl_3$) δ 2.67 – 2.60 (m, 1H), 2.57 – 2.51 (m, 1H), 2.15 – 2.08 (m, 1H), 1.92 – 1.87 (m, 1H), 1.87 – 1.75 (m, 3H), 1.72 – 1.67 (m, 2H), 1.53 (tt, *J* = 12.1, 3.0 Hz, 1H), 1.30 (s, 3H), 1.28 – 1.03 (m, 4H), 1.02 – 0.91 (m, 1H);

^{13}C NMR (126 MHz, CDCl$_3$) δ 177.3, 89.7, 47.6, 31.5, 29.3, 27.3, 27.2, 26.4, 26.3, 26.2, 22.6;
HRMS (ESIpos) calculated for $C_{11}H_{18}O_2$ Na [M+Na]$^+$: 205.119899, found: 205.119790.
Enantiomeric ratio was determine by GC analysis Column: 30 m Cyclosil B, Temperature: 220/140 5/min170 40min iso/350; Gas: 0,50 bar H_2., t_R (major) = 33.0 min., t_R (minor) = 34.0 min.
[α]$_D$^{25} = -6.1 (*c* 0.23, $CHCl_3$)

(R)-5-butyl-5-methyldihydrofuran-2(3H)-one (6t)

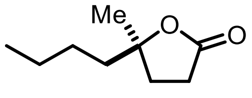

Prepared according to the general procedure in 0.2 mmol scale, using Cyclohexane as solvent and catalyst 7g (Ar = *p*-^{t}Bu-C_6H_4, R = C_6F_5), and at 10 °C for 8 d, purified by flash column chromatography using 15-25% Et2O in hexane as eluents, The desired product (volatile) was obtained as colorless oil (26 mg, 83% yield, **93.5:6.5 e.r.**);
^{1}H NMR (501 MHz, CDCl$_3$) δ 2.66 – 2.54 (m, 2H), 2.09 (ddd, *J* = 12.9, 9.5, 7.9 Hz, 1H), 1.97 (ddd, *J* = 12.9, 9.5, 6.5 Hz, 1H), 1.71 – 1.61 (m, 2H), 1.38 (s, 3H), 1.36 – 1.21 (m, 4H), 0.92 (t, *J* = 6.9 Hz, 3H);
^{13}C NMR (126 MHz, CDCl$_3$) δ 177.0, 87.1, 40.9, 33.1, 29.3, 26.1, 25.8, 23.1, 14.1;
HRMS (ESIpos) calculated for $C_9H_{16}O_2Na$ [M+Na]$^+$: 179.104249, found: 179.104150.
Enantiomeric ratio was determine by GC analysis Column: 30 m Cyclosil B, Temperature: 220/80 5/min 220 5min iso/350; Gas: 0,50 bar H_2., t_R (major) = 21.4 min., t_R (minor) = 21.7 min.
[α]$_D$^{25} = -17.82 (*c* 0.18, $CHCl_3$)

Application of the Asymmetric Hydrolactonization: Synthesis of Bioactive structures

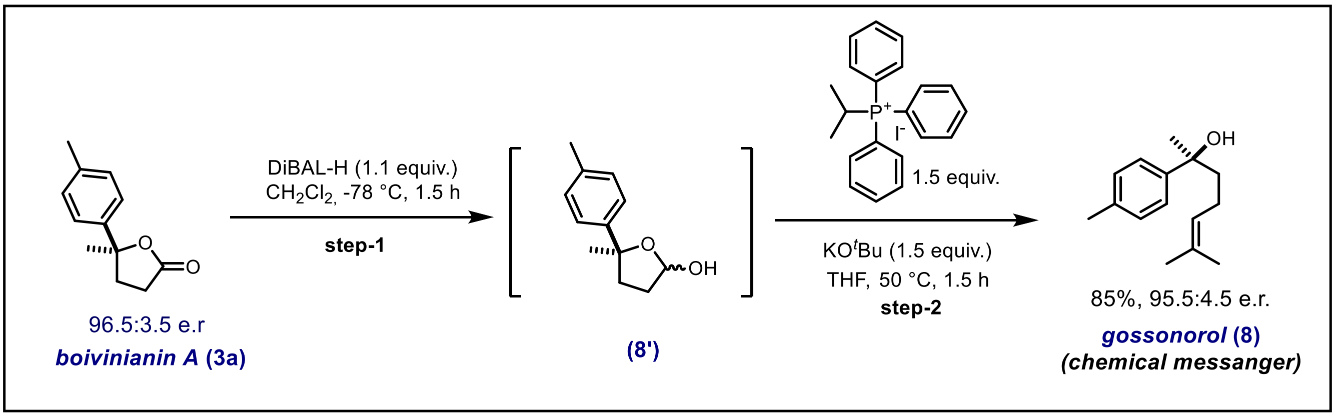

***Figure S5*: Application of the method towards natural product synthesis**

Step-1: To a stirr solution of enantiopure lactone (**3a**) (29 mg , 0.15 mmol, 1.0 equiv.) in CH_2Cl_2 (1 mL) at –78 °C, was added a solution of DIBAL-H (0.17 mL , 0.17 mmol, 1.1 equiv.) in heptane (1 M) over 5 min. Then the reaction mixture was allowed to stir 1.5 h. Afterwards the reaction mixture was treated with MeOH (0.2 mL) at –78 °C and slowly warmed up to rt. Then 5 mL of saturated aqueous solution of sodium potassium salt of tartaric acid was added and diluted with CH_2Cl_2 (5 mL) with continuous stirring for 1 h. The organic layer was separated out, washed with brine and dried over anhydrous MgSO4. The crude product (**8'**) was directly charged for the next step.

Step-2: A flame dried schlenk flask under argon was charged with isopropyl triphenyl phosphoniumiodide (98 mg , 0.23 mmol, 1.5 equiv.) in THF (1 mL). A solution of KO^{t}Bu (25 mg, 0.23 mmol, 1.5 equiv.) in THF (1 M) was added at rt drop-wise over 2 min. Then the reaction mixture was allowed to stir 10 min. Afterwards a THF solution (0.3 mL) of crude (**8'**) (29 mg , 0.15 mmol, 1.0 equiv.) was added, the reaction mixture was placed over a 50 °C oil bath, and stirring was continued for 1.5 h (TLC showed complete consumption of starting material). Then the reaction mixture was cooled down to rt and treated with H_2O (5 mL), diluted with Et_2O (5 mL). The organic layer was collected and dried over anhydrous $MgSO_4$. The crude product was purified by flash column chromatography using hexane to 5% EtOAc in hexane as eluents. The product was isolated as colorless oil (28 mg, 85%).

(*S*)-5-methyl-5-(p-tolyl)dihydrofuran-2(3H)-one (3)

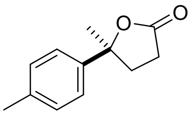

(3)

0.11 mmol, s.m. 25.7 mg, obtain 24.9 mg, 97% yield, colorless liq, e.r. 96.5:3.5.
^{1}H NMR (^{1}H NMR (501 MHz, $CDCl_3$) δ 7.31 – 7.22 (m, 2H), 7.18 (d, *J* = 8.0 Hz, 2H), 2.66 – 2.56 (m, 1H), 2.56 – 2.44 (m, 2H), 2.44 – 2.36 (m, 1H), 2.35 (s, 3H), 1.71 (s, 3H).
^{13}C NMR (126 MHz, $CDCl_3$) δ 176.6, 141.3, 137.4, 129.3, 124.1, 87.0, 36.2, 29.5, 29.0, 20.9.
HRMS (ESI) (m/z): calculated for $C_{12}H_{14}O_2$ $[M]^+$: 190.0994; found : 190.0994
The enantiomeric ratio was measured by HPLC analysis using following parameters: Daicel Chiralcel IC-3 column: *n*Hept : *i*PrOH = 95:5, flow rate 1.0 mL/ min, t_{major} = 17.3 min, t_{minor} = 20.1 min
$[\alpha]_D^{25}$ = –27.4 (*c* 0.23, $CHCl_3$)

(*S*)-6-methyl-2-(p-tolyl)hept-5-en-2-ol (8)

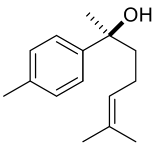

(8)

0.1 mmol, s.m. 23.5 mg, obtain 30 mg, 86% yield, colorless oil, e.r. 95.5:4.5.
^{1}H NMR (501 MHz, $CDCl_3$) δ 7.31 (d, *J* = 7.9 Hz, 2H), 7.15 (d, *J* = 7.8 Hz, 2H), 5.09 (m, 1H), 2.34 (s, 3H), 1.99–1.79 (m, 5H), 1.65 (s, 3H), 1.53 (s, 3H), 1.49 (s, 3H);
^{13}C NMR (126 MHz, $CDCl_3$) δ 145.1, 136.1, 132.3, 129.0, 124.9, 124.4, 75.0, 43.9, 30.7, 25.8, 23.1, 21.1, 17.8;
HRMS (ESI) (m/z) calculated for $C_{15}H_{22}ONa$ $[M+Na]^+$: 241.156284, found: 241.156180.
The enantiomeric ratio was determined by HPLC analysis using following parameters: Daicel Chiralpak AD-3 column: *n*Hept:*i*PrOH = 99:1, flow rate 1.0 mL/ min, t_{major} = 5.1 min, t_{minor} = 6.5 min.
$[\alpha]_D^{25}$ = –4.3 (*c* 0.19, $CHCl_3$)

Limitation of Substrate Scope:

All these substrates are prepared according to the general methods. However, these substrates turned out to be not reacting/decomposed under reaction condition.

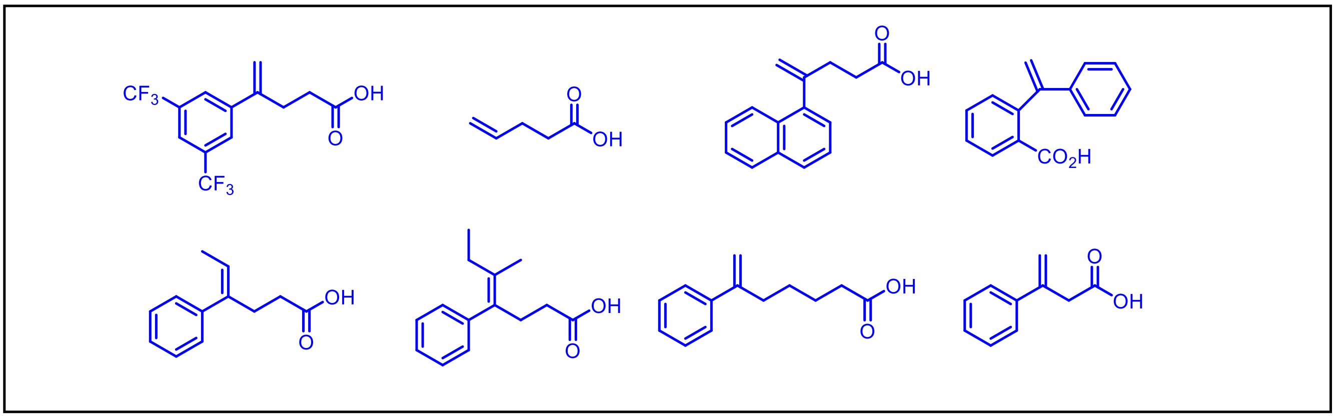

***Figure S6*:** Substrates that are found to be incompatible for asymmetric hydrolactonization

6. Determination of the Absolute Configuration

The absolute configuration was assigned based on known values documented in the literature.[9-11]

compound	structure	measured $[\alpha]_D^{25}$	literature
**6a**	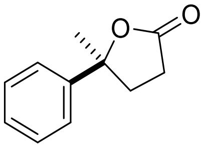	$[\alpha]_D^{25}$ = -22.69 (*c* 0.29, $CHCl_3$), *S*, 96.5:3.5 e.r.	$[\alpha]_D^{25}$ = –32.1 (*c* 1.0, $CHCl_3$), *S*, 92:8 e.r.
**3**	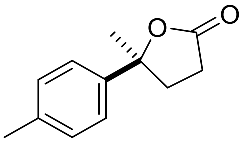	–27.4 (*c* 0.23, $CHCl_3$) *S*, 96.5:3.5 e.r.	$[\alpha]_D^{25}$ = –37.7 (*c* 1.1, $CHCl_3$), *S*, 89.5:10.5 e.r.
**6b**	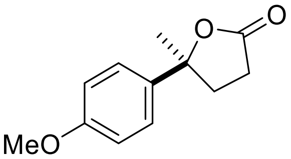	–58.5 (*c* 0.24, $CHCl_3$) *S*, 97.5:2.5 e.r.	$[\alpha]_D^{25}$ = -35.3 (*c* 1.0, $CHCl_3$) *S*, 91:9 e.r.
**6n**	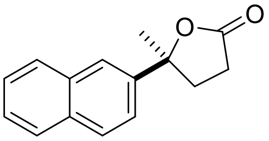	–19.4 (*c* 0.12, $CHCl_3$) *S*, 96:4 e.r.	$[\alpha]_D^{25}$ = –28.6.1 (*c* 0.8, $CHCl_3$), *S*, 93.5:6.5 e.r.
**6p**	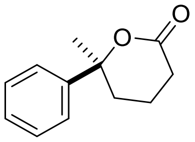	-33.9 (*c* 0.45, $CHCl_3$) *S*, 94.5:5.5 e.r.	$[\alpha]_D^{20}$ = –39.0 (*c* 0.67, $CHCl_3$) *S,* 97.5:2.5 e.r.

7. Kinetic Studies to Investigate the Reaction Mechanism

General NMR data processing:

NMR data was imported into MNOVA 14.2.3 with the reaction monitoring plugin and processed therein. After phase and baseline correction NMR concentration profiles were generated by the olefinic signal at 5.26 ppm (1 × H) and the CH_3 group (3 × H) of the product **3a** at 1.70 ppm. All concentration points were referenced to the first acquired ^{1}H NMR spectrum. The actual catalyst concentration in solution was also extracted from the NMR data and used for the analysis. (Signal at 0.88 ppm, 2 × *t*Bu, 18 × H).
Exemplary stacked ^{1}H NMR spectra acquired for a reaction under standard conditions (0.2M **4u**; 4.3 mol% **7g**) is shown in the following figure:

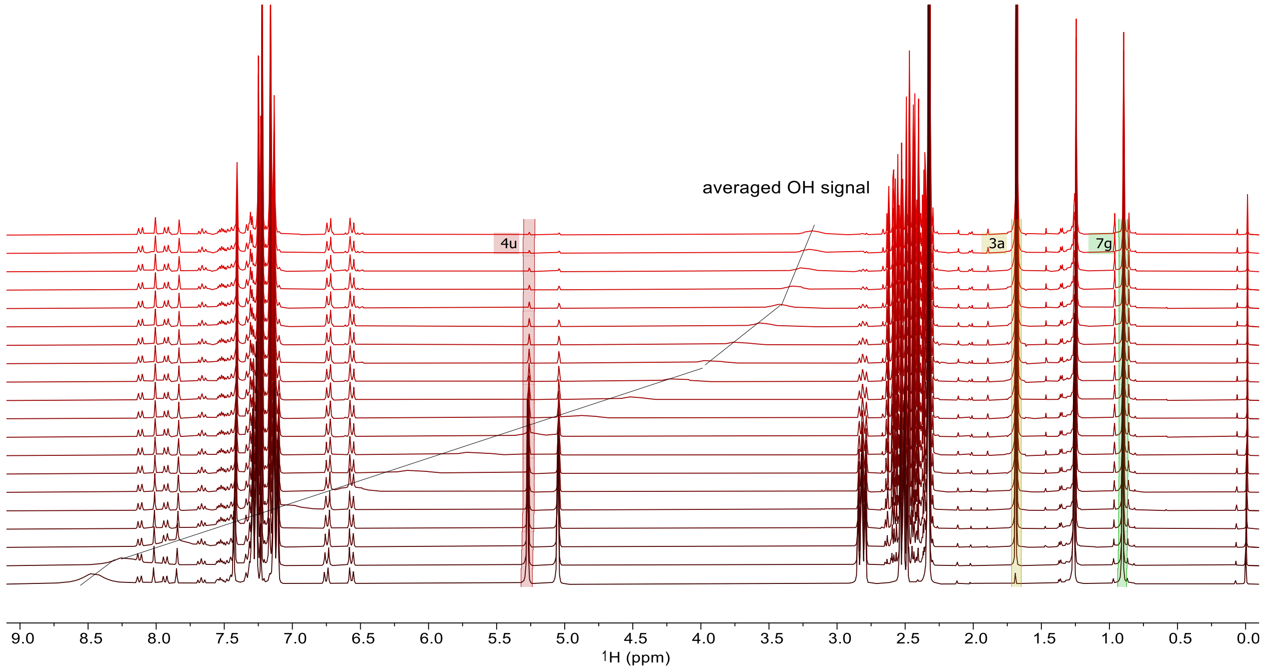

Figure S7: Stacked ^{1}H NMR spectra at different time points during the hydrolactionisation of **4a** (0.2M in $CDCl_3$) in the presence of catalyst **7g** (4.3 mol%).

Besides the decay of the substrate **4u** and the formation of **6a** a broad signal attributed to the average OH species (catalyst proton, carboxylic acid proton, residual water) is shifting from 8.5 to 3.2 ppm over the course of the reaction and appears therefore in some of the concentration profiles as a 'dip'.

(A) Reaction order determination

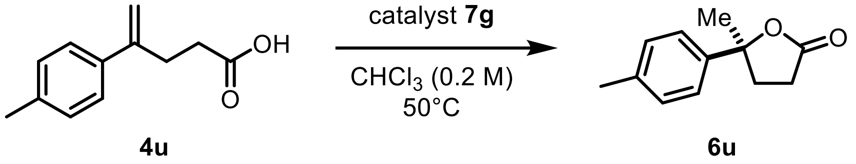

The reaction orders of alkenoic acid **4u** and catalyst **7g** were determined by variable time normalization analysis (VTNA).[13] Three different concentrations of each component of interest were used to determine the respective order. Overall 5 experiments were performed for this study.

(1) Catalyst Order

Sample preparation

To a J. Young NMR tube was added alkenoic acid (**4u**, 0.1 mmol, 19.0 mg) and anhydrous $CDCl_3$ (0.5 mL) along with catalyst **7g** (0.4 mol%, 2.5 mol%, 4.3 mol%). The NMR tube was then transferred quickly to NMR spectrometer preheated to 323 K (50°C) and after fast shimming, ^{1}H NMR spectra were acquired every 10–15 min until the starting material was > 80% consumed.

The following figure shows concentration profiles of **4u** (0.2M in $CDCl_3$) in the presence of different amount of catalyst **7g** with times scales normalized to different catalyst orders (0th,1st,2nd):

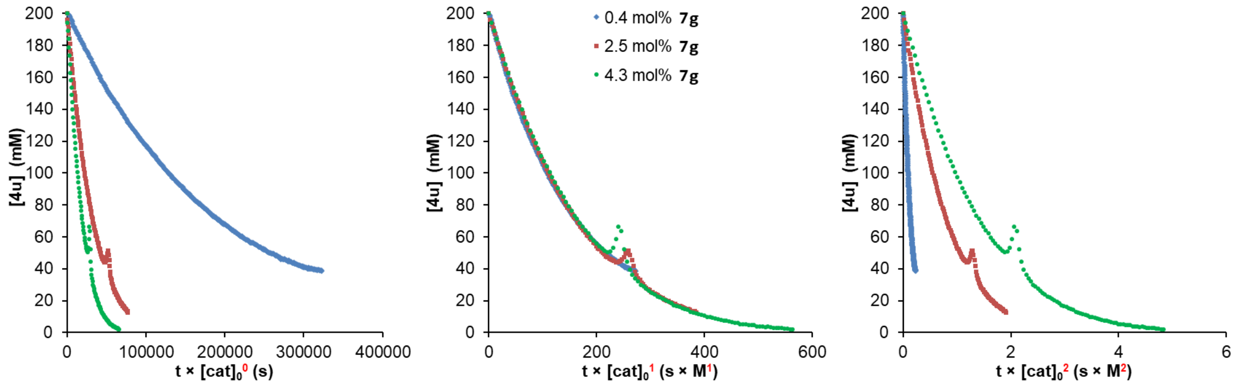

Figure S8: NMR concentration profiles with time scales normalized to different catalyst orders (left: zeroth order, middle: first order, right: second order)

The best overlap is found when a first order dependence in catalyst concentration is assumed. This suggests that only one catalyst molecule is involved in the rate limiting step of the reaction.

(2) Substrate order

The reaction order of substrate (**4u**) by variable time normalization analysis (VTNA).[13] Three different concentrations were used to determine the substrate reaction.

Sample preparation

To a J. Young NMR tube was added alkenoic acids (**4u**, 0.1 mmol/0.072mmol/0.2mmol) and anhydrous $CDCl_3$ (0.5 mL) along with catalyst **7g** (7.1 mg, 17.1 µmol). The NMR tube was then transferred quickly to NMR spectrometer preheated to 323 K (50°C) and after fast shimming, ^{1}H NMR spectra were acquired every 10 min until the starting material was > 95% consumed.

The following concentration profiles were obtains for using different initial concentrations of **4a** (145mM, 200mM, 400 mM):

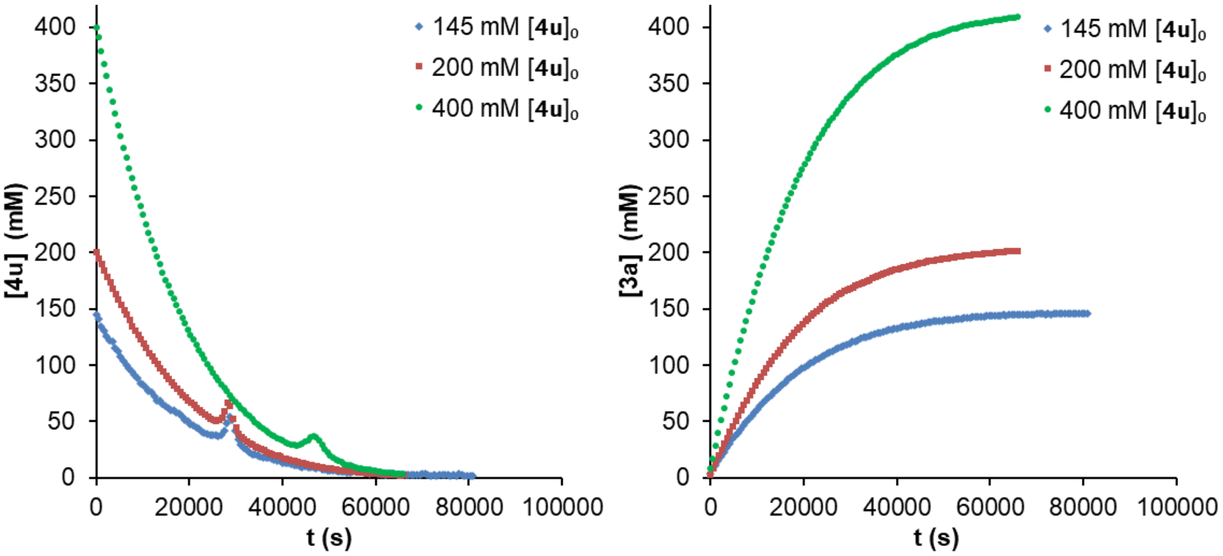

Figure S9: Concentration profiles obtained for different initial concentration of **4a** in presence of 8.6 mM catalyst **7g**.

Concentration profiles normalized to different reaction order of substrate **4a** can be found the following figure:

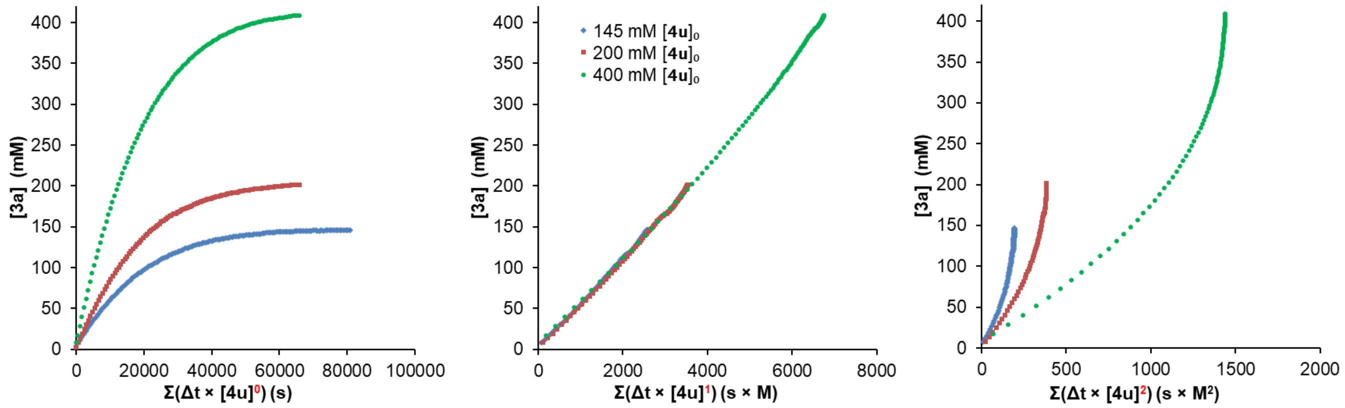

Figure S10: NMR concentration profiles with time scales normalized to different reactant orders (left: zeroth order, middle: first order, right: second order)

The best overlap is obtained when the time scales are normalized to a first order dependence of reactant concentration.

Remark: The concentrations of **4u** relevant for the normalization were used as obtained. Although the 'OH-dip' was not removed during the normalization process, it did not seem to influence the VTNA outcome significantly.

After the reaction order determination of the individual reaction components, all acquired datasets were plotted in the same graph and after normalisation a good overlap of all reaction profiles was observed:

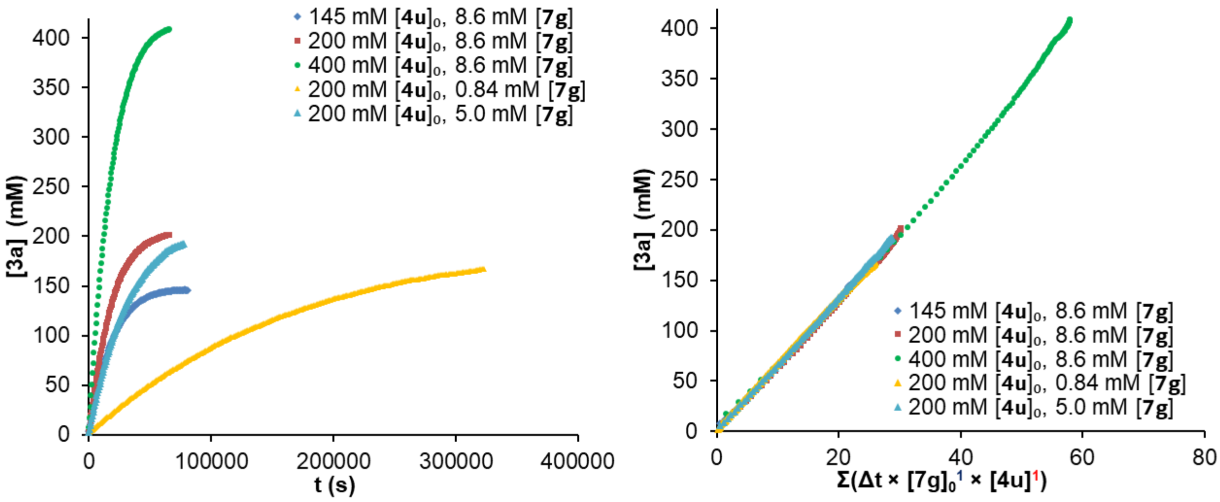

Figure S11: NMR concentration profiles obtained for the reaction order determination before (left) and after (right) VTNA.

(B) Hammett analysis

The Hammett analysis was performed by comparing the relative rates of product formation of *p*-X substituted aryl alkenoic acids **4** to that of the *p*-H substituted aryl alkenoic acid **4a** (Fig. **S12**).[12]

Sample preparation

To a J. Young NMR tube was added 0.1 mmol alkenoic acid (**4**) and anhydrous $CDCl_3$ (0.5 mL) along with ~3-10 mol% (see Table S1) of catalyst **7g**. The NMR tube was then transferred quickly to NMR spectrometer preheated to 323K (50°C) and after fast shimming, ^{1}H NMR spectra were acquired. The *k* values were obtained from an exponential fit in Microsoft Excel by plotting [4] against t x [**7g**]$_0$. All concentration profiles are shown in the following figure:

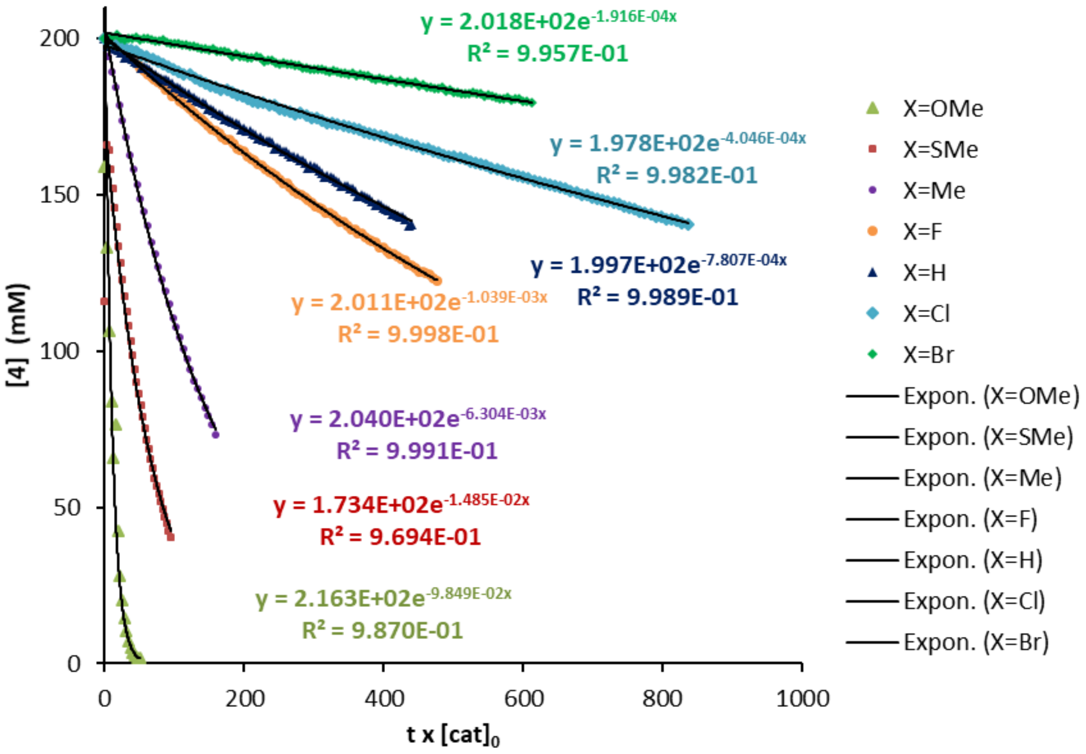

Figure 12: Concentration profiles of **4** used for Hammett studies. Inserts show the fitting results used for generating the Hammett plot.

Remarks: **4c** (X=SMe) used for the measurements contained an impurity (~ 20mol%; likely oxidized species X = S(=O)Me), which did not convert significantly during the measurement time.
All extracted *k* values and relevant parameters used for the analysis are given in the following table:

Table S3: Overview of all parameters used for the Hammett Analysis

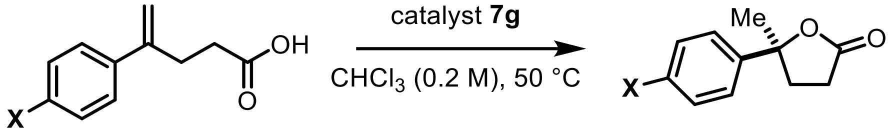

para substitutent	σ_{para}	σ^{+}_{para}	$[7g]_0$ (M)	k ($M^{-1} \times s^{-1}$)	log (k_X/k_H)
**X = H**	0.000	0.000	6.66E-03	7.81E-04	0.00
**X = Me**	-0.170	-0.311	8.56E-03	6.30E-03	0.91
**X = Cl**	0.227	0.114	8.44E-03	4.05E-04	-0.29
**X = F**	0.062	-0.073	5.68E-03	1.05E-03	0.13
**X = Br**	0.232	0.135	6.18E-03	1.92E-04	-0.61
**X = OMe**	-0.268	-0.78	1.04E-02	9.85E-02	2.10
**X = SMe**	0	-0.6	1.09E-02	1.48E-02	1.22

A plot of log (k_X/k_H) vs. the corresponding σ_p and σ_p+ resulted in the following Hammett plots:

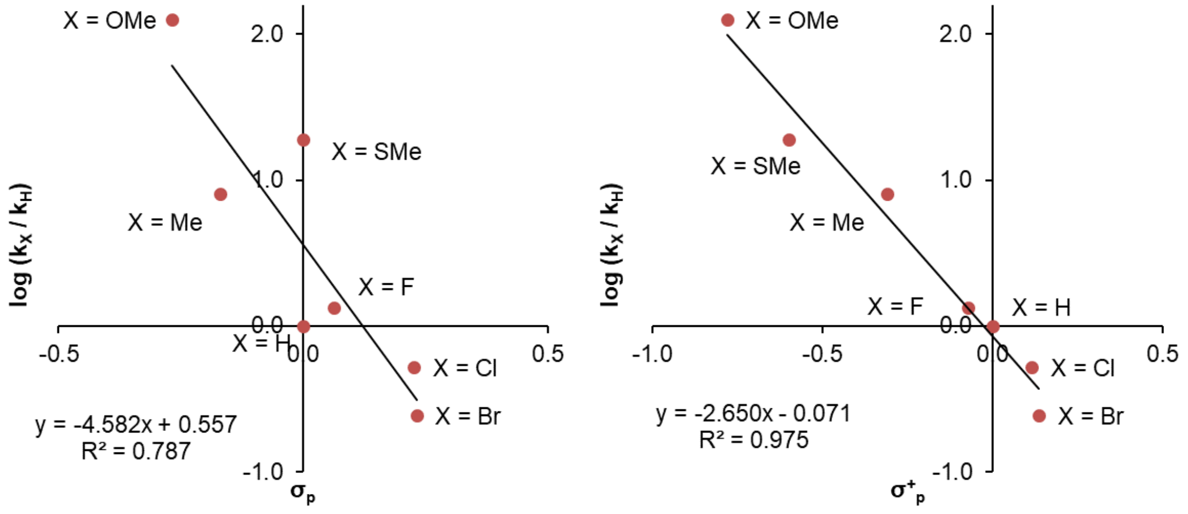

Figure S13: *Hammett plots obtained after plotting log(k_x/k_H) against σ_p (left) and $\sigma^+{}_p$ (right).*

The substrates have the good linear correlation with a negative slope ($\rho^+ = -2.65 \pm 0.19$; R^2=0.975) when plotting log(k_X/k_H) against $\sigma_p{}^+$. This is consistent with a charge accumulation in the transition state of the cationic nature of the transition step and a significant charge accumulation. Notably, the correlation σ_p against is significantly weaker.

(C)Determination of thermodynamic parameters

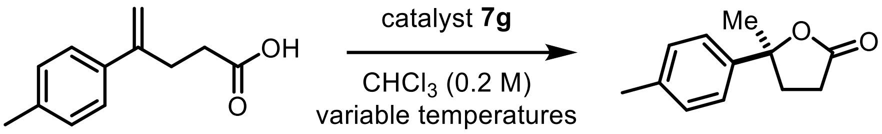

Sample preparation

To a J. Young NMR tube was added alkenoic acid (**4u**, 0.1 mmol, 19.0 mg) and anhydrous $CDCl_3$ (0.5 mL) along with catalyst **7g** (2.5 – 5 mol%; see Table S2). The NMR tube was then transferred quickly to NMR spectrometer preheated to the corresponding temperature (30 °C, 40 °C, 50 °C or 60 °C) and after fast shimming, ^{1}H NMR spectra were acquired every 10–15 min until the reaction reached at least >40% conversion.

The following graph shows the concentration profiles of **4u** against t x [**7g**]$_0$.

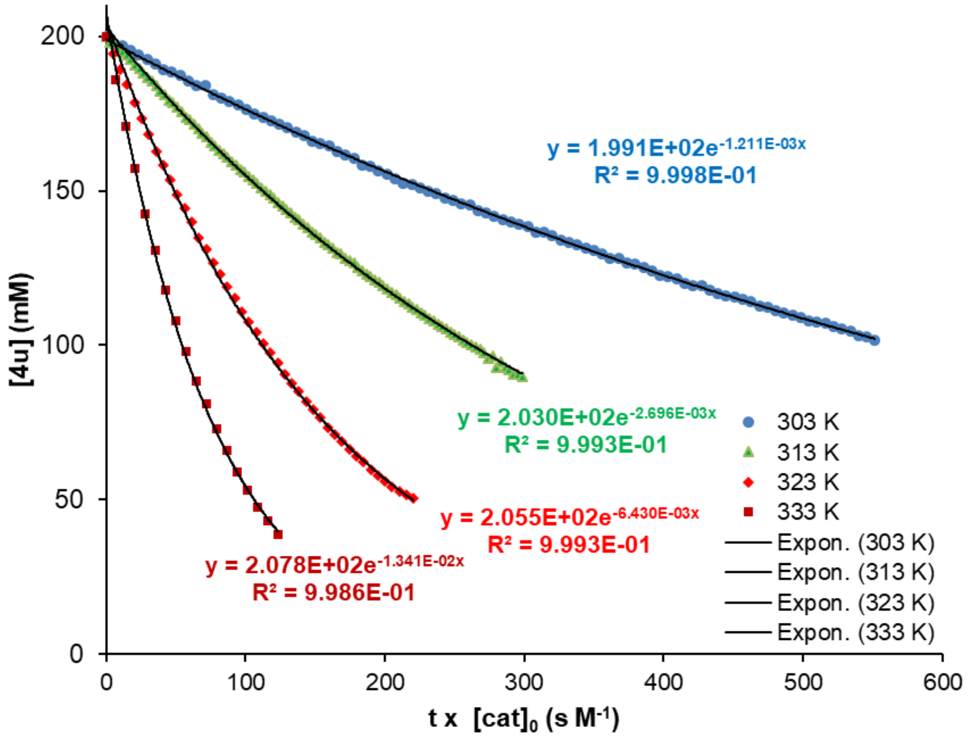

Figure 14: Concentration profiles of 4u at different temperatures (303K, 313K, 323K, 333K) plotted against t x [**7g**]$_0$. The insets show the results of the exponential fitting in Microsoft Excel at the different temperatures used for the Eyring analysis.

The *k* values for the Eyring Analysis were extracted from the concentration plots by exponential fitting in Microsoft Excel. An overview of all extracted values is shown in **Table S5**. The physical constants used for calculation are given in **Table S4**.

When plotting ln $((k \times h/(k_b \times T)))$ against 1/T one can extract the thermodynamical parameters $-\Delta H^‡/R$ and $\Delta S^‡/R$ as slope and intercept respectively after linear regression in Microsoft Excel (**Table S6**).

The values $\Delta H^‡$, $\Delta S^‡$ *and* $\Delta G^‡_T$ $(=\Delta H^‡ - T\Delta S^‡)$ can be derived from these parameters (**Table S7**). The Eyring plot including the linear regression result and the values of the activation enthalpy $\Delta H^‡$, activation entropy $\Delta S^‡$ and Gibb energy of activation $\Delta G^‡_{313K}$ are shown in the following figure:

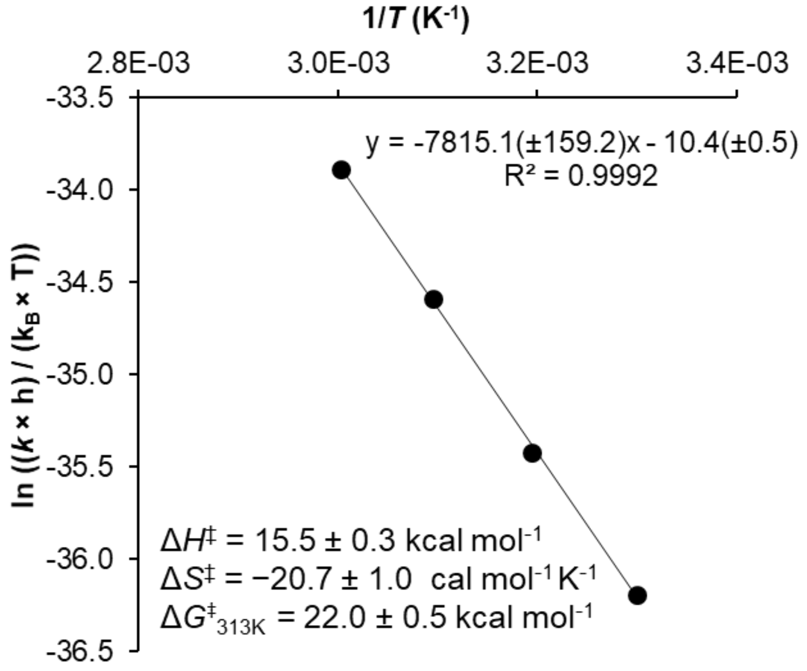

Figure 15: Eyring plot obtained for the hydrolactonisation of **4u** in presence of catalyst **7g**. The insert shows the linear fitting result and the derived thermodynamical parameters $\Delta H^{\ddagger}$,$\Delta S^{\ddagger}$ and $\Delta G^{\ddagger}_{313K}$.

Table S4: Physical constants used for the Eyring analysis.

	**value**	**unit**
R	8.3144598	J K^{-1} mol^{-1}
h	6.62607E-34	J s
k_B	1.38064852E-23	J K^{-1}

Table S5: Overview of all parameters used for the Hammett Analysis

**T(K)**	**1/T (K^{-1})**	**[7g] (M)**	***k*** **(s^{-1})**	**($k \times h/(k_b \times T)$)**	**ln (($k \times h/(k_b \times T)$))**
303.0	0.0033	9.89E-03	1.21E-03	1.92E-16	-36.19
313.0	0.003195	5.03E-03	2.70E-03	4.13E-16	-35.42
323.0	0.003096	8.56E-03	6.43E-03	9.55E-16	-34.58
333.0	0.003003	8.10E-03	1.33E-02	1.92E-15	-33.89

Table S6: Linear regression results from plotting ln (($k \times h/(k_b \times T)$)) against 1/T

	**Slope** $-\Delta H^{\ddagger}/R$	**Intercept** $\Delta S^{\ddagger}/R$	
**value**	-7815.094	-10.415	**value**
**error**	159.2929186	0.50185	**error**
**R^2**	0.9992	0.03531	*stdev(A)*

Table S7: Derived parameters after linear regression.

$\Delta H^{\ddagger}$	65.0	± 1.3	kJ mol^{-1}
**$\Delta H^{\ddagger}$**	**15.5**	**± 0.3**	**kcal mol^{-1}**
$\Delta S^{\ddagger}$	-86.6	± 4.2	J mol^{-1} K^{-1}
**$\Delta S^{\ddagger}$**	**-20.7**	**± 1.0**	**cal $mol^{-1}K^{-1}$**
$\Delta G^{\ddagger}_{313\ K}$	92.1	± 1.9	kJ mol^{-1}
**$\Delta G^{\ddagger}_{313\ K}$**	**22.0**	**± 0.5**	**kcal mol^{-1}**

(D) Comments on the Catalyst Resting State and Stability

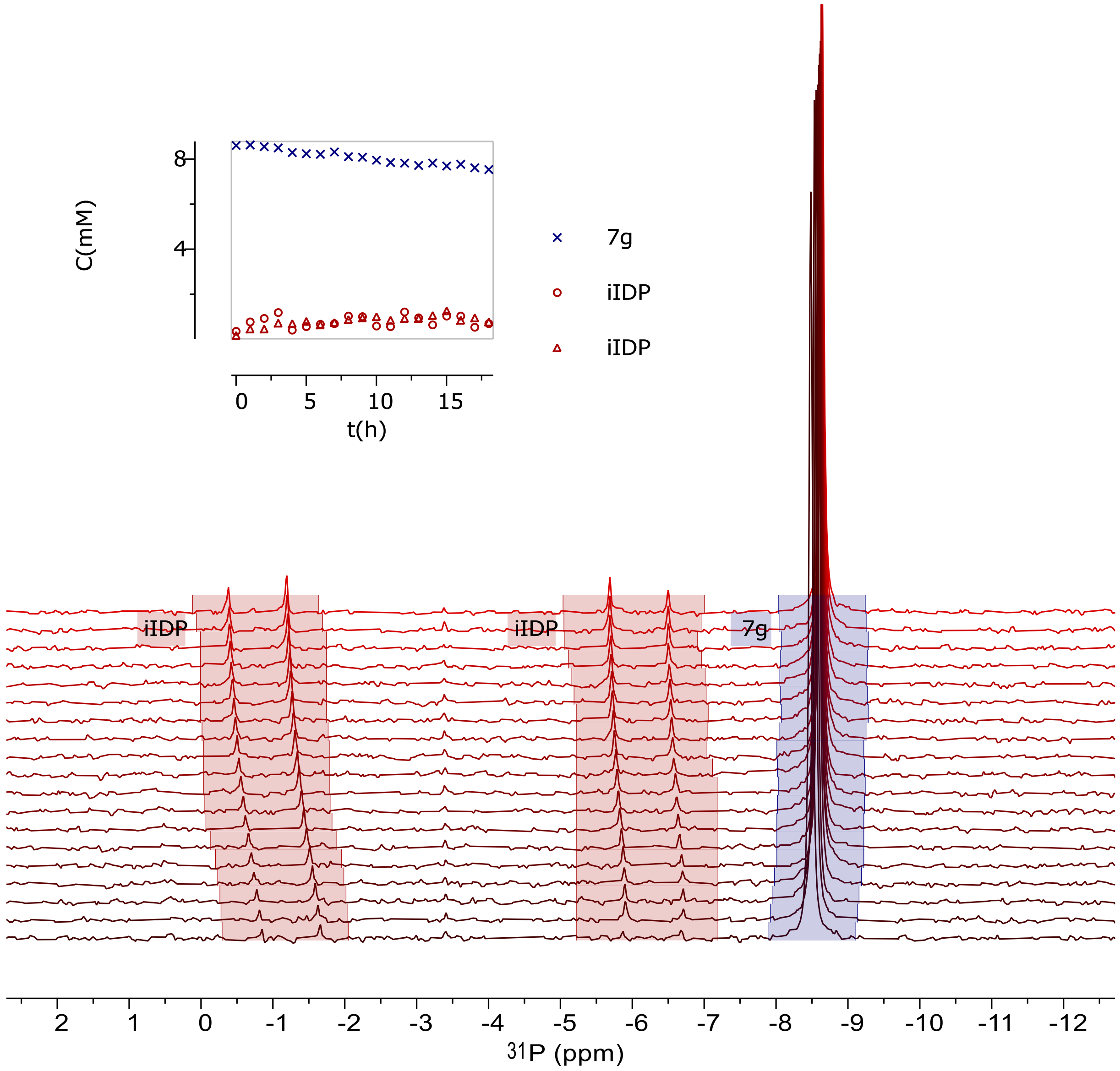

***Figure S16*:** Determination of Catalyst resting state of the enantioselective hydrolactonization for substrate **4u** catalyzed by **7g**

The reaction shown in **Figure S7** was also followed by ^{31}P NMR every 60 min for approximately 18 h (**Figure S16**). The main catalyst peak remains peak remains intact and shifts slightly during the reaction. This is in line with the uncoordinated catalyst being the resting state during the reaction. A significant amount of a transient catalyst adduct with the substrate was not observed. However, a slight catalyst decomposition (~13 mol% over the monitoring period) was observed at elevated temperature over time. Two ^{31}P doublets grow with intensity which can be assigned to the iIDP variant of **7g** through hydrolysis by residual water in the sample. This finding seems not to have influenced the VTNA analysis significantly as most of the substrate was already converted.

8. Computational Studies

8.1 Methods

All calculations presented in this paper were carried out with a development version of the ORCA suite of programs based on version 4.2.[14] Molecular geometries were optimized in the gas-phase using the PBE functional[15] in conjunction with the D3 version of Grimme's dispersion correction with Becke-Johnson damping function,[16] using the resolution of identity approximation. The def2-SVP basis set was used for all atoms with matching auxiliary basis. Analytic frequency calculations were performed to verify the nature of all stationary points (minima and transition states) and to calculate free energies and enthalpies at 313.15 K by using the rigid-rotor harmonic oscillator (RRHO) approximation.[17] An exhaustive manual conformational search has been performed on possible catalyst substrate orientations. Analytic frequency calculations were performed to verify the nature of all stationary points (minima and transition states) and to calculate free energies and enthalpies at 313.15 K by using the rigid-rotor harmonic oscillator (RRHO) approximation. Transition state structures were verified by the presence of a single imaginary vibrational frequency. Buried volume analysis following the protocol by Cavallo and coworkers.[18]

Single-point energies are calculated at B3LYP-D3/def2-TZVP level of theory and the C-PCM-(Chloroform) solvent model has been used as implemented in ORCA. Distortion-Interaction[19] and fragmentation analysis[20] has been performed to qualitatively understand the reason behind stereoinduction. Molecular structures were generated using CYLview program.[21]

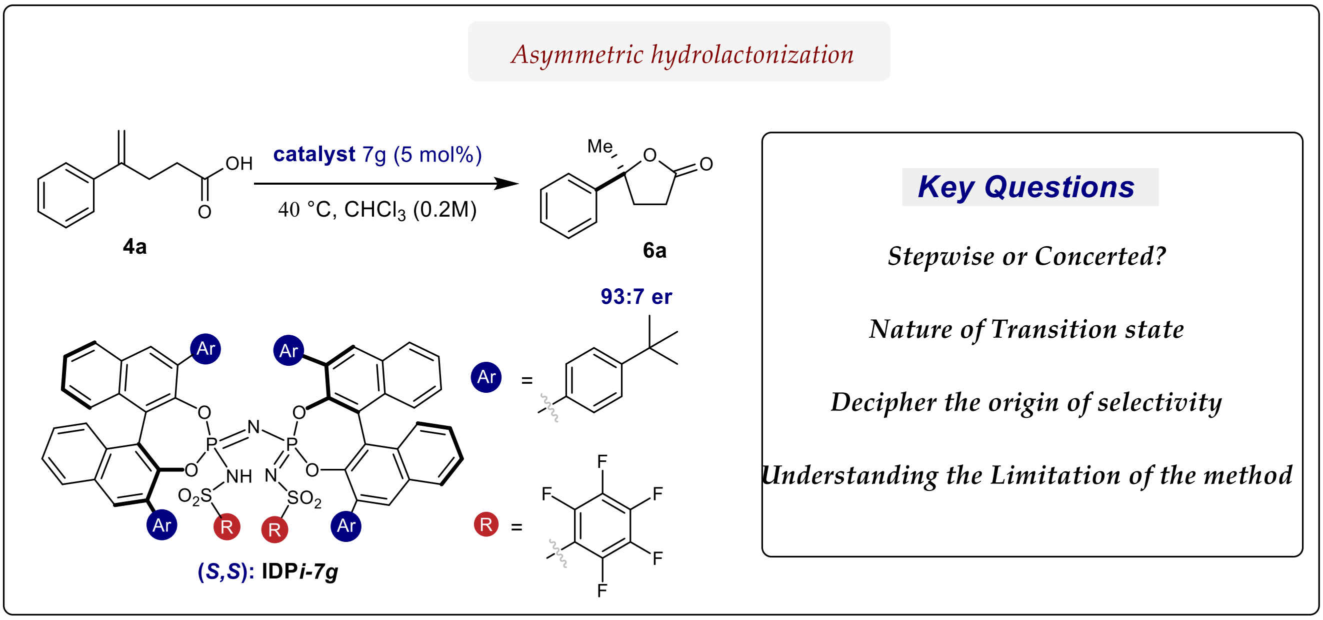

Figure S17: Summary of the overall computational study undertaken for this work

8.2 Results and Discussions

(a) Reaction pathway: Stepwise or concerted?

Previous studies on electrophilic lactonizations have suggested two distinct mechanistic pathways: (i) stepwise protonation and subsequent stereoselective cyclization, or (ii) concerted stereoselective cyclization. To understand which pathway is operative (Figure **S18**) in this case, we undertook extensive DFT calculations.

Computed stepwise pathway (Fig **S19-** solid back line) has an energetic span[22] of 33.4 kcal·mol^{-1}, where pre-reaction complex for the protonation is the TOF determining intermediate (TDI) while cyclization step (**TS-C1)** the TOF determining TS (TDTS). Noticeably, within the stepwise pathway, olefin protonation is rate-limiting (25 kcal·mol^{-1}) and cyclization (**TS2)** is selectivity determining. Similar to previous reports, protonation occurs preferentially via the nitrogen atom (rather than oxygen: e.g TS-B2). In contrast, the concerted pathway (dotted line) is significantly more stable, and our computed energy barriers (22.5 kcal·mol^{-1}) comport with the experimental observations (22.0 ± 0.5 kcal·mol^{-1}). The optimized concerted TS (TS-A1) is highly asynchronous, where alkene protonation C–H (1.18 Å) precedes ring closure C–O (2.33 Å) and therefore resulting in a net accumulation of charge (NBO charge) at the quaternary carbon center C_{ortho} (+0.26*e*), which accounts for the Hammett analysis. Therefore, based on our physical organic and computational study, we concluded that asymmetric hydrolactonization follow a highly asynchronous concerted mechanism. Moreover, it is noteworthy that the H-bond established as the early molecular recognition between the carboxylic end of the substrate and the sulfone moiety of the catalyst (OH···O interaction) remains conserved along the entire reaction pathway (Fig **S19**). Such attribute is reminiscent of enzymatic catalysis in *dethiobiotin synthetase* and *chorismate mutase,* where similar carboxyl mediated substrate recognition was achieved.[23] All these structural constraints lead to an appreciable reduction of degrees of freedom of the reaction partners, in concert with the high activation entropy $\Delta S^{\ddagger}$ (-20.7 ± 1 kcal mol^{-1}), as determined based on Eyring equation.

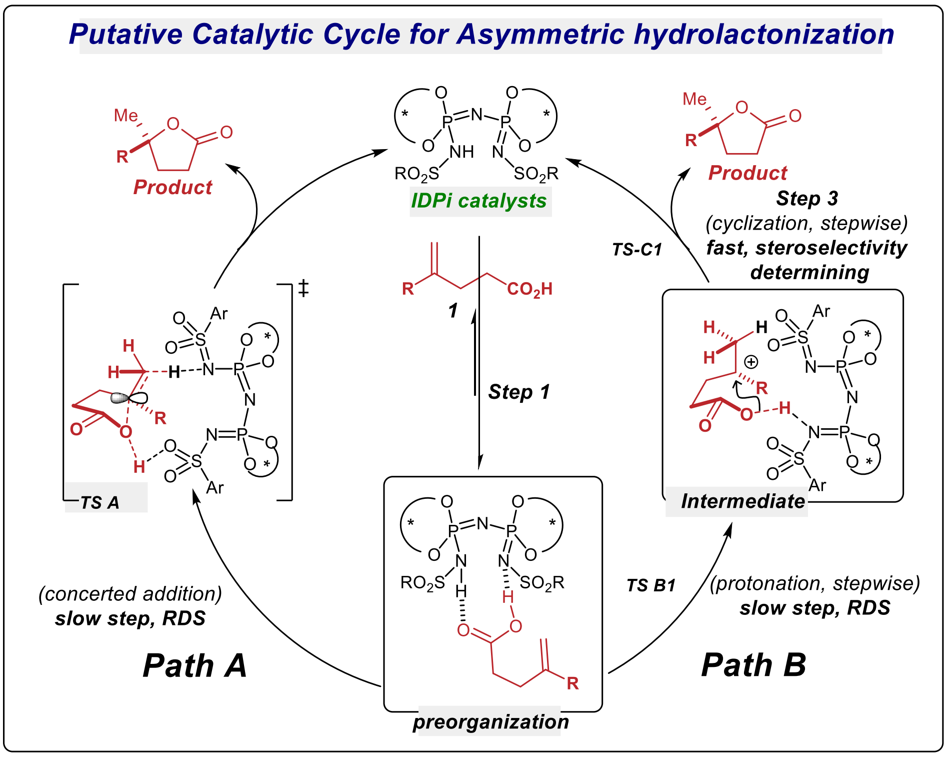

Figure S18: Putative mechanistic possibilities of asymmetric hydrolactonization

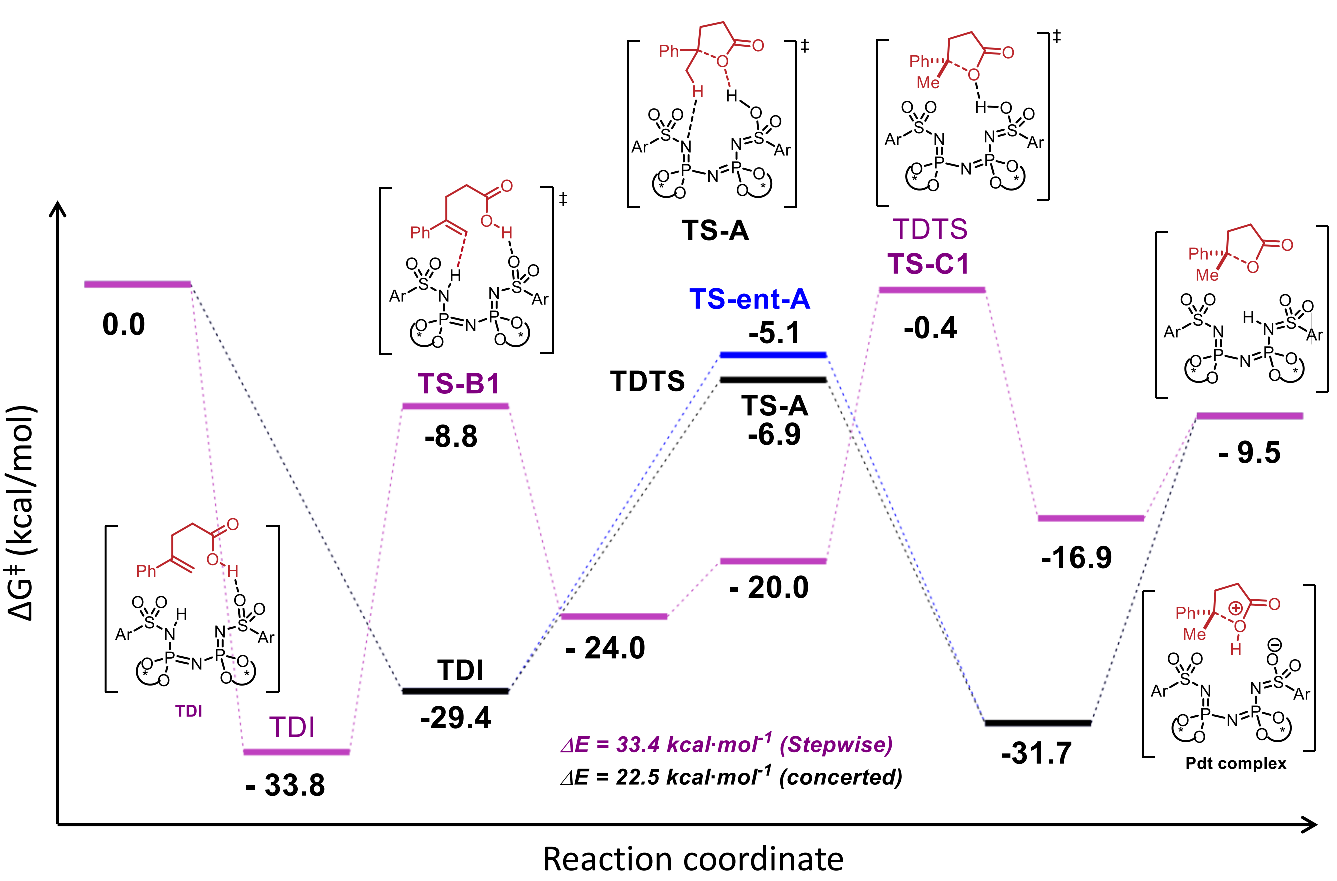

Figure S19. Key TS structures and free energy differences computed at the *B3LYP-D3/def2-TZVP + CPCM(chloroform)//PBE-D3/def2-SVP level of theory.*

Table S8. Computed energy diagram of the asymmetric hydrolactonization

TS No	PBE RRHO corrections	*B3LYP-D3/def2-TZVP* single point ($CHCl_3$ solv)	*Imaginary Freq.*	□□G(TS) Final	Relative Free Energy (kcal/mol)
Rct	0.05147017	-576.7835072	NA	-576.7321	0.0
Pdt	0.04867459	-576.795698	NA	-576.7472	-9.47
Catalyst	0.22529841	-6792.93293	NA	-6792.7076	NA
Total				-7369.4397	0.0
Pdt+cat				-7369.4548	
Concerted Pathway (dotted line)					
Rct_complex	-0.2483756	-7369.734795	NA	-7369.4864	-29.30
TS A1(major)	0.27157676	-7369.72205813	-69.4	-7369.4500	-6.91
TS_enan_A1 (minor)	0.26706166	-7369.7141072	-134.25	-7369.4471	-5.12
TS_closed_ A	0.2724427	-7369.718378	-128.08	-7369.4459	
Pdt_complex	0.24899	-7369.739204		-7369.4902	-31.68
Stepwise Pathway (solid line)					
Step 1: Protonation					
Rct_complex_B1	0.250528	-7369.744082	NA	-7639.4935	-33.76
TS_B1	0.27231343	-7369.72606	- 1214.94	-7369.4537	-8.78
Pdt_complex_B1	0.24824	-7369.72625	NA	-7369.47801	**-24.03**
	Step 2: Cyclization				
Rct_complex_C1	0.25112023	-7369.722836	NA	-7369.4716	-20.01
TS_C1	0.27233818	-7369.712625	-59.25	-7369.44042	-0.4
Pdt_complex_C1	0.24939617	-7369.71618600	NA	-7369.4668	-16.93
Protonation via oxygen atom of the IDPi Catalyst (stepwise)					
TS_B2	.27312540	-7369.708732	-894.2	-7369.4356	11.35

(b) Substrate binding within the confined catalyst cavity:

Despite the presence of pronounced π stacking between the per-fluorinated sulfonamide substituents and the BINOL backbone of the catalyst (both in crystal structure[24] and DFT optimized ground state structure), during the reaction, the catalyst sacrifices one of the stacking to accommodate the substrate within its confined cavity ($\Delta\Delta G^{\ddagger}$ =2.52 kcal mol^{-1}). Using Houk-Bickelhaupt Distortion-Interaction(Table S9) analysis, we concluded, that the associated enthalpic loss in the opening of one of the catalyst arm has been more than compensated by the reduced angular distortion caused by greater available space for substrate orientation. Notably, a similar effect recently observed during our Asymmetric Spirocyclizing Diels–Alder Reaction. [25]

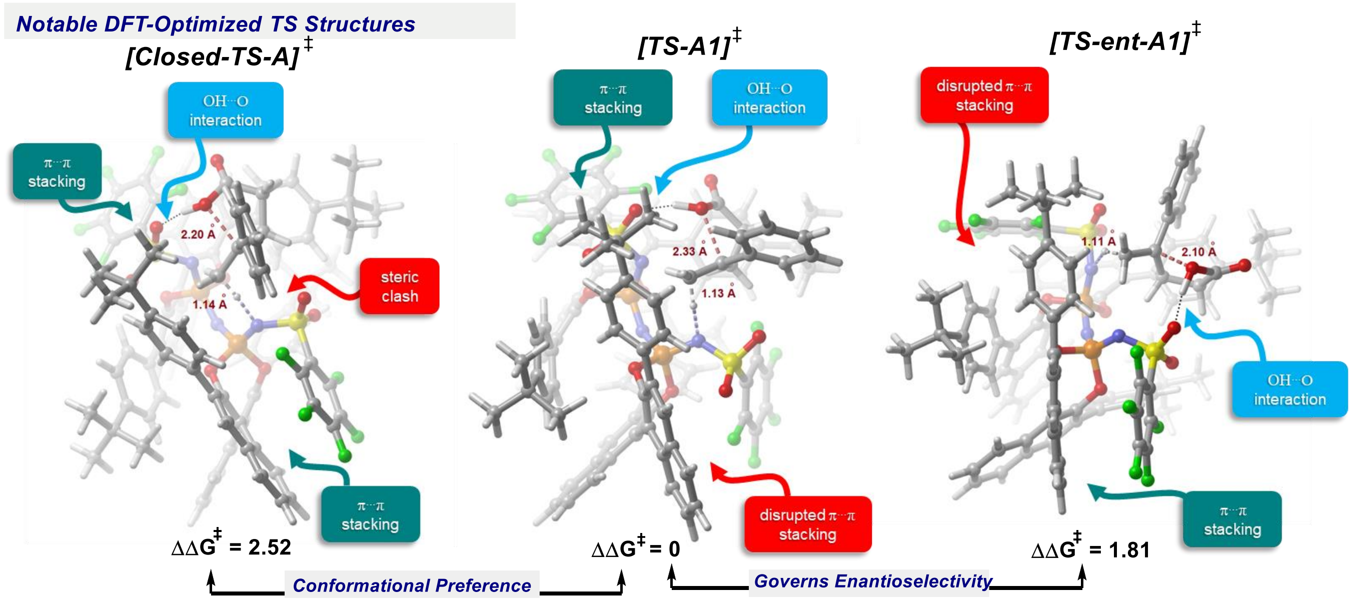

Figure S20. Summary of the overall Computational study undertaken for this work stereo determining TS structures at the *B3LYP-D3/def2-TZVP + CPCM(chloroform)//PBE-D3/def2-SVP level of theory.*

Table S9. Distortion-Interaction Analysis to understand the conformational preference (TS leading to major isomer)

TS No	*B3LYP-D3/def2-TZVP* single point (gas phase)	Relative Energy (kcal/mol) Δ E
TS-A1	-7369.687918	0.62
TS-closed-A	-7369.6869908	
Substrates Only		
Subst_TS-A1	-577.09587	13.52
Subst_TS-closed-A	-577.077949	
Catalyst Only		
Cat_TS-closed-A1	-6792.40964	-5.20
Cat_TS-A2	-6792.417910	
Total Distortion		8.32

(c) Understanding the origin of stereoselectivity

With the elementary steps of the catalytic cycle, and conformational preference deduced from theory, we next turned towards understanding the stereoselectivity of this transformation. DFT computed er values B3LYP-D3/def2-TZVP+C-PCM-(Chloroform)//PBE-D3/def2-SVP+C-PCM-(Chloroform) 94.5:5.5 ($\Delta\Delta G^{\ddagger}$ = 1.8 kcal·mol^{-1}) are in excellent agreement with the experimental observations 93:7 ($\Delta\Delta G^{\ddagger}$ = 1.7 kcal·mol^{-1}). To interrogate the nature of interactions between catalyst and substrate, we employed the Distortion-Interaction analysis (protonated substrate and catalyst counteranion), which indicates substrate distortion is the predominantly factor for selectivity (Total net distortion 5.94 kcal·mol^{-1}). Notably, such high geometrical distortion from the substrate can be understood considering the reduced catalyst cavity size (mean distance of two sulfonyl groups within the chiral counteranion from 6.85 Å to 6.53 Å) in minor isomer, thereby resulting an angular strain developed within the substrate during adopting the required conformation for cyclization in the TS leading to the minor isomer. This is also results in less distortion observed for the catalyst counteranion for TS_{Minor} which preserves most of its stacking from the ground state. All these insights garnered from the Distortion Interaction analysis, also consistent with the reaction selectivity observed in the presence of similar catalysts. Based on this DFT model, one should anticipate, such destabilization of the minor isomer would have been more pronounced for larger perflourinated sulfonamide substituted (*e.g.* Catalyst **7h**), hence steering the process more selective. In contrast, a relatively smaller sulfonamide substituent (*e.g.* Catalyst **7b**) would not provide sufficient rigidity and therefore results in poor selectivity. Indeed, the experimentally observed selectivity for catalyst 7h and 7b [er 96:4 and 67:33 respectively] is in qualitative agreement with this trend.

Table S10. Distortion-Interaction Analysis to understand the Origin of enantioselectivity

TS No	*B3LYP-D3/def2-TZVP* single point (gas phase)	Relative Energy (kcal/mol) Δ E
TS-A1	-7369.687918	1.94
TS-enant-A1	-7369.684800	
Substrates Only		
Subst_TS-A1	-577.09587	9.03
Subs_TS-enan-A1	-577.08140	
Catalyst Only		
Cat_TS-A1	-6792.40964	-3.08
Cat_TS-enan-A1	-6792.41452	
Total Distortion		5.94

Taken together, the computational analysis has ascertained the preferred reaction pathway, deconstructed the conformational preferences, and rationalized the stereoselectivity.

(d) Understanding the limitation of Substrate Scope

Finally, based on our computed TS structures, we wanted to rationalize the limitation of our method for *tri*- and *tetra*-substituted olefins. As mentioned earlier, these reactions occur *via* a highly organized TS assembly, where the proton transfer from the catalyst occurs while conserving the OH···O interaction between the substrate carboxylic acid group and the sulfone moiety of the catalyst. Notably, a successful olefin protonation requires the olefin to be accommodated deep inside the catalyst cavity without creating steric congestion. Given the average distance between two Nitrogen atoms ~3.5 Å, a more substituted olefin will likely create a steric destabilization to fit into the cavity, explaining its incompatibility towards asymmetric hydrofunctionalization.

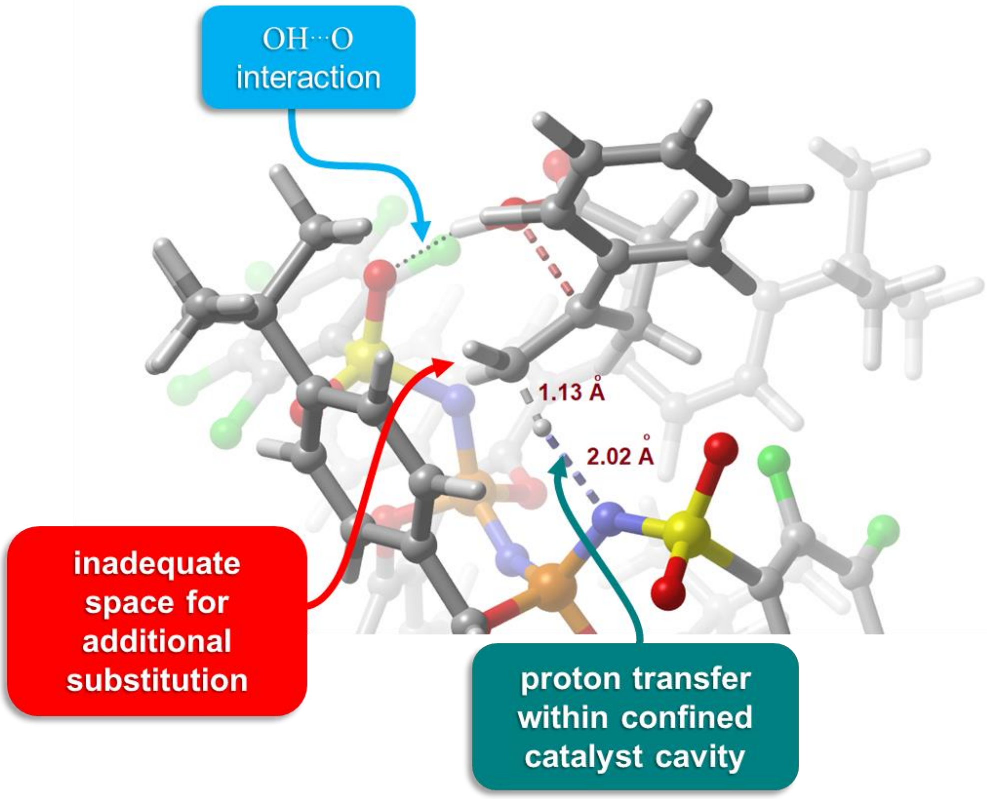

Figure S21. Theoretical rationale behind the observed incompatibility with *tri*- and *tetra*-substituted olefins (zoomed in structure for TS leading to major isomer has shown here).

9. References

Substrate Preperation reference:

1. Whitehead, D. C.; Yousefi, R.; Jaganathan, A.; Borhan, B. An Organocatalytic Asymmetric Chlorolactonization. *Journal of the American Chemical Society* **2010**, *132*, 3298-3300.
2. Nakatsuji, H.; Sawamura, Y.; Sakakura, A.; Ishihara, K. Cooperative Activation with Chiral Nucleophilic Catalysts and N-Haloimides: Enantioselective Iodolactonization of 4-Arylmethyl-4-pentenoic Acids. *Angewandte Chemie International Edition* **2014**, *53*, 6974-6977.
3. Zhou, L.; Tan, C. K.; Jiang, X.; Chen, F.; Yeung, Y.-Y. Asymmetric Bromolactonization Using Amino-thiocarbamate Catalyst. *Journal of the American Chemical Society* **2010**, *132*, 15474-15476.
4. Jiang, X.; Tan, C. K.; Zhou, L.; Yeung, Y.-Y. Enantioselective Bromolactonization Using an S-Alkyl Thiocarbamate Catalyst. *Angewandte Chemie International Edition* **2012**, *51*, 7771-7775.

Catalyst Synthesis:

5. Kaib, P. S. J.; Schreyer, L.; Lee, S.; Properzi, R.; List, B., Extremely Active Organocatalysts Enable a Highly Enantioselective Addition of Allyltrimethylsilane to Aldehydes. *Angewandte Chemie International Edition* **2016,** *55* (42), 13200-13203.
6. Tsuji, N.; Kennemur, J. L.; Buyck, T.; Lee, S.; Prévost, S.; Kaib, P. S. J.; Bykov, D.; Farès, C.; List, B. Activation of Olefins via Asymmetric Brønsted Acid Catalysis. *Science* **2018**, *359* (6383), 1501–1505.
7. Ghosh, S.; Erchinger, J. E.; Maji, R.; List, B. Catalytic Asymmetric Spirocyclizing Diels–Alder Reactions of Enones: Stereoselective Total and Formal Syntheses of α-Chamigrene, β-Chamigrene, Laurencenone C, Colletoic Acid, and Omphalic Acid. *J. Am. Chem. Soc.* **2022**, *144* (15), 6703–6708.
8. Zou, L.-M.; Huang, X.-Y.; Zheng, C.; Cheng, Y.-Z.; You, S.-L. Chiral Brønsted Acid-Catalyzed Intramolecular Asymmetric Allylic Alkylation of Indoles with Primary Alcohols. *Organic Letters* **2022**, *24*, 3544-3548.

Determination of Absolute configuration:

9. Wang, B.; Shen, Y.-M.; Shi, Y. Enantioselective Synthesis of γ-Aryl-γ-butyrolactones by Sequential Asymmetric Epoxidation, Ring Expansion, and Baeyer–Villiger Oxidation. *The Journal of Organic Chemistry* **2006**, *71*, 9519-9521.
10. Nishikawa, Y.; Hamamoto, Y.; Satoh, R.; Akada, N.; Kajita, S.; Nomoto, M.; Miyata, M.; Nakamura, M.; Matsubara, C.; Hara, O. Enantioselective Bromolactonization of Trisubstituted Olefinic Acids Catalyzed by Chiral Pyridyl Phosphoramides. *Chemistry – A European Journal* **2018**, *24*, 18880-18885.
11. Klake, R. K.; Gargaro, S. L.; Gentry, S. L.; Elele, S. O.; Sieber, J. D. Development of a Strategy for Linear-Selective Cu-Catalyzed Reductive Coupling of Ketones and Allenes for the Synthesis of Chiral γ-Hydroxyaldehyde Equivalents. *Organic Letters* **2019**, *21*, 7992-7998.

Physical Organic Study:

12. Zhang, P Zhou, H.; Bae, H. Y.; Leutzsch, M.; Kennemur, J. L.; Bécart, D.; List, B., The Silicon–Hydrogen Exchange Reaction: A Catalytic σ-Bond Metathesis Approach to the Enantioselective Synthesis of Enol Silanes. *J. Am. Chem. Soc.* **2020**, 142, 13695–13700;
13. Burés, J., Variable Time Normalization Analysis: General Graphical Elucidation of Reaction Orders from Concentration Profiles. *Angewandte Chemie International Edition* **2016,** *55* (52), 16084-16087

Computational:

14. Neese, F. Wiley Interdiscip. Rev.: Comput. Mol. Sci. 2011, 2, 73–78.
15. Zhang, Y.; Yang, W. Comment on "Generalized gradient approximation made simple". *Phys. Rev. Lett.* **1998**, *80*, 890–890.
16. (a) Grimme, S.; Antony, J.; Ehrlich, S.; Krieg, H. A consistent and accurate *ab initio* parametrization of density functional dispersion correction (DFT-D) for the 94 elements H-Pu. *J. Chem. Phys.* **2010**, *132*, 154104. (b) Grimme, S.; Ehrlich, S.; Goerigk, L. Effect of the damping function in dispersion corrected density functional theory. *J. Comput. Chem.* **2011**, *32*, 1456–1465.
17. Weigend, F.; Ahlrichs, R. Balanced basis sets of split valence, triple zeta valence and quadruple

zeta valence quality for H to Rn: Design and assessment of accuracy. *Phys. Chem. Chem. Phys.* **2005**, *7*, 3297–3305.

18. Falivene, L.; Cao, Z.; Petta, A.; Serra, L.; Poater, A.; Oliva, R.; Scarano, V.; Cavallo, L. Towards the Online Computer-Aided Design of Catalytic Pockets. *Nat. Chem.* **2019**, *11* (10), 872–879.
19. Bickelhaupt, F. M.; Houk, K. N. Analyzing Reaction Rates with the Distortion/Interaction-Activation Strain Model. *Angew. Chem. Int. Ed.* **2017**, *56* (34), 10070–10086.
20. Maji, R.; Champagne, P. A.; Houk, K. N.; Wheeler, S. E., Activation Mode and Origin of Selectivity in Chiral Phosphoric Acid-Catalyzed Oxacycle Formation by Intramolecular Oxetane Desymmetrizations. *ACS Catalysis* **2017,** *7* (10), 7332-7339.
21. Legault, C. Y.: CYLview, 1.0b; Université de Sherbrooke, **2009** (http://www.cylview.org).
22. Kozuch, S.; Shaik, S. How to Conceptualize Catalytic Cycles? The Energetic Span Model. *Acc. Chem. Res.* **2011**, *44* (2), 101–110.
23. Huang, W.; Jia, J.; Gibson, K. J.; Taylor, W. S.; Rendina, A. R.; Schneider, G.; Lindqvist, Y., Mechanism of an ATP-Dependent Carboxylase, Dethiobiotin Synthetase, Based on Crystallographic Studies of Complexes with Substrates and a Reaction Intermediate. *Biochemistry* **1995,** *34* (35), 10985-10995.
24. Zhang, P.; Tsuji, N.; Ouyang, J.; List, B. Strong and Confined Acids Catalyze Asymmetric Intramolecular Hydroarylations of Unactivated Olefins with Indoles. *J. Am. Chem. Soc.* **2021**, 143 (2), 675–680.
25. Ghosh, S.; Erchinger, J. E.; Maji, R.; List, B. Catalytic Asymmetric Spirocyclizing Diels–Alder Reactions of Enones: Stereoselective Total and Formal Syntheses of α-Chamigrene, β-Chamigrene, Laurencenone C, Colletoic Acid, and Omphalic Acid. *J. Am. Chem. Soc.* **2022**, *144* (15), 6703–6708.

10. NMR Spectra of All New Catalyst, Starting Materials and Products

Catalyst Charecterization:

^{1}H NMR spectra (600 MHz, $CDCl_3$)

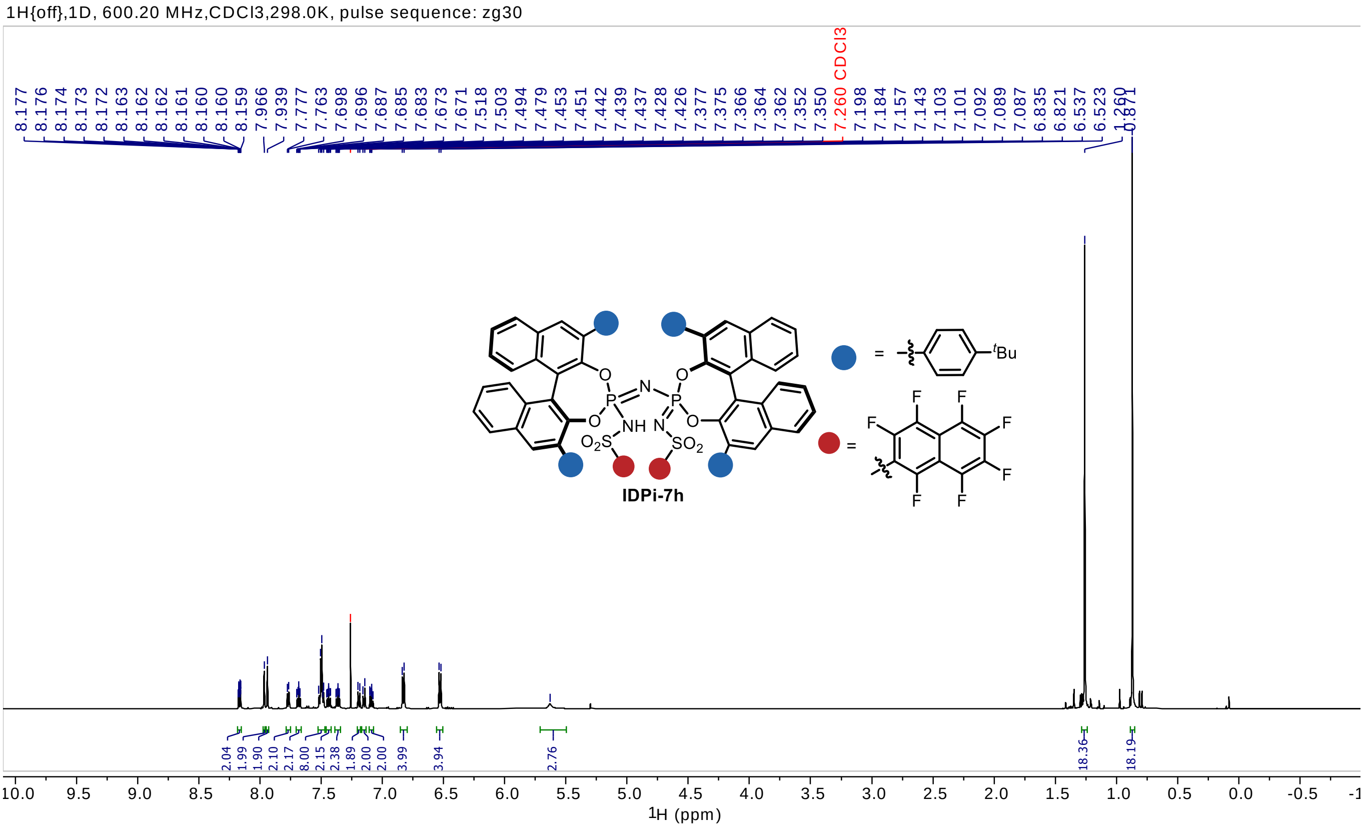

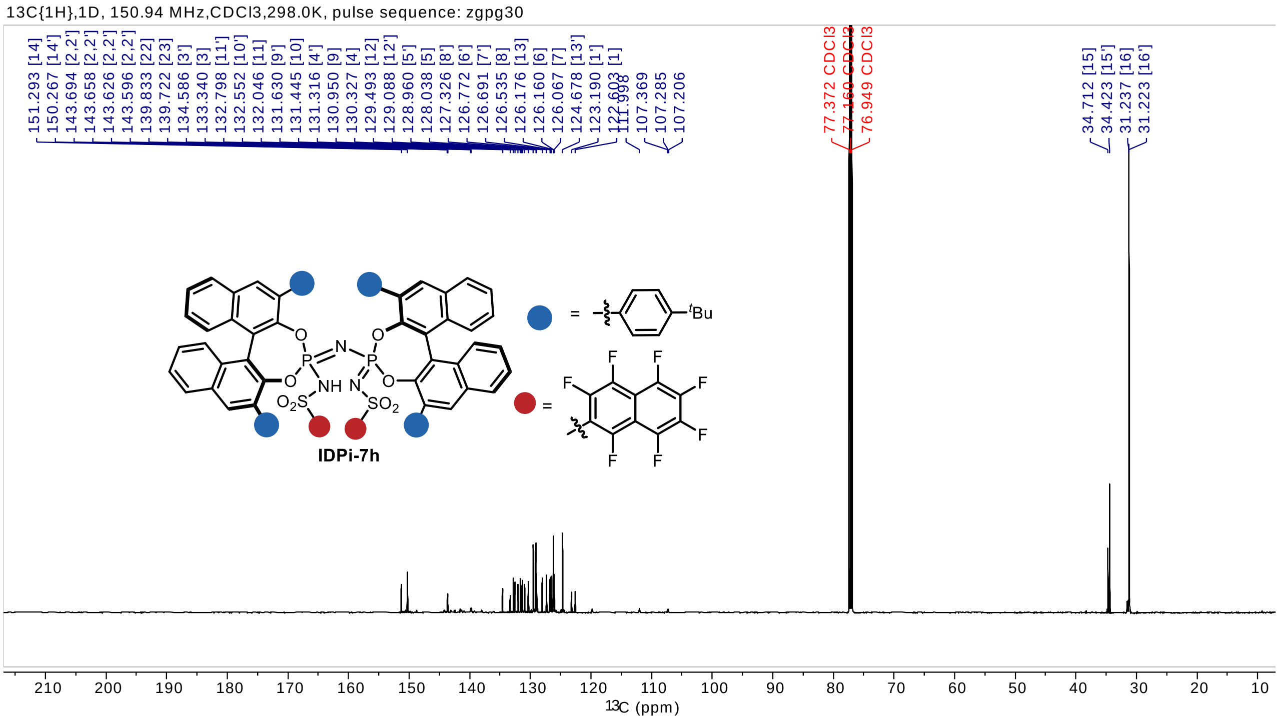

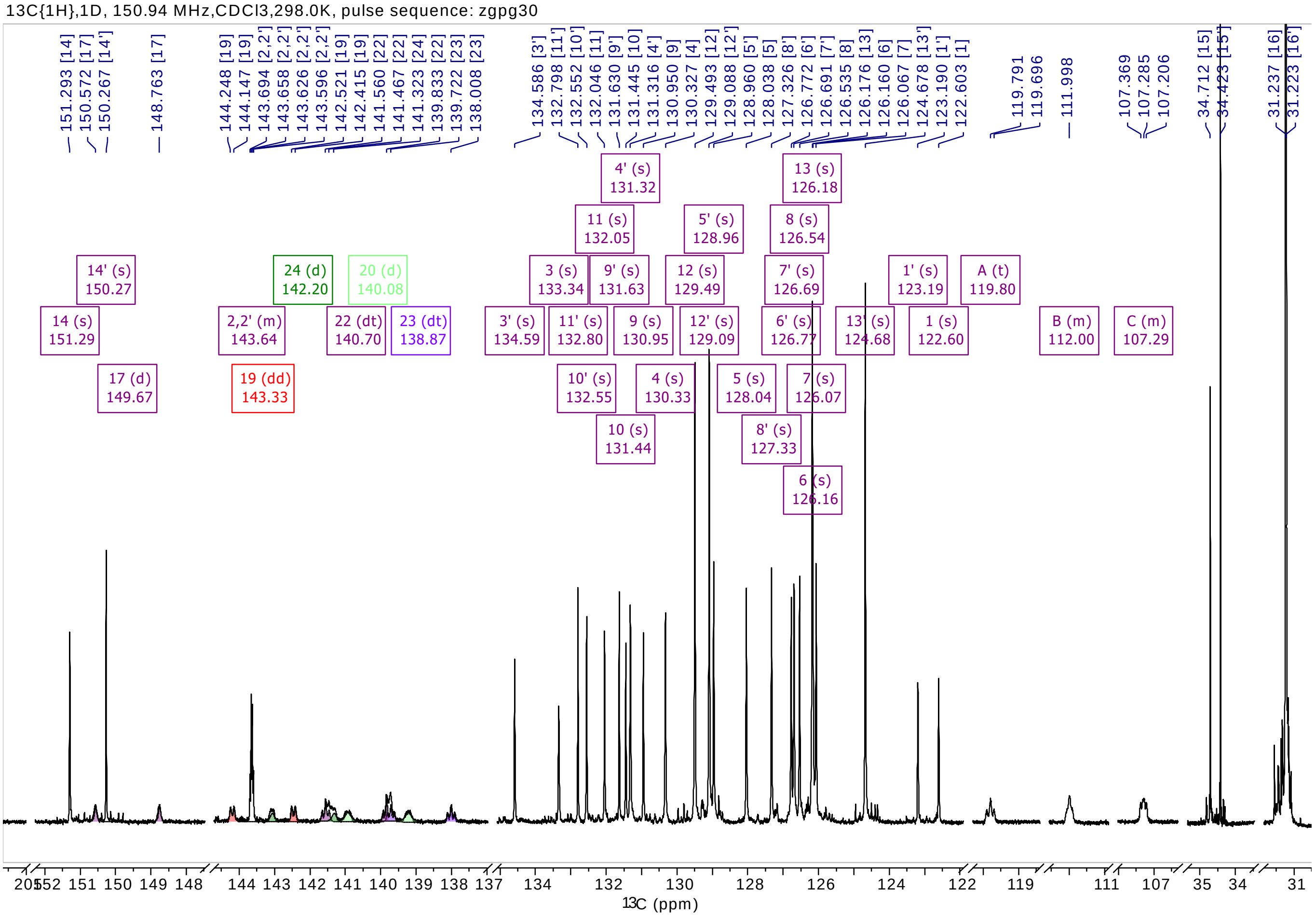
13C{1H},1D, 150.94 MHz,CDCl3,298.0K, pulse sequence: zgpg30
13C (ppm)

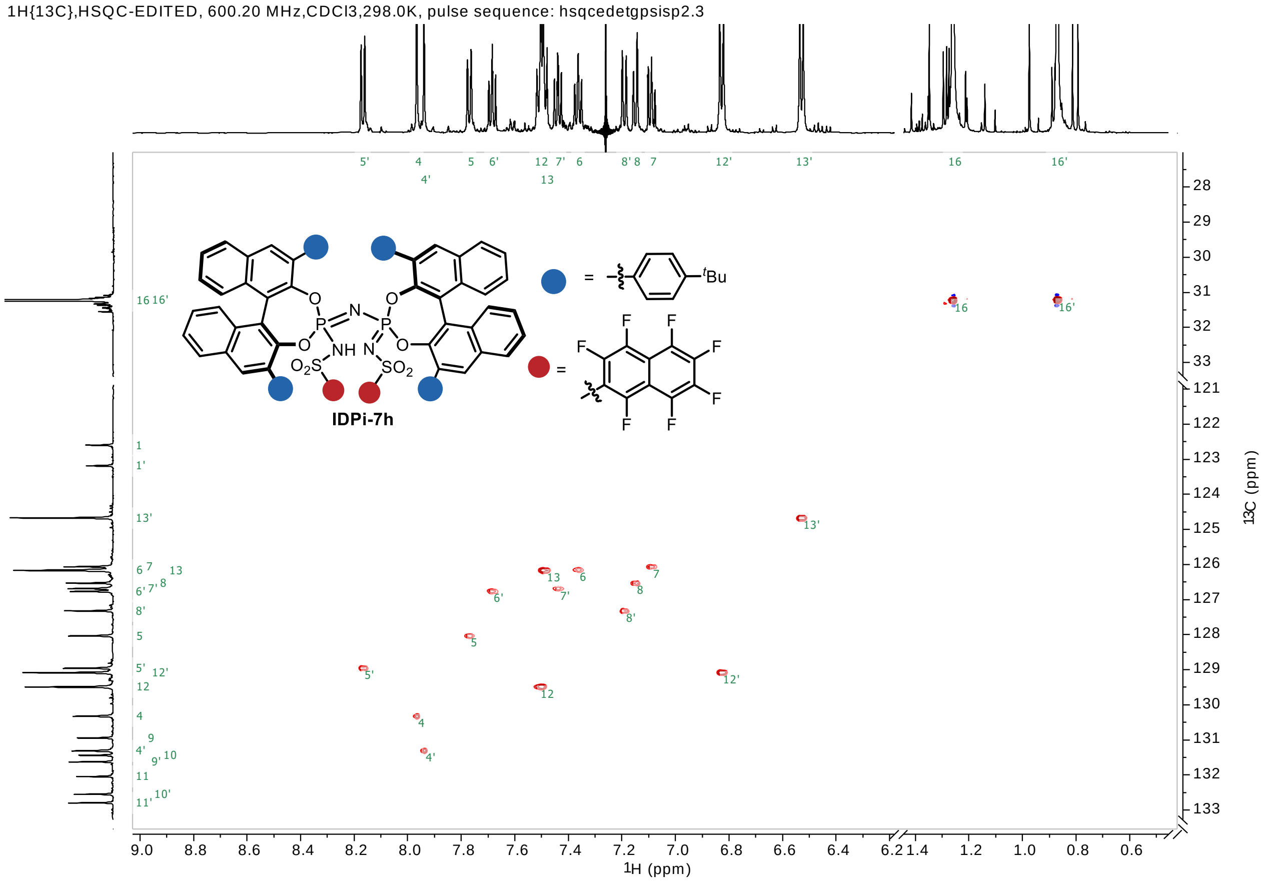
1H{13C},HSQC-EDITED, 600.20 MHz,CDCl3,298.0K, pulse sequence: hsqcedetgpsisp2.3
IDPi-7h
1H (ppm)
13C (ppm)

1H{off},ROESY, 600.20 MHz,CDCl3,298.0K, pulse sequence: roesyadjsph

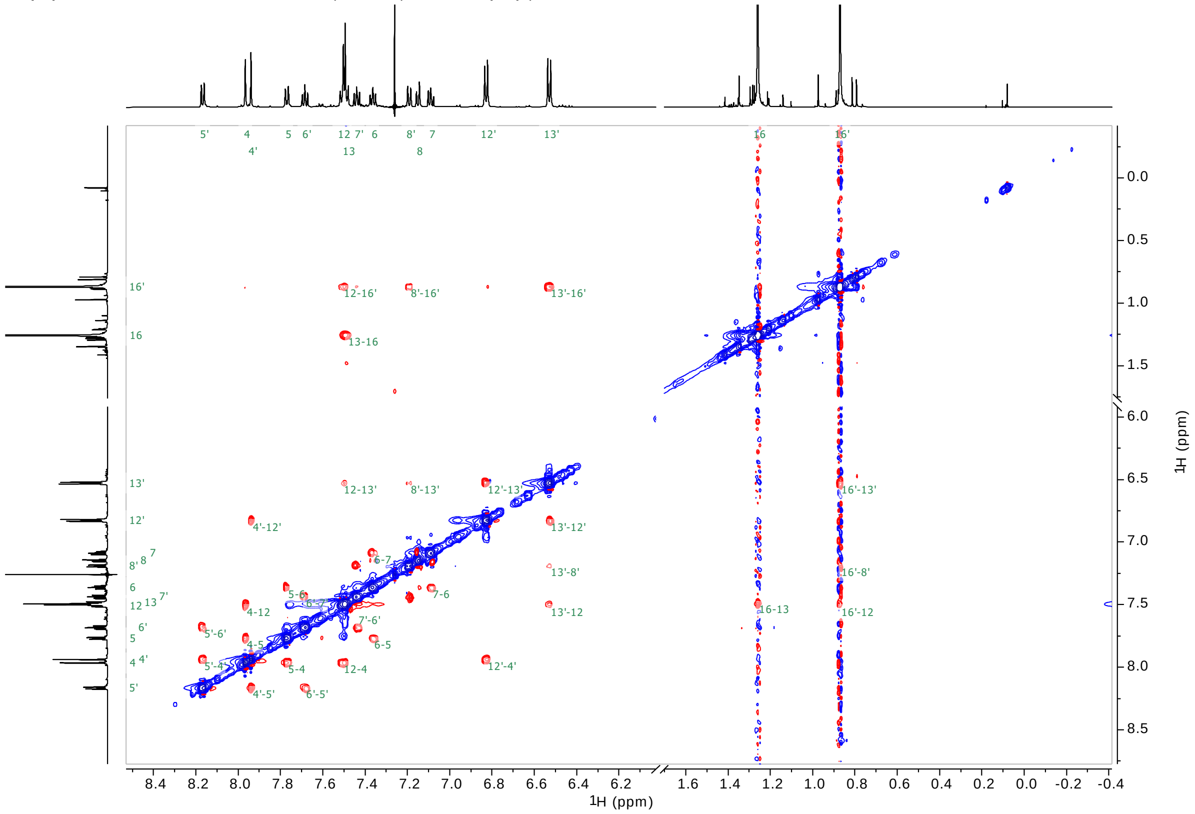

31P{1H},1D, 242.98 MHz,CDCl3,298.0K, pulse sequence: zgpg30

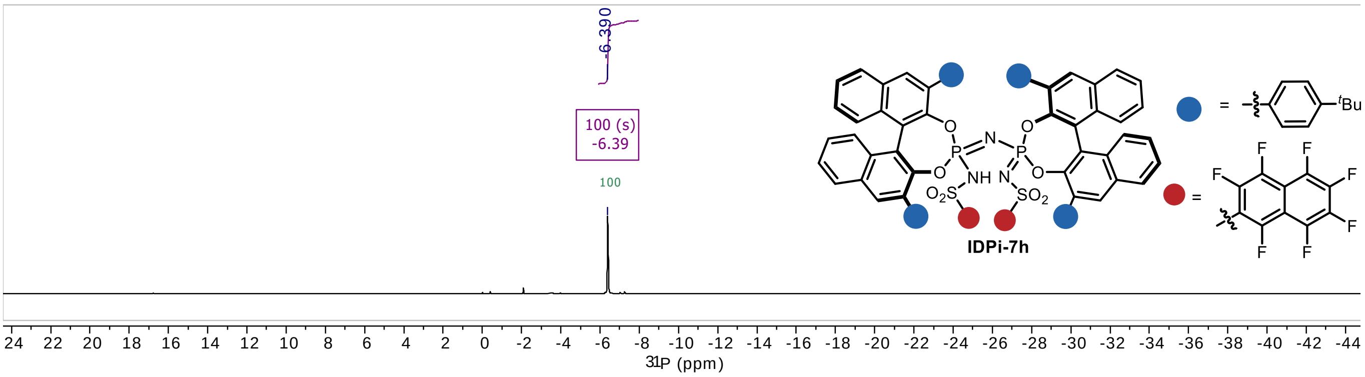

1H{31P},HMBC, 600.20 MHz,CDCl3,298.0K, pulse sequence: hmbcgpndqf

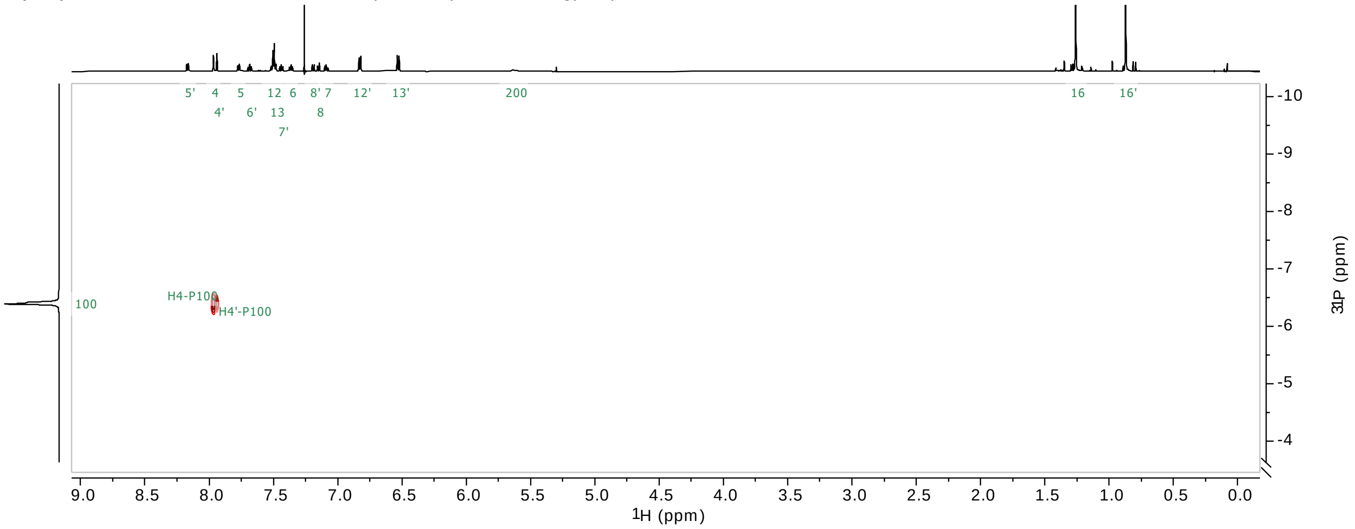

19F{off},1D, 564.72 MHz,CDCl3,298.0K, pulse sequence: zg30

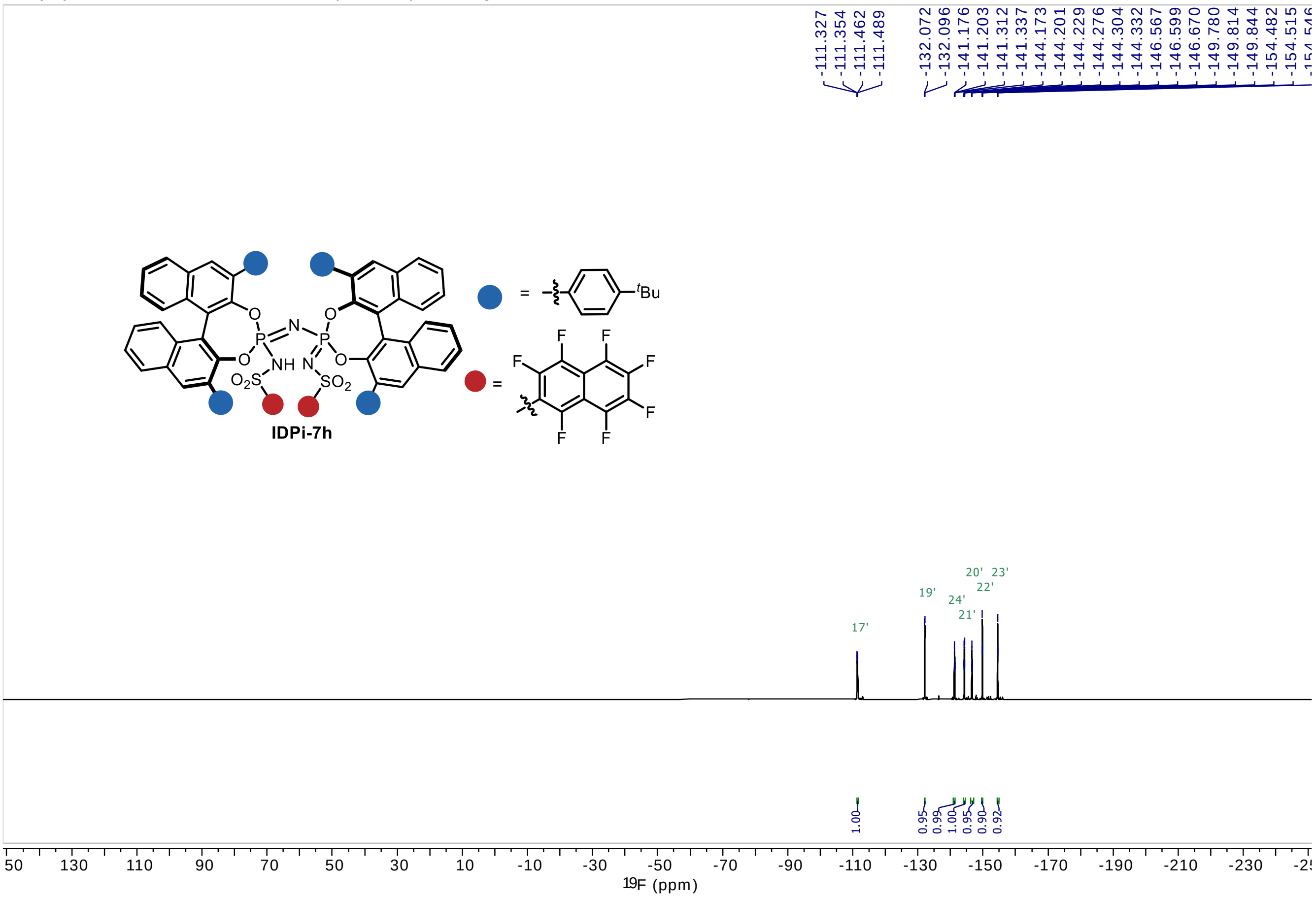

19F{off},1D, 564.72 MHz,CDCl3,298.0K, pulse sequence: zg30

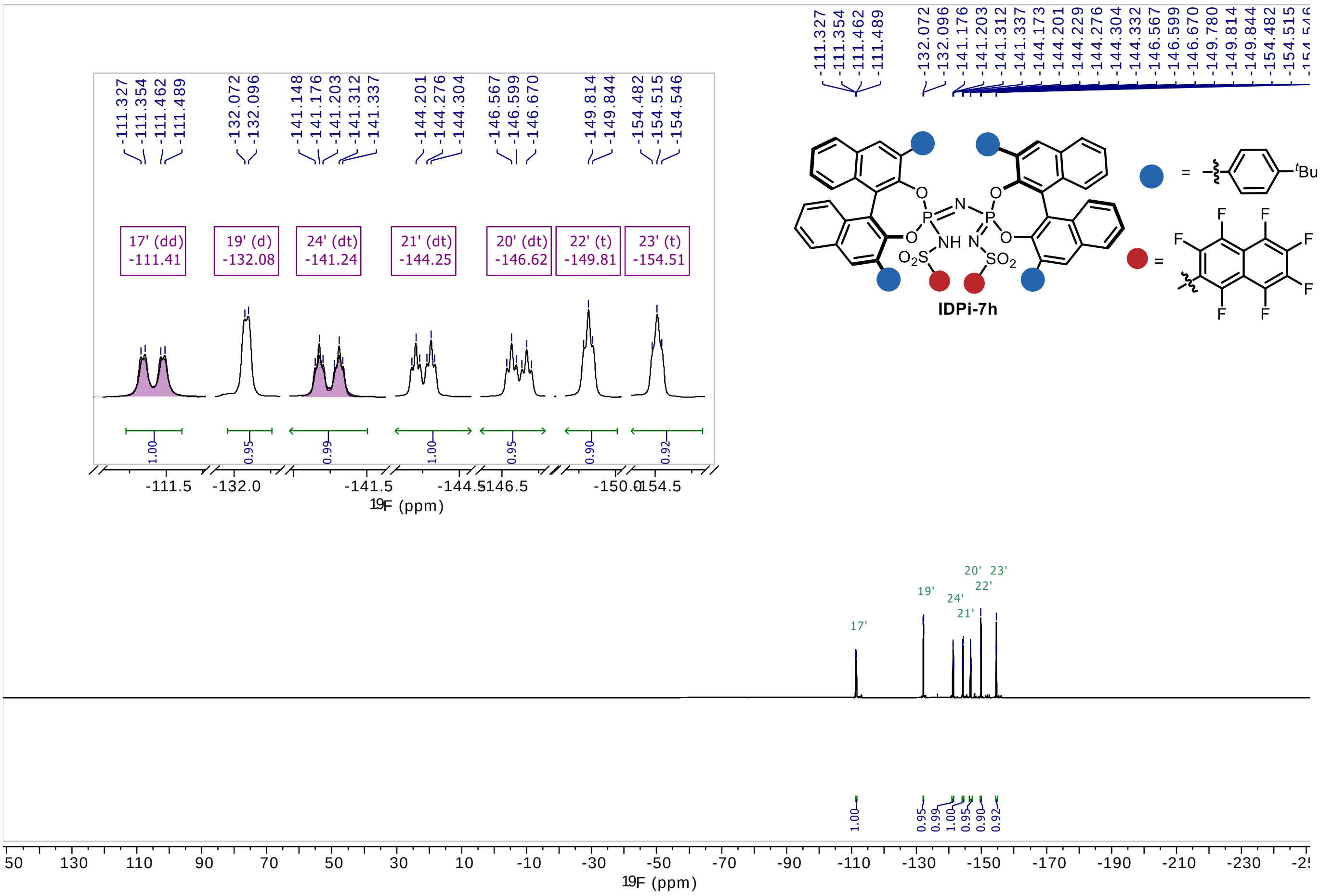

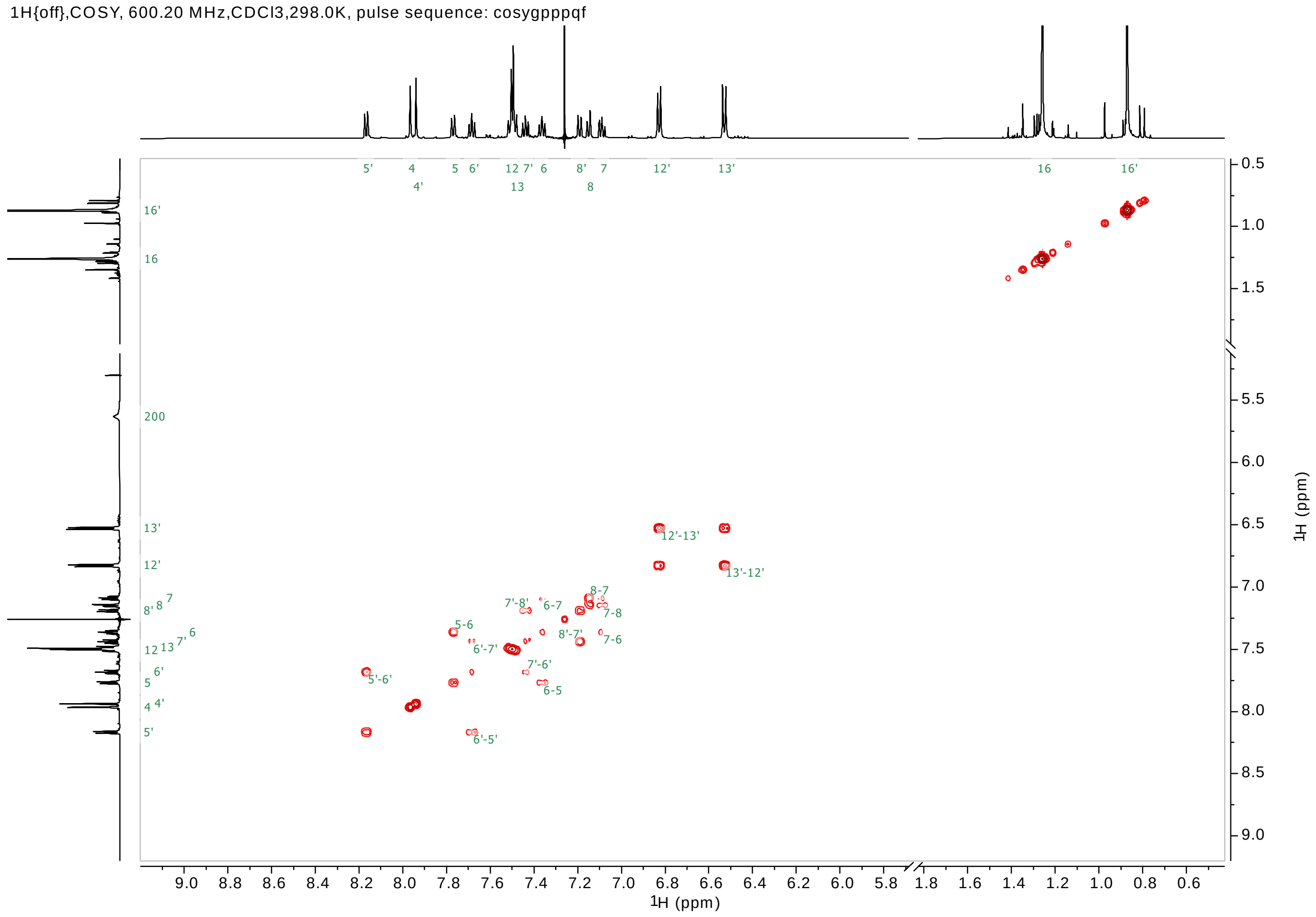
1H{off},COSY, 600.20 MHz,CDCl3,298.0K, pulse sequence: cosygpppqf
1H (ppm)

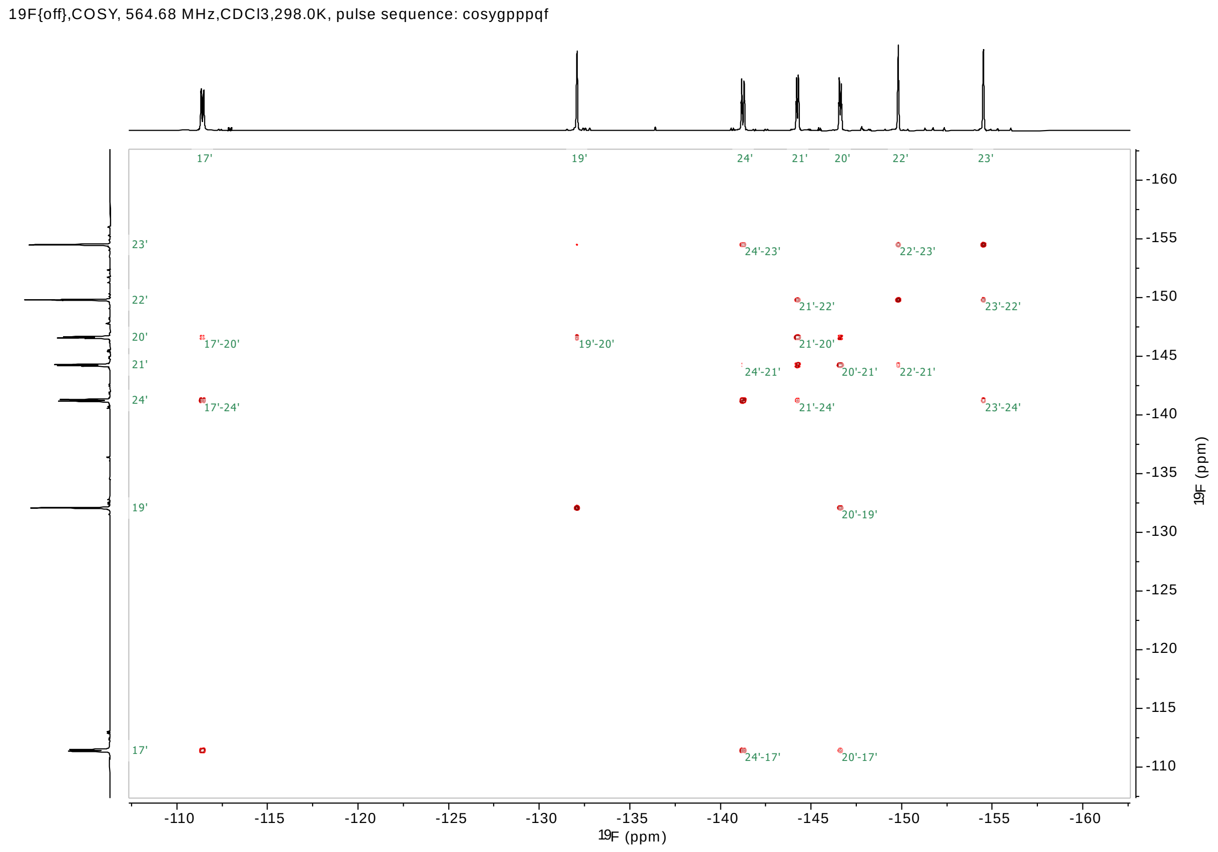
19F{off},COSY, 564.68 MHz,CDCl3,298.0K, pulse sequence: cosygpppqf
19F (ppm)

New Substrate Charecterization:

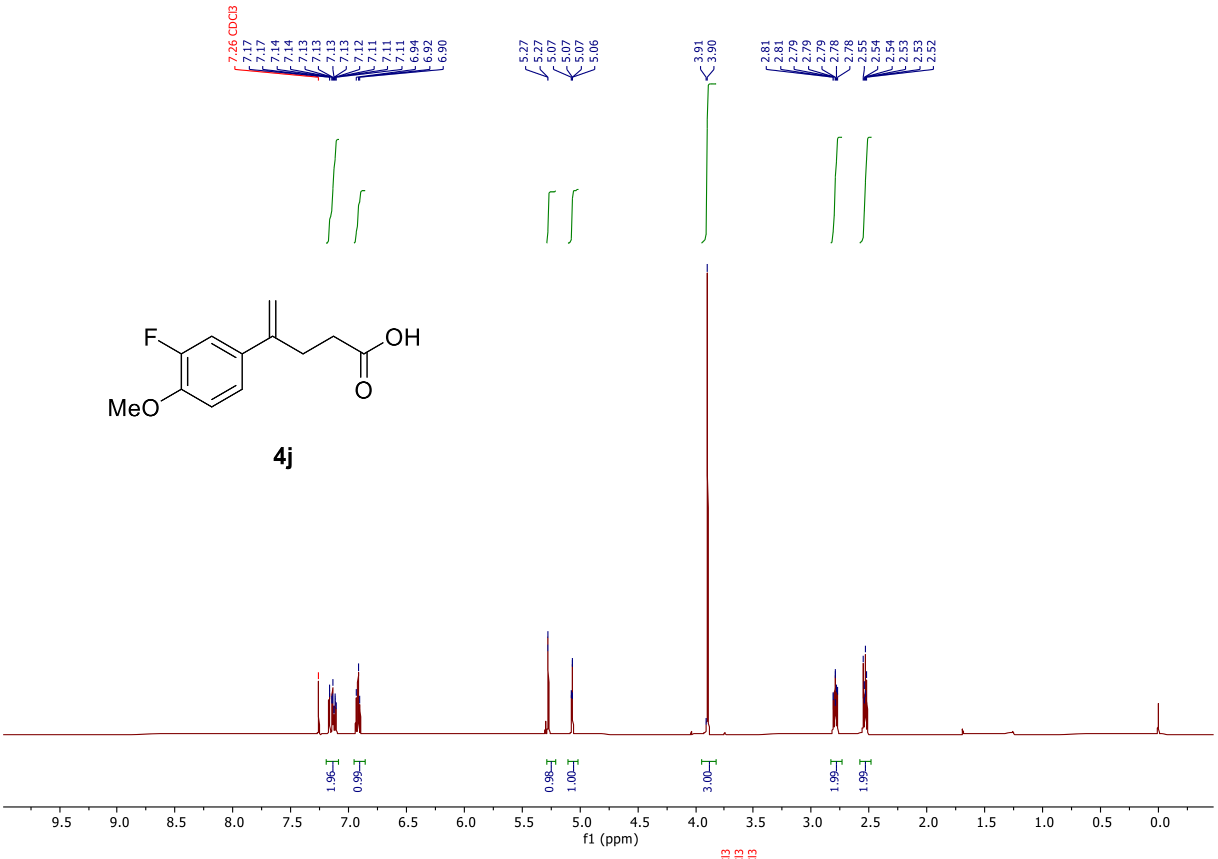

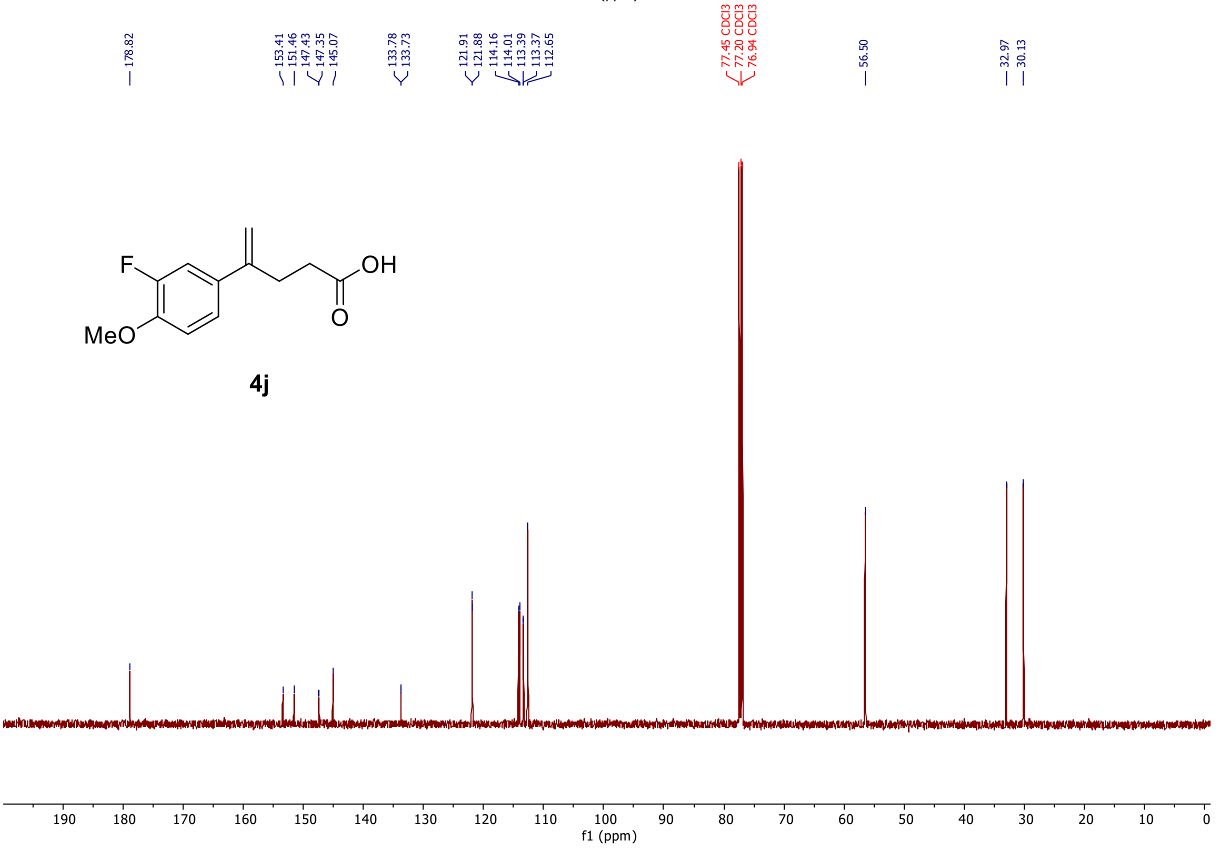

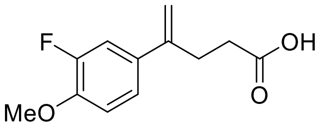

4j

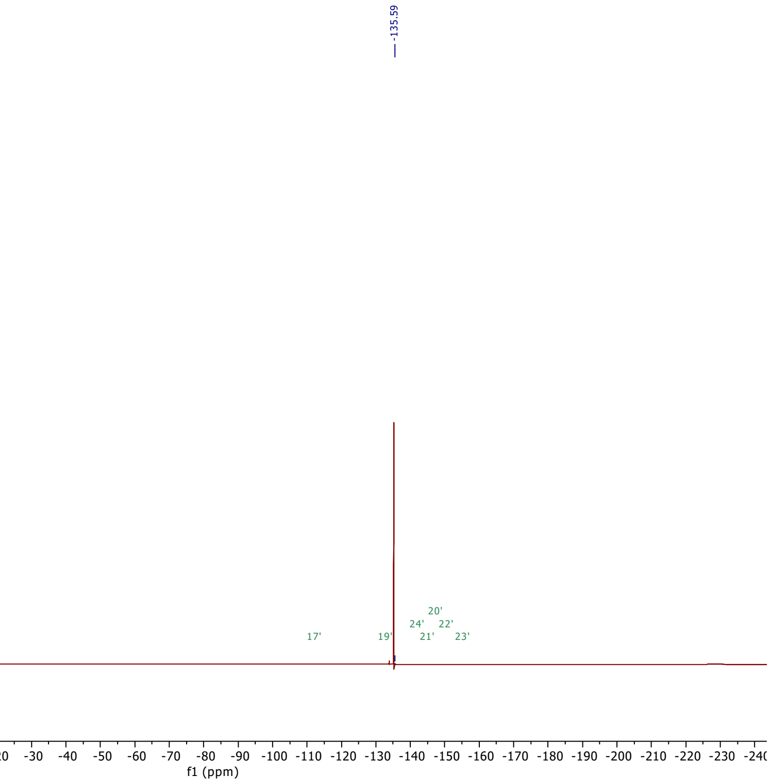
-135.59
20'
24' 22'
17' 19' 21' 23'
70 60 50 40 30 20 10 0 -10 -20 -30 -40 -50 -60 -70 -80 -90 -100 -110 -120 -130 -140 -150 -160 -170 -180 -190 -200 -210 -220 -230
f1 (ppm)

Chiral lactone Charecterization:

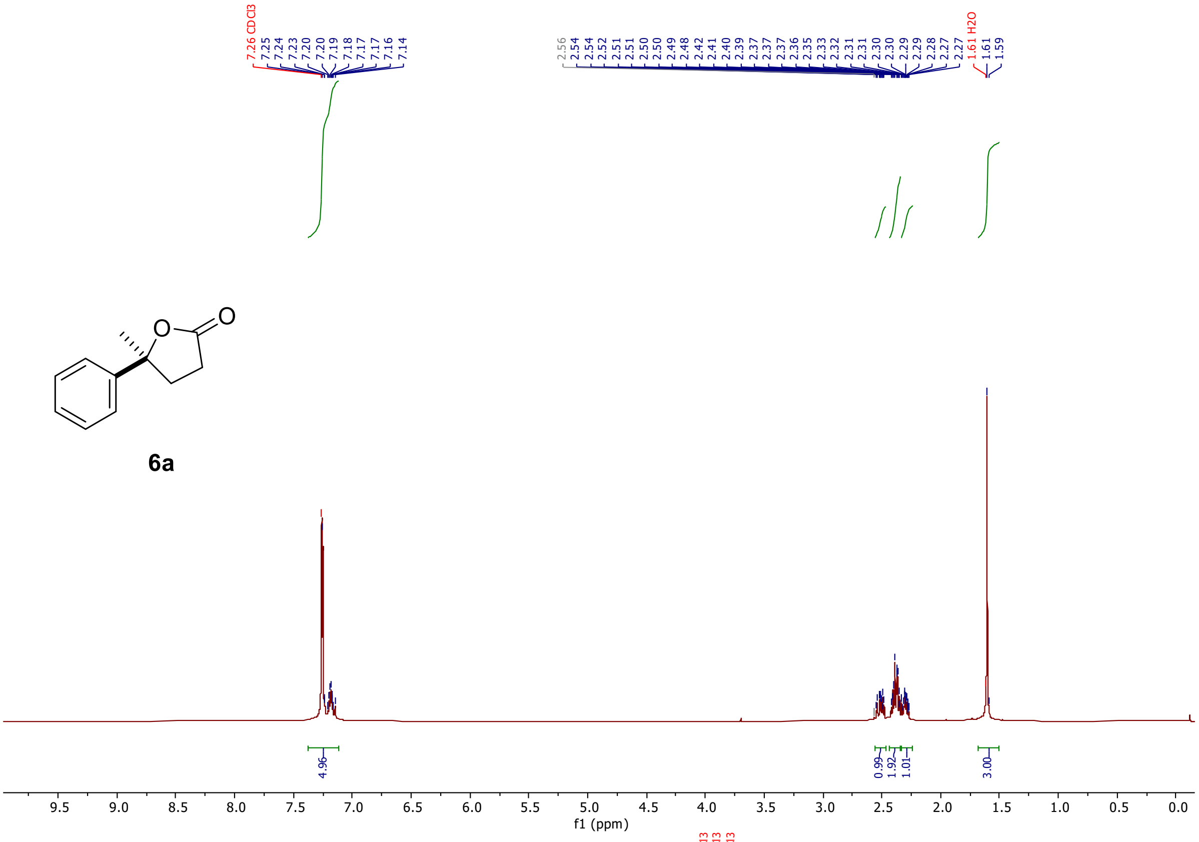

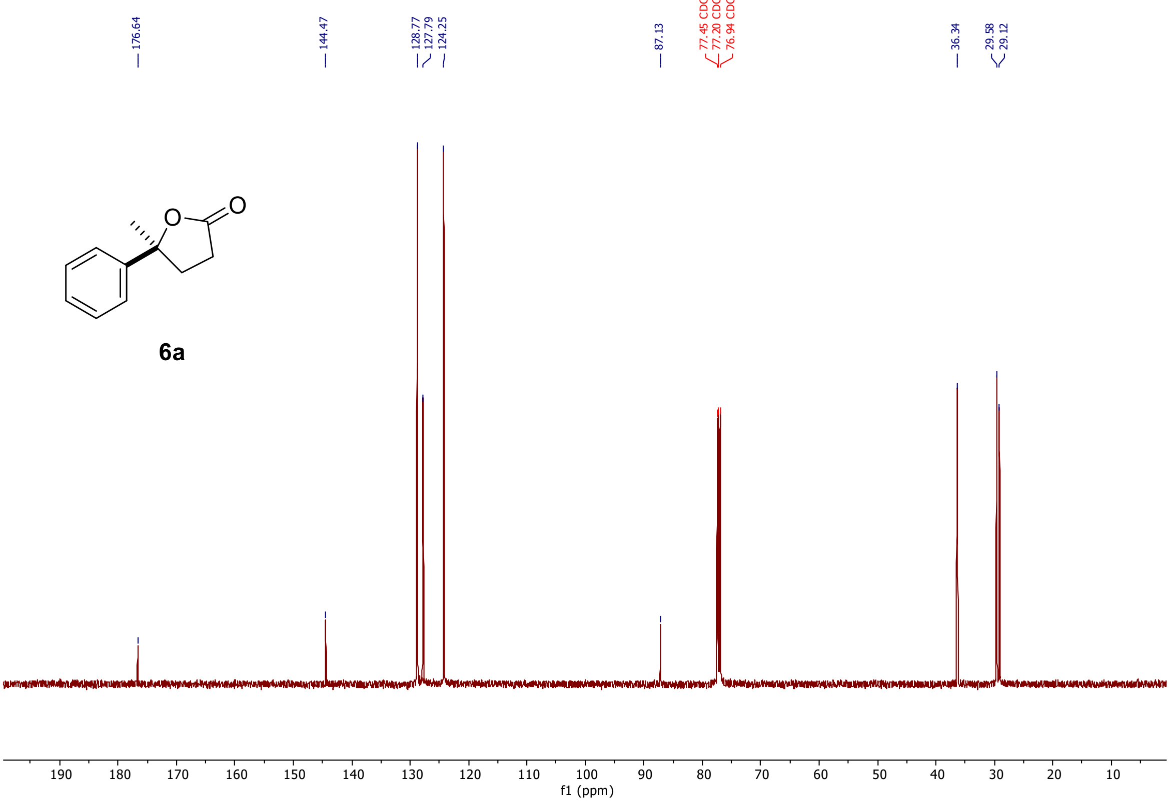

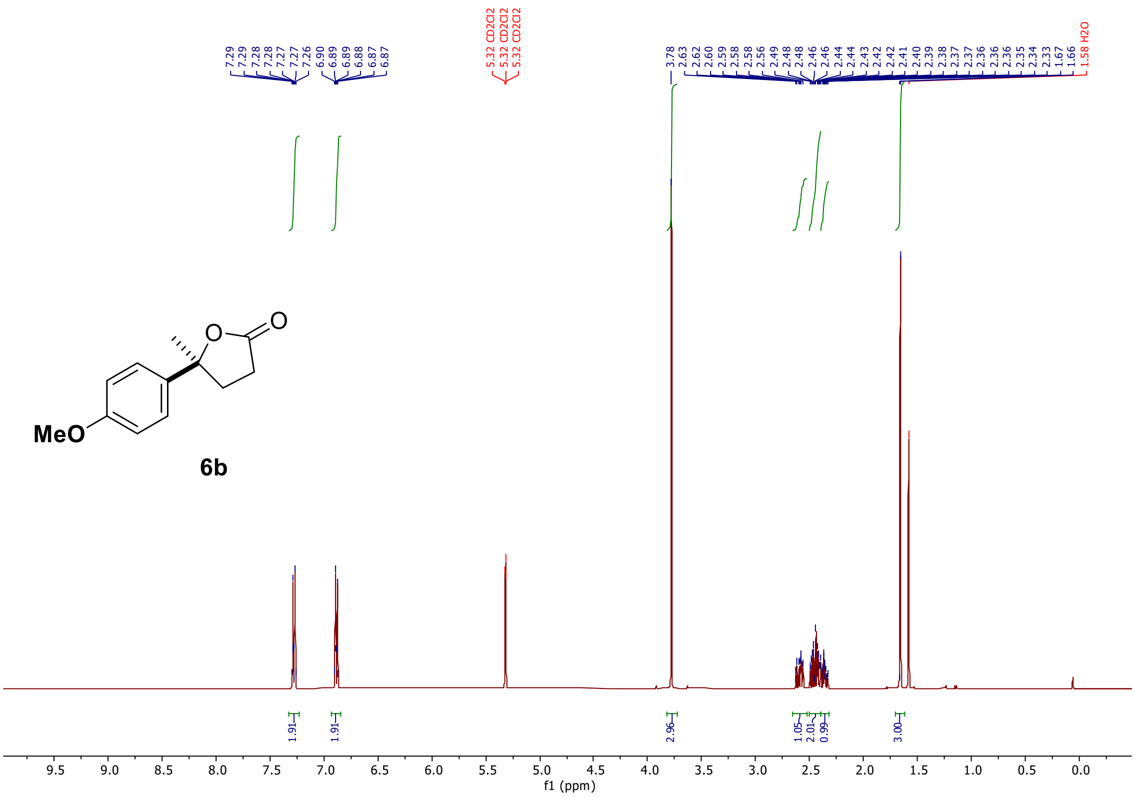

7.29
7.29
7.28
7.28
7.27
7.27
7.26
6.90
6.89
6.89
6.88
6.87
6.87
5.32 CD2Cl2
5.32 CD2Cl2
5.32 CD2Cl2
3.78
2.63
2.62
2.60
2.59
2.58
2.58
2.56
2.49
2.48
2.48
2.46
2.46
2.44
2.44
2.43
2.42
2.42
2.41
2.40
2.39
2.38
2.37
2.37
2.36
2.36
2.35
2.34
2.33
1.67
1.66
1.58 H2O
MeO
6b
1.91
1.91
2.96
1.05
2.01
0.99
3.00
f1 (ppm)

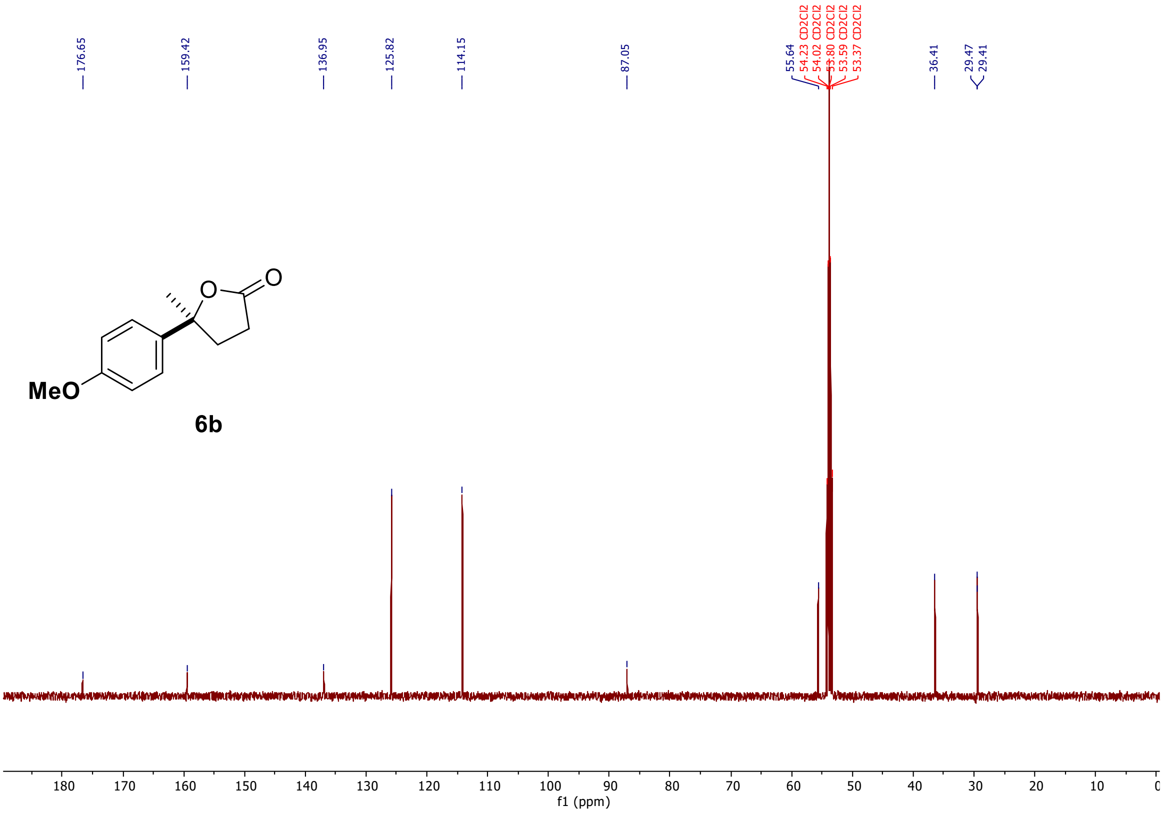

176.65
159.42
136.95
125.82
114.15
87.05
55.64
54.23 CD2Cl2
54.02 CD2Cl2
53.80 CD2Cl2
53.59 CD2Cl2
53.37 CD2Cl2
36.41
29.47
29.41
MeO
6b
f1 (ppm)

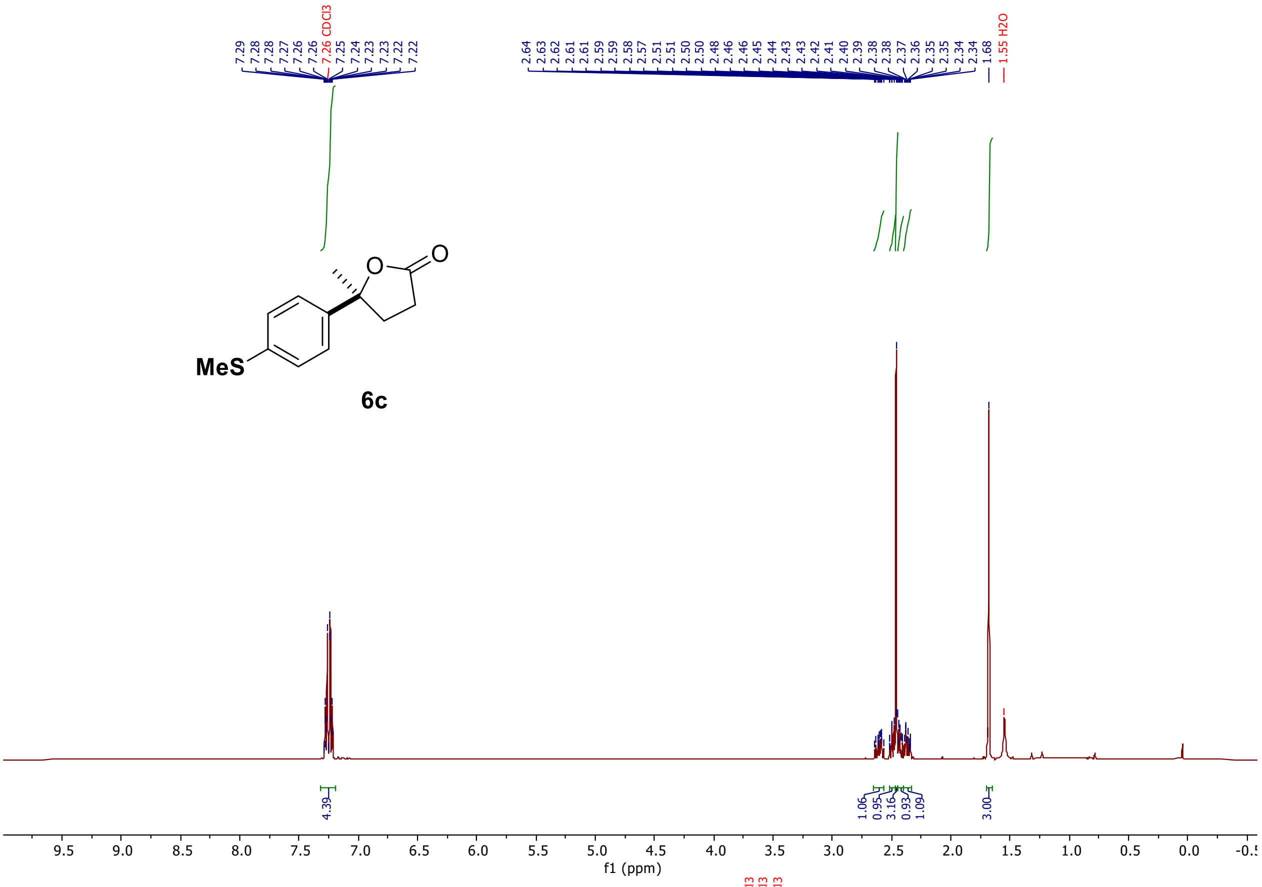
7.29
7.28
7.28
7.27
7.26
7.26
7.26 CDCl3
7.25
7.24
7.23
7.23
7.22
7.22
2.64
2.63
2.62
2.61
2.61
2.59
2.59
2.58
2.57
2.51
2.51
2.50
2.50
2.48
2.46
2.46
2.45
2.44
2.43
2.43
2.42
2.41
2.40
2.39
2.38
2.38
2.37
2.36
2.35
2.35
2.34
2.34
1.68
1.55 H2O
MeS
O
O
6c
4.39
1.06
0.95
3.16
0.93
1.09
3.00
9.5
9.0
8.5
8.0
7.5
7.0
6.5
6.0
5.5
5.0
4.5
4.0
3.5
3.0
2.5
2.0
1.5
1.0
0.5
0.0
f1 (ppm)

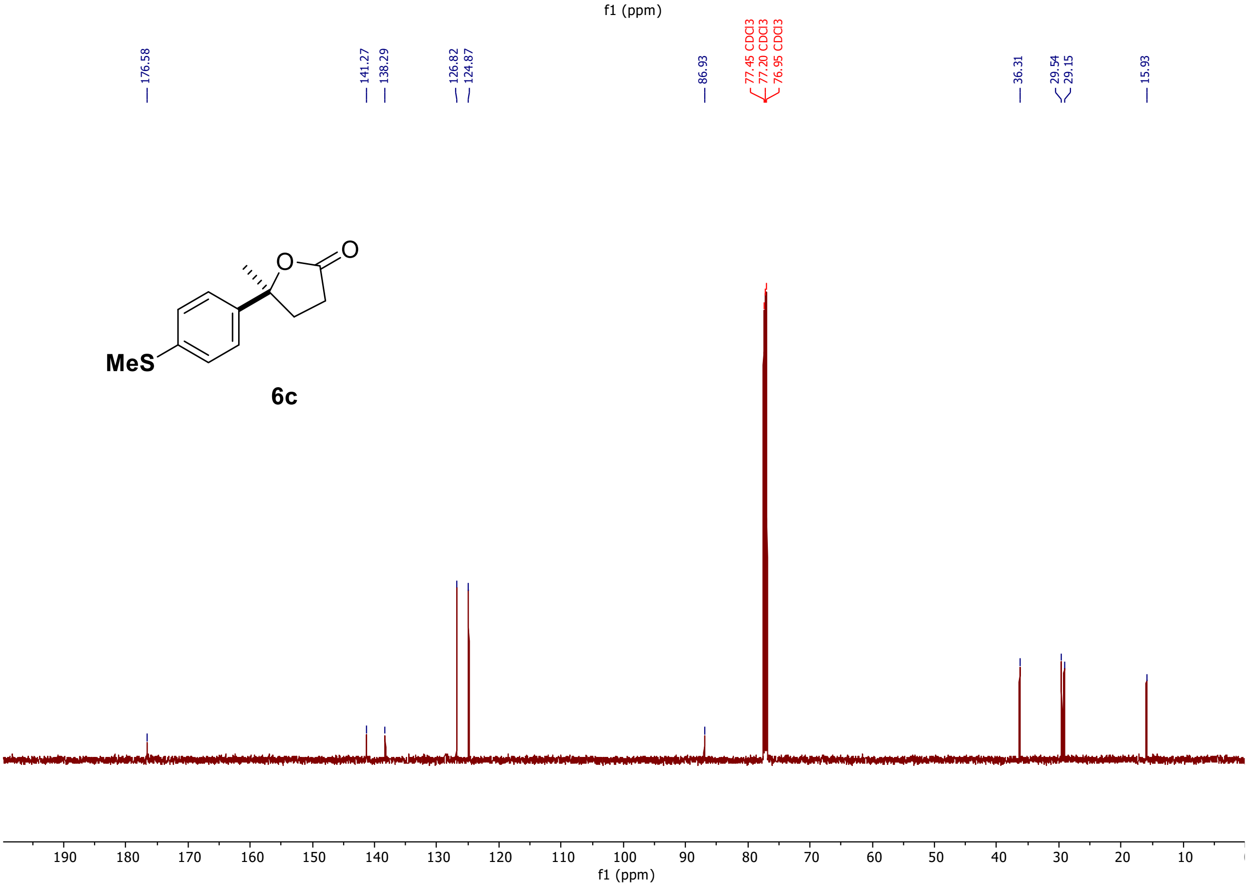
176.58
141.27
138.29
126.82
124.87
86.93
77.45 CDCl3
77.20 CDCl3
76.95 CDCl3
36.31
29.54
29.15
15.93
MeS
O
O
6c
190
180
170
160
150
140
130
120
110
100
90
80
70
60
50
40
30
20
10
f1 (ppm)

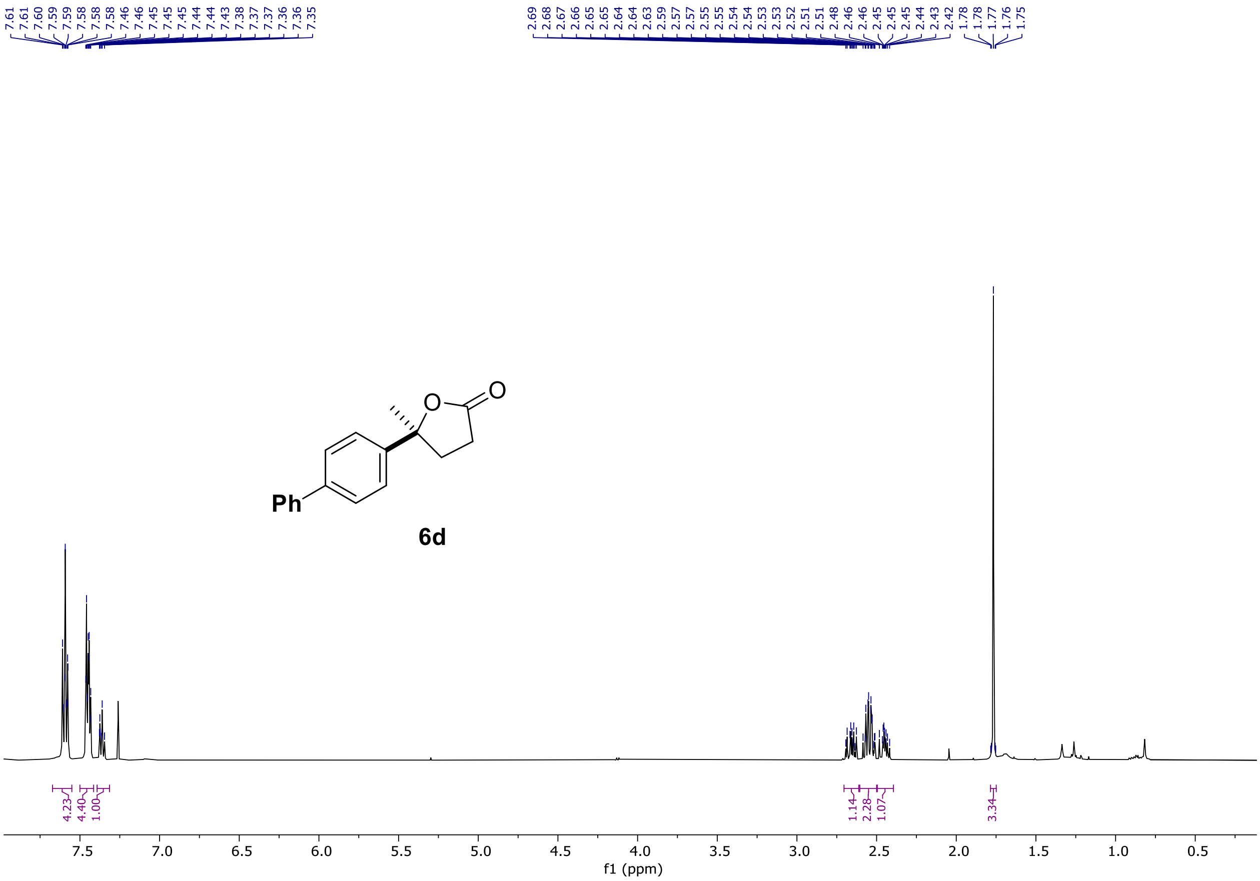
7.61 7.61 7.60 7.59 7.59 7.58 7.58 7.58 7.46 7.46 7.45 7.45 7.45 7.44 7.44 7.43 7.38 7.37 7.37 7.36 7.35
2.69 2.68 2.67 2.66 2.65 2.65 2.64 2.64 2.63 2.59 2.57 2.57 2.55 2.55 2.54 2.54 2.53 2.53 2.52 2.51 2.51 2.48 2.46 2.46 2.45 2.45 2.45 2.45 2.44 2.43 2.42 1.78 1.78 1.77 1.76 1.75
O
O
Ph
6d
4.23
4.40
1.00
1.14
2.28
1.07
3.34
7.5 7.0 6.5 6.0 5.5 5.0 4.5 4.0 3.5 3.0 2.5 2.0 1.5 1.0 0.5
f1 (ppm)

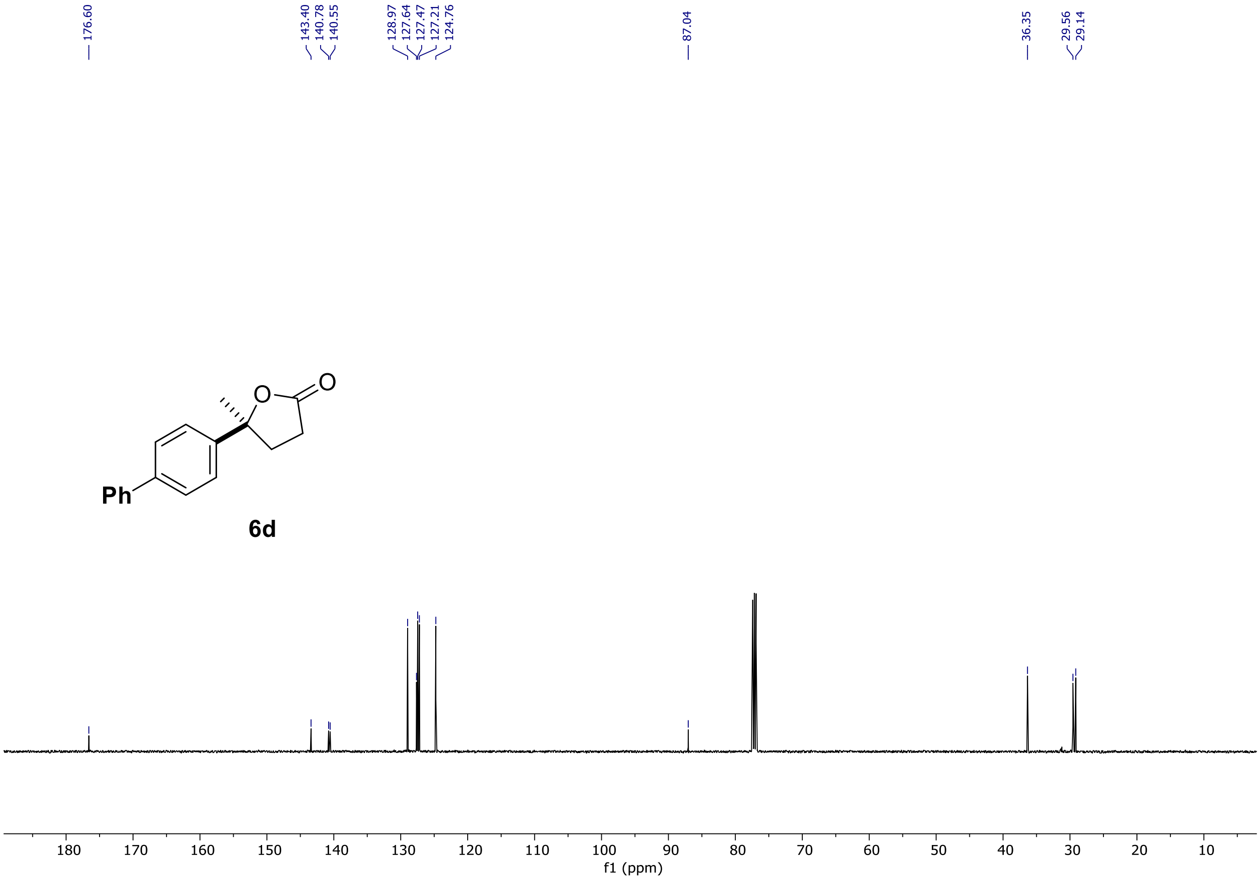
176.60
143.40 140.78 140.55
128.97 127.64 127.47 127.21 124.76
87.04
36.35
29.56 29.14
O
O
Ph
6d
180 170 160 150 140 130 120 110 100 90 80 70 60 50 40 30 20 10
f1 (ppm)

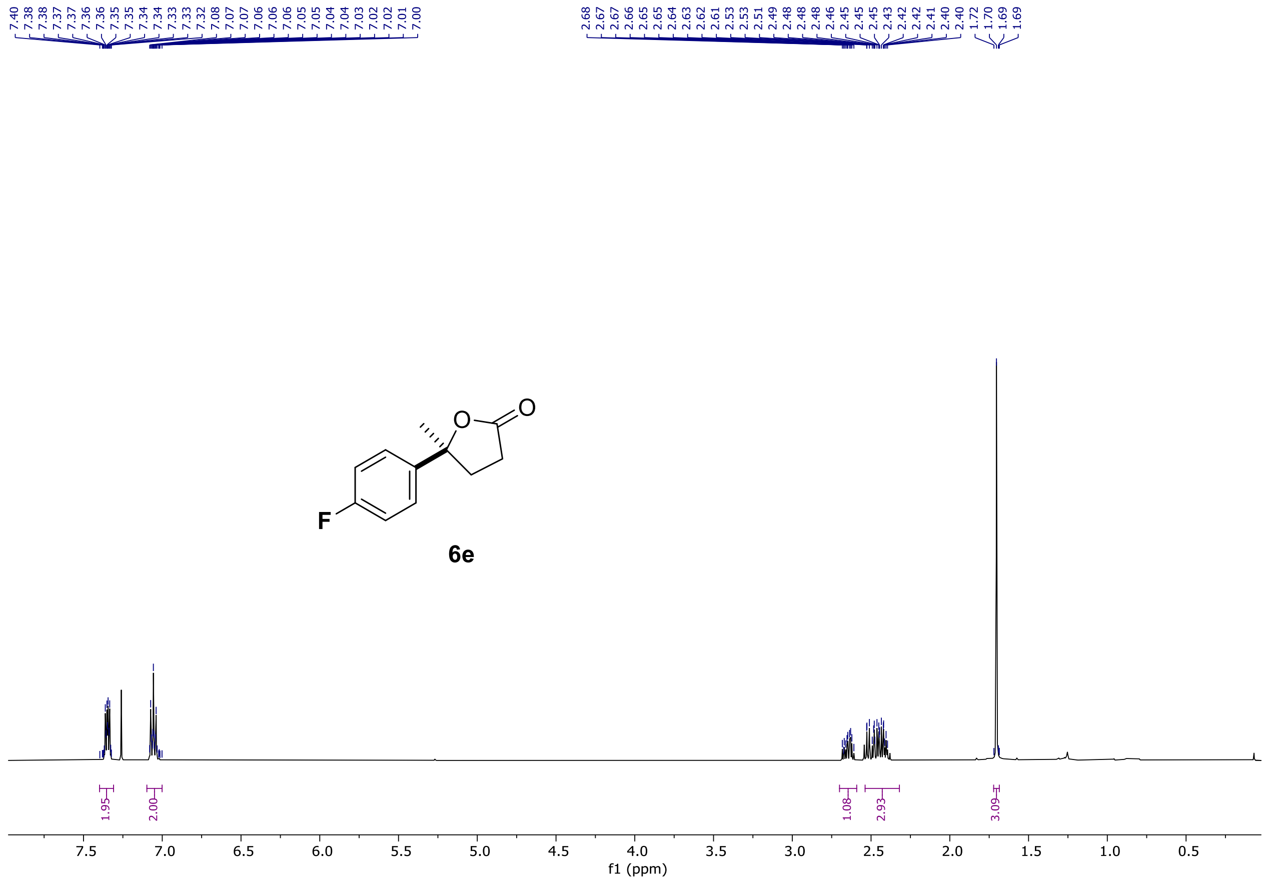
7.40 7.38 7.38 7.37 7.37 7.36 7.36 7.35 7.35 7.34 7.34 7.33 7.33 7.32 7.08 7.07 7.07 7.06 7.06 7.05 7.05 7.04 7.04 7.03 7.02 7.02 7.01 7.00
2.68 2.67 2.67 2.66 2.65 2.65 2.64 2.63 2.62 2.61 2.53 2.53 2.53 2.51 2.49 2.48 2.48 2.48 2.46 2.45 2.45 2.45 2.43 2.42 2.42 2.41 2.40 1.72 1.70 1.69 1.69
O
O
F
6e
1.95
2.00
1.08
2.93
3.09
f1 (ppm)

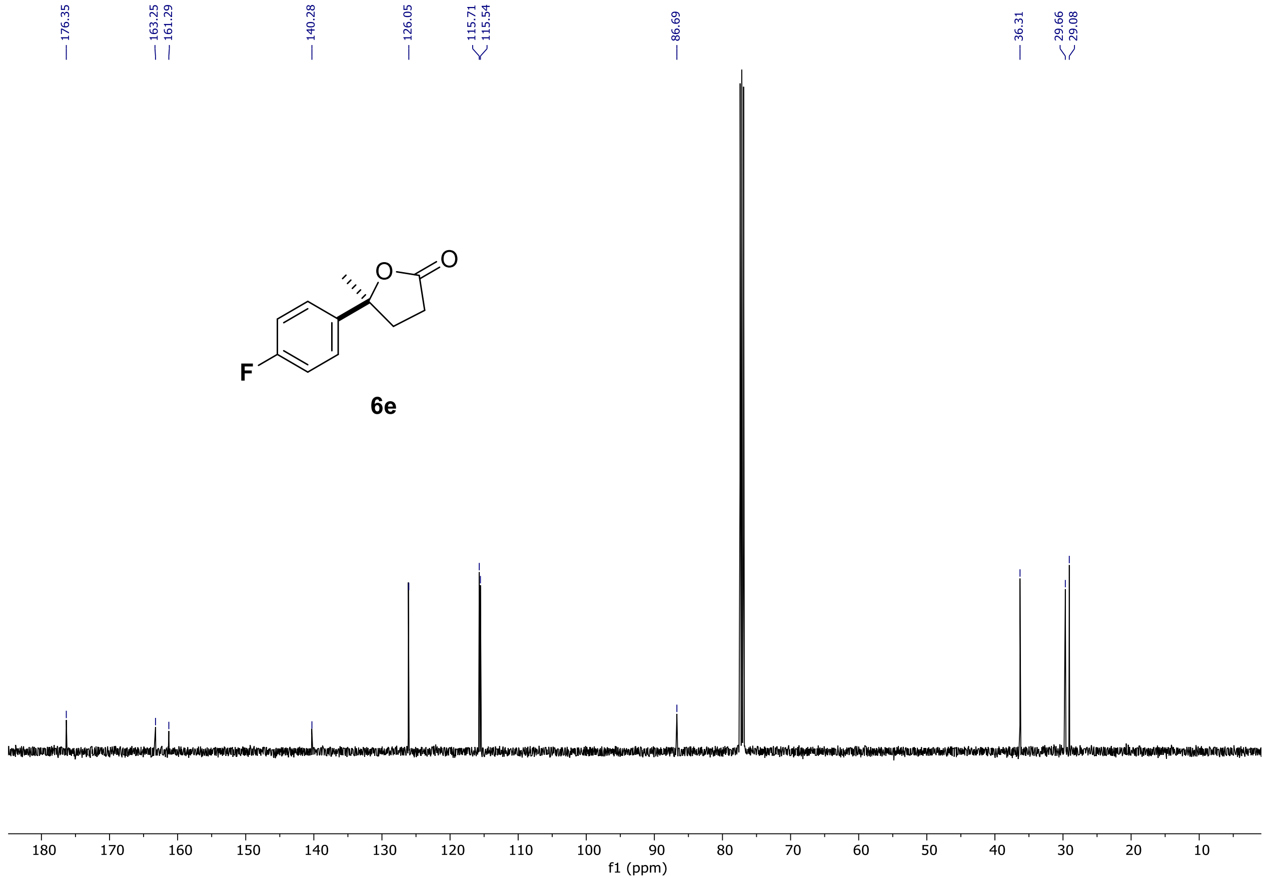
176.35
163.25
161.29
140.28
126.05
115.71
115.54
86.69
36.31
29.66
29.08
O
O
F
6e
f1 (ppm)

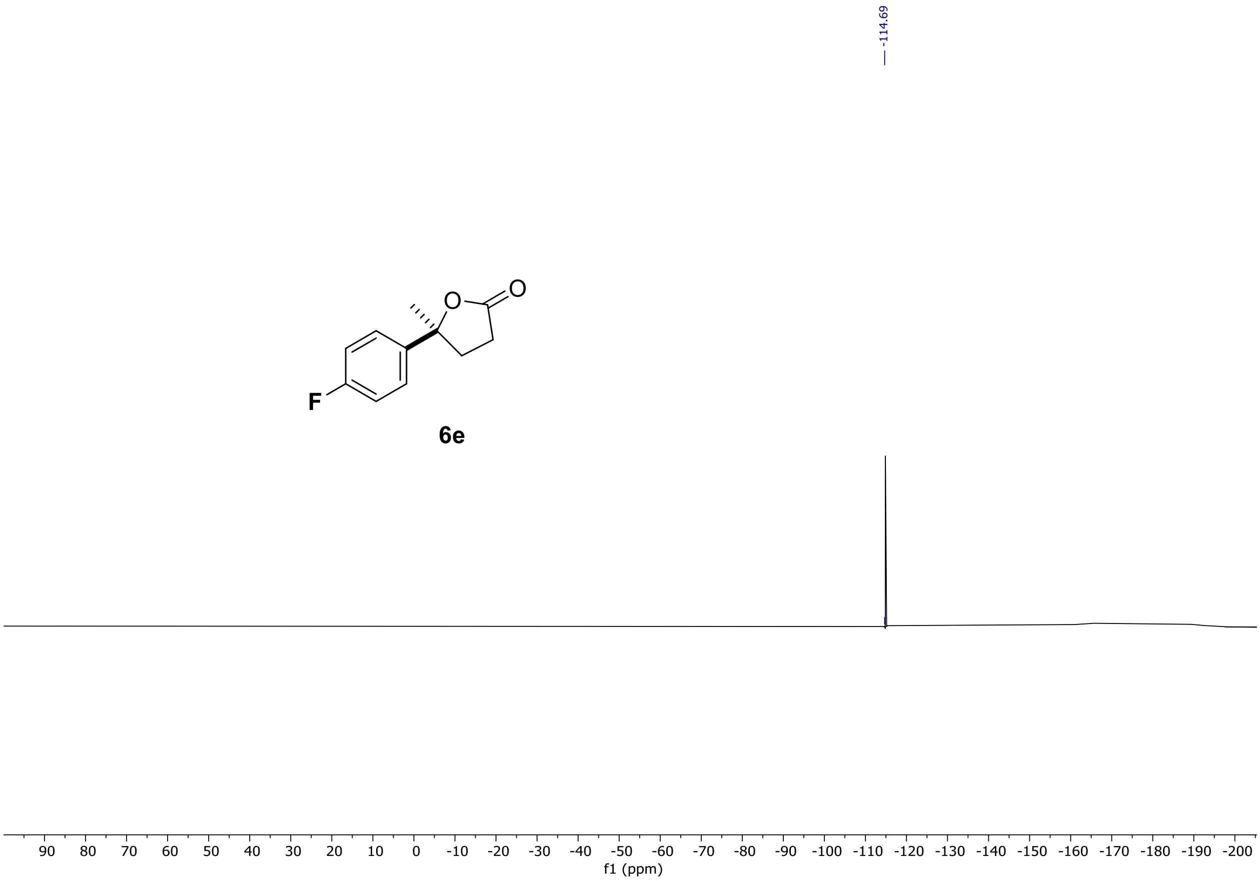
6e
-114.69
90 80 70 60 50 40 30 20 10 0 -10 -20 -30 -40 -50 -60 -70 -80 -90 -100 -110 -120 -130 -140 -150 -160 -170 -180 -190 -200
f1 (ppm)

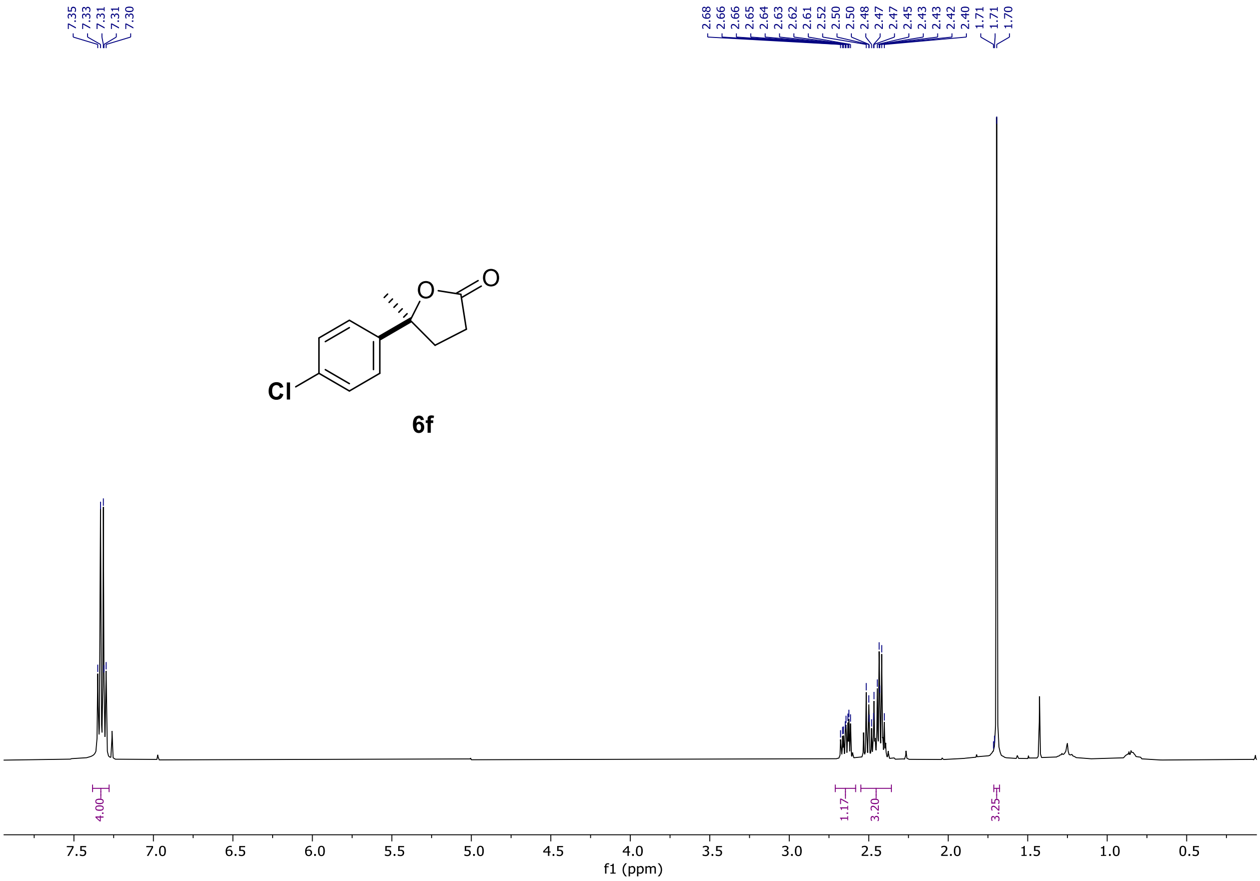
6f
7.35 7.33 7.31 7.31 7.30
2.68 2.66 2.66 2.65 2.64 2.63 2.62 2.61 2.52 2.50 2.50 2.48 2.47 2.47 2.45 2.43 2.43 2.42 2.40 1.71 1.71 1.70
4.00
1.17 3.20
3.25
7.5 7.0 6.5 6.0 5.5 5.0 4.5 4.0 3.5 3.0 2.5 2.0 1.5 1.0 0.5
f1 (ppm)

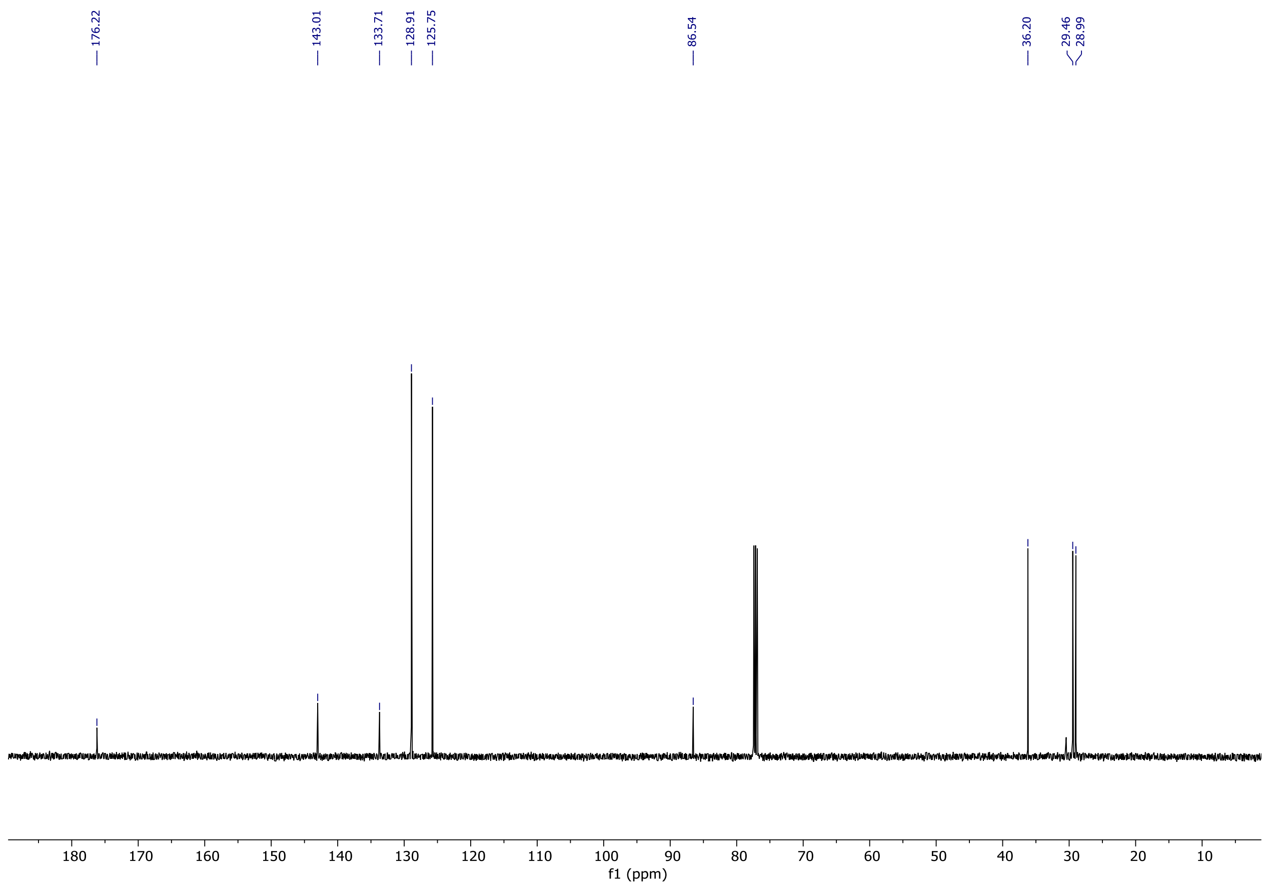
176.22
143.01
133.71
128.91
125.75
86.54
36.20
29.46
28.99
180 170 160 150 140 130 120 110 100 90 80 70 60 50 40 30 20 10
f1 (ppm)

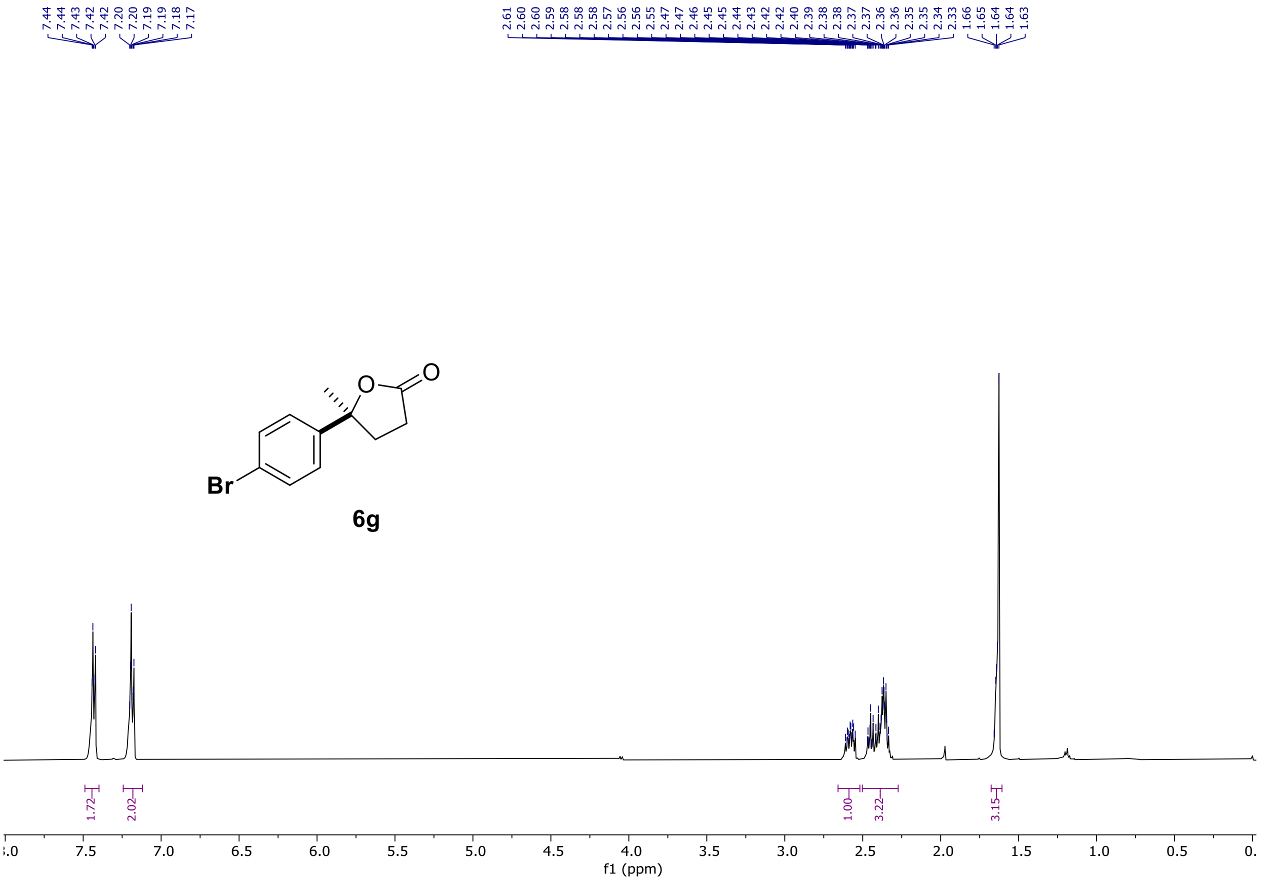
7.44 7.44 7.43 7.42 7.42 7.20 7.19 7.19 7.18 7.17
2.61 2.60 2.60 2.59 2.58 2.58 2.58 2.57 2.56 2.56 2.55 2.47 2.47 2.46 2.45 2.45 2.44 2.43 2.42 2.42 2.40 2.39 2.38 2.38 2.37 2.37 2.36 2.36 2.35 2.35 2.34 2.33 1.66 1.65 1.64 1.64 1.63
O
O
Br
6g
1.72
2.02
1.00
3.22
3.15
f1 (ppm)

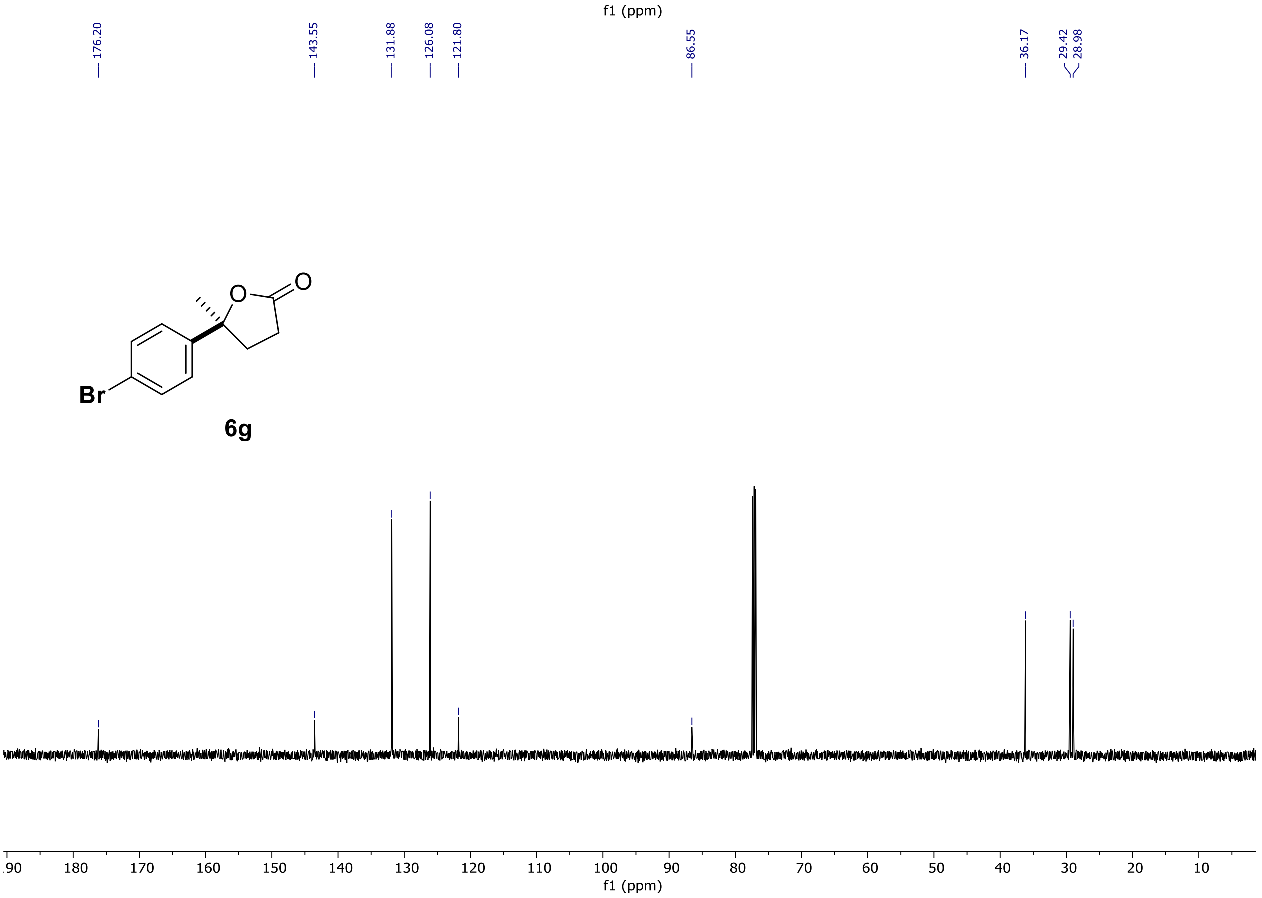
176.20
143.55
131.88
126.08
121.80
86.55
36.17
29.42
28.98
O
O
Br
6g
f1 (ppm)

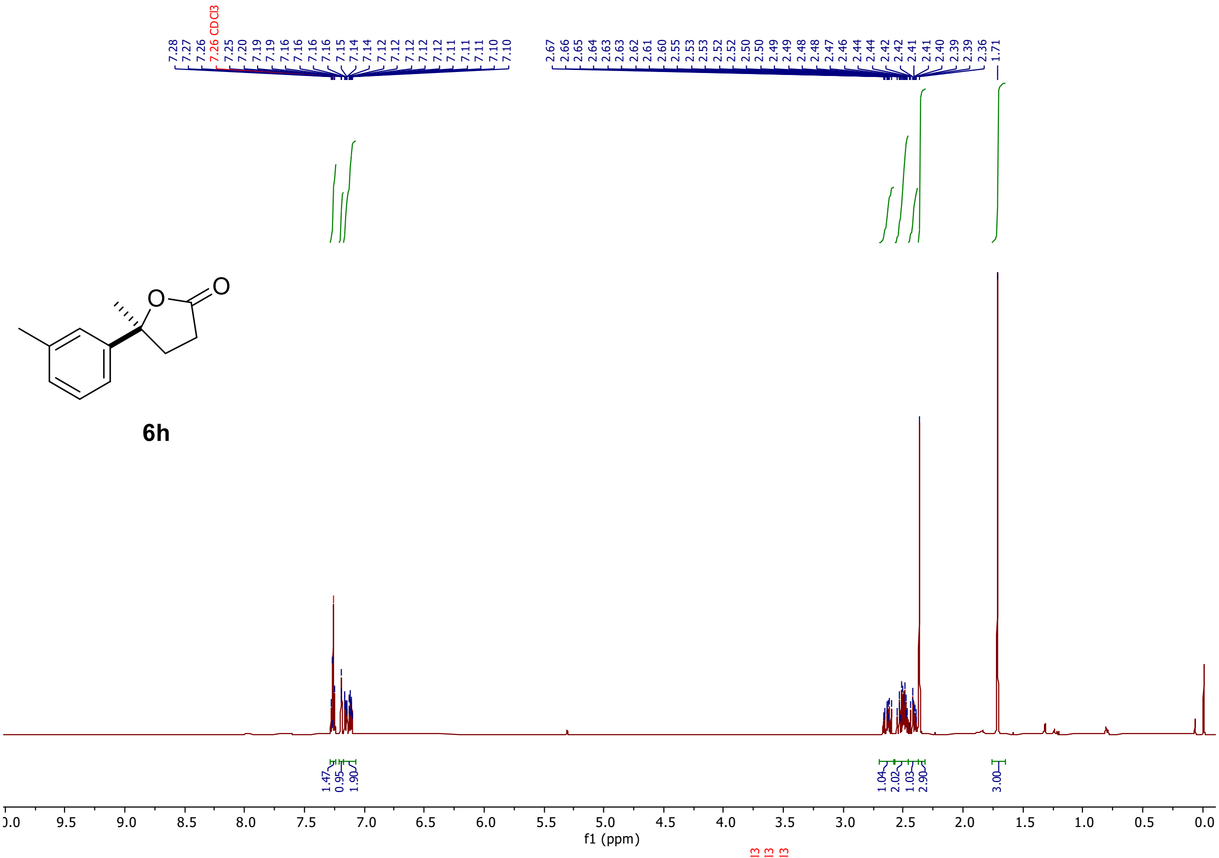
7.28
7.27
7.26
7.26 CDCl3
7.25
7.20
7.19
7.19
7.16
7.16
7.16
7.15
7.14
7.14
7.12
7.12
7.12
7.12
7.12
7.11
7.11
7.11
7.10
7.10
2.67
2.66
2.65
2.64
2.63
2.63
2.62
2.61
2.60
2.55
2.53
2.53
2.52
2.52
2.50
2.50
2.49
2.49
2.48
2.47
2.46
2.44
2.44
2.42
2.42
2.41
2.41
2.40
2.39
2.39
2.36
1.71
6h
1.47
0.95
1.90
1.04
2.02
1.03
2.90
3.00
f1 (ppm)

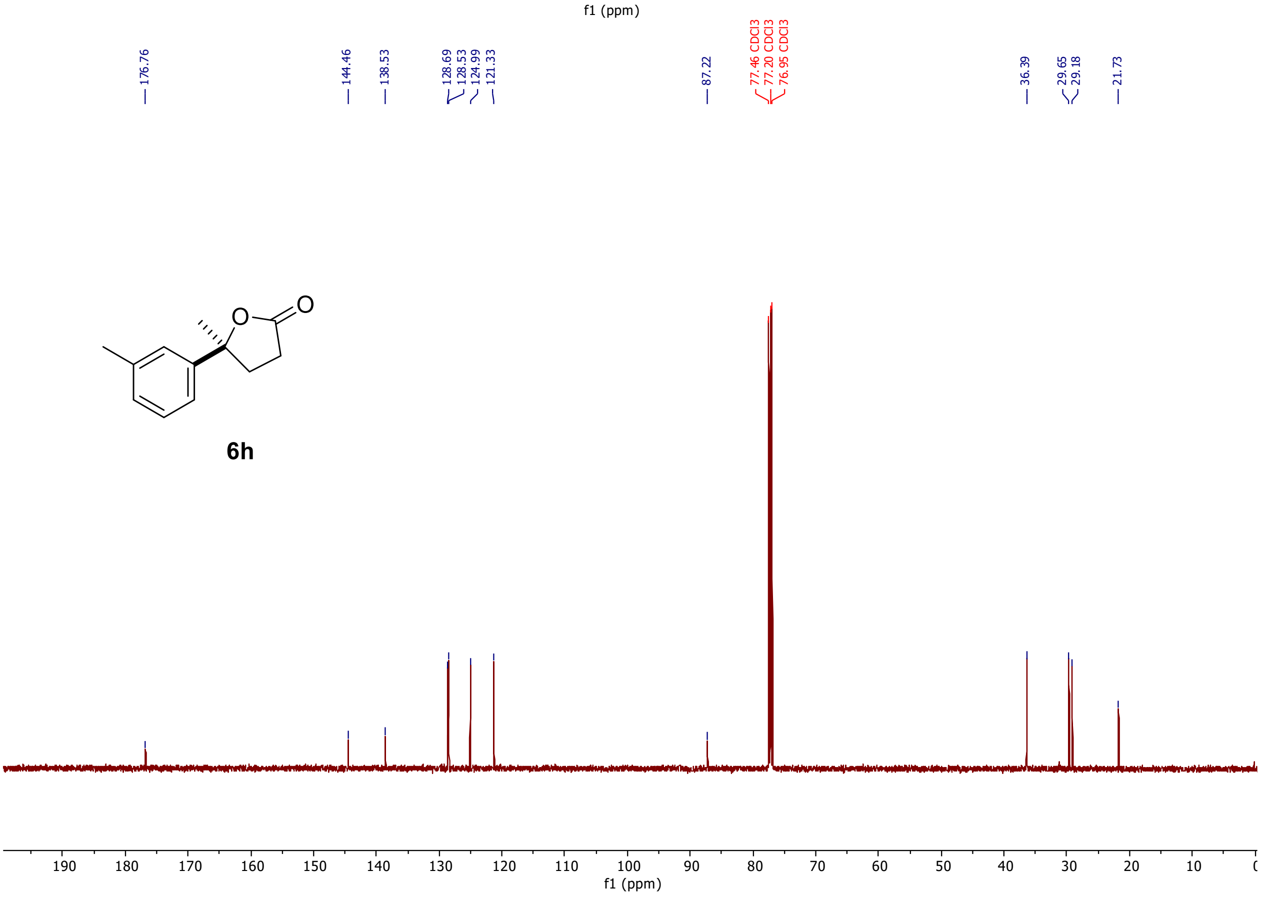
176.76
144.46
138.53
128.69
128.53
124.99
121.33
87.22
77.46 CDCl3
77.20 CDCl3
76.95 CDCl3
36.39
29.65
29.18
21.73
6h
f1 (ppm)

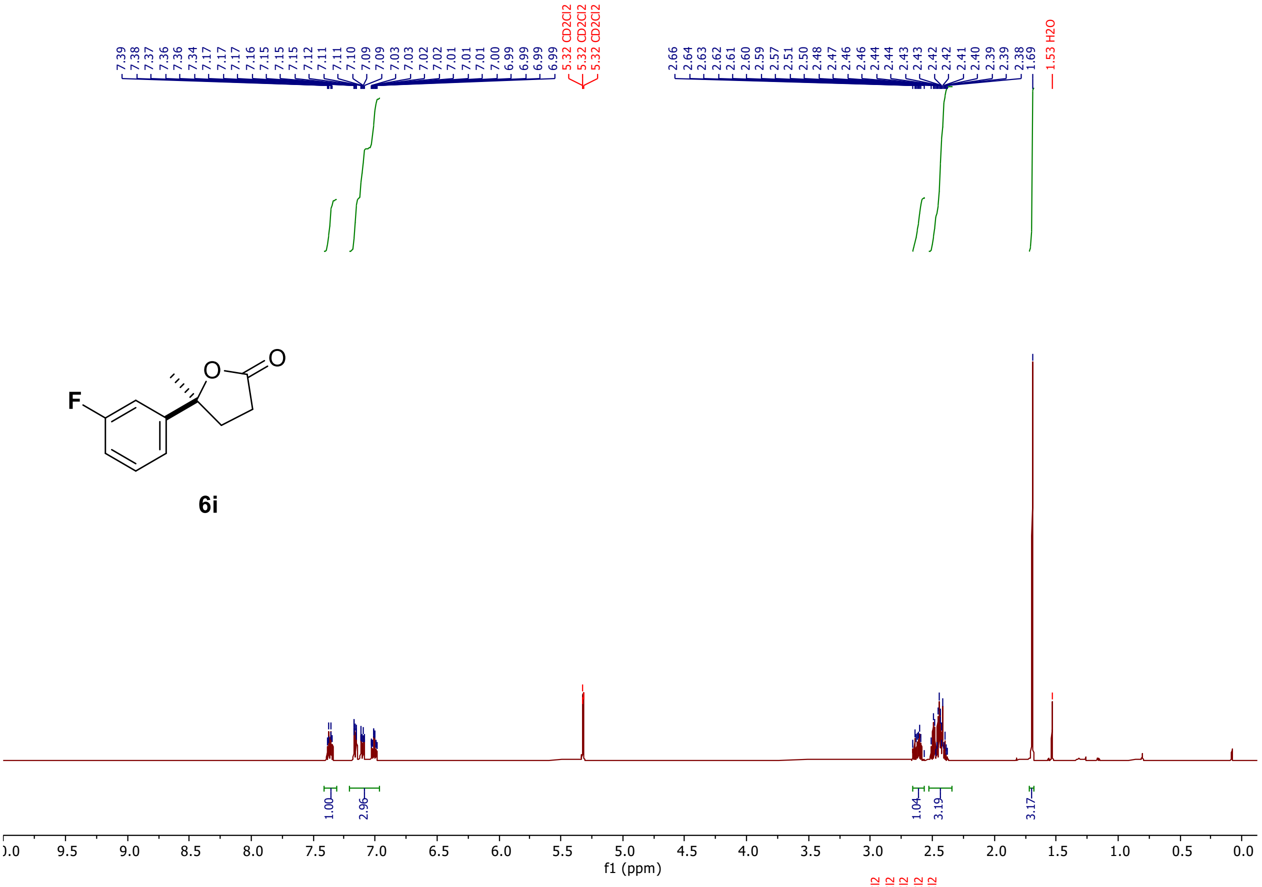

7.39 7.38 7.37 7.36 7.36 7.34 7.17 7.17 7.17 7.16 7.15 7.15 7.15 7.12 7.11 7.11 7.10 7.09 7.09 7.03 7.03 7.02 7.02 7.01 7.01 7.01 7.00 6.99 6.99 6.99 6.99
5.32 CD2Cl2 5.32 CD2Cl2 5.32 CD2Cl2
2.66 2.64 2.63 2.62 2.61 2.60 2.59 2.57 2.51 2.50 2.48 2.47 2.46 2.46 2.44 2.44 2.43 2.43 2.42 2.42 2.41 2.40 2.39 2.39 2.38 1.69
1.53 H2O
F
O
O
6i
1.00
2.96
1.04
3.19
3.17
f1 (ppm)

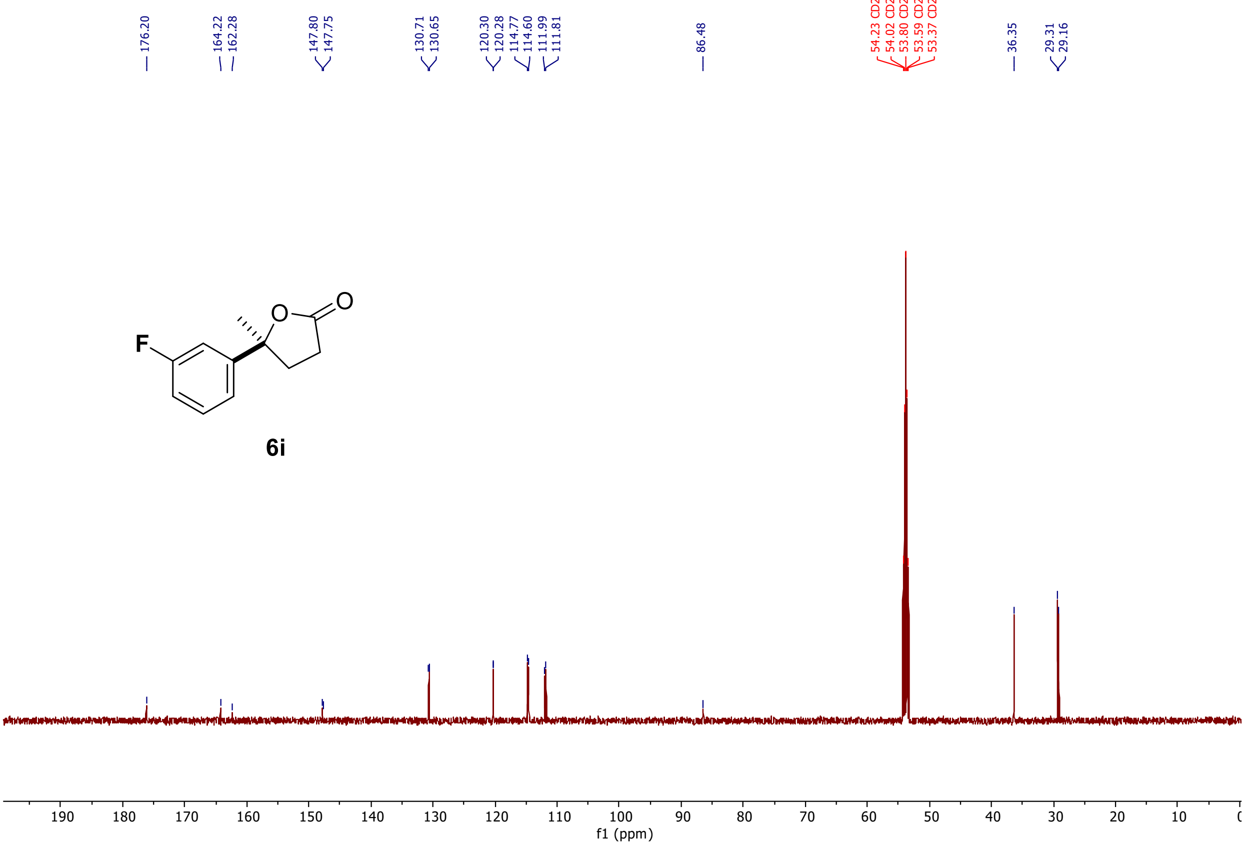

176.20
164.22 162.28
147.80 147.75
130.71 130.65
120.30 120.28 114.77 114.60 111.99 111.81
86.48
54.23 CD2Cl2 54.02 CD2Cl2 53.80 CD2Cl2 53.59 CD2Cl2 53.37 CD2Cl2
36.35
29.31 29.16
F
O
O
6i
f1 (ppm)

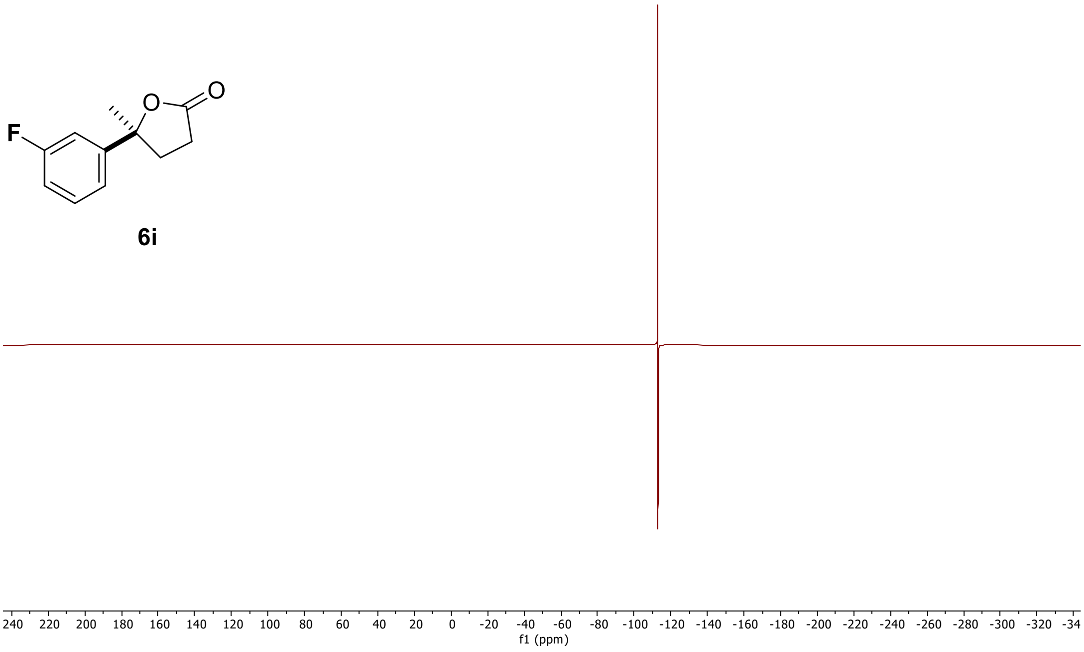
F
O
O
6i
240 220 200 180 160 140 120 100 80 60 40 20 0 -20 -40 -60 -80 -100 -120 -140 -160 -180 -200 -220 -240 -260 -280 -300 -320 -34
f1 (ppm)

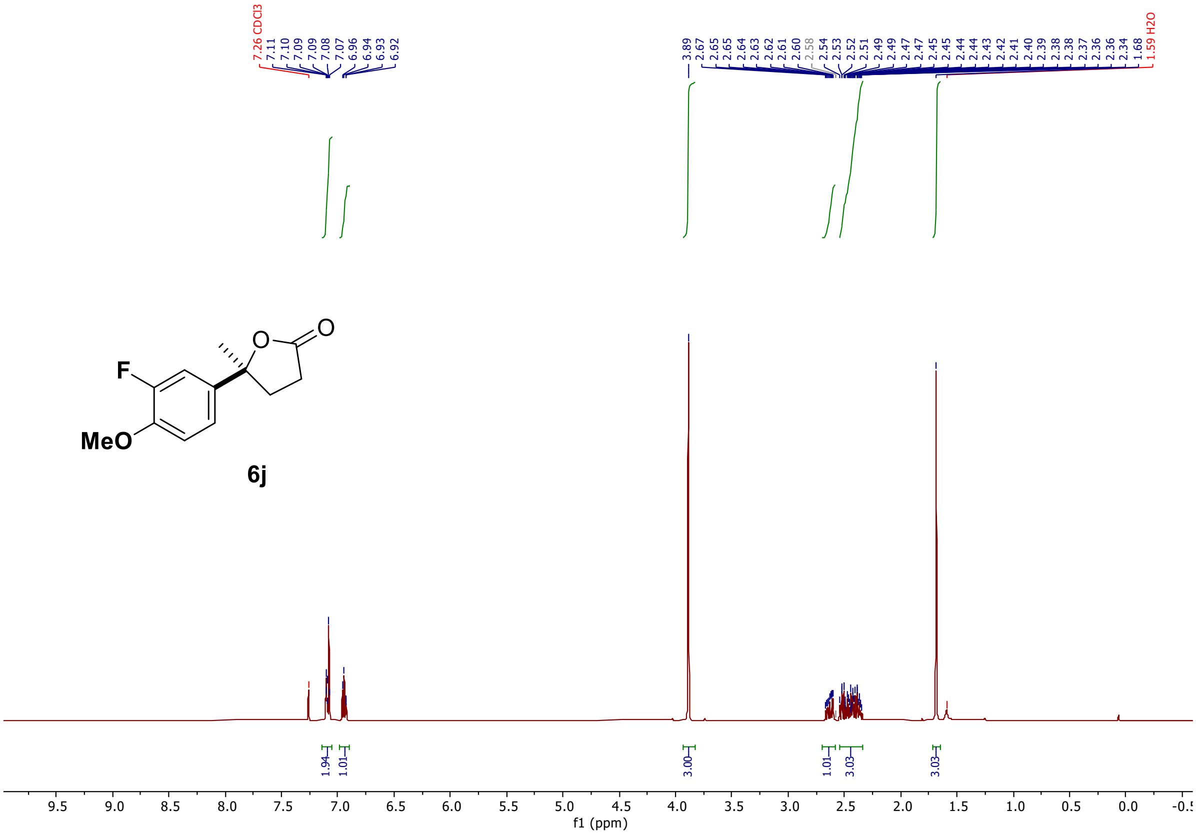
7.26 CDCl3
7.11
7.10
7.09
7.09
7.08
7.07
6.96
6.94
6.93
6.92
3.89
2.67
2.65
2.65
2.64
2.63
2.62
2.61
2.60
2.58
2.54
2.53
2.52
2.51
2.49
2.49
2.47
2.47
2.45
2.45
2.44
2.44
2.43
2.42
2.41
2.40
2.39
2.38
2.38
2.37
2.36
2.36
2.34
1.68
1.59 H2O
F
MeO
O
O
6j
1.94
1.01
3.00
1.01
3.03
3.03
9.5 9.0 8.5 8.0 7.5 7.0 6.5 6.0 5.5 5.0 4.5 4.0 3.5 3.0 2.5 2.0 1.5 1.0 0.5 0.0 -0.5
f1 (ppm)

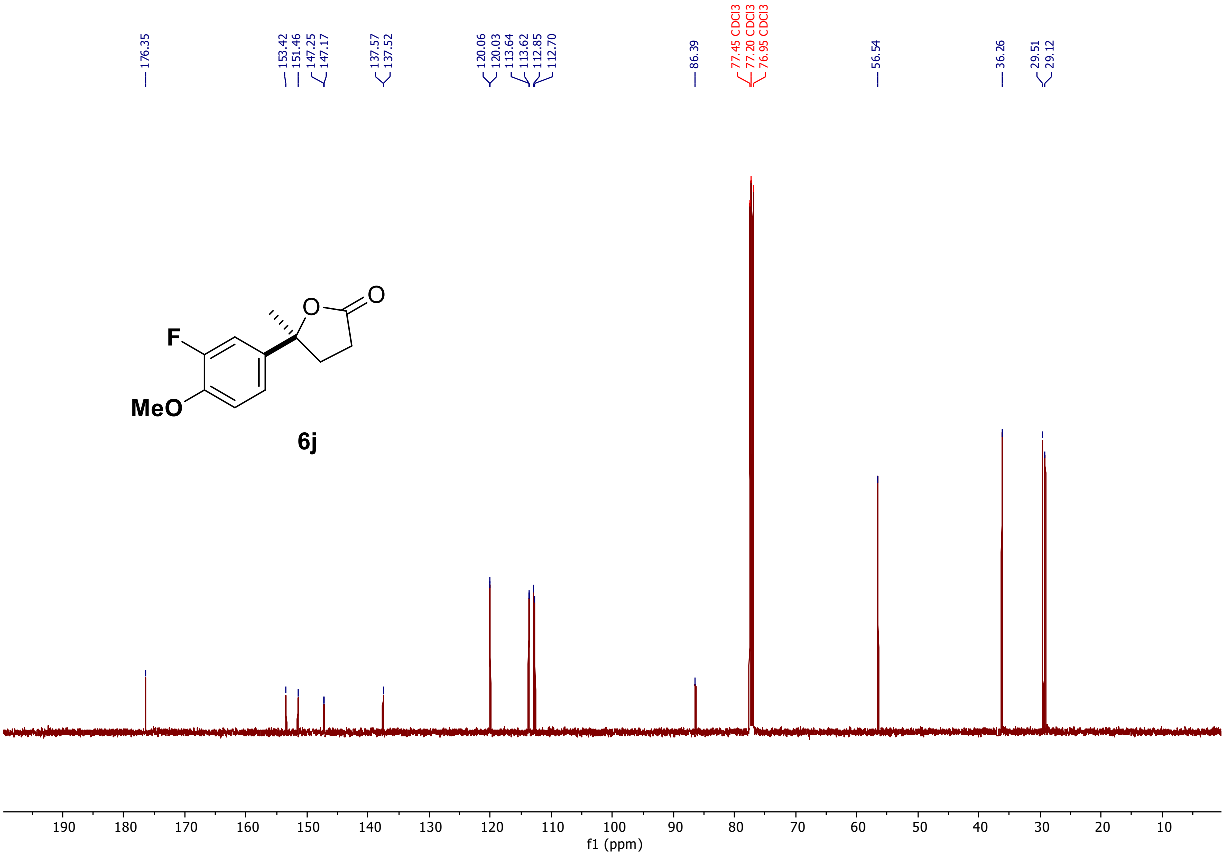
176.35
153.42
151.46
147.25
147.17
137.57
137.52
120.06
120.03
113.64
113.62
112.85
112.70
86.39
77.45 CDCl3
77.20 CDCl3
76.95 CDCl3
56.54
36.26
29.51
29.12
F
MeO
O
6j
190 180 170 160 150 140 130 120 110 100 90 80 70 60 50 40 30 20 10
f1 (ppm)

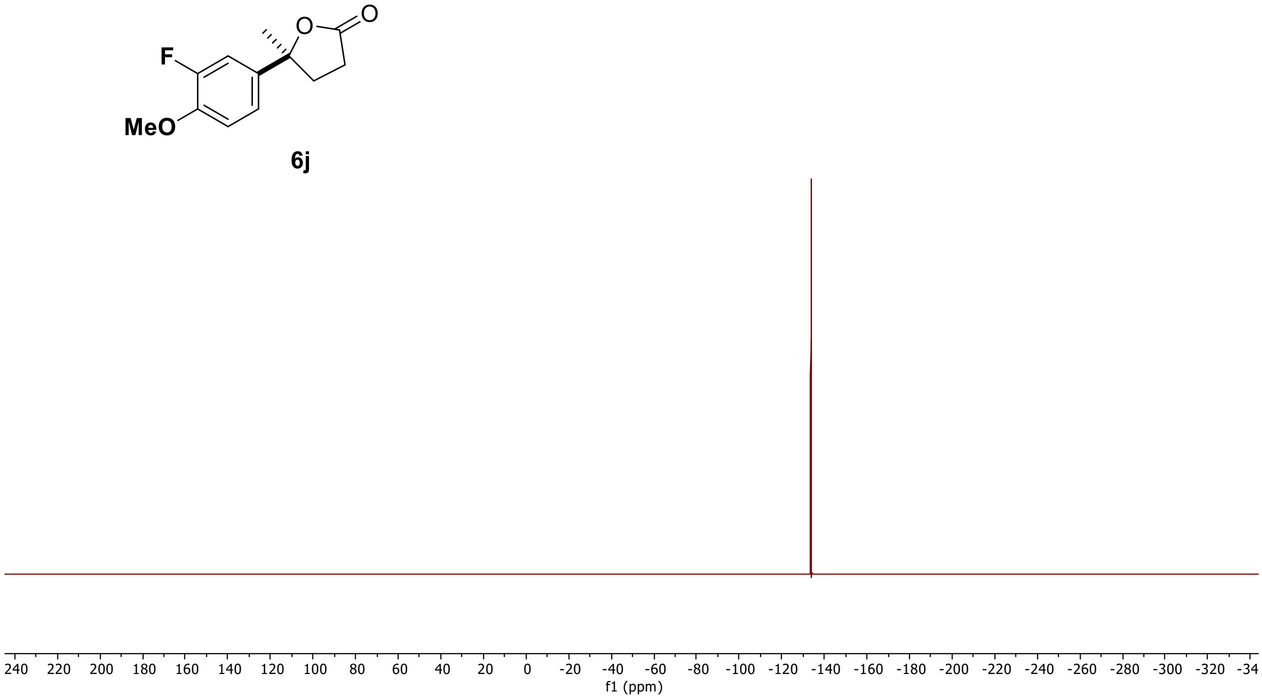
F
MeO
O
6j
240 220 200 180 160 140 120 100 80 60 40 20 0 -20 -40 -60 -80 -100 -120 -140 -160 -180 -200 -220 -240 -260 -280 -300 -320 -34
f1 (ppm)

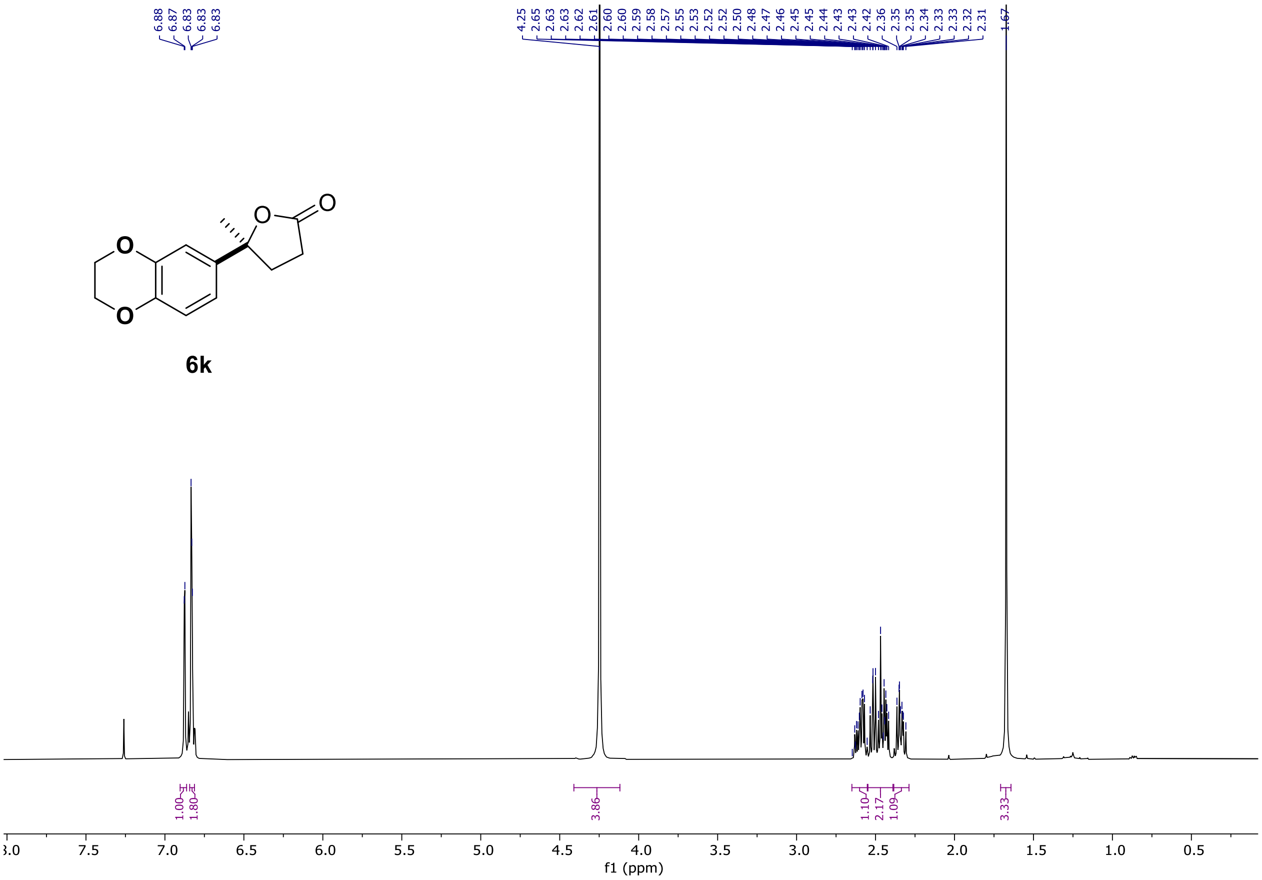
6.88
6.87
6.83
6.83
6.83
6.83
4.25
2.65
2.63
2.63
2.62
2.61
2.60
2.60
2.59
2.58
2.57
2.55
2.53
2.52
2.52
2.50
2.48
2.47
2.46
2.45
2.45
2.44
2.43
2.43
2.42
2.36
2.35
2.35
2.34
2.33
2.33
2.32
2.31
1.67
6k
1.00
1.80
3.86
1.10
2.17
1.09
3.33
f1 (ppm)

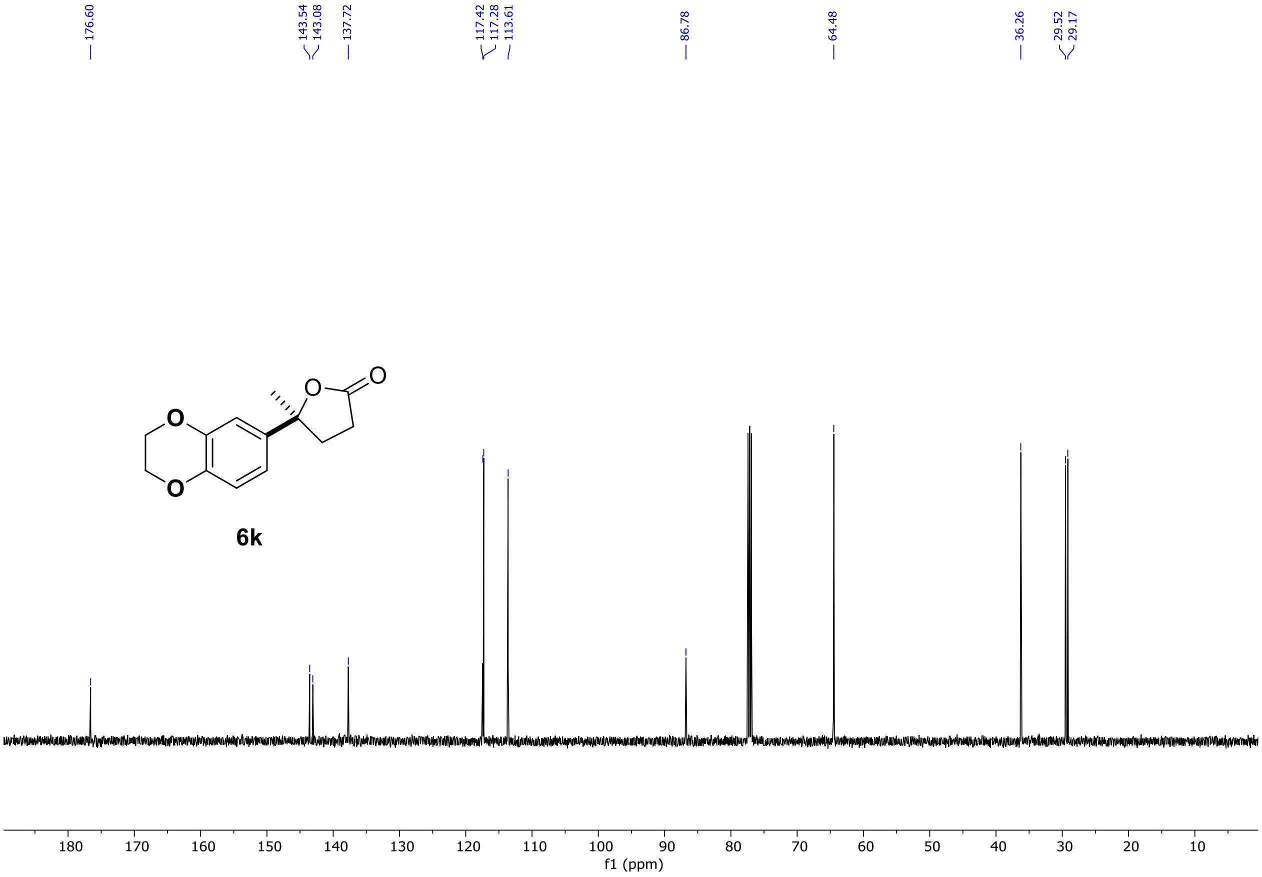
176.60
143.54
143.08
137.72
117.42
117.28
113.61
86.78
64.48
36.26
29.52
29.17
6k
f1 (ppm)

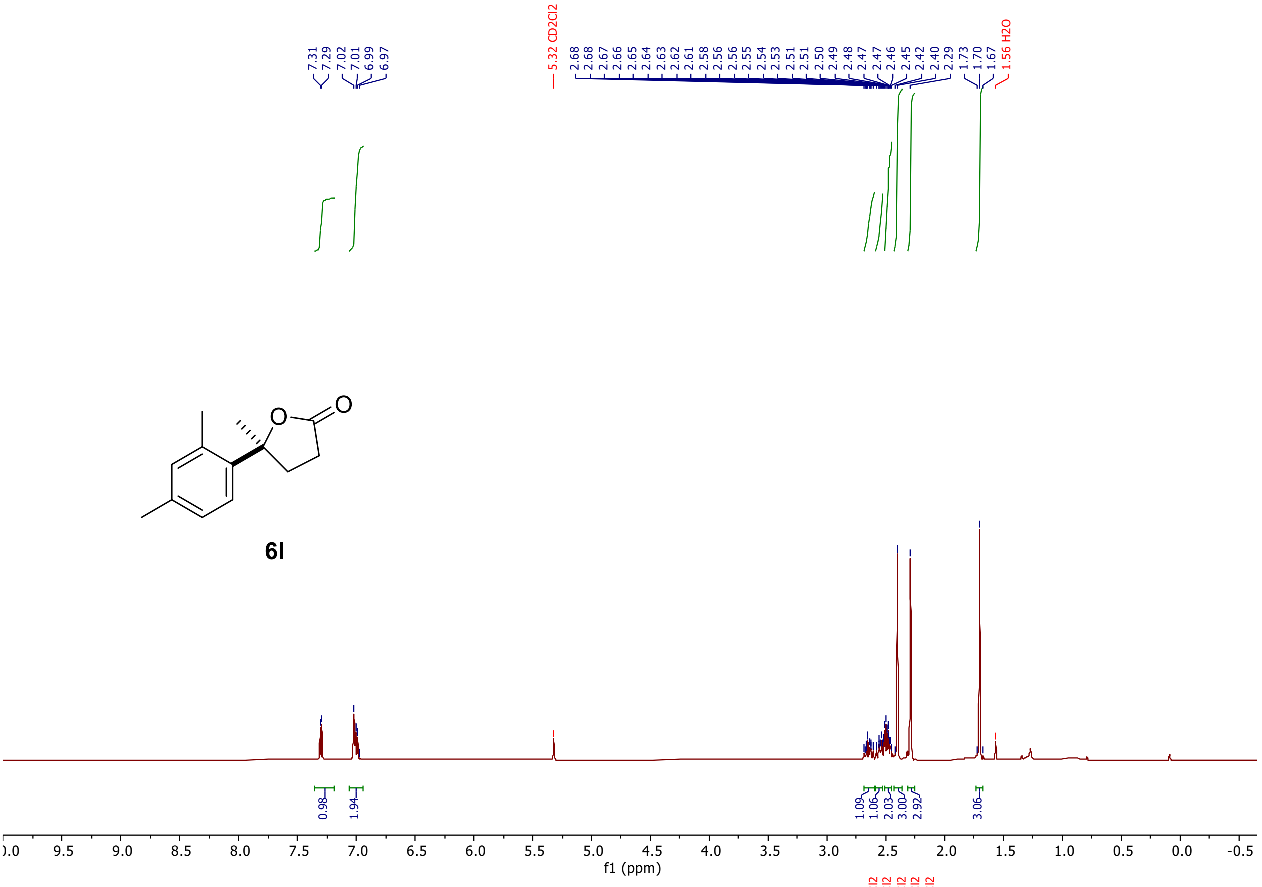
7.31
7.29
7.02
7.01
6.99
6.97
5.32 CD2Cl2
2.68
2.68
2.67
2.66
2.65
2.64
2.63
2.62
2.61
2.58
2.56
2.56
2.55
2.54
2.53
2.51
2.51
2.50
2.49
2.48
2.47
2.47
2.46
2.45
2.42
2.40
2.29
1.73
1.70
1.67
1.56 H2O
6l
0.98
1.94
1.09
1.06
2.03
3.00
2.92
3.06
f1 (ppm)

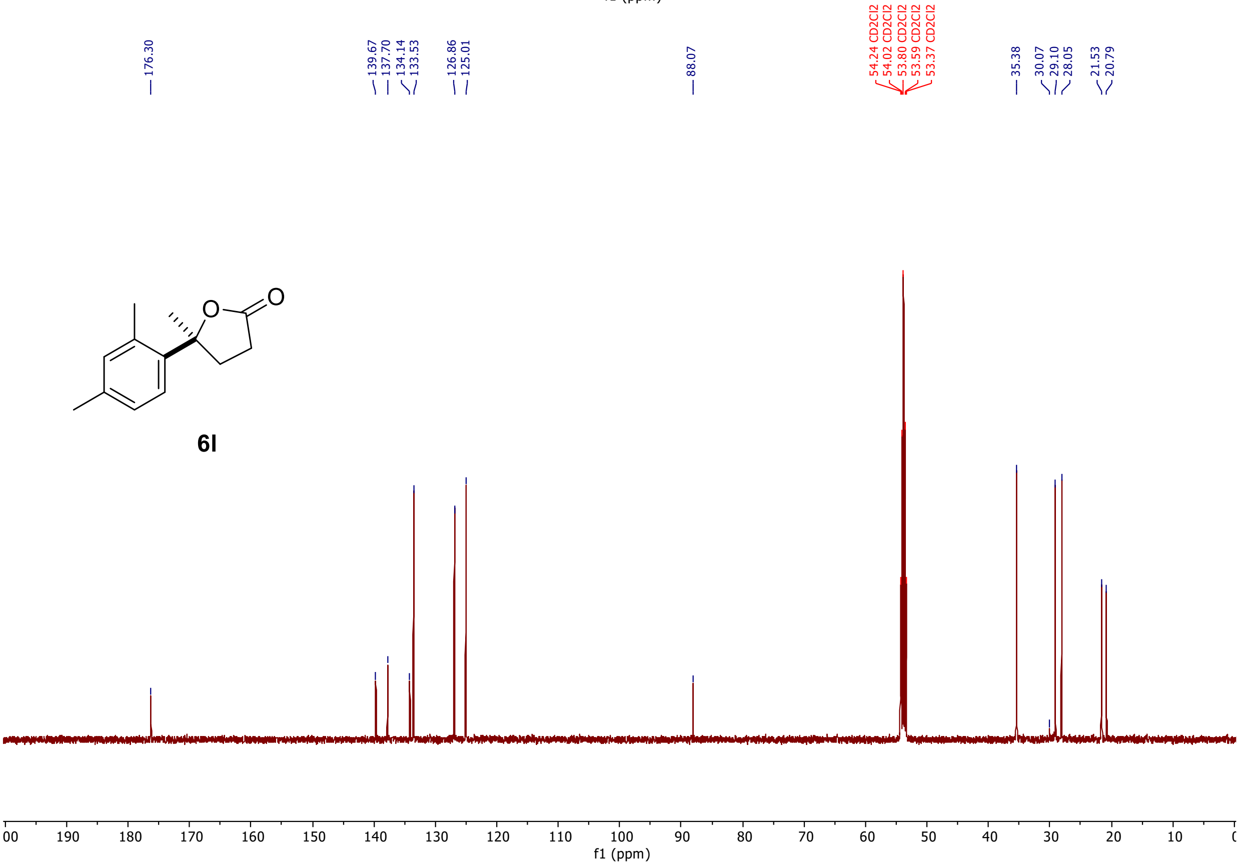
176.30
139.67
137.70
134.14
133.53
126.86
125.01
88.07
54.24 CD2Cl2
54.02 CD2Cl2
53.80 CD2Cl2
53.59 CD2Cl2
53.37 CD2Cl2
35.38
30.07
29.10
28.05
21.53
20.79
6l
f1 (ppm)

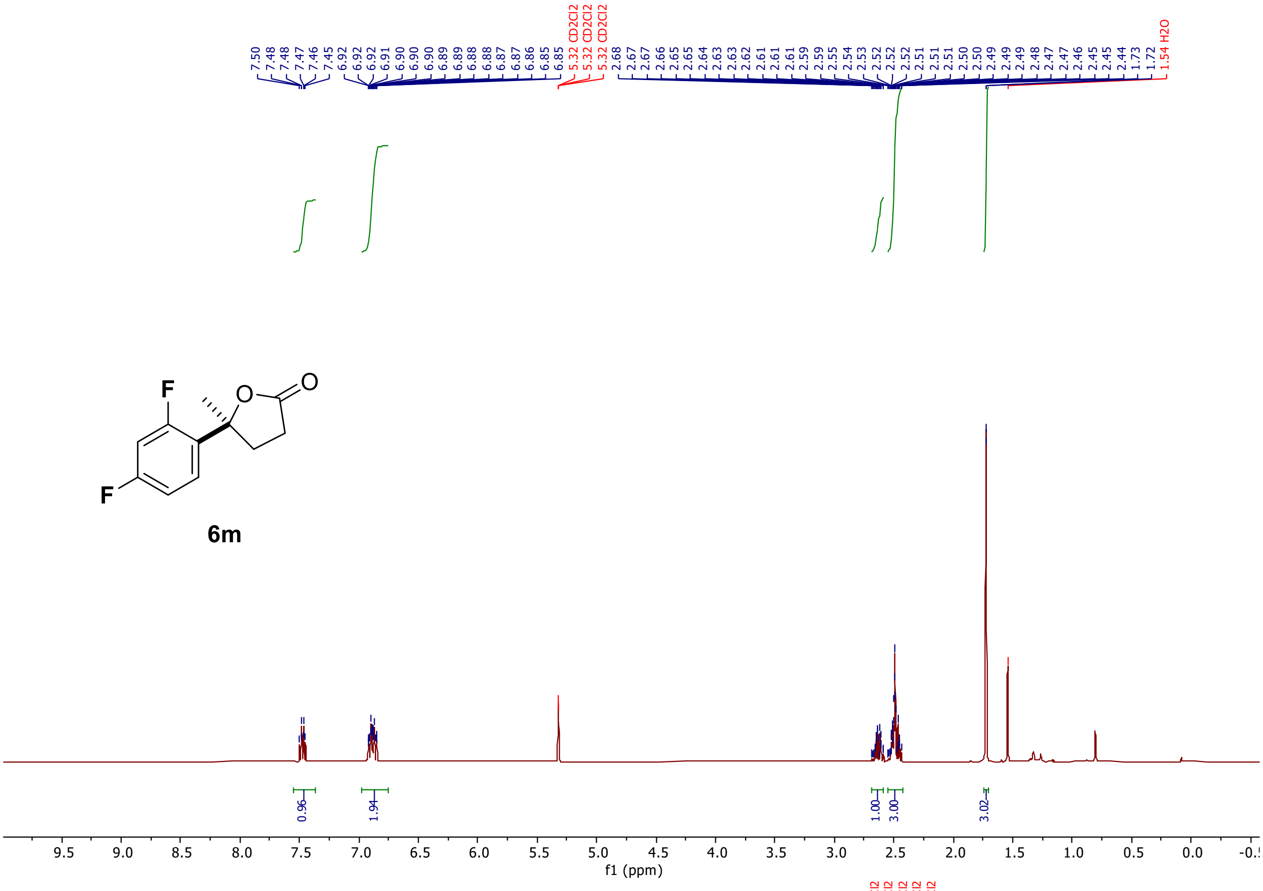
7.50
7.48
7.48
7.47
7.46
7.45
6.92
6.92
6.92
6.91
6.90
6.90
6.90
6.89
6.89
6.88
6.88
6.87
6.87
6.86
6.85
6.85
5.32 CD2Cl2
5.32 CD2Cl2
5.32 CD2Cl2
2.68
2.67
2.67
2.66
2.65
2.65
2.64
2.63
2.63
2.62
2.61
2.61
2.61
2.59
2.59
2.55
2.55
2.54
2.53
2.52
2.52
2.52
2.51
2.51
2.51
2.50
2.50
2.49
2.49
2.49
2.48
2.47
2.47
2.46
2.45
2.45
2.44
1.73
1.72
1.54 H2O
F
O
O
F
6m
0.96
1.94
1.00
3.00
3.02
9.5
9.0
8.5
8.0
7.5
7.0
6.5
6.0
5.5
5.0
4.5
4.0
3.5
3.0
2.5
2.0
1.5
1.0
0.5
0.0
f1 (ppm)

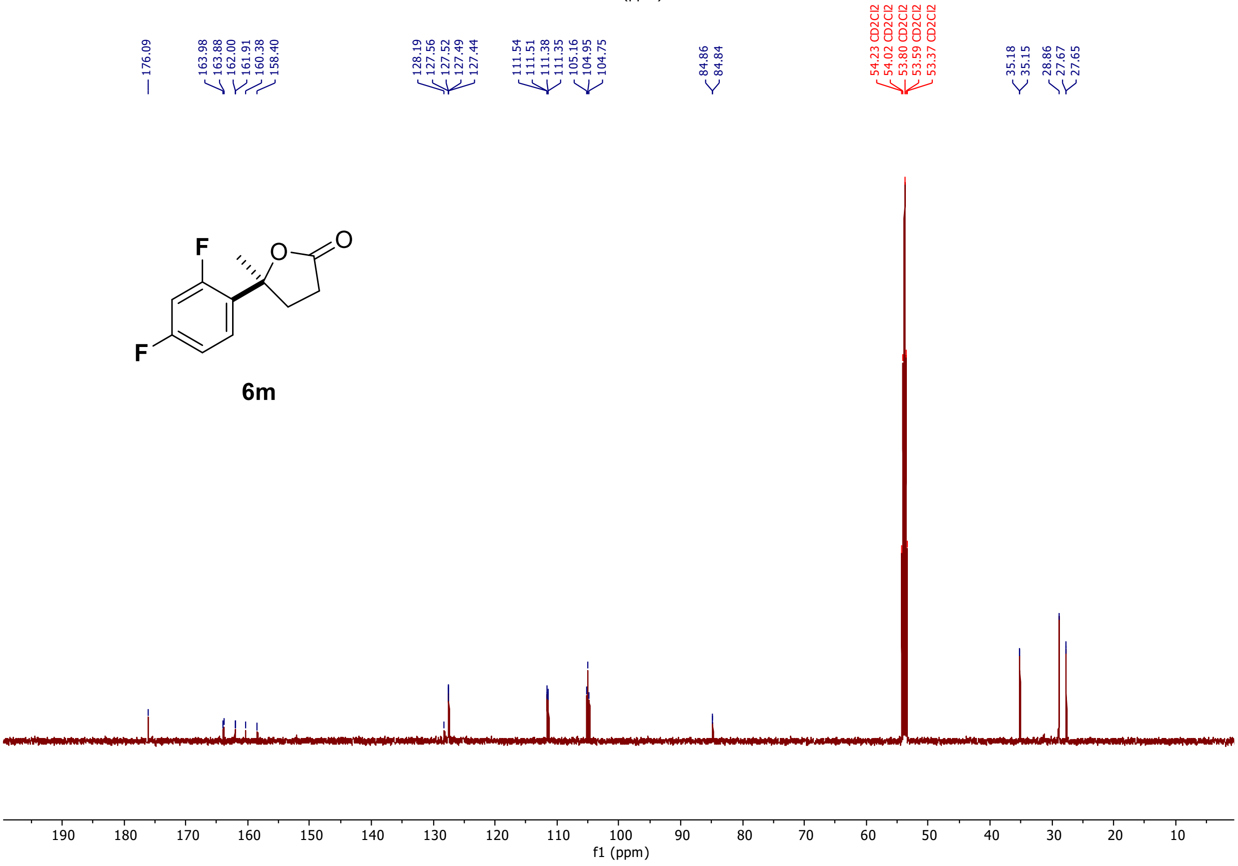
176.09
163.98
163.88
162.00
161.91
160.38
158.40
128.19
127.56
127.52
127.49
127.44
111.54
111.51
111.38
111.35
105.16
104.95
104.75
84.86
84.84
54.23 CD2Cl2
54.02 CD2Cl2
53.80 CD2Cl2
53.59 CD2Cl2
53.37 CD2Cl2
35.18
35.15
28.86
27.67
27.65
F
O
O
F
6m
190
180
170
160
150
140
130
120
110
100
90
80
70
60
50
40
30
20
10
f1 (ppm)

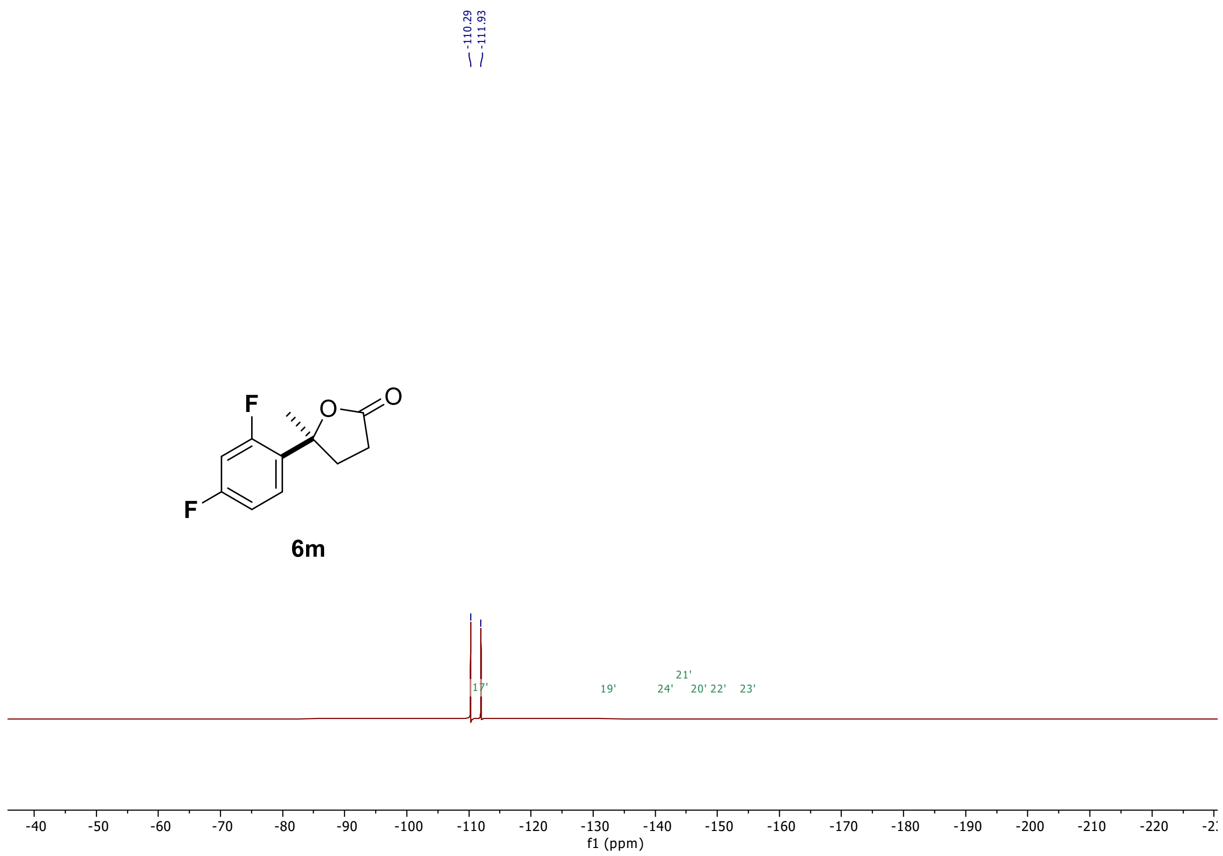
-110.29
-111.93
F
O
O
F
6m
17'
19'
24'
21'
20' 22'
23'
-40 -50 -60 -70 -80 -90 -100 -110 -120 -130 -140 -150 -160 -170 -180 -190 -200 -210 -220
f1 (ppm)

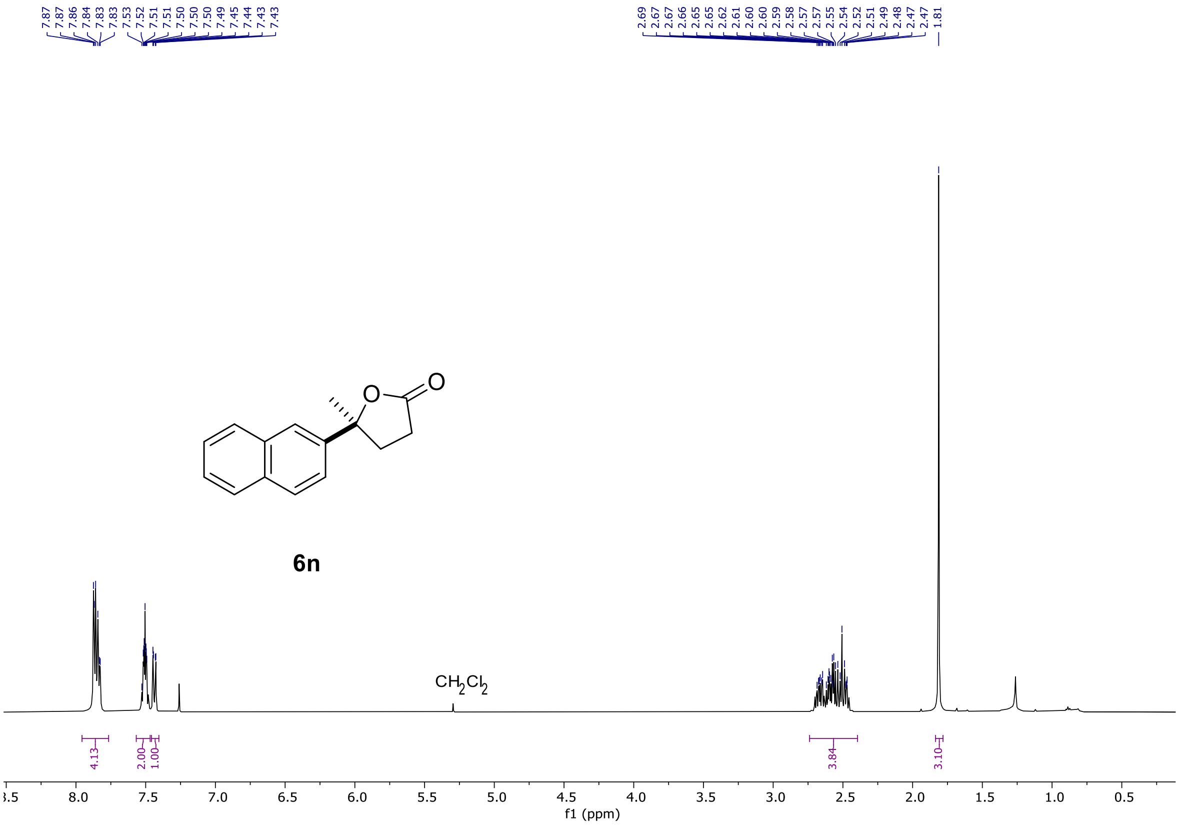
7.87
7.87
7.86
7.84
7.83
7.83
7.53
7.52
7.51
7.51
7.50
7.50
7.50
7.49
7.45
7.44
7.43
7.43
2.69
2.67
2.67
2.66
2.65
2.65
2.62
2.61
2.60
2.59
2.58
2.57
2.57
2.55
2.54
2.52
2.51
2.49
2.48
2.47
2.47
1.81
6n
CH_2Cl_2
4.13
2.00
1.00
3.84
3.10
f1 (ppm)

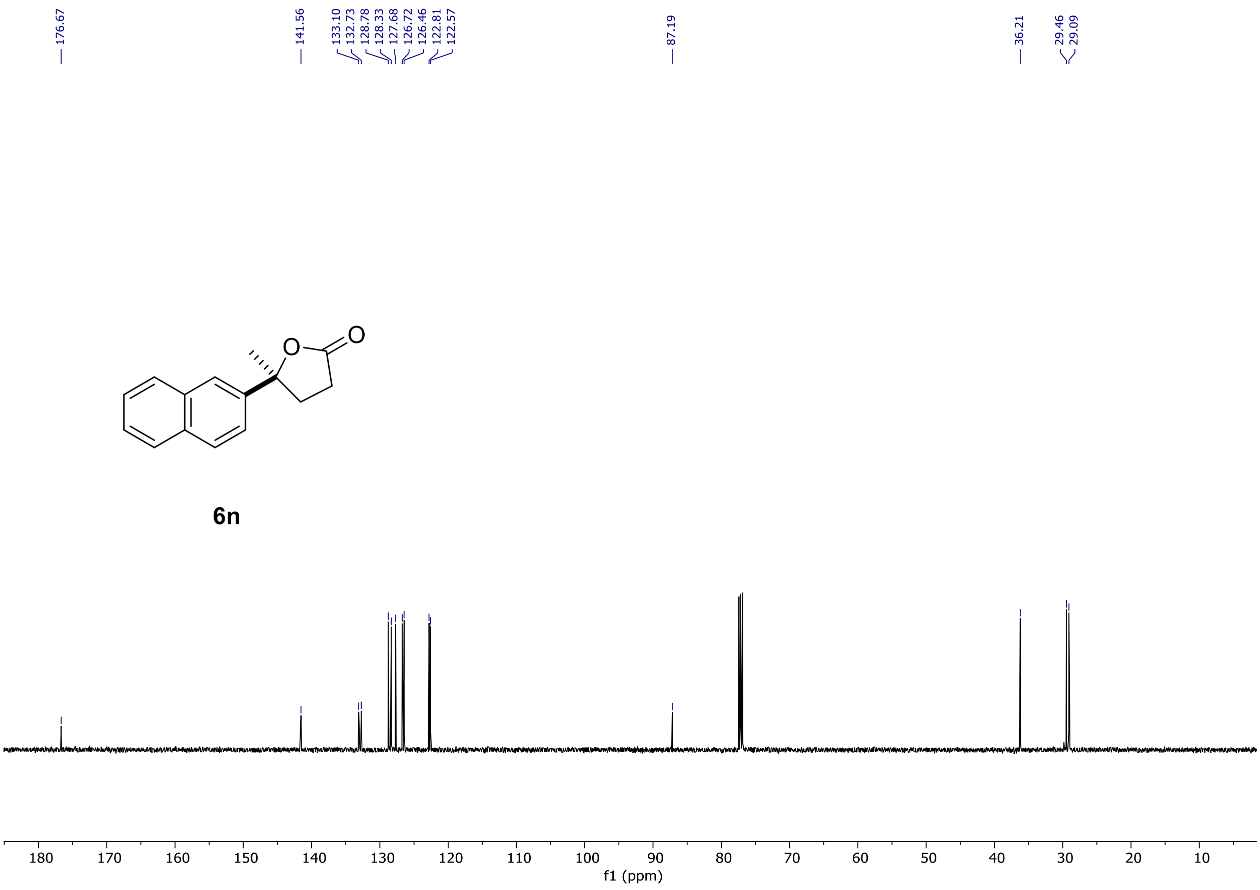
176.67
141.56
133.10
132.73
128.78
128.33
127.68
126.72
126.46
122.81
122.57
87.19
36.21
29.46
29.09
6n
f1 (ppm)

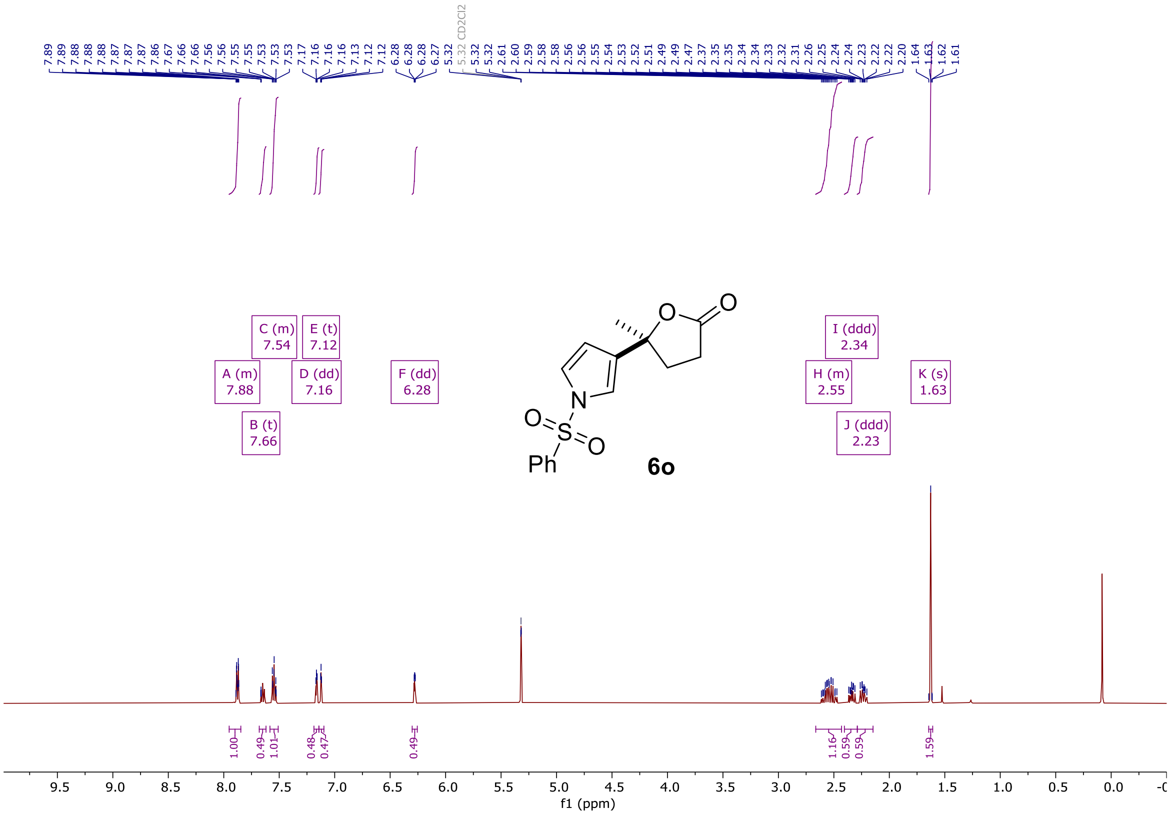
7.89 7.89 7.88 7.88 7.87 7.87 7.87 7.86 7.67 7.66 7.66 7.56 7.56 7.55 7.55 7.53 7.53 7.53 7.17 7.16 7.16 7.16 7.13 7.12 7.12 6.28 6.28 6.28 6.27 5.32 CD2Cl2 5.32 5.32 2.61 2.60 2.59 2.58 2.58 2.56 2.55 2.54 2.53 2.52 2.51 2.49 2.49 2.47 2.37 2.35 2.35 2.34 2.34 2.33 2.32 2.31 2.26 2.25 2.24 2.24 2.23 2.23 2.22 2.22 2.20 1.64 1.63 1.62 1.61
C (m) 7.54
E (t) 7.12
A (m) 7.88
D (dd) 7.16
B (t) 7.66
F (dd) 6.28
I (ddd) 2.34
H (m) 2.55
J (ddd) 2.23
K (s) 1.63
O
N
O
S
O
Ph
6o
1.00 0.49 1.01 0.48 0.47 0.49 1.16 0.59 0.59 1.59
9.5 9.0 8.5 8.0 7.5 7.0 6.5 6.0 5.5 5.0 4.5 4.0 3.5 3.0 2.5 2.0 1.5 1.0 0.5 0.0
f1 (ppm)

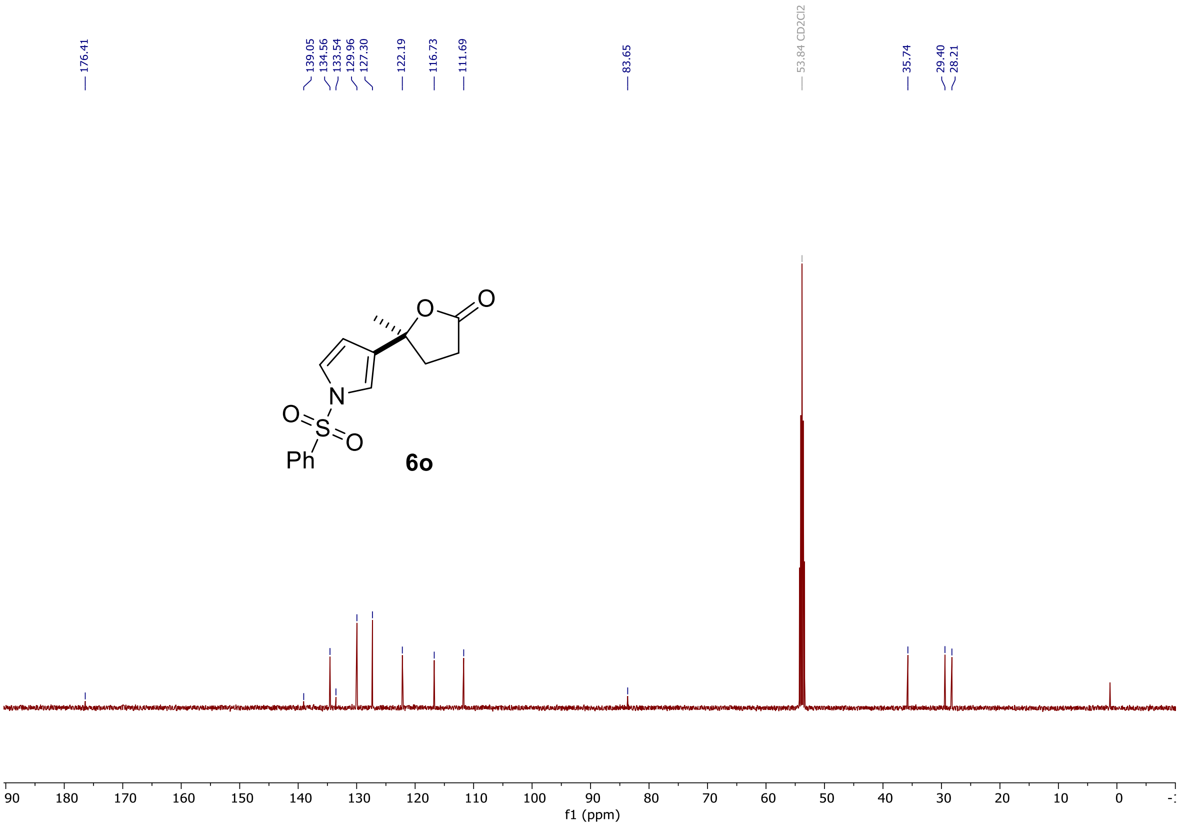
176.41
139.05 134.56 133.54 129.96 127.30
122.19
116.73
111.69
83.65
53.84 CD2Cl2
35.74
29.40 28.21
O
N
O
S
O
Ph
6o
90 180 170 160 150 140 130 120 110 100 90 80 70 60 50 40 30 20 10 0
f1 (ppm)

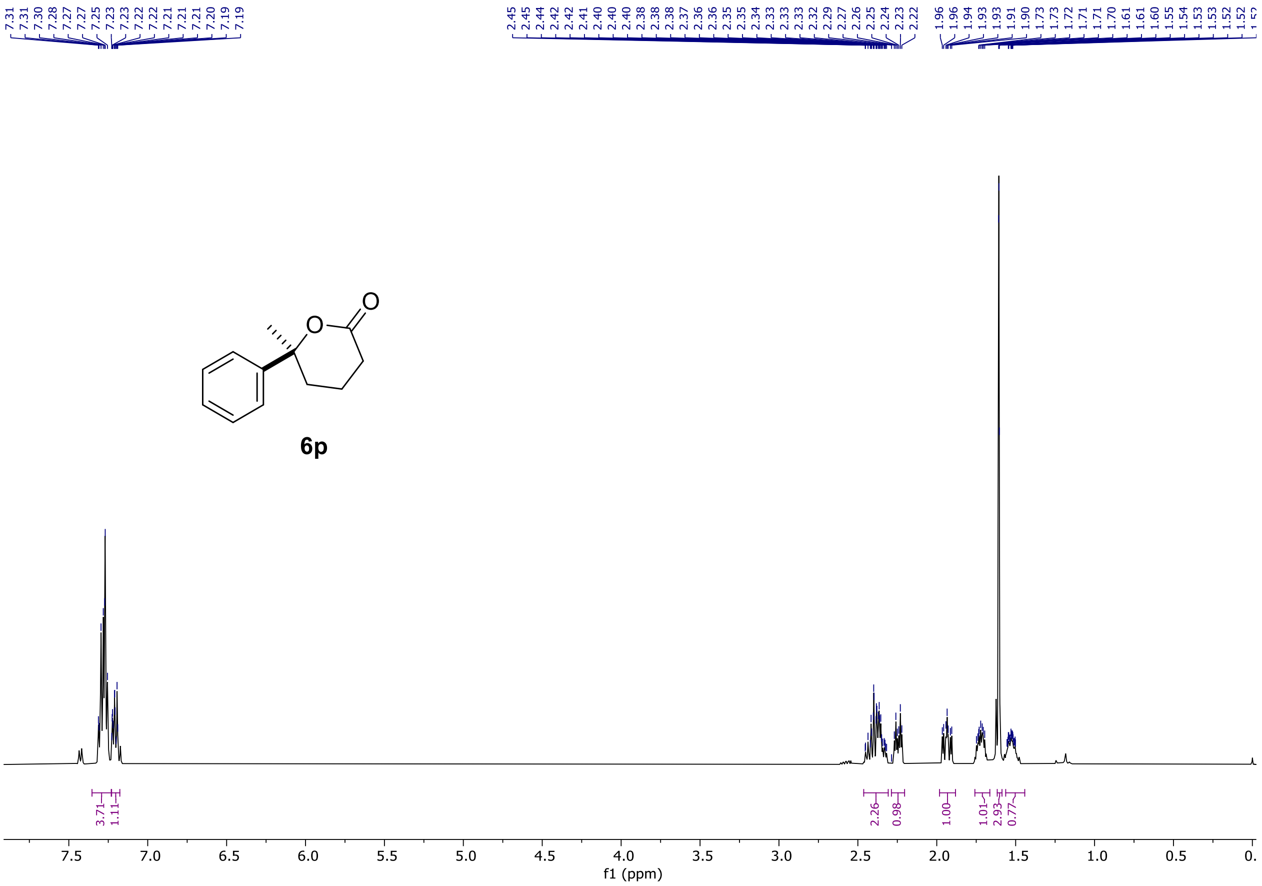
6p
f1 (ppm)

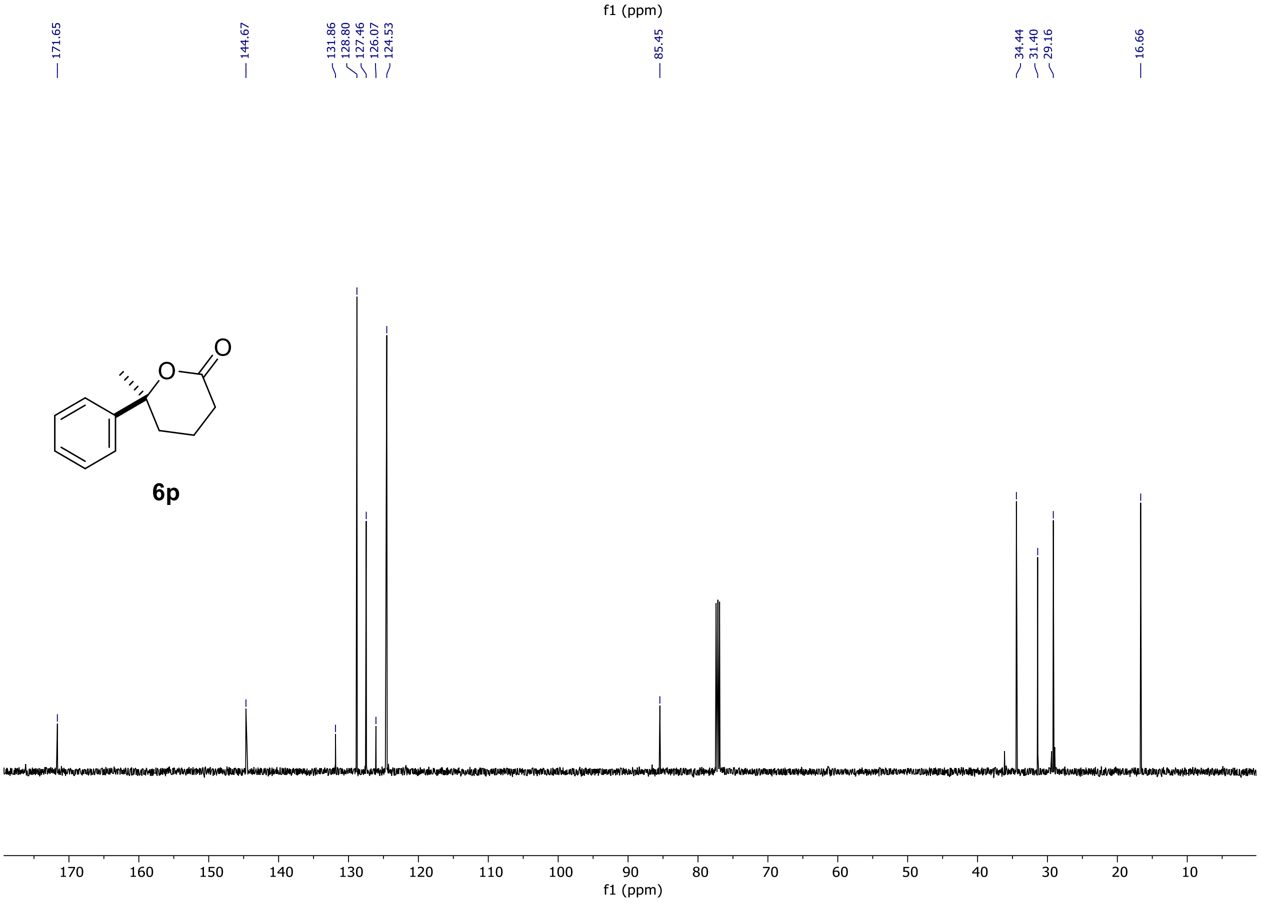
171.65
144.67
131.86
128.80
127.46
126.07
124.53
85.45
34.44
31.40
29.16
16.66
6p
f1 (ppm)

^{1}H NMR spectra (500 MHz, $CDCl_3$)

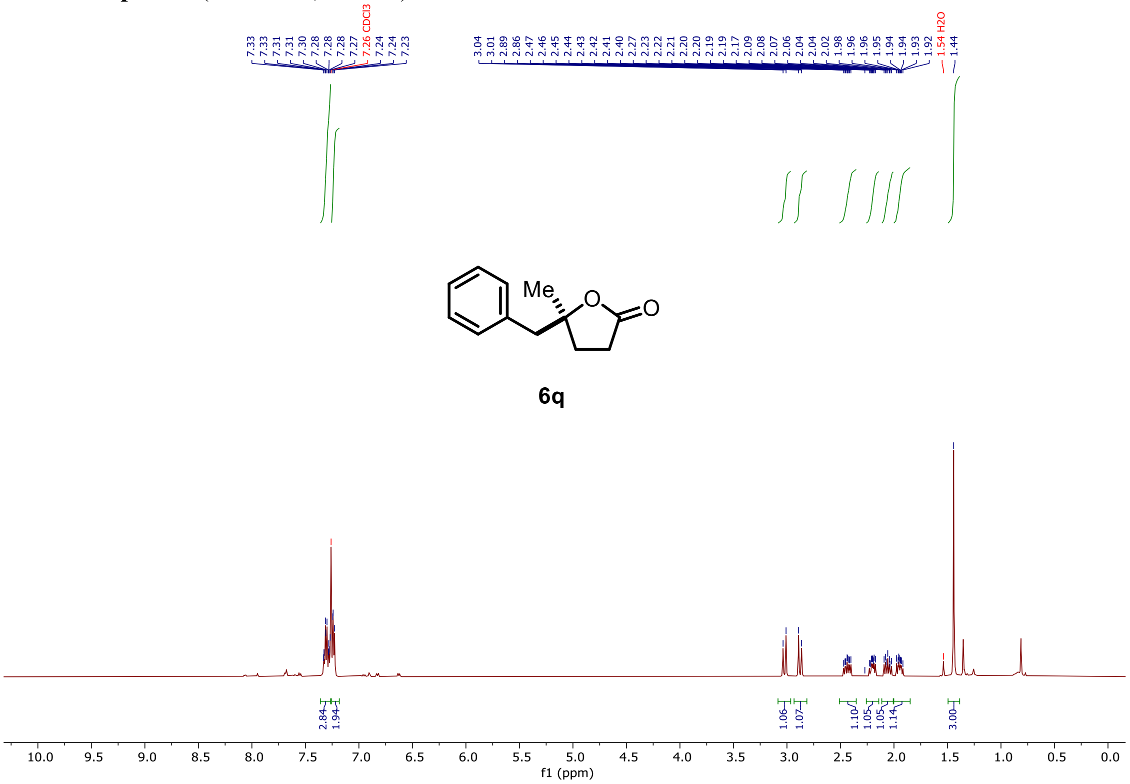

6q

^{13}C NMR spectra (125 MHz, $CDCl_3$)

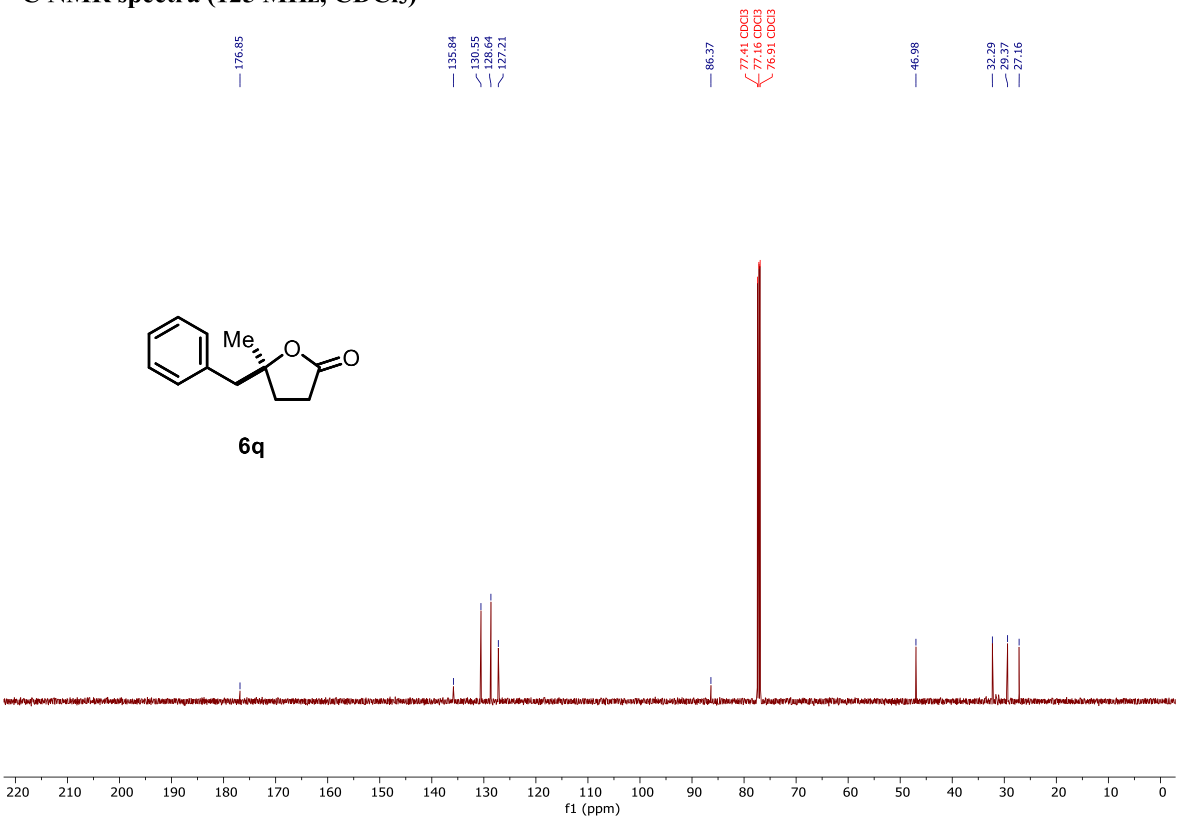

6q

^{1}H NMR spectra (500 MHz, $CDCl_3$)

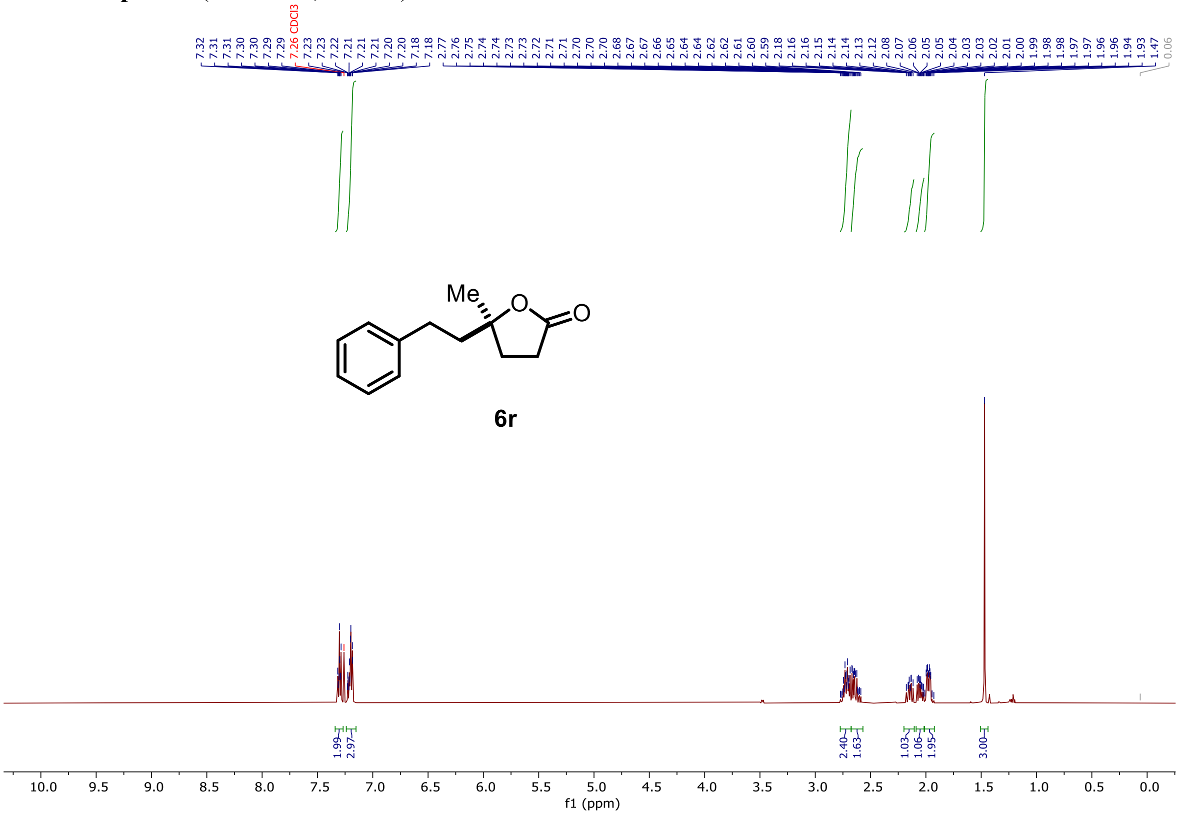

^{13}C NMR spectra (125 MHz, $CDCl_3$)

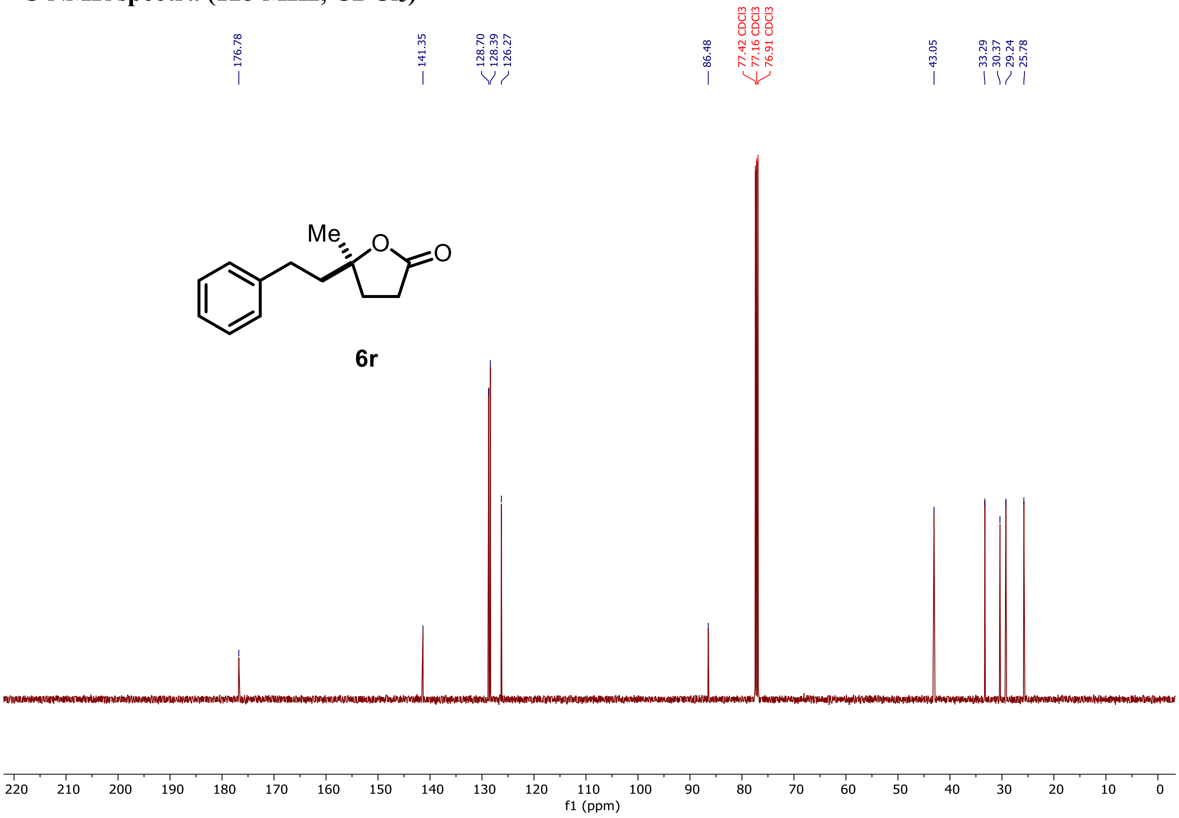

^{1}H NMR Spectra (500 MHz, $CDCl_3$)

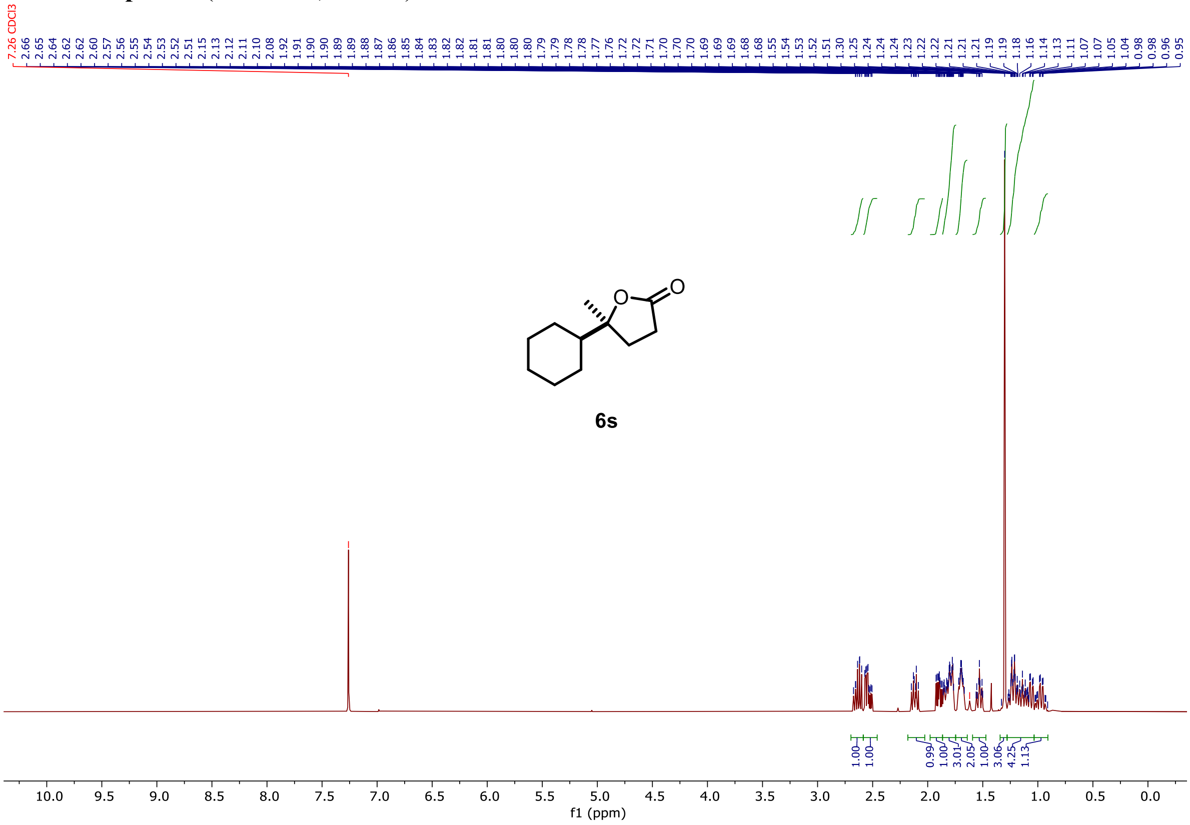

^{13}C NMR spectra (125 MHz, $CDCl_3$)

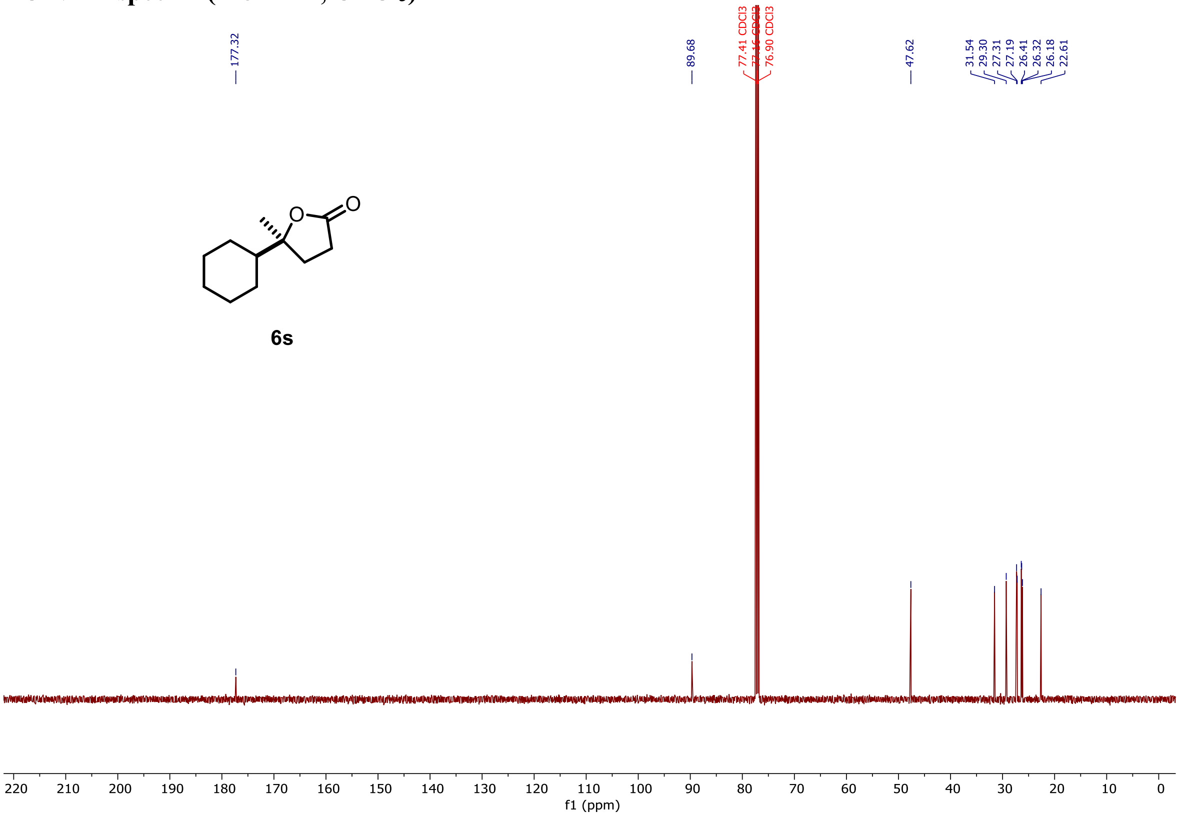

^{1}H NMR spectra (500 MHz, $CDCl_3$)

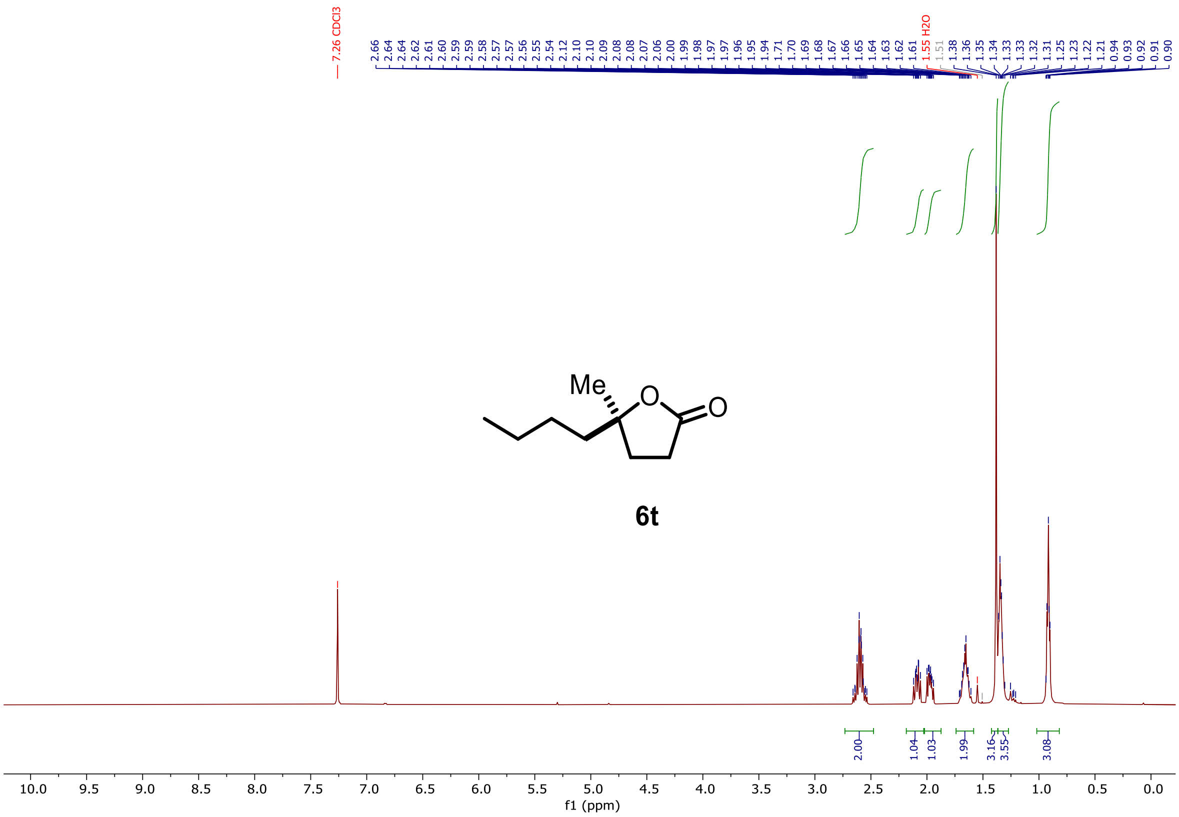

^{13}C NMR spectra (125 MHz, $CDCl_3$)

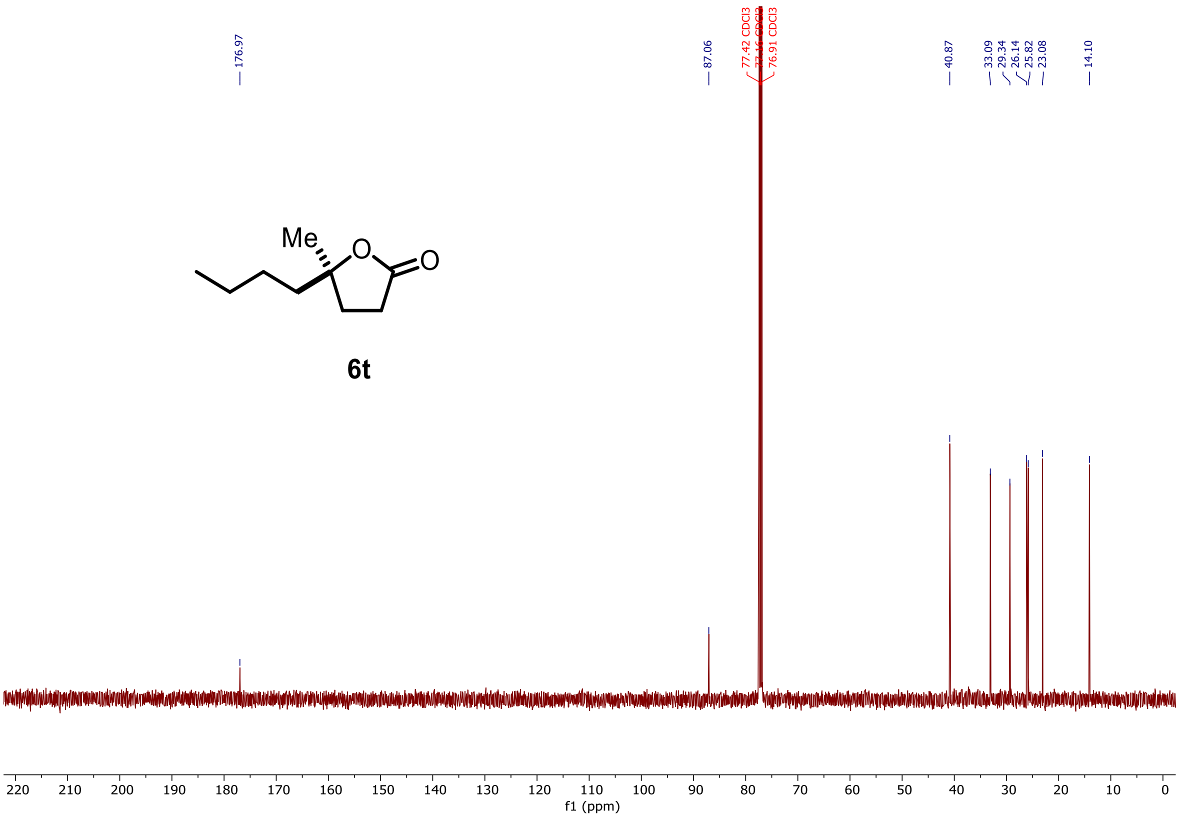

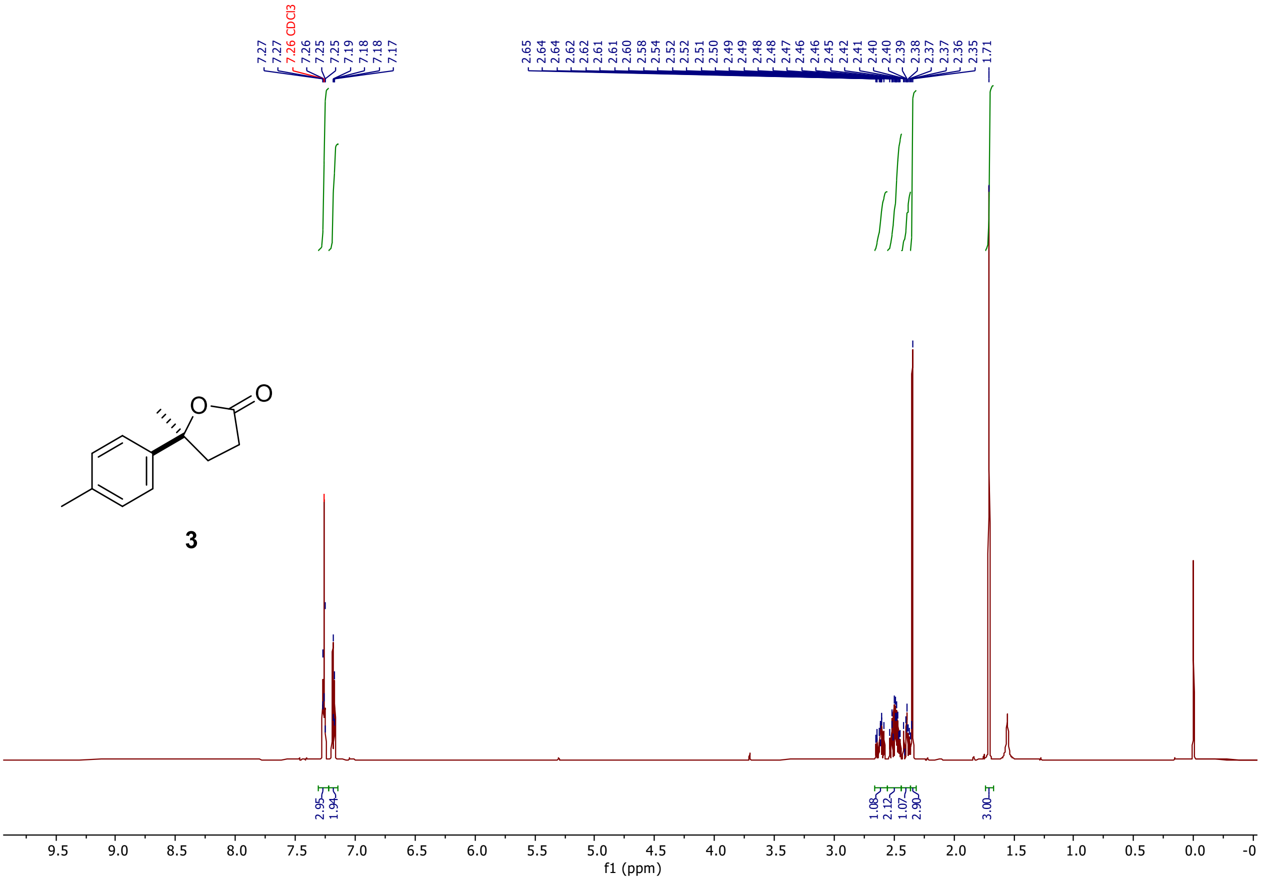
7.27 7.27 7.26 CDCl3 7.26 7.25 7.25 7.19 7.18 7.18 7.17
2.65 2.64 2.64 2.62 2.62 2.61 2.61 2.60 2.58 2.54 2.52 2.52 2.51 2.50 2.49 2.49 2.48 2.48 2.47 2.46 2.46 2.45 2.42 2.41 2.40 2.40 2.39 2.38 2.37 2.37 2.36 2.35 1.71
3
2.95 1.94 1.08 2.12 1.07 2.90 3.00
f1 (ppm)

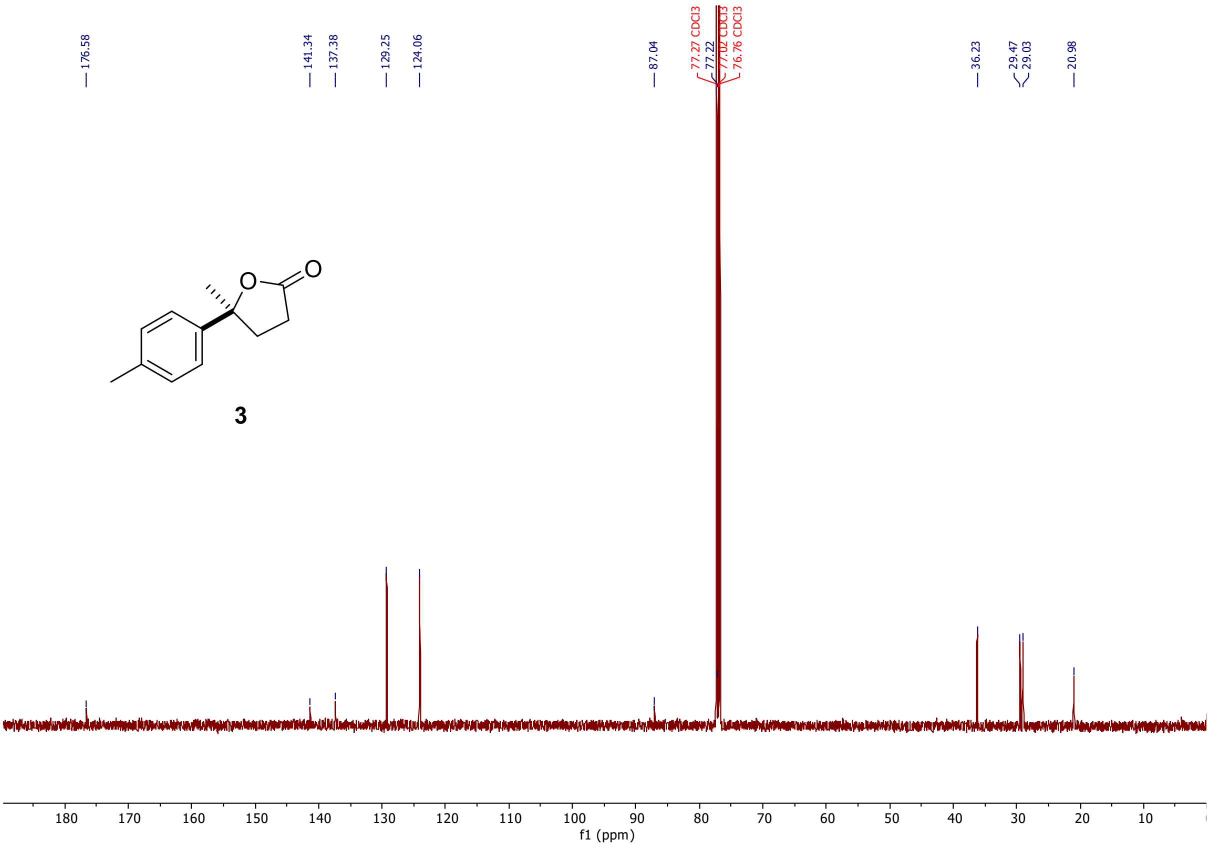
176.58 141.34 137.38 129.25 124.06 87.04 77.27 CDCl3 77.22 77.02 CDCl3 76.76 CDCl3 36.23 29.47 29.03 20.98
3
f1 (ppm)

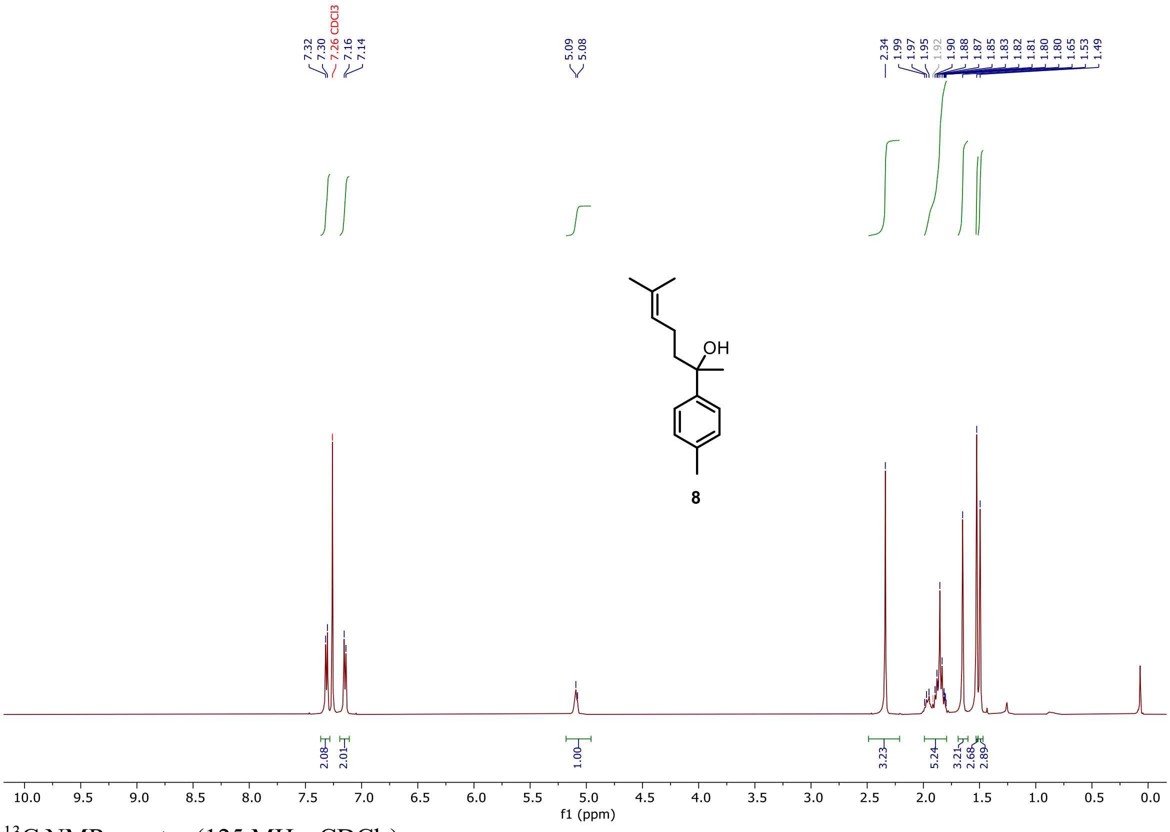

^{13}C NMR spectra (125 MHz, $CDCl_3$)

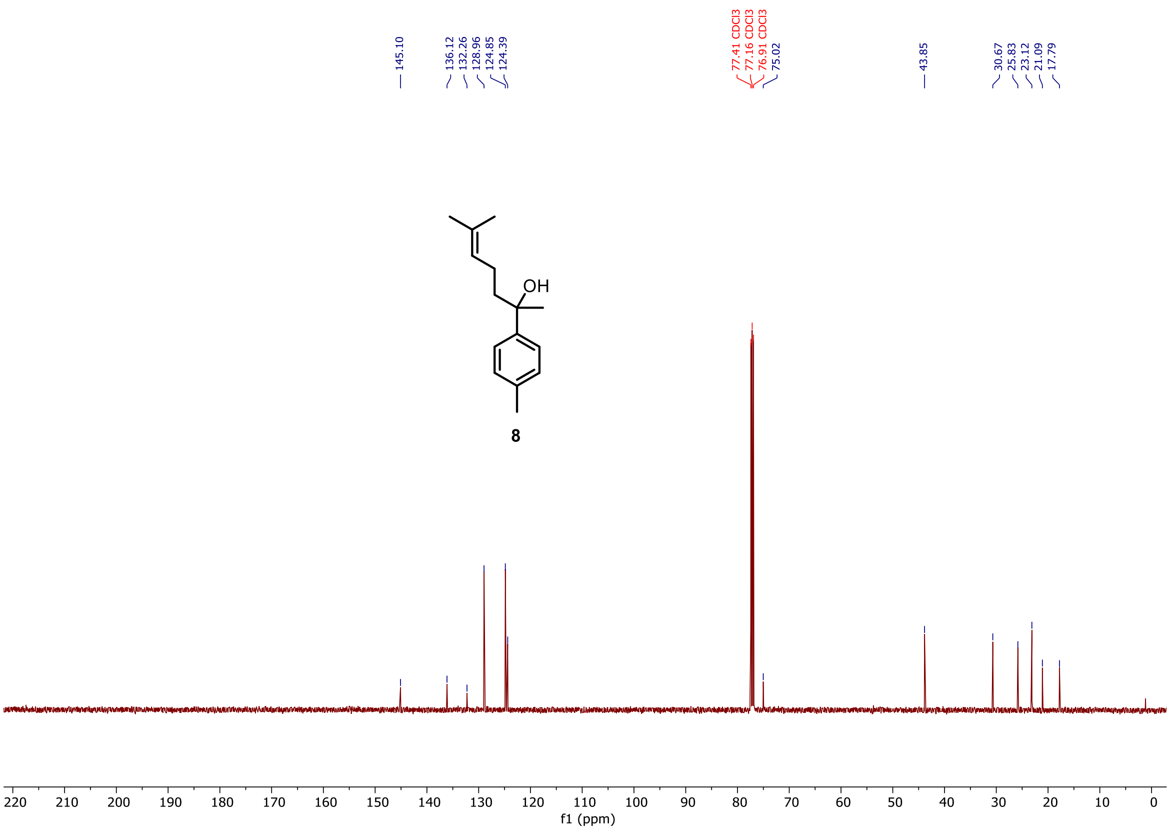

11. HPLC & GC Traces of Lactones

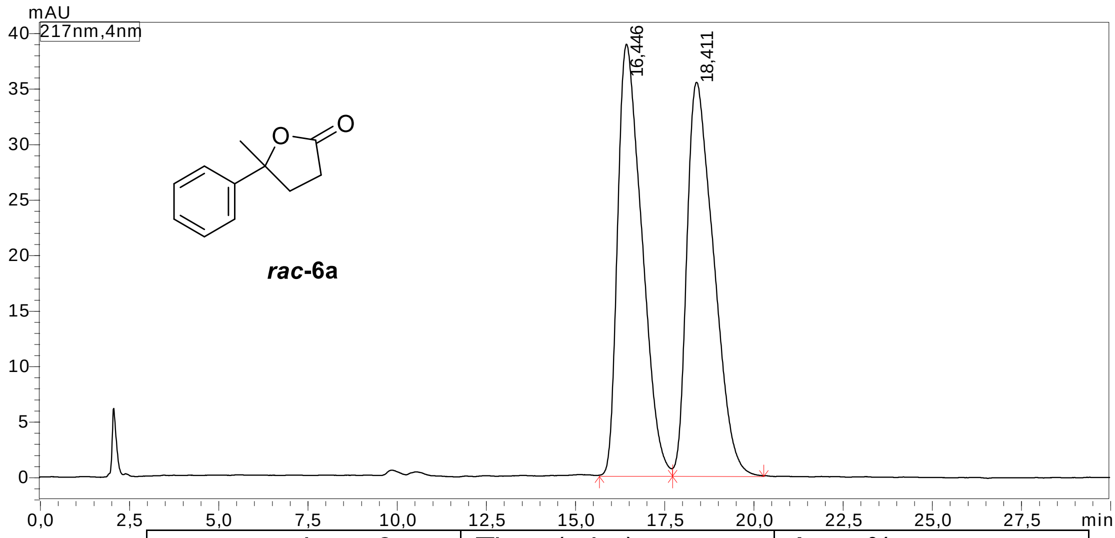

compound *rac*-6a	Time (min.)	Area %
1	16.4	49.75
2	18.4	50.25
Total		100

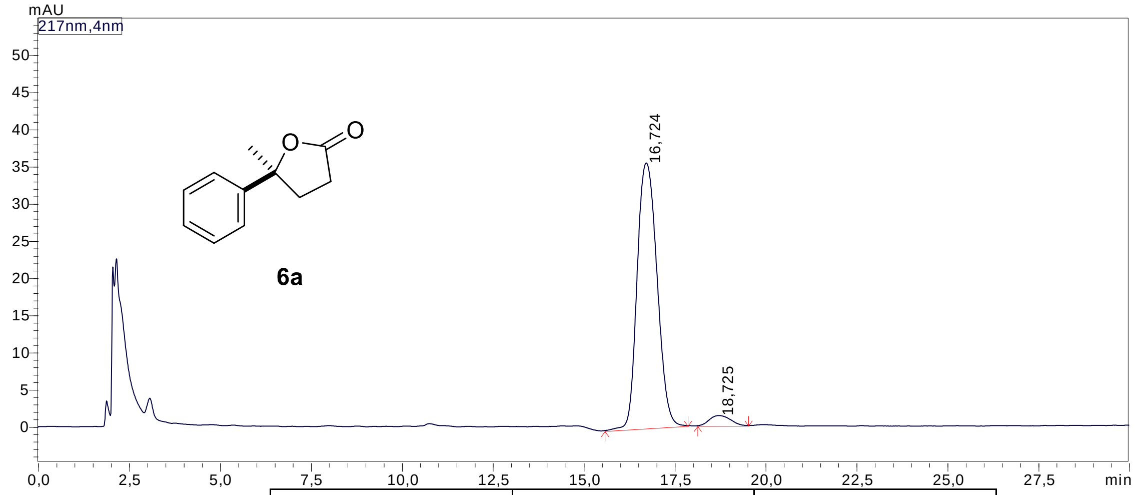

compound 6a	Time (min.)	Area %
1	16.7	96.531
2	18.7	3.469
Total		100

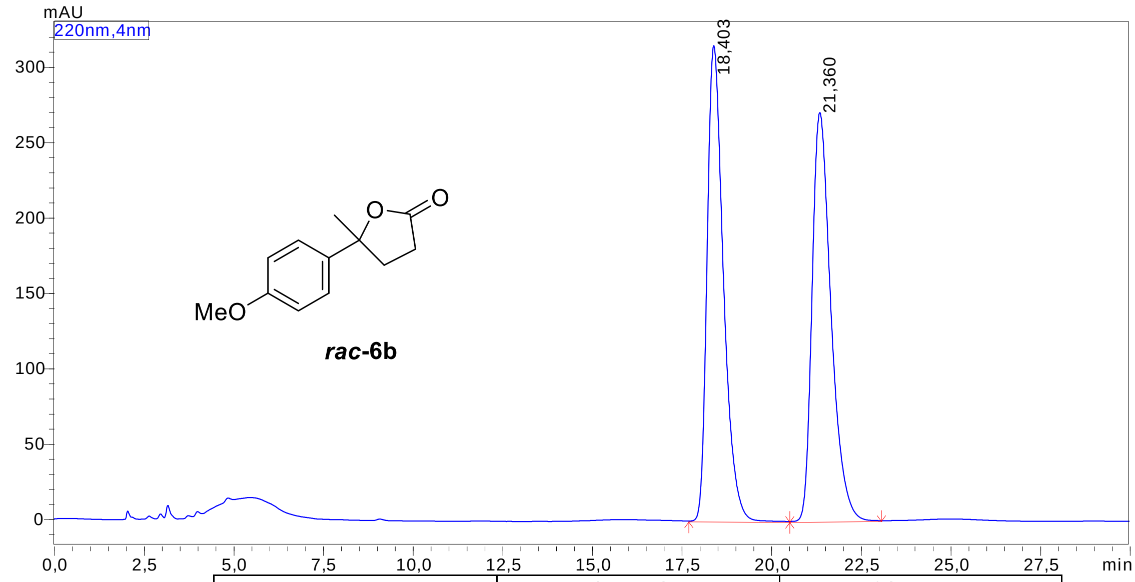

compound *rac*-6b	Time (min.)	Area %
1	18.4	50.03
2	21.3	49.97
Total		100

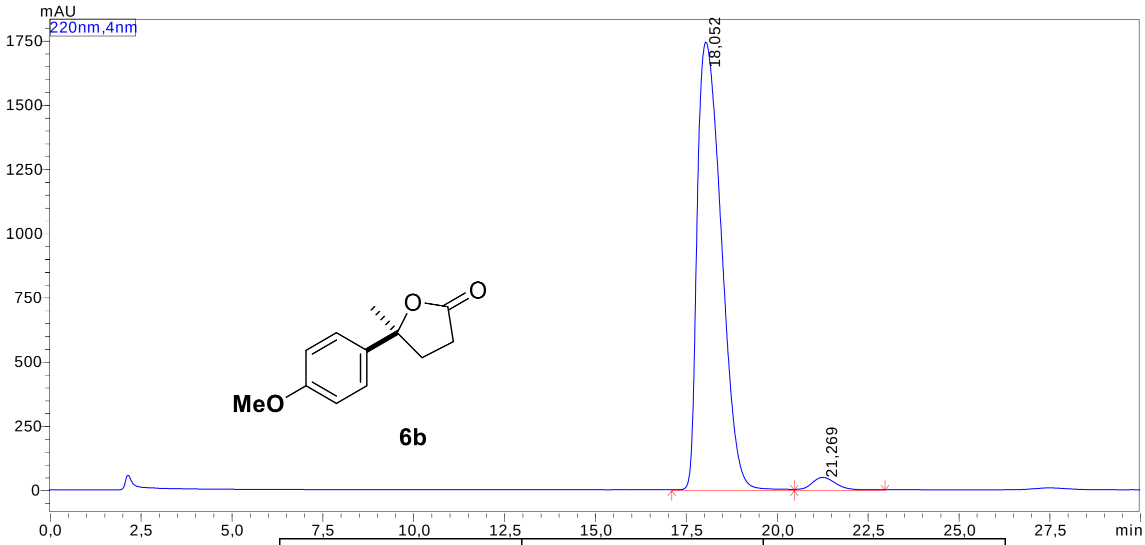

compound 6b	Time (min.)	Area %
1	18.0	97.42
2	21.2	2.58
Total		100

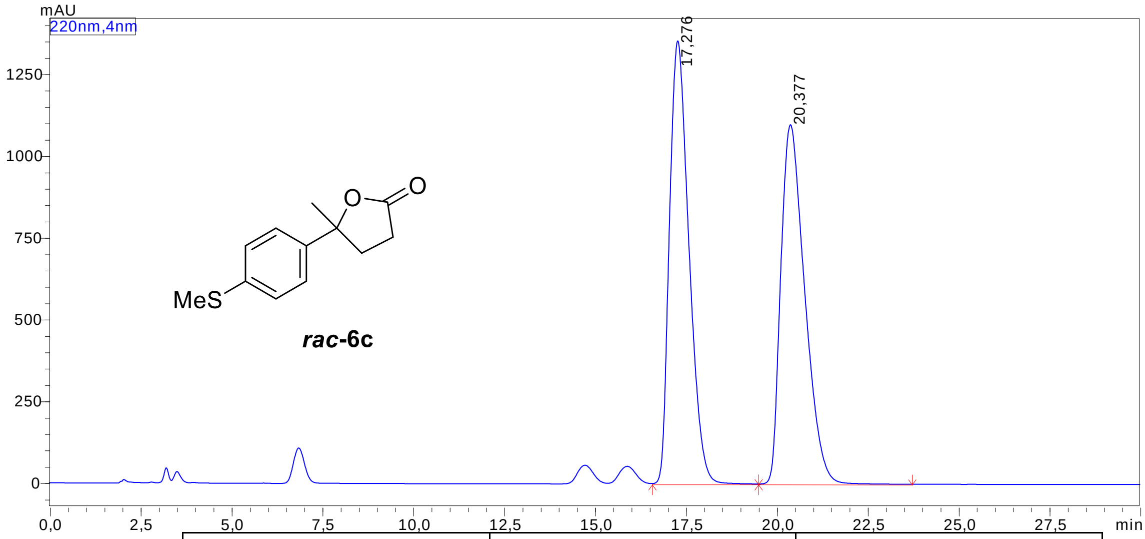

compound *rac*-6c	Time (min.)	Area %
1	17.2	50.09
2	20.37	49.91
Total		100

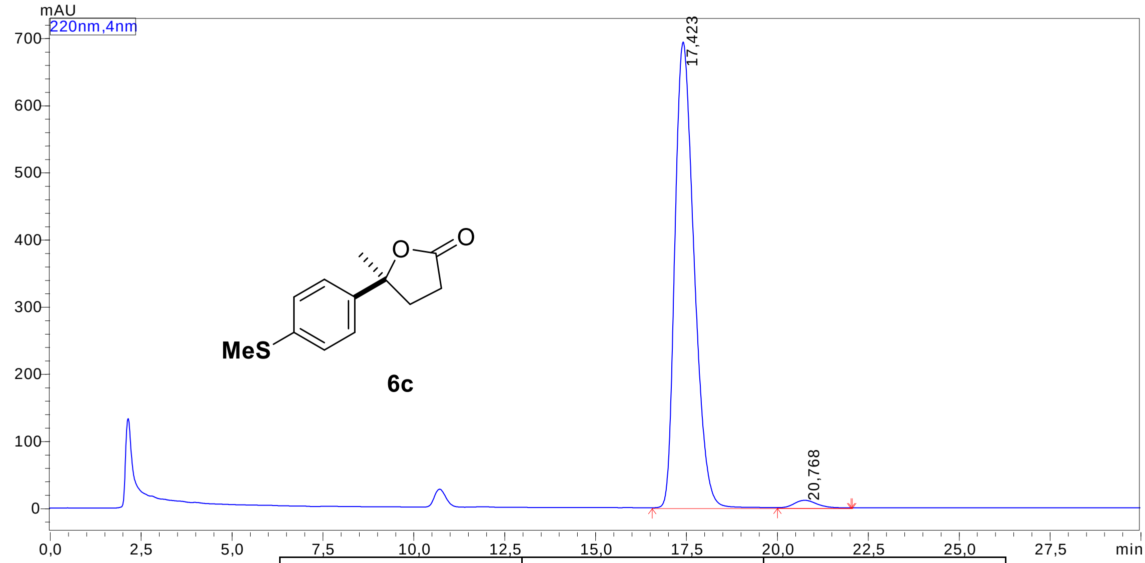

compound 6c	Time (min.)	Area %
1	17.4	98.21
2	20.7	1.79
Total		100

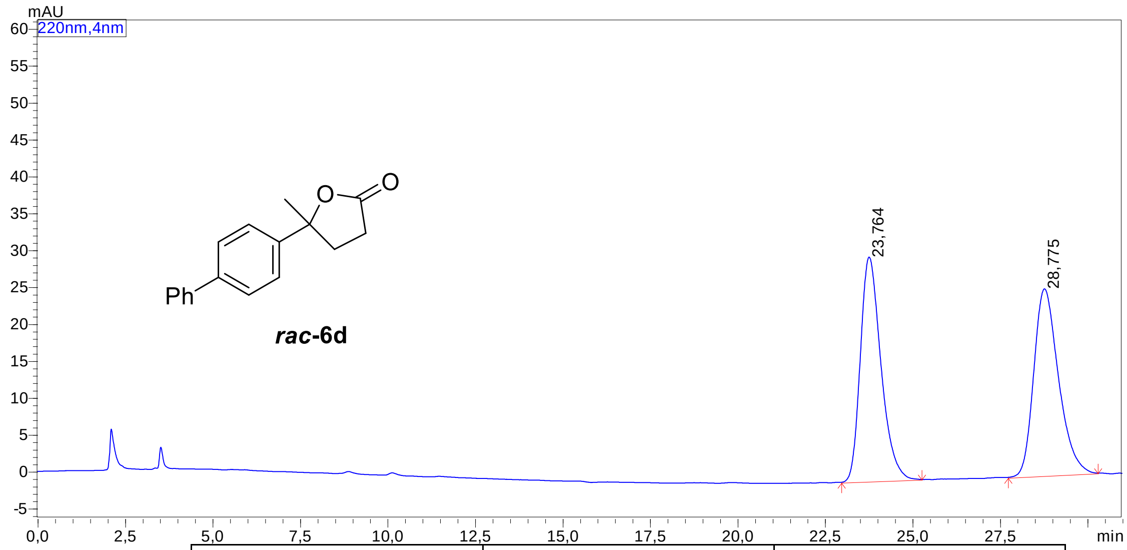

compound *rac*-6d	Time (min.)	Area %
1	23.7	50.07
2	28.7	49.93
Total		100

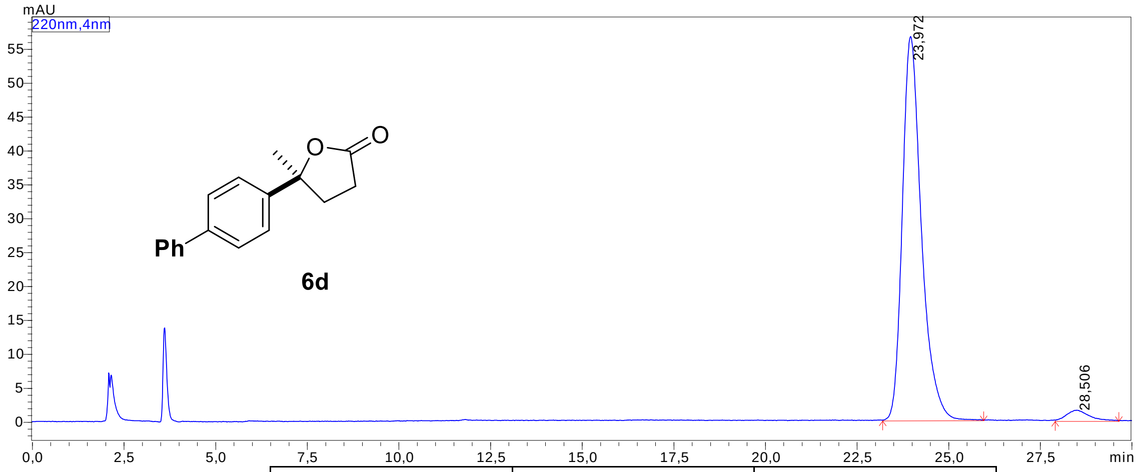

compound 6d	Time (min.)	Area %
1	23.9	96.99
2	28.5	3.00
Total		100

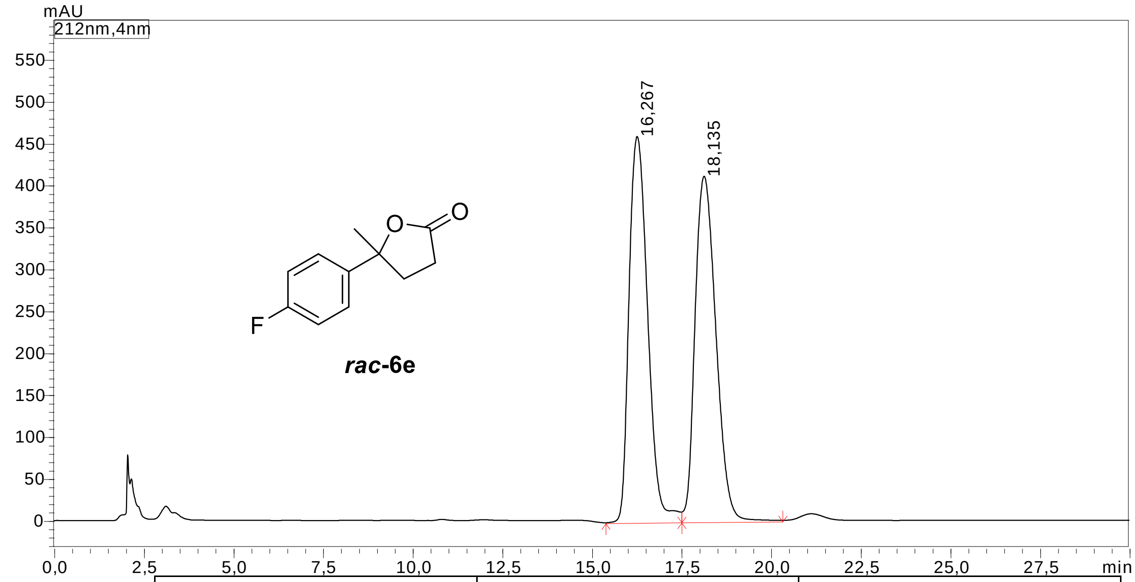

compound *rac*-6e	Time (min.)	Area %
1	16.2	49.97
2	18.1	50.03
Total		100

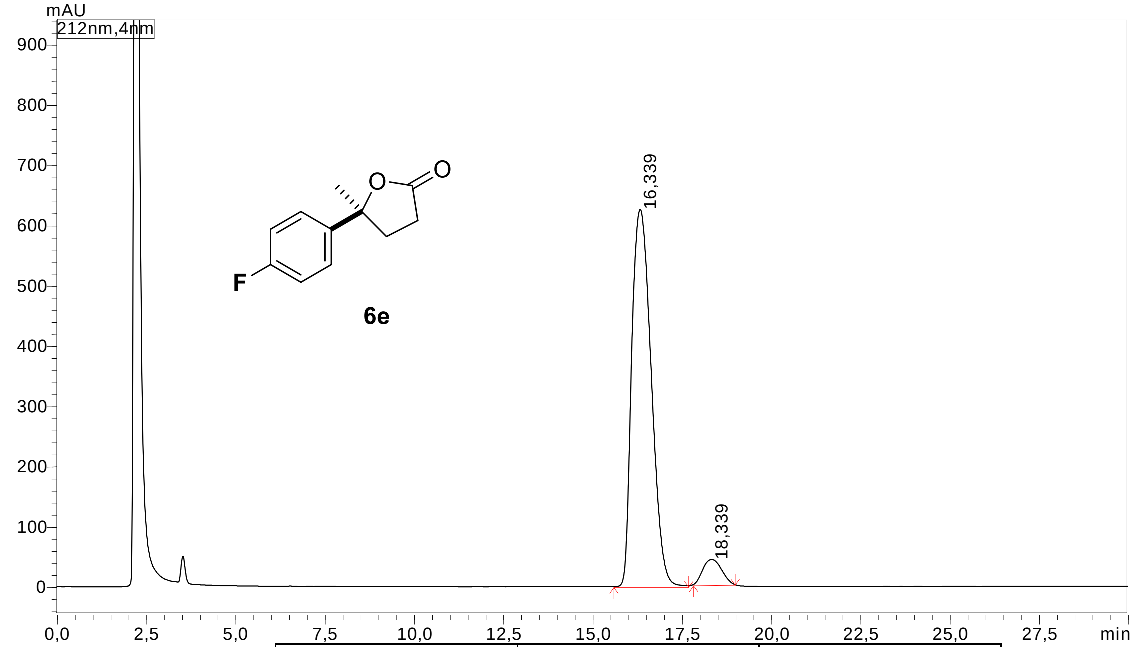

compound 6e	Time (min.)	Area %
1	16.3	94.01
2	18.3	5.99
Total		100

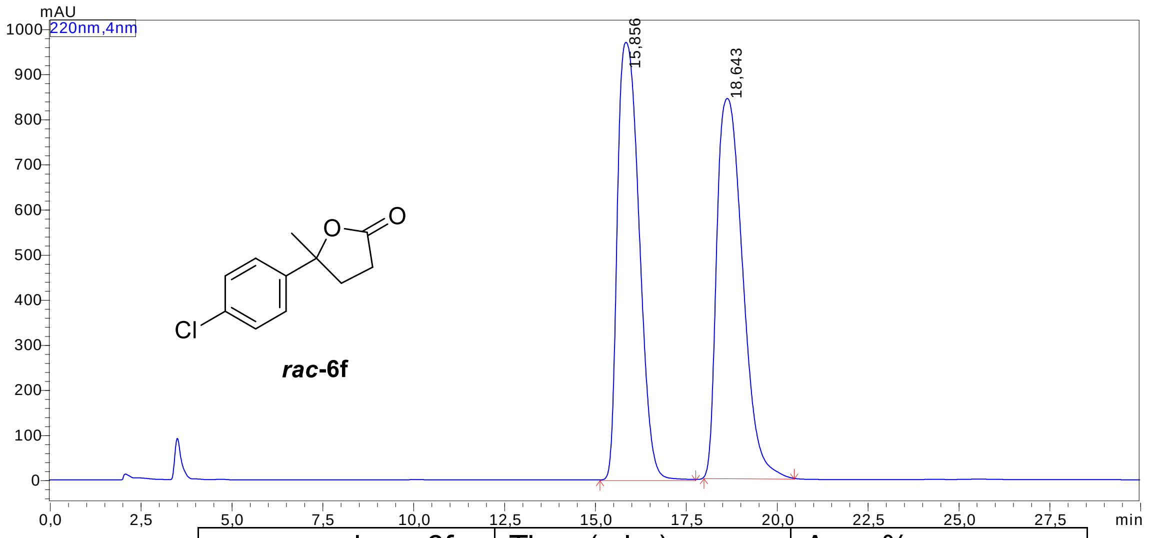

compound *rac*-6f	Time (min.)	Area %
1	15.8	49.22
2	18.6	50.78
Total		100

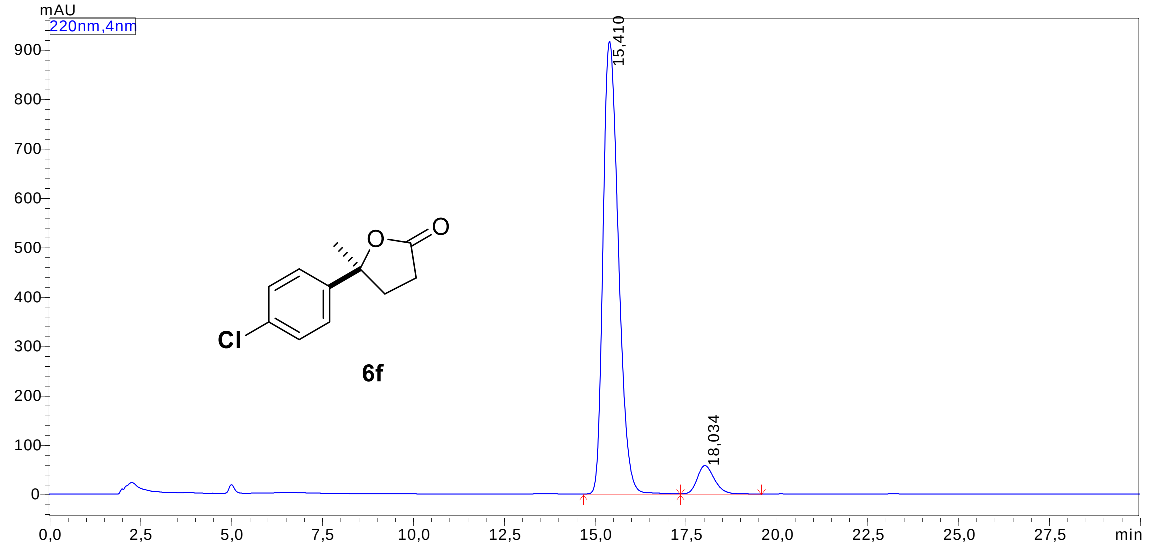

compound 6f	Time (min.)	Area %
1	15.4	93.5
2	18.0	6.5
Total		100

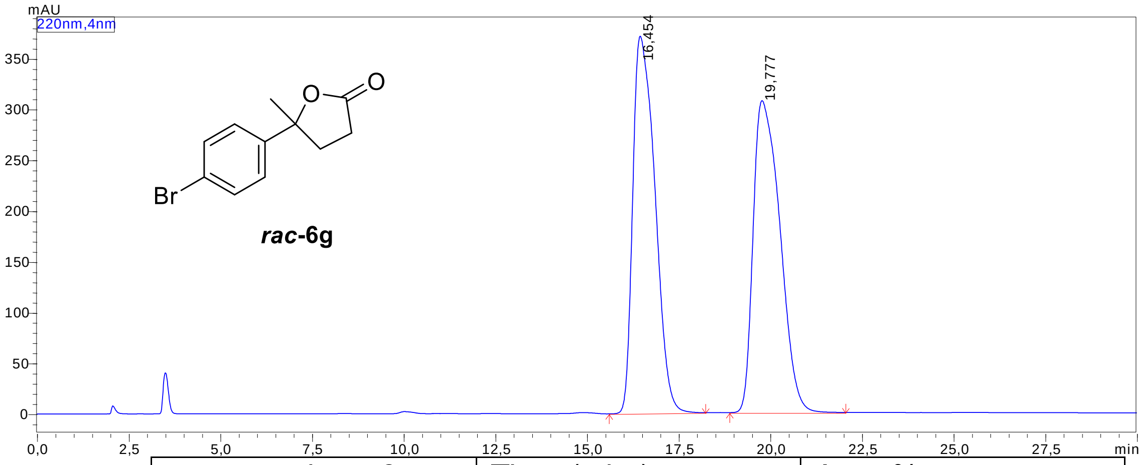

compound *rac*-6g	Time (min.)	Area %
1	16.4	50.07
2	19.7	49.93
Total		100

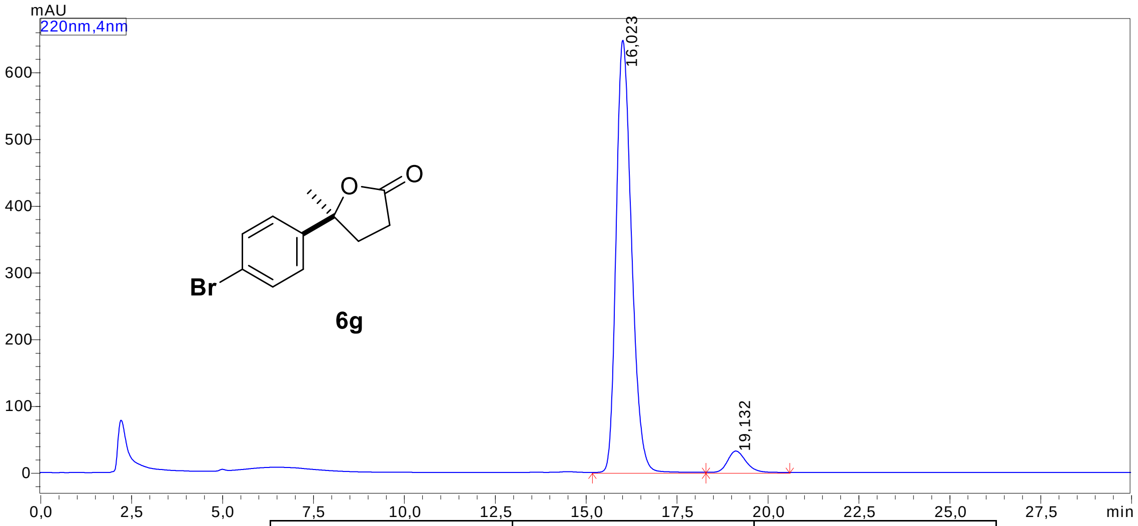

compound 6g	Time (min.)	Area %
1	16.0	94.48
2	19.1	5.52
Total		100

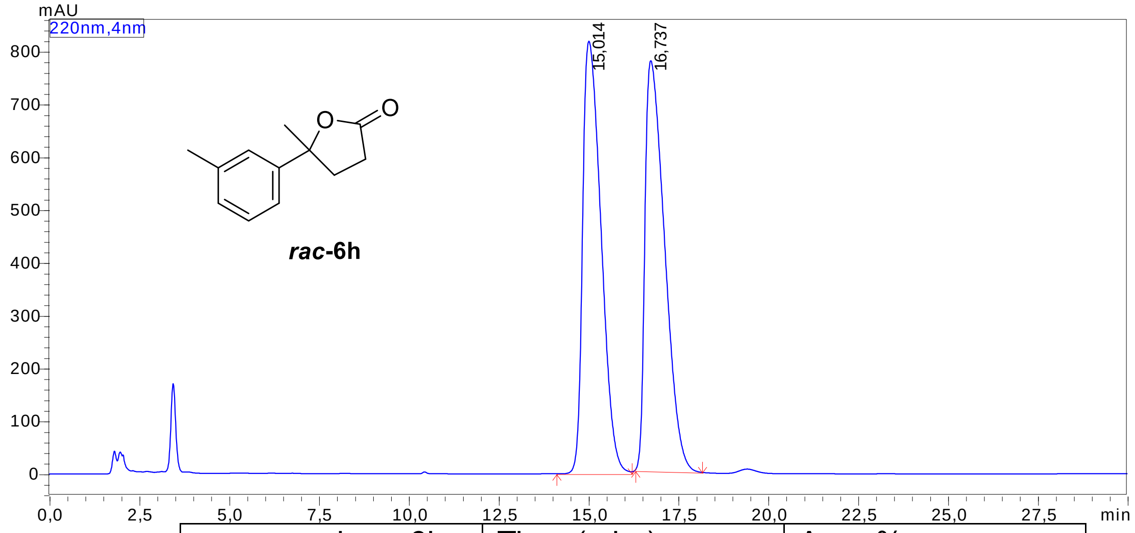

compound *rac*-6h	Time (min.)	Area %
1	15.0	49.72
2	16.7	50.28
Total		100

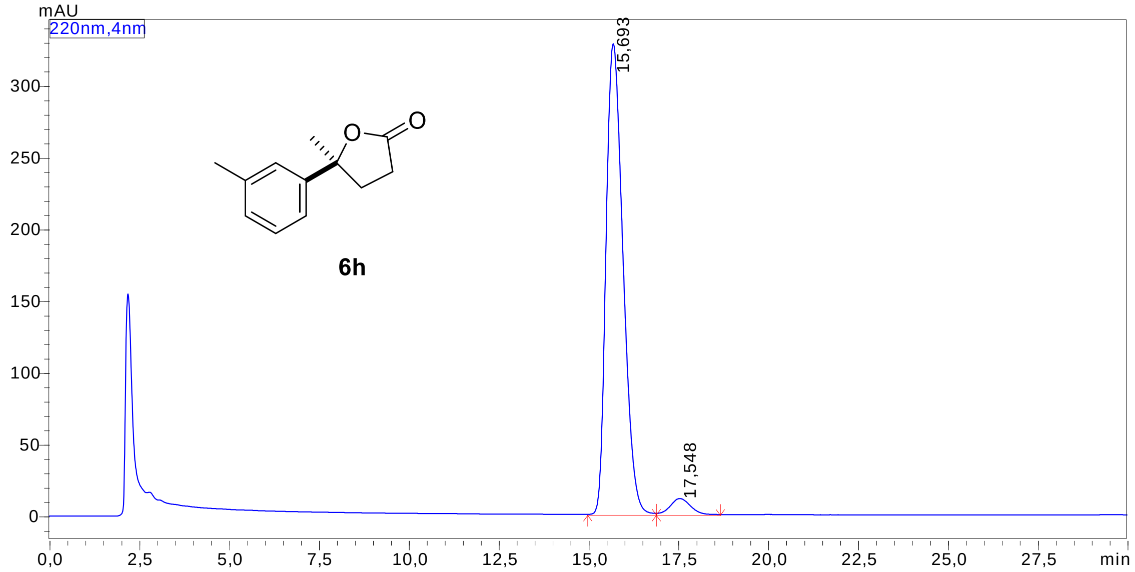

compound 6h	Time (min.)	Area %
1	15.7	96.16
2	17.5	3.84
Total		100

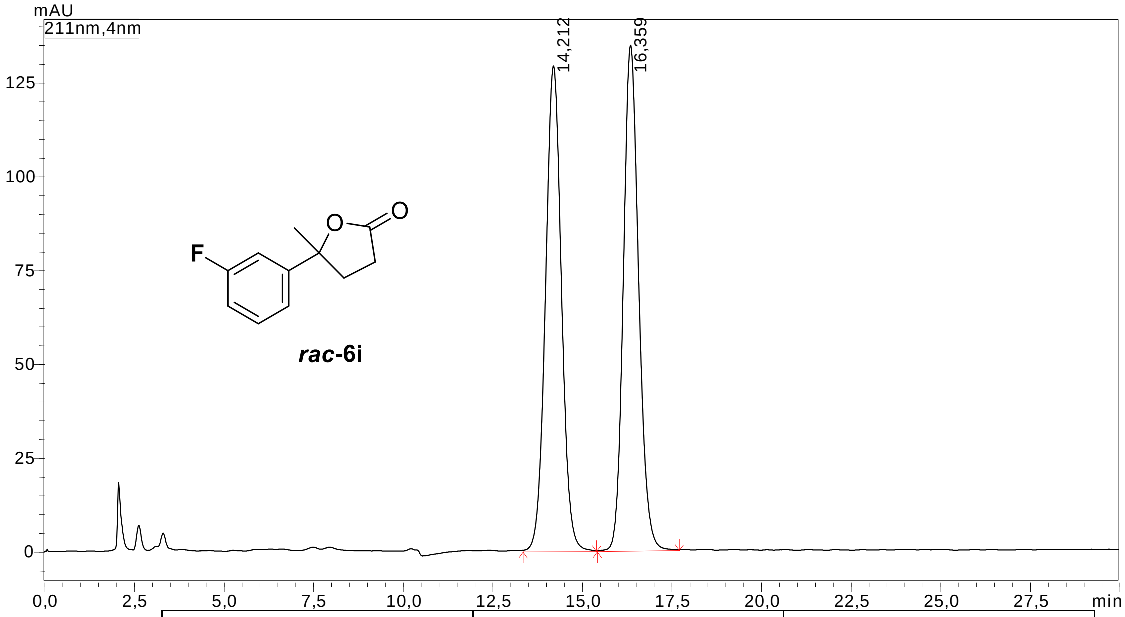

compound *rac*-6i	Time (min.)	Area %
1	14.2	49.98
2	16.3	50.02
Total		100

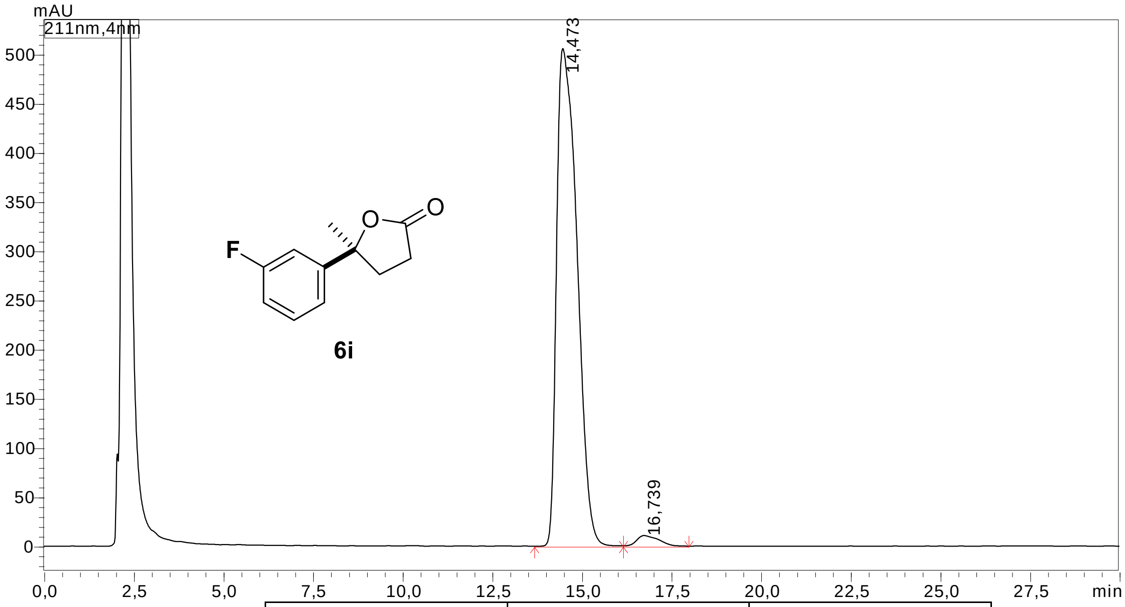

compound 6i	Time (min.)	Area %
1	14.4	97.76
2	16.7	2.23
Total		100

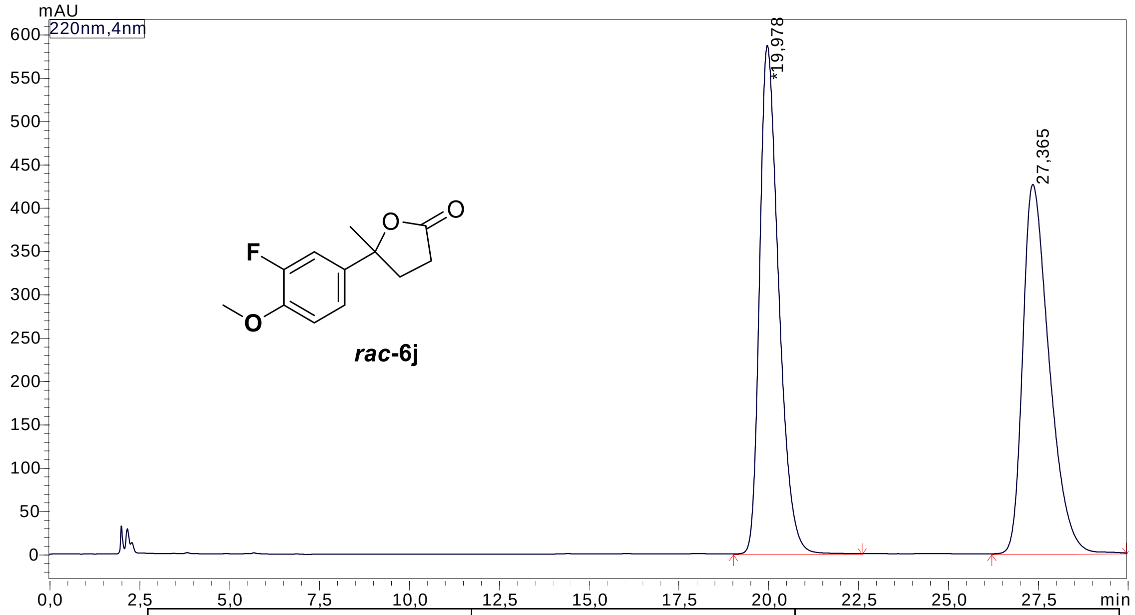

compound *rac*-6j	Time (min.)	Area %
1	19.9	49.90
2	27.3	50.10
Total		100

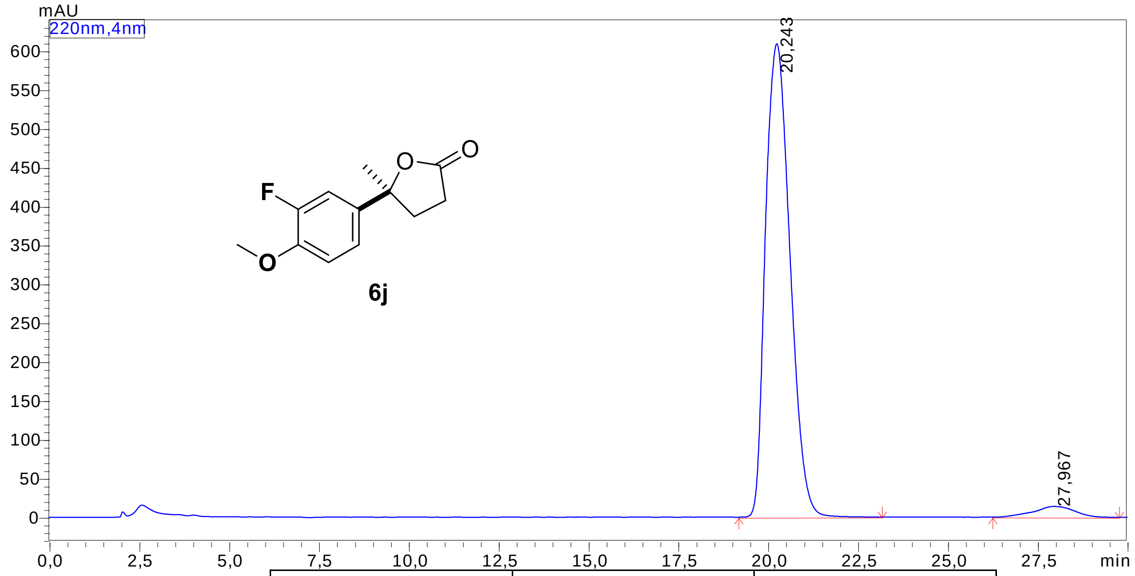

compound 6j	Time (min.)	Area %
1	20.2	96.30
2	27.9	3.70
Total		100

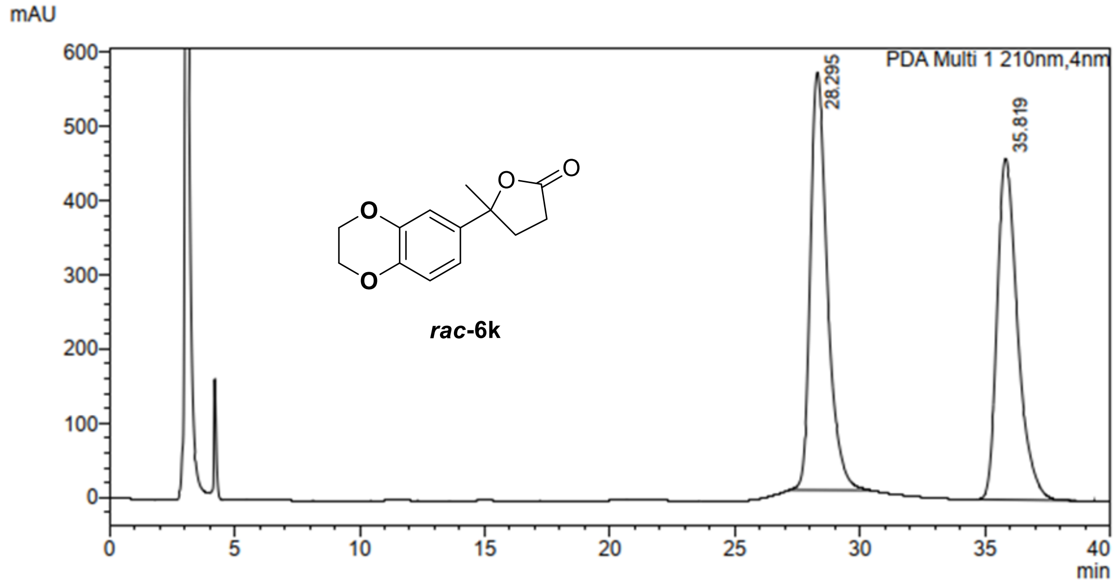

compound *rac*-6k	Time (min.)	Area %
1	28.29	49.90
2	35.81	50.10
Total		100

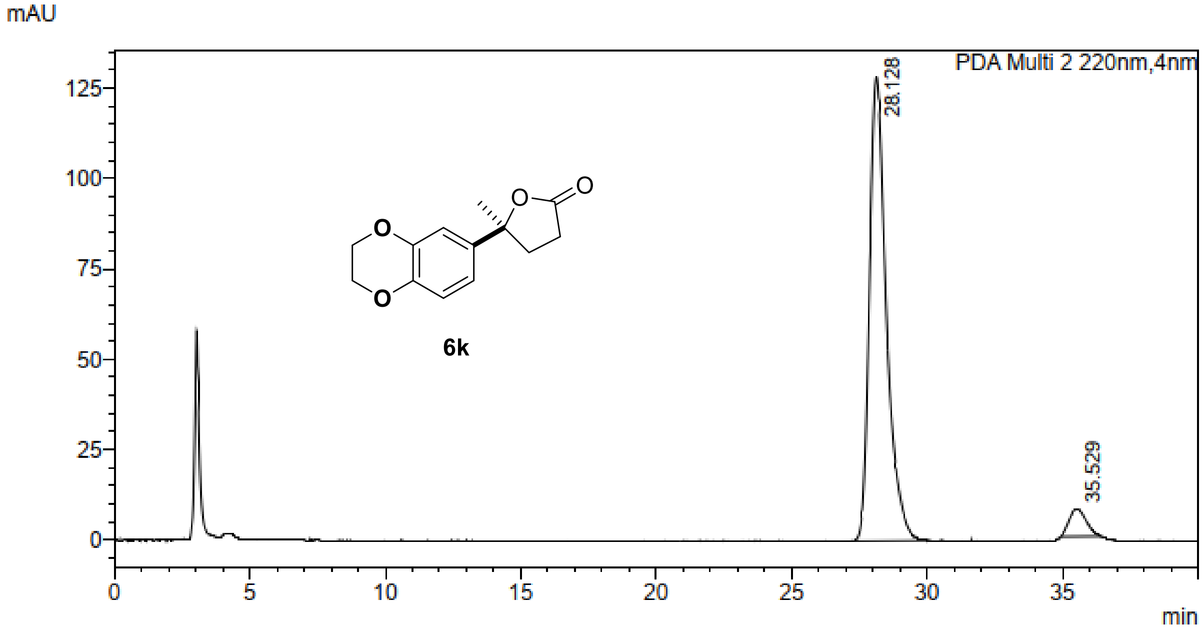

<Peak Table>

PDA Ch2 220nm

Peak#	Ret. Time	Area	Area%	Height	Name
1	28.128	5571346	94.311	128269	
2	35.529	336099	5.689	7724	
Total		5907445	100.000	135992	

compound 6k	Time (min.)	Area %
1	28.12	94.31
2	35.52	5.689
Total		100

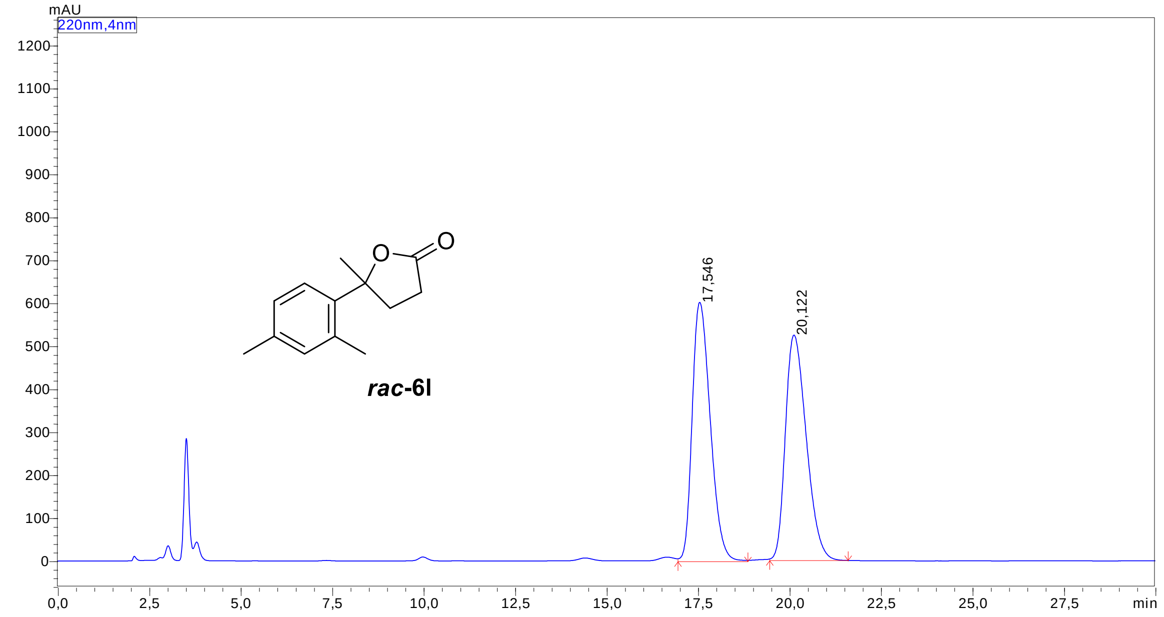

compound *rac*-6l	Time (min.)	Area %
1	17.54	49.98
2	20.12	50.02
Total		100

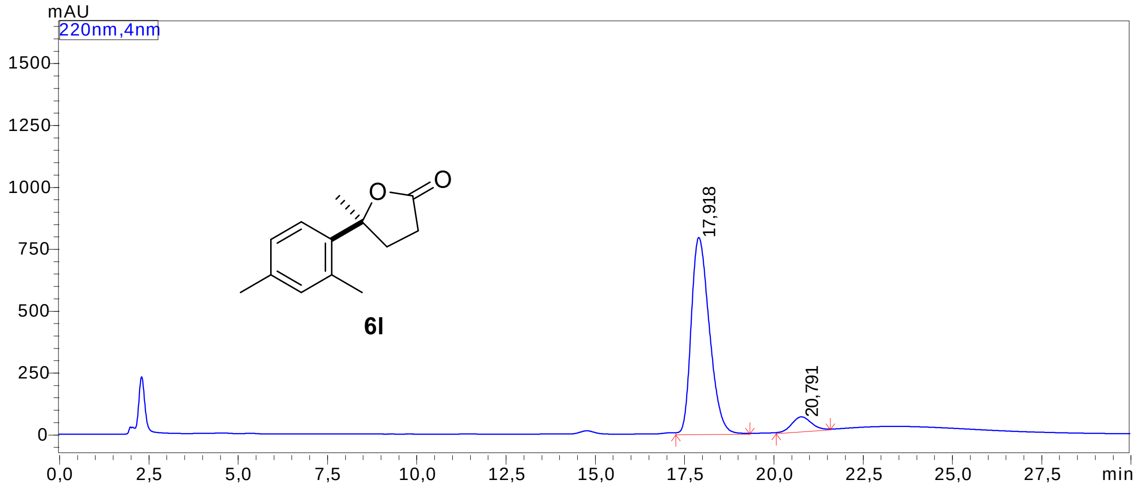

compound 6l	Time (min.)	Area %
1	17.91	93.06
2	20.79	6.94
Total		100

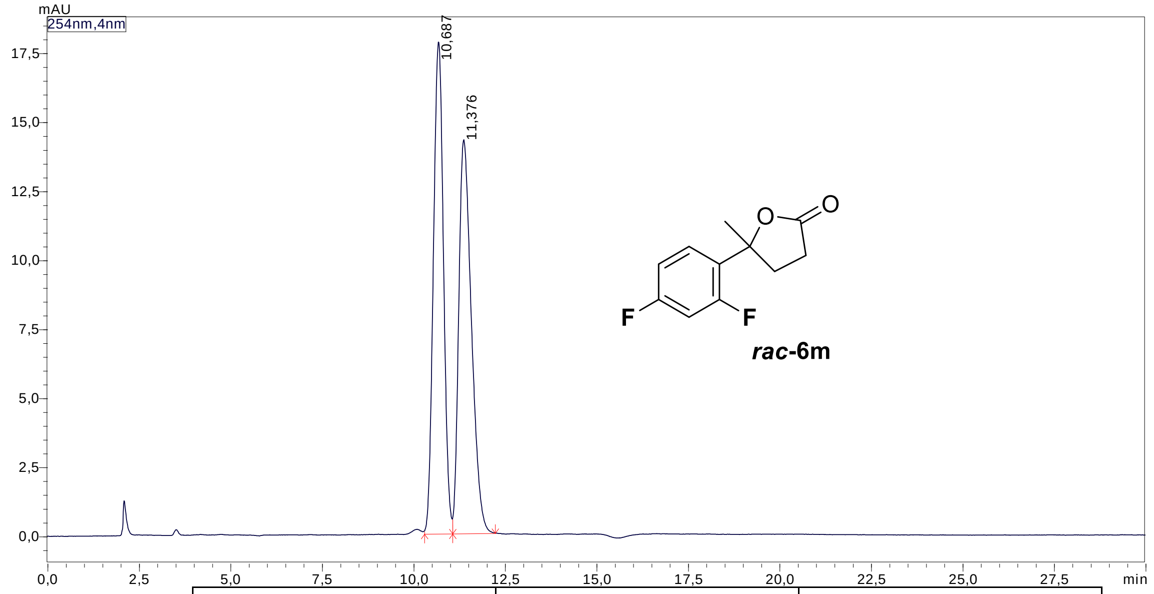

compound *rac*-6m	Time (min.)	Area %
1	10.68	49.84
2	11.37	50.16
Total		100

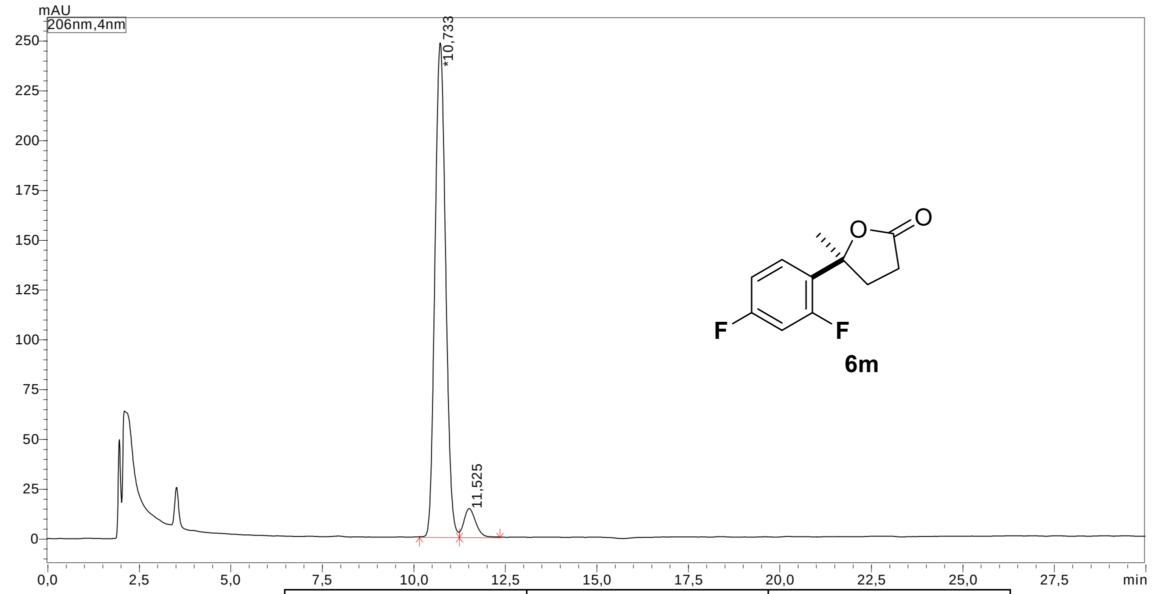

compound 6m	Time (min.)	Area %
1	10.73	94.12
2	11.52	5.88
Total		100

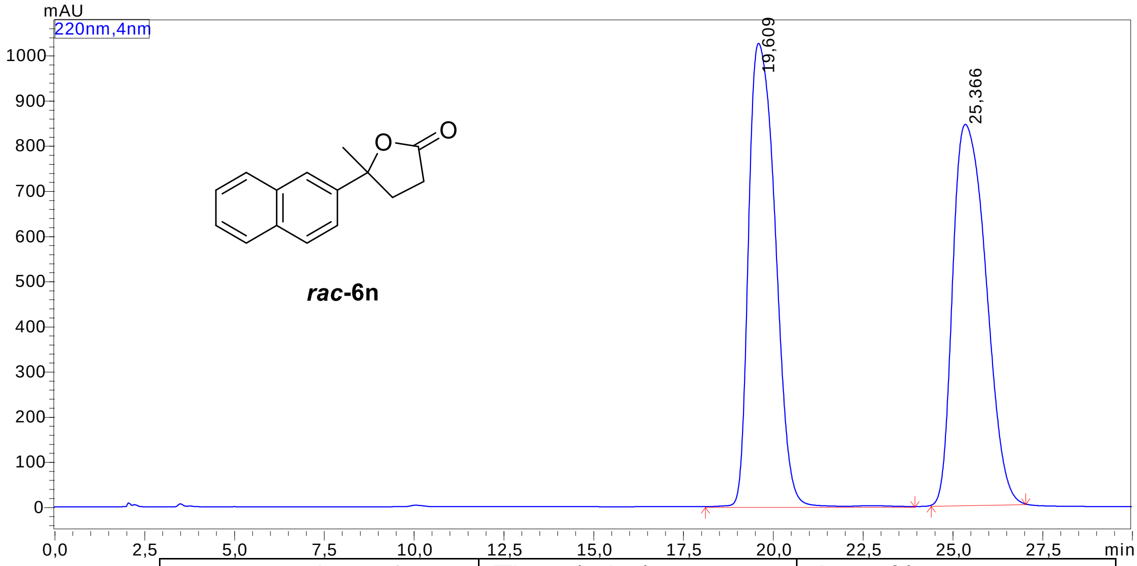

compound *rac*-6n	Time (min.)	Area %
1	19.6	49.38
2	25.3	50.62
Total		100

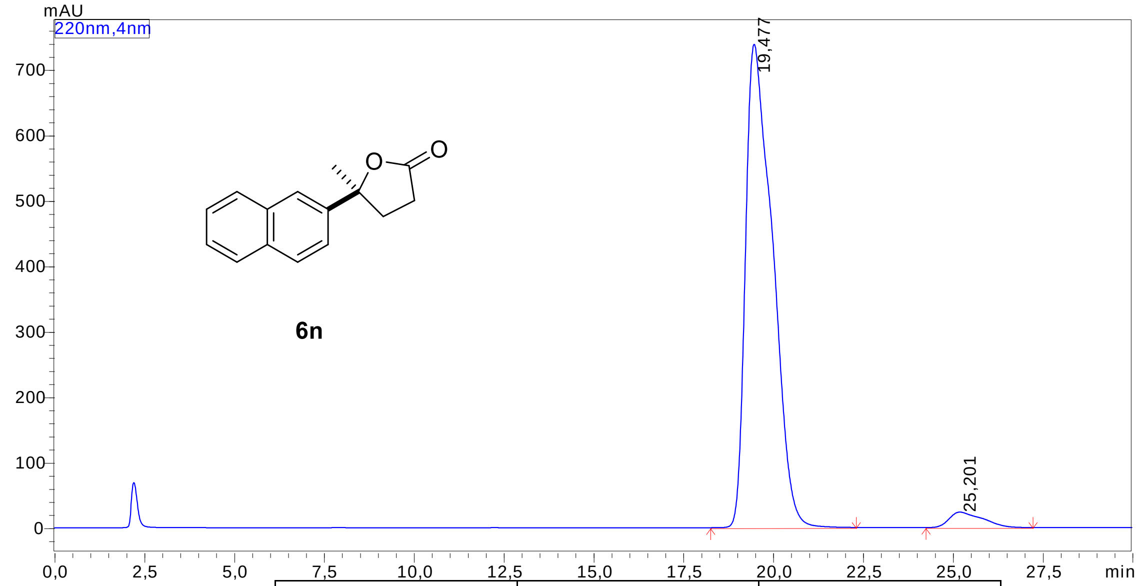

compound 6n	Time (min.)	Area %
1	19.4	96.22
2	25.2	3.78
Total		100

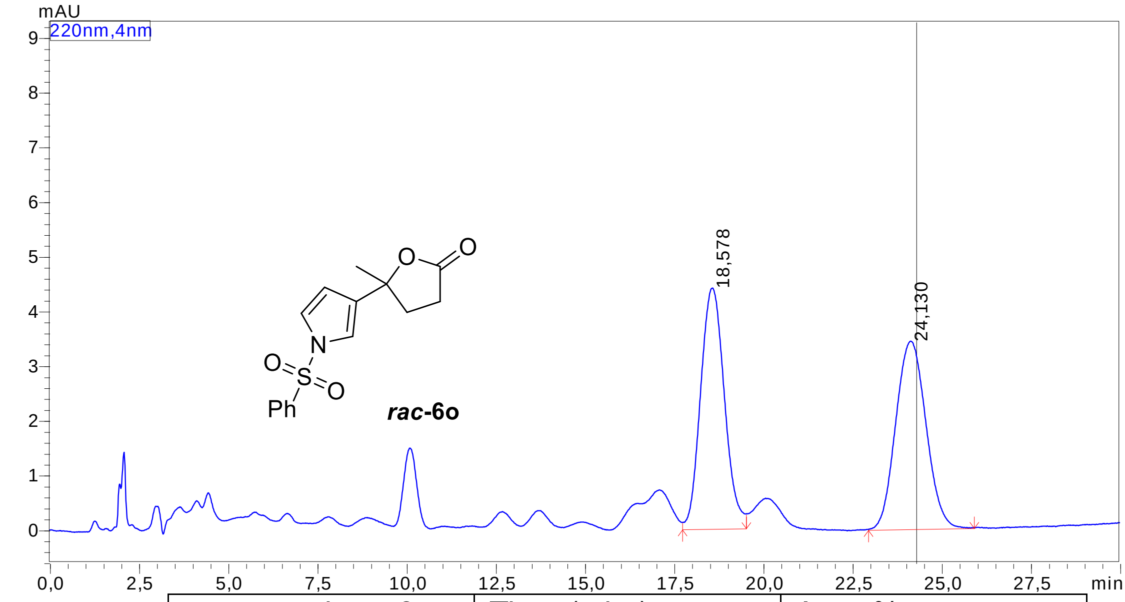

compound *rac*-6o	Time (min.)	Area %
1	18.5	50.5
2	24.1	49.5
Total		100

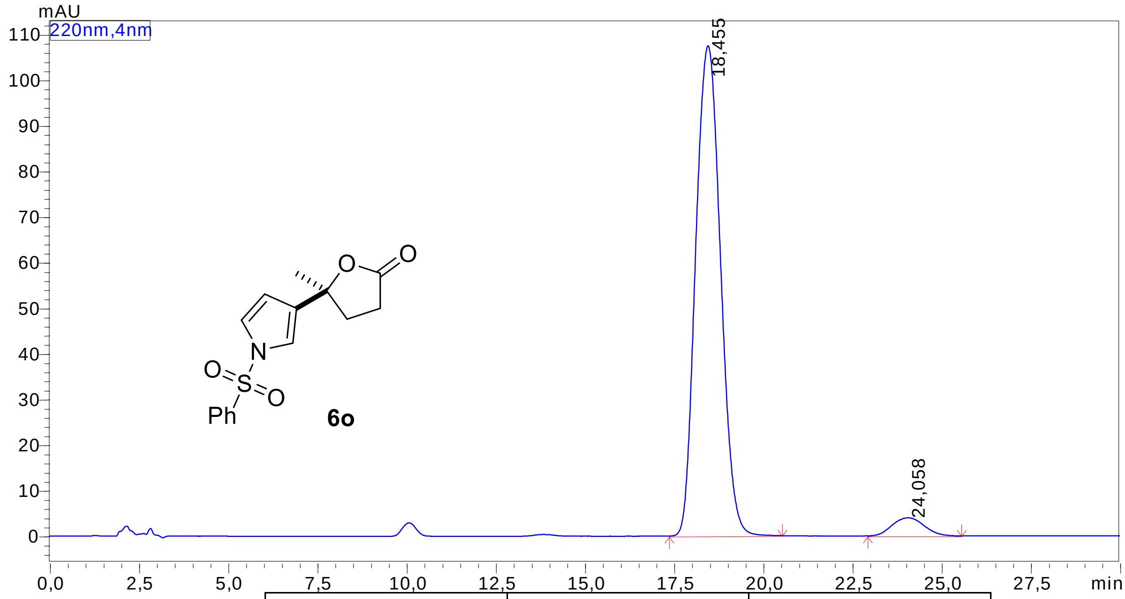

compound 6o	Time (min.)	Area %
1	18.45	95.48
2	24.05	4.52
Total		100

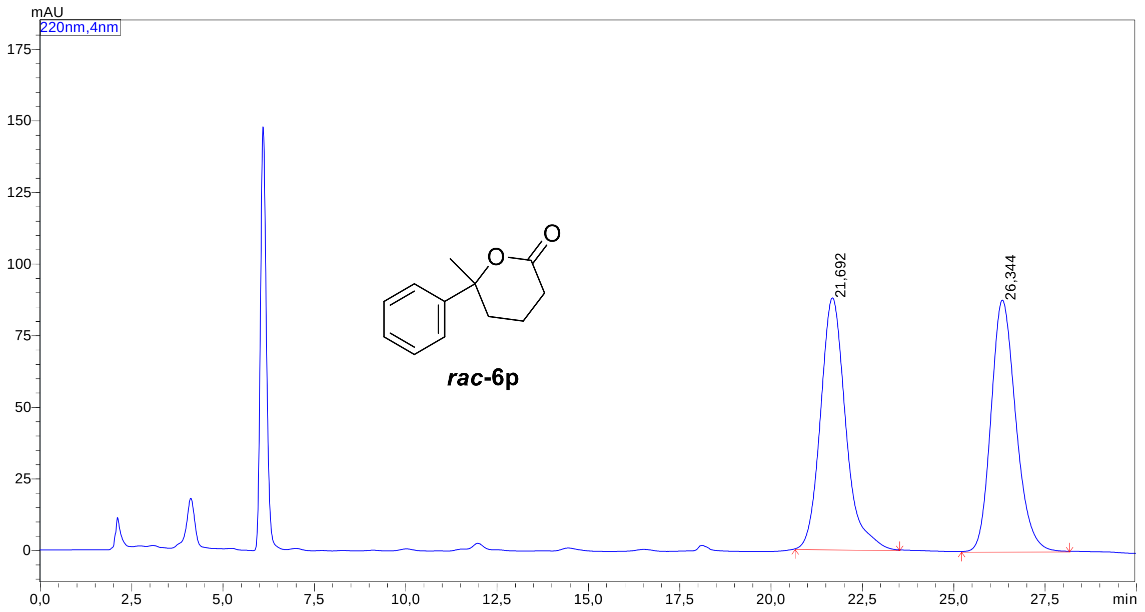

compound *rac*-6p	Time (min.)	Area %
1	21.69	49.98
2	26.34	50.02
Total		100

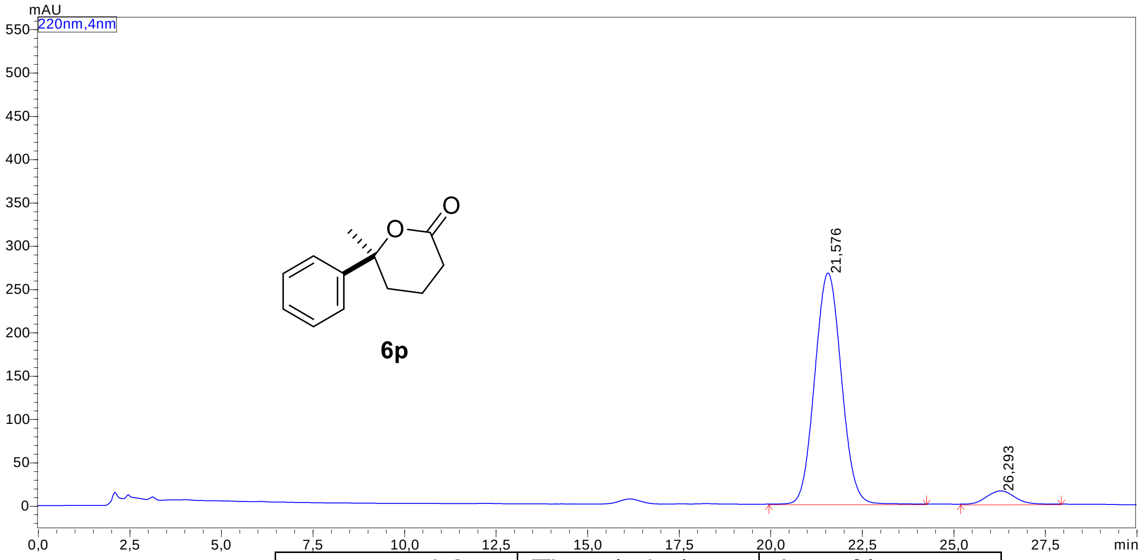

compound 6p	Time (min.)	Area %
1	21.57	94.35
2	26.29	5.65
Total		100

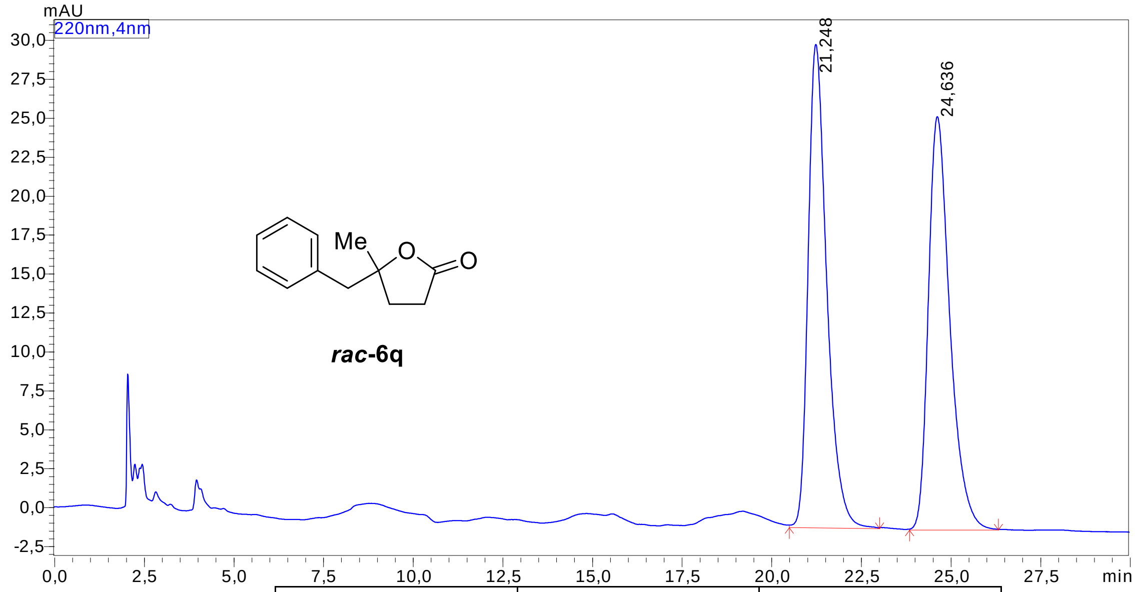

Entry rac-6q	Time (min.)	Area %
1	21.2	50.35
2	24.6	49.65
Total		100

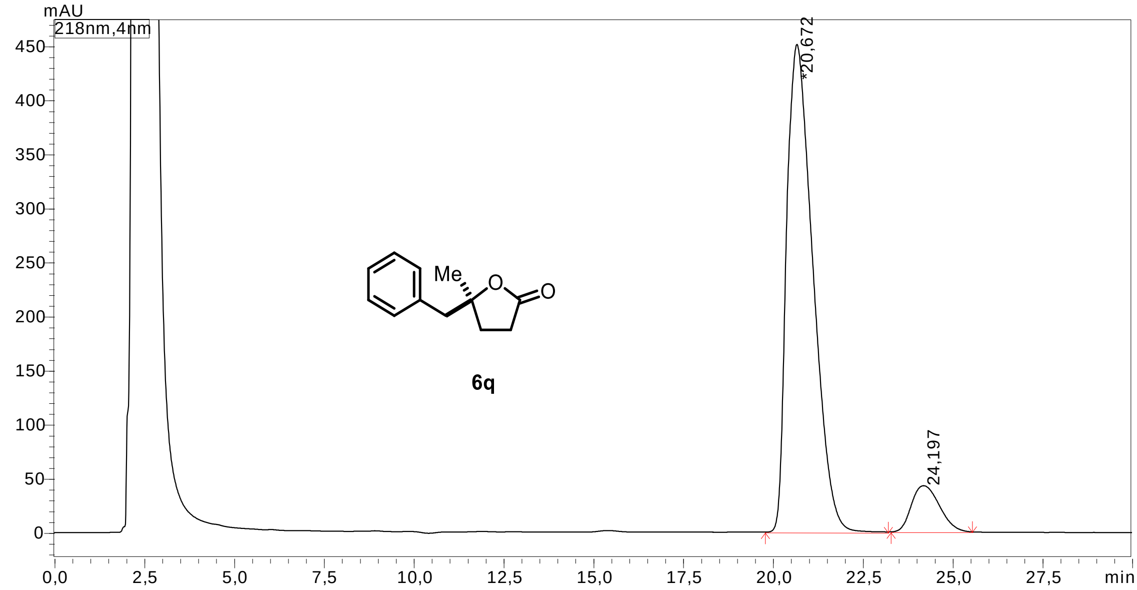

compound 6q	Time (min.)	Area %
1	20.6	90.94
2	24.2	9.06
Total		100

Phenethyl Rac

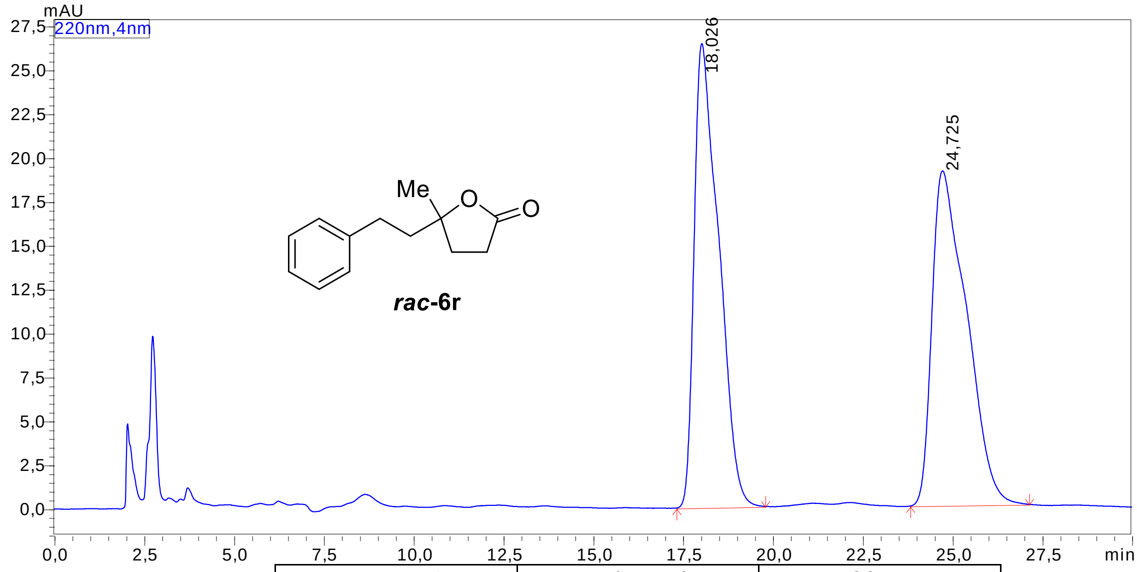

Entry rac-6r	Time (min.)	Area %
1	18.0	49.63
2	24.7	50.37
Total		100

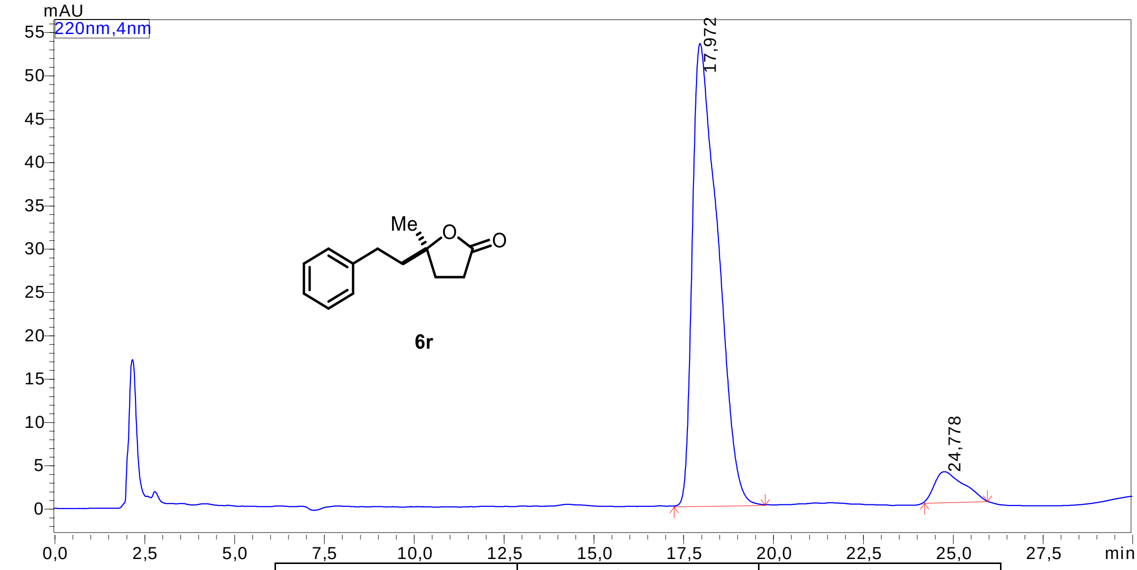

Entry 6r	Time (min.)	Area %
1	18.0	91.49
2	24.7	8.51
Total		100

GC trace for Racemic

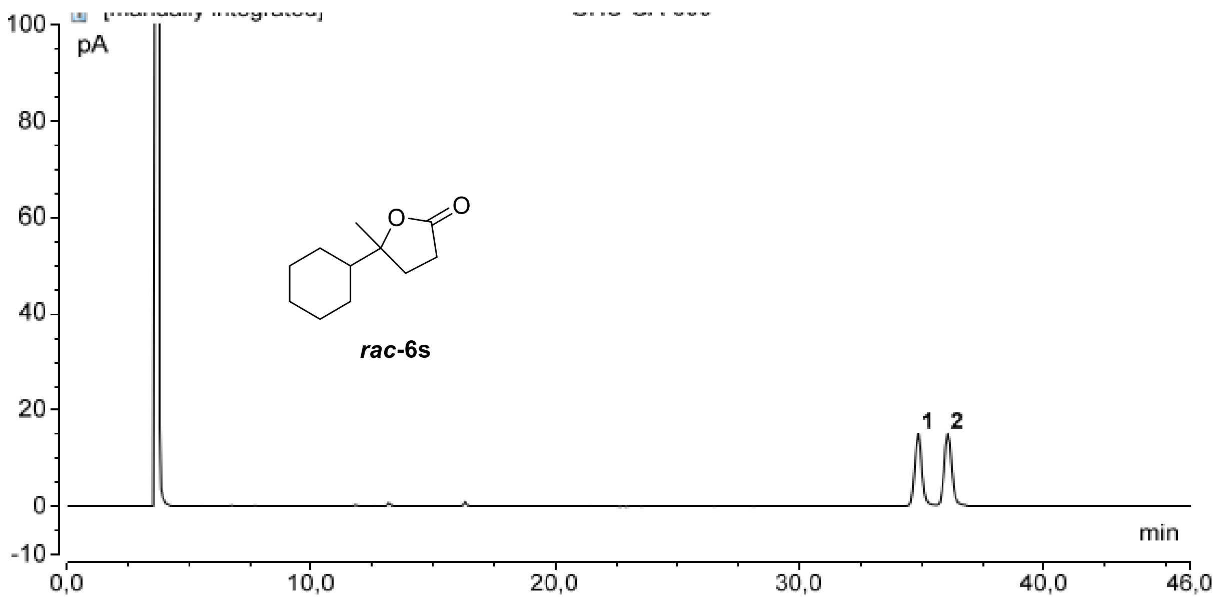

Sample:	**GHS-GA-699**	Instrument:	**GC_Front**
Sequenz:	**Lactonization**	Measured:	**12.11.19 16:29**
Sequenz date:	**04.10.19**	Processing M.:	MPI
		Report-File:	GC1_peak ratio

No.	Ret.Time min	Rel.Area %	Peak Name
1	34,86	48,44	Component 34
2	36,07	51,56	Component 26

Instrument parameters:

Column:	30 m	Cyclosil B
Temperature:	220/140 5/min170 40min iso/350	
Gas:	0,50 bar	H2
Sample size:	1,0 µL	

GC trace for enantiopure sample

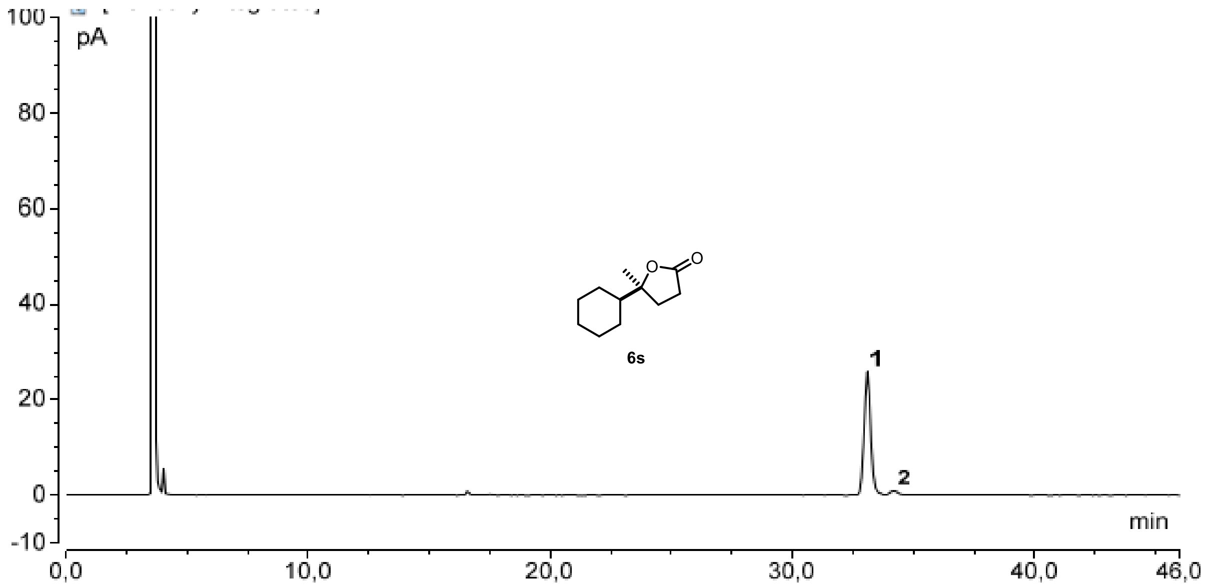

Sample:	**GHS-GB-256-2**	Instrument:	**GC_Front**
Sequenz:	**Lactonization**	Measured:	**20.02.22 20:34**
Sequenz date:	**04.10.19**	Processing M.:	MPI
		Report-File:	GC1_peak ratio

**No.**	**Ret.Time min**	**Rel.Area %**	**Peak Name**
1	33,10	96,77	Component 3
2	34,19	3,23	Component 31

Instrument parameters:

Column:	30 m	Cyclosil B
Temperature:	220/140 5/min170 40min iso/350	
Gas:	0,50 bar	H2
Sample size:	1,0 µL	

GC trace for racemate

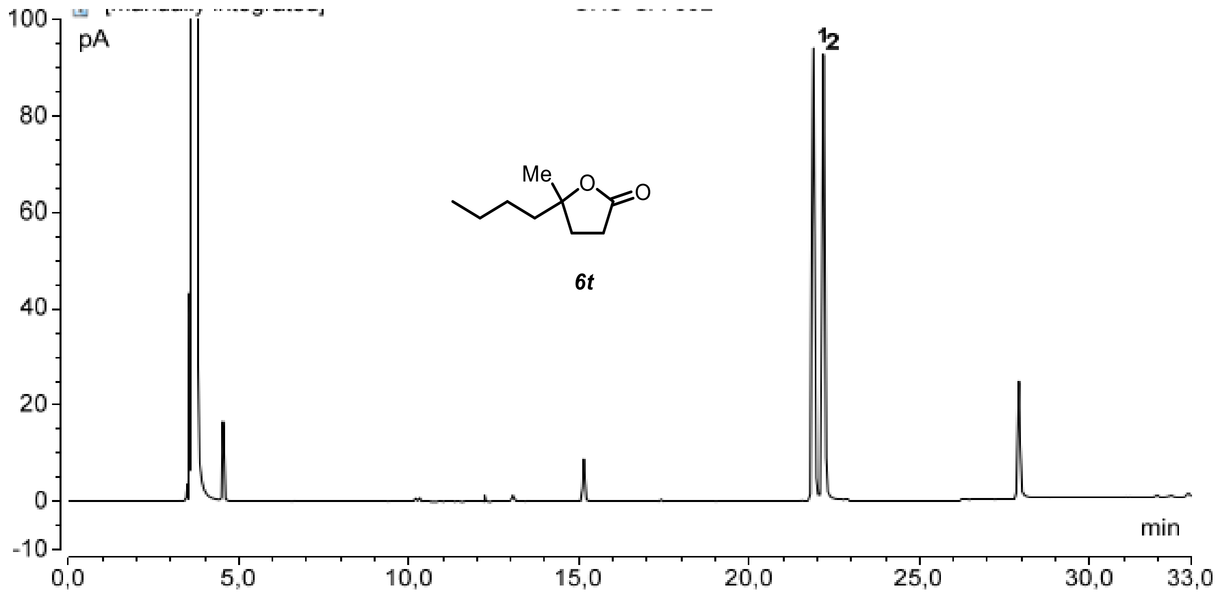

Sample:	**GHS-GA-692**	Instrument:	**GC_Front**
Sequenz:	**Lactonization**	Measured:	**04.10.19 16:01**
Sequenz date:	**04.10.19**	Processing M.:	MPI
		Report-File:	GC1_peak ratio

No.	Ret.Time min	Rel.Area %	Peak Name
1	21,89	49,31	Component 1
2	22,18	50,69	Component 2

Instrument parameters:

Column:	30 m	Cyclosil B
Temperature:	220/80 5/min 220 5min iso/350	
Gas:	0,50 bar	H2
Sample size:	1,0 µL	

GC trace for Enantiopure sample

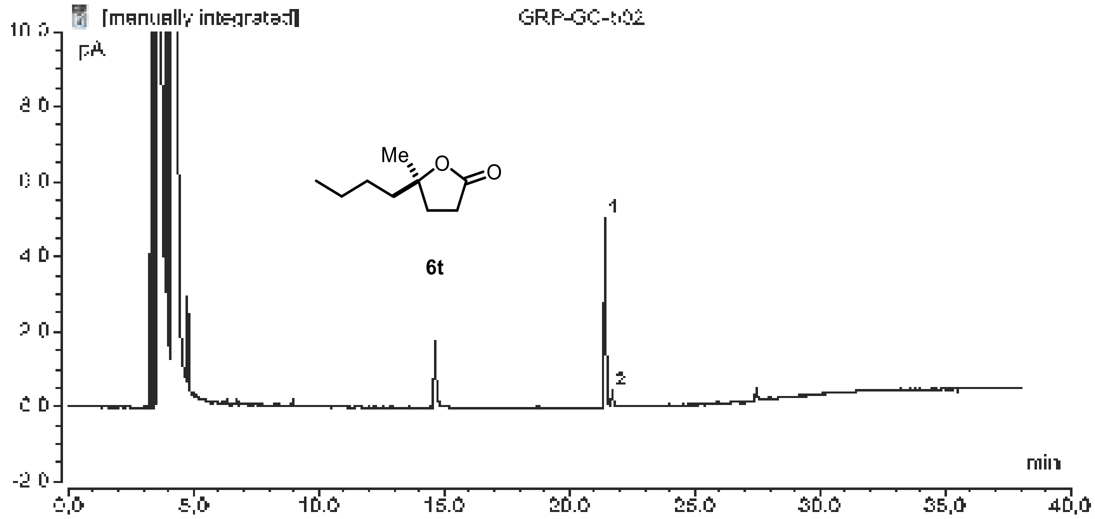

Sample:	GRP-GC-502	Instrument:	GC_Front
Sequenz:	Lactonization	Measured:	24.05.22 14:31
Sequenz date:	08.03.22	Processing M.:	MPI
		Report-File:	GC1_peak ratio

No.	Ret.Time min	Rel.Area %	Peak Name
1	21,41	93,46	Component 1
2	21,70	6,54	Component 2

Instrument parameters:

Column:	30 m	Cyclosil B
Temperature:	80/220 5/min 220 5min	
Gas:	0,50 bar	H2
Sample size:	1,0 µL	

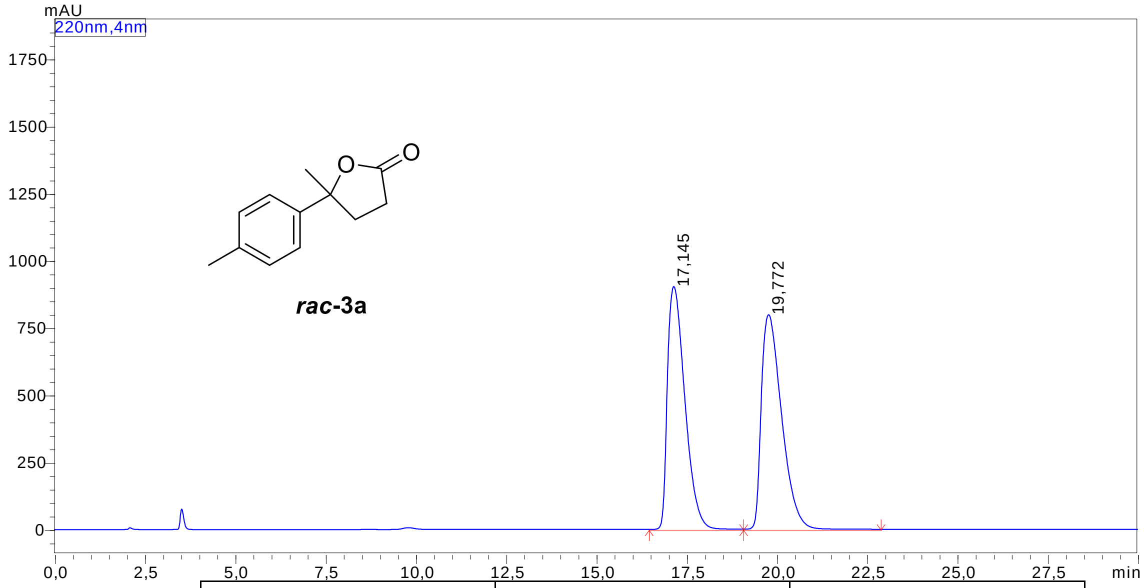

compound *rac*-3a	Time (min.)	Area %
1	17.14	49.35
2	19.77	50.65
Total		100

Application:

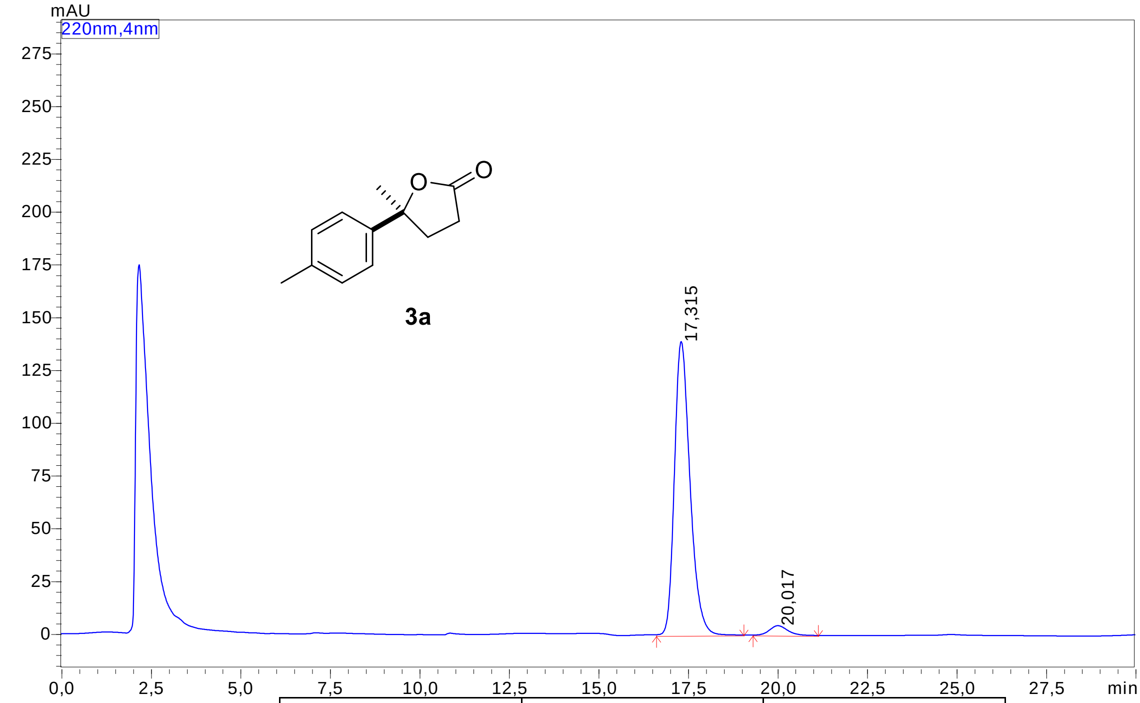

compound 3a	Time (min.)	Area %
1	17.31	96.55
2	20.01	3.45
Total		100

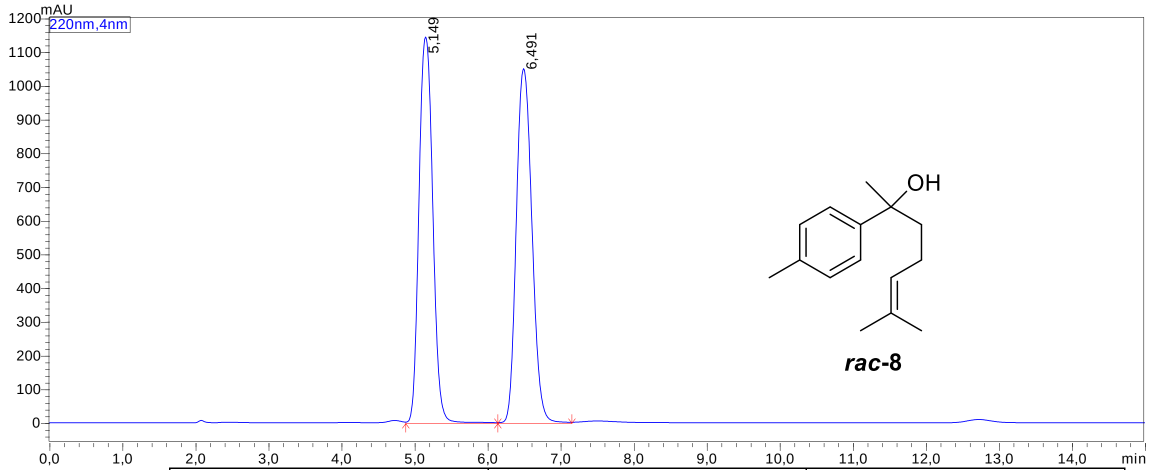

compound *rac*-8	Time (min.)	Area %
1	5.14	49.12
2	6.49	50.88
Total		100

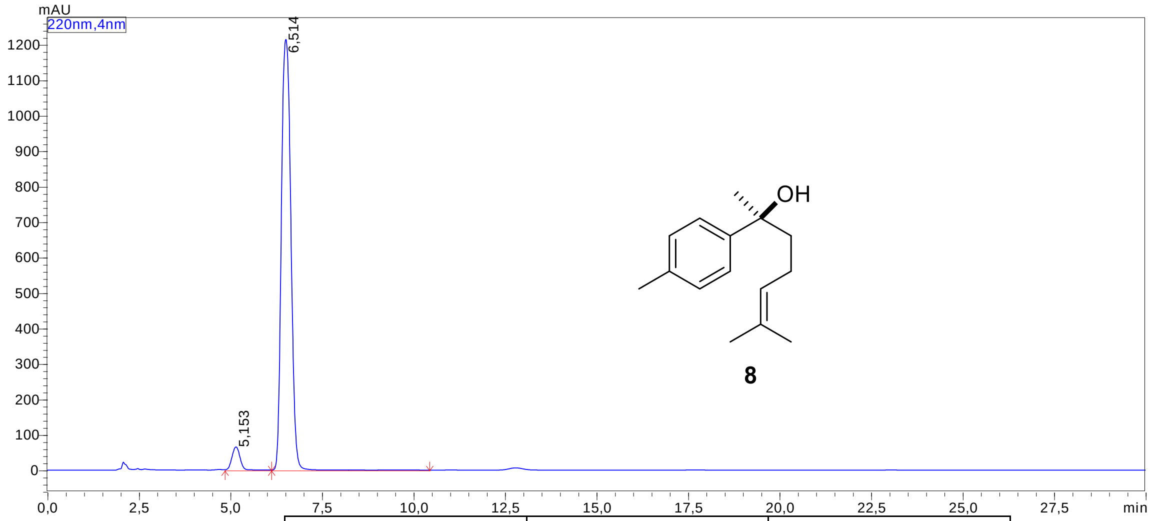

compound 8	Time (min.)	Area %
1	5.15	4.43
2	6.51	95.57
Total		100

12. Optimized Cartersian Coordinates from Computational Analysis

Starting material (alkenoic acid 4a)

6	0.597784492	-0.014540196	0.558689919
6	1.781031325	-0.390567377	-0.263117852
6	2.430232009	-1.630662447	-0.065877153
6	2.279719324	0.468253754	-1.269165337
6	3.551099529	-1.987384489	-0.826622439
1	2.020255612	-2.332828498	0.675084498
6	3.403375133	0.113583366	-2.028710908
1	1.794741885	1.440199316	-1.446544510
6	4.045508395	-1.115176291	-1.809671773
1	4.033908794	-2.962687839	-0.661270036
1	3.781257802	0.804039534	-2.798313695
1	4.922802396	-1.397091200	-2.411422818
6	-0.563818417	0.648912895	-0.151873401
1	-1.324836837	0.958594421	0.590766378
1	-0.229548201	1.571020842	-0.673423437
6	0.526806218	-0.312533493	1.876353667
1	-0.371310847	-0.067742695	2.464401476
1	1.365905113	-0.791008287	2.404333999
6	-1.214564534	-0.287623444	-1.193055975
1	-2.082090715	0.225232789	-1.659282263
1	-0.497987550	-0.558331677	-1.990901650
6	-1.695266971	-1.580875193	-0.567442368
8	-1.318441054	-2.698667869	-0.858888749
8	-2.615818137	-1.351946537	0.410531115
1	-2.831804385	-2.239936445	0.769950743

Final product (lactone: 6a)

6	6.774013674	-2.687395964	-0.129050052
6	8.210160987	-3.022098096	-0.521568068
6	6.850753044	-1.175230655	0.229327929
1	6.026659849	-2.884070957	-0.921162808
1	6.474067853	-3.259314589	0.771130453
1	8.412664976	-2.863072506	-1.602095212
1	8.538867039	-4.049633840	-0.278934340
8	8.222294488	-0.995198429	0.684719966
6	9.043314852	-2.007867257	0.259103449
8	10.226376568	-2.018614308	0.492803128
6	5.904400272	-0.787904483	1.356443631
6	4.520221496	-1.012597617	1.209857748
6	6.377931003	-0.203228469	2.544926090
6	3.627382519	-0.660345894	2.231667510
1	4.130812370	-1.472988814	0.287924831
6	5.482443313	0.148852331	3.567502904
1	7.456571992	-0.030536734	2.661186344
6	4.106508897	-0.076691258	3.415560450
1	2.549924385	-0.844656553	2.102536651

1	5.867702734	0.604582129	4.492505911
6	6.651887518	-0.270915795	-0.994427642
1	5.604906739	-0.321349434	-1.351212585
1	6.879244705	0.778427191	-0.725876317
1	7.321956609	-0.574228447	-1.824357659
1	3.406612707	0.200333229	4.218630637

IDPi Catalyst (7g)

6	2.372138261	1.542155697	2.432246154
6	-0.616451184	-1.124064979	1.269257496
6	2.982691338	0.296559308	2.182429884
6	2.679264669	2.598901863	1.544519630
6	-1.892272112	-0.629422570	0.919386452
6	-0.422965225	-2.478781836	1.569235845
1	0.251494796	-0.453303035	1.297908709
6	3.880026780	0.113675760	1.120994325
1	2.766341416	-0.565053838	2.827976407
6	3.578613809	2.428014184	0.488897856
1	2.203141986	3.583025045	1.671564084
6	-2.127647469	0.786286460	0.546405090
6	-2.965824557	-1.547622474	0.910928508
6	-1.484961006	-3.402642174	1.531106598
1	0.595746768	-2.810340851	1.806205550
6	4.210740180	1.183681063	0.265738595
1	4.327985677	-0.876363888	0.965655696
1	3.766901543	3.265750337	-0.199415686
6	-1.165449949	1.541289494	-0.199140089
6	-3.303993957	1.451755965	0.891156512
6	-2.763814091	-2.896184481	1.210036745
1	-3.962920814	-1.210061651	0.594447419
6	5.179299735	1.039624202	-0.847953540
8	0.019789900	0.909177820	-0.550559137
6	-1.324230976	2.888912754	-0.540692135
1	-4.058484461	0.922696150	1.492012103
6	-3.577711983	2.784712401	0.480908309
1	-3.624233783	-3.579706026	1.150068712
6	5.140869117	-0.068389745	-1.747710789
6	6.131182937	2.020191752	-1.121056357
15	0.372054733	0.565437268	-2.122670659
6	-2.587523394	3.517417191	-0.270061862
6	-0.167762771	3.629319592	-1.119211987
6	-4.820520479	3.417384670	0.782699530
8	4.145635723	-1.042915194	-1.545522594
6	5.953508057	-0.211132515	-2.871500455
1	6.207904540	2.887518508	-0.447963050
6	6.994521347	1.951211415	-2.249366630
8	-0.042070769	1.984906128	-2.852823073
7	1.977494846	0.444840301	-2.088518064
7	-0.314421573	-0.665928456	-2.858233352
6	-2.914822273	4.820555128	-0.747080343
6	0.415228585	4.761633077	-0.441765346

6	0.469017279	3.142576171	-2.260819051
1	-5.565421407	2.856486417	1.367959446
6	-5.100886544	4.694341824	0.324100202
15	2.813177119	-0.825726548	-2.451332046
6	5.710093060	-1.336370076	-3.820111847
6	6.911695852	0.827857851	-3.152780654
6	7.917162012	3.002627605	-2.532610359
16	-1.873209451	-1.087760926	-3.193117234
6	-4.146832390	5.392233262	-0.463373794
1	-2.178910541	5.364286883	-1.356775606
6	-0.096977013	5.282666089	0.783472611
6	1.614046457	5.352842728	-0.986546482
6	1.653008715	3.712517197	-2.825291963
1	-6.070337209	5.162526335	0.551159714
8	3.423793165	-0.636357289	-3.951956463
7	2.089118027	-2.339578917	-2.329559932
6	4.439033913	-1.492117473	-4.382729893
6	6.742443751	-2.260304881	-4.201245434
6	7.732460281	0.834434441	-4.319393524
1	7.976561767	3.849470429	-1.831565006
6	8.708169965	2.972886230	-3.669969886
8	-2.511641457	-1.726155576	-2.032611869
8	-1.850339736	-1.798137083	-4.486200760
1	-4.387483154	6.391813490	-0.855235796
1	-0.996836841	4.832837806	1.221772197
6	0.530265075	6.337883027	1.431992445
6	2.223887807	6.446735970	-0.302946333
6	2.190174594	4.817992589	-2.171920880
6	2.307162504	3.138939823	-4.026909644
16	2.409627911	-3.497039008	-1.090296721
6	4.116741845	-2.453096298	-5.393547464
6	6.462037701	-3.217143506	-5.243725316
6	8.019484608	-2.290253349	-3.568230721
6	8.606410664	1.882485822	-4.573393045
1	7.663097699	0.000354099	-5.029467693
1	9.407940426	3.795689849	-3.879383176
1	0.118818282	6.713466161	2.380918201
6	1.697589063	6.931328128	0.884208046
1	3.134061060	6.891493268	-0.734595425
1	3.094956777	5.292013665	-2.582243847
6	1.576164269	2.794293531	-5.181274979
6	3.707448536	2.953570137	-4.054317735
8	2.616040870	-2.840251789	0.206790824
8	1.377460332	-4.523847152	-1.277376739
6	5.164457763	-3.271487426	-5.820145532
6	2.754397771	-2.585023922	-5.964610651
6	7.495015179	-4.112222308	-5.654708457
6	8.994161125	-3.188989182	-3.978295489
1	8.220289691	-1.595680992	-2.740268397
1	9.222347636	1.867427226	-5.485014854
1	2.184262465	7.769504656	1.405092118
6	2.226610347	2.315696796	-6.327430066
1	0.485937755	2.919861519	-5.198858998

6	4.347836458	2.473127990	-5.200533280
1	4.301015054	3.165711045	-3.151965711
1	4.981668324	-3.992011371	-6.630571363
6	2.275343699	-3.860772340	-6.345129642
6	1.899159072	-1.479609025	-6.176240380
1	7.275344486	-4.834331938	-6.455711596
6	8.738525455	-4.092521587	-5.043938532
1	9.969673077	-3.205374235	-3.469999985
6	3.626359839	2.154575778	-6.373122396
1	1.613893209	2.076190285	-7.206998483
1	5.440220657	2.343530615	-5.173987827
6	1.007595913	-4.015746264	-6.910019707
1	2.893853779	-4.750332150	-6.155957518
6	0.626910309	-1.648307009	-6.737189505
1	2.222187253	-0.466317280	-5.906662806
1	9.521299412	-4.796482088	-5.363098179
6	0.146885741	-2.915187108	-7.121431722
1	0.672564157	-5.029701311	-7.176066575
1	-0.005191104	-0.759824429	-6.856799078
6	-2.699083321	2.650341554	-4.674879063
6	-3.751073783	3.106394593	-3.863474020
6	-2.205623574	1.351196116	-4.501501032
6	-4.310846199	2.252544149	-2.900903703
6	-2.701349068	0.512435366	-3.487216565
6	-3.786798235	0.961068274	-2.710645567
6	4.001578187	-4.916294394	-2.844305476
6	3.989824863	-4.143322012	-1.664511149
6	5.186727135	-5.506922585	-3.302981600
6	5.193575026	-3.954082592	-0.953798785
6	6.372250997	-5.346836671	-2.567198691
6	6.383460480	-4.541442726	-1.417375874
9	5.270945417	-3.214524136	0.147584353
9	7.525752419	-4.346772305	-0.758647576
9	7.492344553	-5.933033701	-2.973759776
9	5.191529433	-6.213606415	-4.434773127
9	2.895955191	-5.082961940	-3.563148434
9	-4.220468235	4.344270823	-4.008454190
9	-4.357828705	0.209882539	-1.771210035
9	-1.194498407	0.959965938	-5.283452255
9	-5.345301984	2.671429886	-2.170492014
9	-2.155824954	3.462911779	-5.588897681
6	-1.284851616	-4.908303869	1.763009379
6	-2.241106980	-5.397062282	2.873026072
1	-3.303757568	-5.223230253	2.611555245
1	-2.112142125	-6.486626178	3.039971929
1	-2.038385247	-4.875397036	3.830609233
6	-1.595106462	-5.647525540	0.439585469
1	-0.913825874	-5.307064353	-0.364840825
1	-1.463715224	-6.742352165	0.569027339
1	-2.636008708	-5.465835308	0.105642709
6	0.158779941	-5.242409389	2.179128179
1	0.885915942	-4.961840390	1.393028622
1	0.444795149	-4.728784200	3.119547097

1	0.254404376	-6.334007165	2.349401180
6	4.372480374	1.697939936	-7.639108852
6	5.306373468	2.844074498	-8.093617011
1	5.853948450	2.554220644	-9.014535171
1	4.728493386	3.765242271	-8.310295376
1	6.056832770	3.089925870	-7.315682550
6	3.409176492	1.359161383	-8.790025993
1	3.986783615	1.026230790	-9.675794201
1	2.715797118	0.538207280	-8.516171130
1	2.805886547	2.237073577	-9.097239638
6	5.219017700	0.443549021	-7.333281734
1	5.929172346	0.617680318	-6.501943779
1	4.576326572	-0.413519152	-7.056214334
1	5.808472783	0.150476712	-8.226571898
6	-1.241883981	-3.131671352	-7.741400810
6	-1.067480459	-3.731087379	-9.155959906
1	-0.533924752	-4.702489579	-9.130551935
1	-2.058768589	-3.903819881	-9.624122271
1	-0.491869985	-3.045733077	-9.811253616
6	-2.031637306	-1.815849650	-7.856226374
1	-2.185833973	-1.356009204	-6.861281167
1	-1.517993869	-1.084074934	-8.512953809
1	-3.027684362	-2.017772796	-8.299615018
6	-2.047832712	-4.110448881	-6.855436326
1	-1.549255245	-5.097174748	-6.774353693
1	-2.172909812	-3.695586345	-5.836841656
1	-3.052999568	-4.279543703	-7.294730524
6	1.440088934	1.788943901	3.631150353
6	2.092688500	2.857292583	4.540049322
1	1.452214906	3.053173722	5.425030770
1	3.086174416	2.520788827	4.899845893
1	2.232657429	3.817210555	4.003857479
6	1.206376393	0.513695464	4.459618447
1	0.729610517	-0.286354439	3.858309266
1	2.150556680	0.116436430	4.883930183
1	0.529114002	0.739103536	5.307845332
6	0.071873927	2.308051773	3.139850803
1	0.174645517	3.224117667	2.527441670
1	-0.448681218	1.548555265	2.526747354
1	-0.580302879	2.549301144	4.004549912
1	1.082680567	-2.311389436	-2.607480899

Concerted Pathway

(a) Major Stereoisomer (TS-A1)

Imaginary frequency = - 69.4

6	-1.913604000	-3.451196000	-2.588372000
6	-1.340967000	-3.062968000	-3.909849000
6	-1.128232000	-4.282153000	-4.816637000
6	-0.196278000	-5.255601000	-4.114645000

8	-0.458466000	-5.262842000	-2.774758000
1	-2.047120000	-2.328120000	-4.355942000
1	-0.670502000	-3.994038000	-5.779152000
6	-3.203587000	-4.136093000	-2.547838000
6	-4.180867000	-3.903798000	-3.550478000
6	-3.508932000	-5.016922000	-1.480864000
6	-5.427017000	-4.532244000	-3.474909000
1	-3.982818000	-3.170333000	-4.343484000
6	-4.743833000	-5.668156000	-1.430851000
1	-2.745787000	-5.220050000	-0.719021000
6	-5.707928000	-5.422453000	-2.424016000
1	-6.190605000	-4.320269000	-4.237777000
1	-4.958473000	-6.370881000	-0.612010000
1	-6.686656000	-5.923754000	-2.376292000
6	-1.353170000	-2.900082000	-1.369925000
1	-1.781953000	-3.273137000	-0.426859000
1	-0.249122000	-2.874980000	-1.382391000
1	-0.367415000	2.381013000	4.745809000
6	0.170724000	2.235121000	3.786440000
6	1.565700000	1.624109000	4.045104000
1	0.238806000	3.221011000	3.286695000
1	-0.445913000	1.575495000	3.146130000
6	2.321802000	1.452600000	2.716281000
6	1.380778000	0.287925000	4.785575000
6	2.381083000	2.581511000	4.945837000
6	-0.360747000	-0.970930000	1.553411000
6	2.882986000	0.226522000	2.304286000
6	2.505964000	2.561179000	1.860641000
1	2.352339000	-0.174778000	5.052413000
1	0.823924000	0.457514000	5.729222000
1	0.802833000	-0.439669000	4.180994000
1	1.866153000	2.731910000	5.917683000
1	3.391131000	2.171500000	5.149117000
1	2.508453000	3.574816000	4.470548000
6	-1.681855000	-0.501738000	1.377637000
6	-0.097456000	-2.286331000	1.951119000
1	0.490654000	-0.302024000	1.385565000
6	3.612051000	0.108596000	1.112570000
1	2.759220000	-0.673259000	2.922008000
6	3.239579000	2.457094000	0.675978000
1	2.066033000	3.536025000	2.117014000
6	-1.961811000	0.912119000	1.017628000
6	-2.727696000	-1.425505000	1.616701000
6	-1.133072000	-3.204968000	2.205587000
1	0.952614000	-2.590825000	2.035721000
6	3.825014000	1.232003000	0.286036000
1	4.026139000	-0.869361000	0.836344000
1	3.340976000	3.339460000	0.026963000
6	-1.062457000	1.676096000	0.205326000
6	-3.090321000	1.580092000	1.487364000
6	-2.457085000	-2.737500000	2.028101000
1	-3.771570000	-1.116095000	1.455947000
6	-0.850605000	-4.651586000	2.646072000

6	4.687704000	1.175821000	-0.918804000
8	0.026389000	1.017389000	-0.355819000
6	-1.209026000	3.040983000	-0.054996000
1	-3.797154000	1.049422000	2.142701000
6	-3.370686000	2.931697000	1.148148000
1	-3.306592000	-3.417628000	2.198682000
6	-1.417122000	-5.633665000	1.596220000
6	-1.531442000	-4.905566000	4.009450000
6	0.658395000	-4.925311000	2.782830000
6	4.651171000	0.082594000	-1.839757000
6	5.586611000	2.202014000	-1.203156000
15	-0.013092000	0.735399000	-1.980911000
6	-2.425174000	3.683008000	0.360227000
6	-0.094804000	3.777404000	-0.721405000
6	-4.574373000	3.568345000	1.572747000
1	-0.918512000	-5.488950000	0.615889000
1	-1.239715000	-6.682350000	1.912190000
1	-2.510744000	-5.505303000	1.464751000
1	-1.341567000	-5.944494000	4.350378000
1	-1.138591000	-4.212613000	4.780619000
1	-2.629465000	-4.762905000	3.955797000
1	0.819377000	-5.976945000	3.095097000
1	1.195528000	-4.767793000	1.826529000
1	1.124143000	-4.274252000	3.549686000
8	3.750888000	-0.946996000	-1.597722000
6	5.464065000	-0.015458000	-2.970073000
1	5.662405000	3.051187000	-0.507189000
6	6.413159000	2.192755000	-2.359967000
8	-0.217082000	2.262627000	-2.567715000
7	1.416577000	0.165476000	-2.305114000
7	-1.258779000	-0.118254000	-2.532046000
6	-2.751549000	5.019376000	-0.010434000
6	0.571705000	4.876798000	-0.068772000
6	0.407420000	3.337888000	-1.947963000
1	-5.286168000	2.990849000	2.182932000
6	-4.855786000	4.876795000	1.211807000
15	2.382133000	-1.053829000	-2.513711000
6	5.332574000	-1.182388000	-3.887748000
6	6.359715000	1.073904000	-3.269566000
6	7.277189000	3.289553000	-2.655513000
16	-2.770058000	0.404694000	-2.867934000
1	-1.605731000	-1.803060000	-1.478104000
6	-3.941066000	5.601961000	0.402942000
1	-2.052761000	5.579576000	-0.647503000
6	0.200999000	5.352131000	1.224597000
6	1.702675000	5.491460000	-0.722701000
6	1.537137000	3.914568000	-2.609266000
1	-5.793330000	5.351200000	1.538775000
8	2.971896000	-0.788511000	-4.032248000
7	1.858196000	-2.577732000	-2.339381000
6	4.091118000	-1.488760000	-4.457789000
6	6.467623000	-1.991064000	-4.243835000
6	7.157108000	1.126731000	-4.451526000

1	7.311572000	4.133177000	-1.948605000
6	8.044419000	3.305708000	-3.809607000
8	-3.400365000	1.199996000	-1.799027000
8	-3.507489000	-0.768906000	-3.398014000
1	-4.180450000	6.631221000	0.095737000
1	-0.639248000	4.877332000	1.747355000
6	0.890817000	6.392073000	1.832193000
6	2.380546000	6.567857000	-0.075561000
6	2.149912000	4.994514000	-1.978160000
6	2.058960000	3.371149000	-3.888123000
16	2.411724000	-3.591976000	-1.204949000
6	3.919118000	-2.449414000	-5.505692000
6	6.319773000	-2.975285000	-5.286433000
6	7.726068000	-1.881993000	-3.581659000
6	7.976151000	2.215739000	-4.716783000
1	7.109809000	0.295866000	-5.167037000
1	8.699759000	4.162232000	-4.027737000
1	0.587331000	6.733241000	2.833429000
6	1.985861000	7.013240000	1.175945000
1	3.236708000	7.031366000	-0.590151000
1	3.010211000	5.480293000	-2.463284000
6	1.212864000	2.995195000	-4.951110000
6	3.450160000	3.238991000	-4.087849000
8	2.518891000	-3.011791000	0.149384000
8	1.613645000	-4.860230000	-1.300348000
6	5.054070000	-3.154457000	-5.906244000
6	2.629513000	-2.636541000	-6.216443000
6	7.451748000	-3.755829000	-5.668378000
6	8.800870000	-2.672575000	-3.961971000
1	7.830524000	-1.170690000	-2.750517000
1	8.572770000	2.234327000	-5.641245000
1	2.523170000	7.839505000	1.665163000
6	1.733958000	2.535545000	-6.168418000
1	0.126937000	3.080514000	-4.829398000
6	3.965266000	2.774106000	-5.301544000
1	4.141003000	3.479228000	-3.267027000
1	4.974027000	-3.869478000	-6.738032000
6	2.270920000	-3.903097000	-6.722417000
6	1.772770000	-1.548872000	-6.513818000
1	7.329519000	-4.499883000	-6.470003000
6	8.670494000	-3.602029000	-5.028000000
1	9.758140000	-2.585183000	-3.426945000
6	3.123311000	2.420527000	-6.379420000
1	1.030332000	2.278262000	-6.972031000
1	5.056895000	2.684191000	-5.401878000
6	1.145861000	-4.066846000	-7.542922000
1	2.864991000	-4.785655000	-6.445382000
6	0.653438000	-1.721444000	-7.332535000
1	1.993861000	-0.548836000	-6.118210000
1	9.531162000	-4.220526000	-5.322644000
6	3.733296000	1.980118000	-7.721129000
6	0.318334000	-2.977611000	-7.885478000
1	0.909111000	-5.079227000	-7.895584000

1	0.029282000	-0.843518000	-7.548748000
6	-1.795555000	1.771549000	-6.627653000
6	4.535640000	3.168601000	-8.301768000
6	2.657719000	1.569714000	-8.742510000
6	4.686758000	0.784202000	-7.502448000
6	-0.891451000	-3.101351000	-8.827920000
6	-2.023074000	3.154897000	-6.554619000
6	-2.030419000	0.967700000	-5.504773000
1	4.992593000	2.888923000	-9.273840000
1	3.879695000	4.047074000	-8.467824000
1	5.351668000	3.479472000	-7.619149000
1	3.141441000	1.261594000	-9.691188000
1	2.054236000	0.711852000	-8.385369000
1	1.968739000	2.406022000	-8.977249000
1	5.505870000	1.035563000	-6.801153000
1	4.148938000	-0.092188000	-7.092612000
1	5.147787000	0.480387000	-8.464835000
6	-0.614527000	-2.248004000	-10.088145000
6	-2.167488000	-2.571762000	-8.133927000
6	-1.139916000	-4.556297000	-9.260155000
6	-2.493539000	3.719732000	-5.358013000
6	-2.512184000	1.514132000	-4.302065000
1	-1.463844000	-2.318729000	-10.799212000
1	-0.475813000	-1.179164000	-9.830172000
1	0.301931000	-2.593935000	-10.607396000
1	-3.034008000	-2.643100000	-8.823253000
1	-2.406347000	-3.162569000	-7.226589000
1	-2.065510000	-1.513722000	-7.828453000
1	-0.275425000	-4.977199000	-9.811595000
1	-1.345537000	-5.214119000	-8.391178000
1	-2.019773000	-4.604361000	-9.932925000
6	-2.725081000	2.903883000	-4.233427000
6	4.210396000	-4.838209000	-2.914073000
6	4.080008000	-4.076909000	-1.735686000
6	5.455861000	-5.338545000	-3.318055000
6	5.237029000	-3.771984000	-0.993311000
6	6.595850000	-5.068153000	-2.544641000
6	6.491815000	-4.254263000	-1.406711000
1	0.403004000	-5.266522000	-2.221167000
1	-2.085924000	-4.798836000	-5.039687000
1	-0.387922000	-2.524479000	-3.756991000
8	0.665759000	-5.925019000	-4.639896000
9	-1.772981000	3.925872000	-7.610924000
9	-1.312067000	1.237850000	-7.761427000
9	-1.760199000	-0.338830000	-5.615567000
9	-3.121556000	3.512329000	-3.120329000
9	-2.695627000	5.036622000	-5.283399000
9	5.211881000	-3.021104000	0.106567000
9	7.589737000	-3.949062000	-0.710576000
9	7.780772000	-5.557289000	-2.903872000
9	5.561573000	-6.055178000	-4.440580000
9	3.154326000	-5.096337000	-3.682536000

(b) Minor Stereoisomer (TS-ent-A1)

Imaginary frequency = - 134.25

6	-2.591333000	-3.329542000	-0.405293000
6	-2.092968000	-2.782983000	0.908379000
6	-2.013641000	-3.894525000	1.968900000
6	-2.059043000	-5.251730000	1.307891000
8	-1.665409000	-5.174995000	-0.037121000
1	-1.115698000	-2.301824000	0.747295000
1	-2.820698000	-3.842052000	2.718309000
6	-3.976807000	-3.812265000	-0.482000000
6	-4.385825000	-4.657331000	-1.543115000
6	-4.934397000	-3.419300000	0.485247000
6	-5.707966000	-5.097165000	-1.632126000
1	-3.643366000	-5.000199000	-2.277115000
6	-6.261331000	-3.846553000	0.380612000
1	-4.658623000	-2.735726000	1.299655000
6	-6.651499000	-4.687373000	-0.673219000
1	-6.008382000	-5.765878000	-2.452202000
1	-6.997350000	-3.510884000	1.126035000
1	-7.695852000	-5.026113000	-0.749967000
6	-1.892560000	-2.928733000	-1.638550000
1	-0.792415000	-2.915547000	-1.528759000
1	-2.223292000	-3.468303000	-2.540428000
1	-2.179710000	2.133086000	7.238728000
6	-1.551707000	2.113787000	6.324175000
6	-0.227325000	1.365269000	6.599097000
1	-1.374063000	3.163317000	6.016861000
1	-2.135829000	1.625033000	5.520725000
6	0.647947000	1.365299000	5.334029000
6	-0.553374000	-0.057327000	7.085241000
6	0.544276000	2.096627000	7.723368000
6	-2.789808000	-0.692832000	4.011416000
6	1.160132000	0.195596000	4.736993000
6	1.008111000	2.593320000	4.738186000
1	0.364334000	-0.626127000	7.336597000
1	-1.176430000	-0.007081000	8.000928000
1	-1.123482000	-0.632586000	6.330256000
1	-0.058876000	2.119570000	8.654873000
1	1.502666000	1.584527000	7.944140000
1	0.777326000	3.143300000	7.443619000
6	-3.875751000	0.041635000	3.489472000
6	-2.972702000	-1.989399000	4.517199000
1	-1.791511000	-0.235396000	4.054641000
6	2.015190000	0.241856000	3.624571000
1	0.906257000	-0.792722000	5.144934000
6	1.855681000	2.650261000	3.631201000
1	0.617021000	3.539179000	5.139606000
6	-3.776023000	1.483685000	3.155901000
6	-5.122792000	-0.618667000	3.397781000
6	-4.228697000	-2.630232000	4.491133000

1	-2.102113000	-2.503275000	4.947858000
6	2.398580000	1.477403000	3.057047000
1	2.391611000	-0.701792000	3.212160000
1	2.081612000	3.629825000	3.188020000
6	-2.650786000	2.056310000	2.482649000
6	-4.799402000	2.348553000	3.532861000
6	-5.290024000	-1.919071000	3.885038000
1	-5.965893000	-0.098763000	2.920507000
6	-4.485638000	-4.009460000	5.127024000
6	3.350807000	1.608159000	1.924512000
8	-1.643866000	1.196299000	2.061489000
6	-2.511066000	3.430414000	2.266791000
1	-5.665027000	1.945197000	4.079264000
6	-4.776763000	3.735355000	3.227051000
1	-6.284299000	-2.385711000	3.805900000
6	-5.132405000	-4.974076000	4.106285000
6	-5.451181000	-3.806097000	6.319280000
6	-3.193783000	-4.660543000	5.652810000
6	3.423990000	0.667866000	0.848058000
6	4.210290000	2.704884000	1.838921000
15	-1.320833000	0.966639000	0.454811000
6	-3.622732000	4.293577000	2.571362000
6	-1.241718000	3.987201000	1.725173000
6	-5.876485000	4.586072000	3.544752000
1	-4.438883000	-5.225151000	3.278974000
1	-5.401095000	-5.927339000	4.605784000
1	-6.059767000	-4.557386000	3.666120000
1	-5.657112000	-4.774870000	6.819914000
1	-5.014670000	-3.114686000	7.068121000
1	-6.420201000	-3.379637000	5.991337000
1	-3.431003000	-5.641597000	6.110617000
1	-2.463212000	-4.848334000	4.840739000
1	-2.703071000	-4.040889000	6.429869000
8	2.608048000	-0.454751000	0.891827000
6	4.261661000	0.805280000	-0.262108000
1	4.219240000	3.440828000	2.656474000
6	5.072126000	2.922612000	0.730306000
8	-1.434132000	2.508615000	-0.139193000
7	0.173036000	0.455707000	0.476434000
7	-2.341516000	0.062826000	-0.386799000
6	-3.649354000	5.671363000	2.206428000
6	-0.490392000	4.980032000	2.449376000
6	-0.737383000	3.524707000	0.509723000
1	-6.747171000	4.148636000	4.057661000
6	-5.862124000	5.928161000	3.198373000
15	1.271436000	-0.516838000	-0.070197000
6	4.209623000	-0.212353000	-1.351126000
6	5.096644000	1.973379000	-0.356164000
6	5.887457000	4.091748000	0.649056000
16	-3.923526000	0.337658000	-0.681686000
6	-4.743660000	6.468575000	2.510597000
1	-2.795120000	6.096423000	1.661764000
6	-0.859139000	5.435593000	3.749570000

6	0.716265000	5.502834000	1.857584000
6	0.445829000	4.039368000	-0.109766000
1	-6.720995000	6.572048000	3.440586000
8	1.836598000	0.110001000	-1.485527000
7	0.983175000	-2.092024000	-0.310771000
6	2.990299000	-0.502076000	-1.974701000
6	5.383299000	-0.927982000	-1.775359000
6	5.904204000	2.266211000	-1.495117000
1	5.867020000	4.803226000	1.489339000
6	6.673067000	4.339044000	-0.465460000
8	-4.685209000	0.800640000	0.497596000
8	-4.432183000	-0.830582000	-1.431969000
1	-4.747007000	7.526841000	2.208788000
1	-1.759638000	5.022566000	4.223413000
6	-0.088217000	6.371125000	4.425390000
6	1.479094000	6.474035000	2.573344000
6	1.138147000	5.028709000	0.586601000
6	0.917739000	3.589660000	-1.441654000
16	1.428093000	-3.270568000	0.679824000
6	2.854960000	-1.418576000	-3.065139000
6	5.280523000	-1.830792000	-2.895176000
6	6.638411000	-0.813221000	-1.106453000
6	6.671784000	3.421347000	-1.549368000
1	5.906129000	1.567682000	-2.342707000
1	7.289962000	5.248686000	-0.517581000
1	-0.388822000	6.699044000	5.431948000
6	1.088122000	6.902007000	3.832360000
1	2.395055000	6.868486000	2.106163000
1	2.039985000	5.468229000	0.134744000
6	0.031157000	3.330977000	-2.507637000
6	2.300373000	3.491205000	-1.708999000
8	1.261866000	-2.980348000	2.122583000
8	0.773199000	-4.532218000	0.183276000
6	4.017961000	-2.044130000	-3.512167000
6	1.553302000	-1.671320000	-3.731673000
6	6.450688000	-2.513110000	-3.345099000
6	7.751690000	-1.507775000	-1.556496000
1	6.712041000	-0.171876000	-0.217450000
1	7.280971000	3.628794000	-2.442005000
1	1.689271000	7.646059000	4.376261000
6	0.506458000	3.026727000	-3.791098000
1	-1.050439000	3.393158000	-2.337044000
6	2.768140000	3.177112000	-2.987129000
1	3.023420000	3.647503000	-0.896215000
1	3.960414000	-2.725911000	-4.373866000
6	1.197000000	-2.972181000	-4.150630000
6	0.668550000	-0.619116000	-4.047669000
1	6.363204000	-3.191228000	-4.207336000
6	7.664130000	-2.349297000	-2.697402000
1	8.706597000	-1.414518000	-1.018287000
6	1.887571000	2.948894000	-4.067183000
1	-0.229076000	2.862764000	-4.590492000
1	3.855543000	3.113348000	-3.138677000

6	0.026453000	-3.197832000	-4.884713000
1	1.840705000	-3.820961000	-3.880230000
6	-0.483134000	-0.850462000	-4.810427000
1	0.895270000	0.404446000	-3.721905000
1	8.555006000	-2.891497000	-3.047272000
6	2.450605000	2.679788000	-5.473127000
6	-0.832738000	-2.140568000	-5.259096000
1	-0.212611000	-4.230383000	-5.180028000
1	-1.117871000	0.008498000	-5.058726000
6	-3.162727000	2.520526000	-4.076366000
6	3.165933000	3.963068000	-5.957894000
6	1.345174000	2.321951000	-6.482376000
6	3.467262000	1.516398000	-5.427519000
6	-2.099114000	-2.405239000	-6.094684000
6	-3.433040000	3.845213000	-3.695537000
6	-3.368616000	1.479015000	-3.162518000
1	3.591028000	3.810699000	-6.971858000
1	2.460014000	4.816935000	-6.003262000
1	3.995135000	4.246281000	-5.279006000
1	1.794969000	2.128241000	-7.476924000
1	0.793880000	1.409073000	-6.181434000
1	0.613292000	3.145500000	-6.604838000
1	4.312366000	1.728448000	-4.742819000
1	2.989209000	0.576106000	-5.091193000
1	3.892301000	1.341388000	-6.437237000
6	-1.787527000	-3.396031000	-7.237535000
6	-2.648137000	-1.110609000	-6.724634000
6	-3.189274000	-3.010188000	-5.178333000
6	-3.919818000	4.112556000	-2.405351000
6	-3.853214000	1.728488000	-1.866745000
1	-2.691616000	-3.554538000	-7.860376000
1	-0.981856000	-3.008193000	-7.893168000
1	-1.471037000	-4.388612000	-6.860966000
1	-3.519975000	-1.348582000	-7.366982000
1	-2.987765000	-0.384134000	-5.963373000
1	-1.885573000	-0.613488000	-7.358000000
1	-2.848793000	-3.967642000	-4.732457000
1	-3.453905000	-2.318197000	-4.354426000
1	-4.111363000	-3.219978000	-5.759667000
6	-4.121831000	3.058347000	-1.494647000
6	3.566574000	-4.213447000	-0.837729000
6	3.188205000	-3.567122000	0.356596000
6	4.910688000	-4.504666000	-1.109759000
6	4.197221000	-3.161294000	1.252693000
6	5.904910000	-4.112777000	-0.200208000
6	5.549661000	-3.419299000	0.966503000
1	-0.669392000	-4.858250000	-0.036070000
1	-1.052829000	-3.811797000	2.522338000
1	-2.770936000	-1.967342000	1.231897000
9	3.933066000	-2.527348000	2.392594000
9	6.500805000	-3.015441000	1.810773000
9	7.183540000	-4.390493000	-0.443869000
9	5.243420000	-5.135964000	-2.238588000

9	2.664137000	-4.575432000	-1.750626000
9	-3.059397000	0.239459000	-3.557680000
9	-4.548488000	3.385413000	-0.278490000
9	-4.171059000	5.371320000	-2.039275000
9	-3.221980000	4.840731000	-4.554353000
9	-2.667494000	2.265765000	-5.297842000
8	-2.441018000	-6.287209000	1.789152000
1	-2.197264000	-1.859423000	-1.760051000

Pre-reaction complex for the concerted cycloaddition

6	-1.901693000	-3.269473000	-2.956163000
6	-1.084649000	-3.568414000	-4.191570000
6	-0.994775000	-5.085600000	-4.511738000
6	0.184415000	-5.705571000	-3.785221000
8	0.027562000	-5.635293000	-2.451318000
1	-1.520362000	-3.054277000	-5.068903000
1	-0.833569000	-5.243139000	-5.593214000
6	-3.377568000	-3.428558000	-3.036384000
6	-4.071907000	-3.286876000	-4.259704000
6	-4.136673000	-3.716055000	-1.877670000
6	-5.466795000	-3.405644000	-4.318736000
1	-3.518916000	-3.049134000	-5.178308000
6	-5.529249000	-3.836344000	-1.935017000
1	-3.616125000	-3.865368000	-0.921991000
6	-6.203929000	-3.678817000	-3.156984000
1	-5.982664000	-3.279221000	-5.282959000
1	-6.094238000	-4.065367000	-1.018265000
1	-7.299364000	-3.775047000	-3.203578000
6	-1.285695000	-2.902215000	-1.799487000
1	-1.850275000	-2.701286000	-0.877189000
1	-0.186689000	-2.878091000	-1.743891000
1	-0.293391000	2.250018000	5.047319000
6	0.226466000	2.173253000	4.070248000
6	1.596314000	1.482129000	4.247732000
1	0.327282000	3.199803000	3.665580000
1	-0.424255000	1.602093000	3.380616000
6	2.326397000	1.398064000	2.896743000
6	1.359014000	0.092716000	4.865445000
6	2.467550000	2.315911000	5.216352000
6	-0.357551000	-1.178515000	1.530777000
6	2.874197000	0.201273000	2.392887000
6	2.508944000	2.561057000	2.116920000
1	2.310838000	-0.431395000	5.084341000
1	0.811308000	0.197499000	5.823666000
1	0.752415000	-0.553973000	4.199756000
1	1.972578000	2.403572000	6.206055000
1	3.458669000	1.841918000	5.365992000
1	2.638163000	3.340141000	4.827882000
6	-1.625490000	-0.567547000	1.403833000
6	-0.227825000	-2.563854000	1.694664000
1	0.554364000	-0.569899000	1.503477000
6	3.579135000	0.160036000	1.181880000

1	2.763859000	-0.738380000	2.950701000
6	3.209735000	2.532019000	0.908584000
1	2.093000000	3.521691000	2.452718000
6	-1.786827000	0.897268000	1.220832000
6	-2.759382000	-1.412755000	1.458724000
6	-1.352954000	-3.409210000	1.757907000
1	0.787796000	-2.977622000	1.744158000
6	3.771313000	1.331627000	0.417731000
1	3.984791000	-0.800931000	0.844914000
1	3.305437000	3.457623000	0.322627000
6	-0.868071000	1.663838000	0.437951000
6	-2.856965000	1.599020000	1.772398000
6	-2.621596000	-2.793713000	1.642995000
1	-3.763411000	-0.983759000	1.319412000
6	-1.233306000	-4.931882000	1.932419000
6	4.586944000	1.351411000	-0.822037000
8	0.184126000	0.969097000	-0.171927000
6	-0.977707000	3.033789000	0.193941000
1	-3.573697000	1.066053000	2.414970000
6	-3.073036000	2.980086000	1.515690000
1	-3.532631000	-3.411196000	1.674440000
6	-1.878980000	-5.646103000	0.722553000
6	-1.970887000	-5.342225000	3.227667000
6	0.234027000	-5.384970000	2.035669000
6	4.573853000	0.278870000	-1.770465000
6	5.425894000	2.427181000	-1.108641000
15	0.009735000	0.657893000	-1.760844000
6	-2.143031000	3.713765000	0.692404000
6	0.083560000	3.728305000	-0.591511000
6	-4.220153000	3.653862000	2.031121000
1	-1.375326000	-5.378705000	-0.227798000
1	-1.803561000	-6.745964000	0.847755000
1	-2.955908000	-5.400441000	0.625554000
1	-1.902486000	-6.439591000	3.377148000
1	-1.524848000	-4.845268000	4.113111000
1	-3.046054000	-5.073899000	3.194185000
1	0.272767000	-6.485189000	2.166811000
1	0.816201000	-5.138014000	1.126300000
1	0.744352000	-4.927688000	2.907447000
8	3.748332000	-0.802366000	-1.498346000
6	5.369771000	0.239017000	-2.919129000
1	5.489729000	3.259568000	-0.391893000
6	6.213041000	2.487177000	-2.290093000
8	-0.223211000	2.151331000	-2.380750000
7	1.218992000	-0.160902000	-2.251286000
7	-1.456600000	-0.019333000	-2.170643000
6	-2.445674000	5.066177000	0.361411000
6	0.807021000	4.852088000	-0.050404000
6	0.464167000	3.248745000	-1.848012000
1	-4.916271000	3.089359000	2.670566000
6	-4.469826000	4.980231000	1.717036000
15	2.431518000	-1.166952000	-2.414814000
6	5.331793000	-0.940403000	-3.831146000

6	6.187356000	1.387269000	-3.223416000
6	7.012263000	3.632890000	-2.584222000
16	-2.958019000	0.737612000	-2.505745000
1	-1.496963000	-1.080816000	-2.237418000
6	-3.582701000	5.684718000	0.860880000
1	-1.771439000	5.614669000	-0.310559000
6	0.570679000	5.369702000	1.257837000
6	1.855007000	5.450979000	-0.841325000
6	1.523969000	3.794029000	-2.638078000
1	-5.363844000	5.483913000	2.114293000
8	2.943575000	-0.778189000	-3.926169000
7	2.137283000	-2.728563000	-2.214461000
6	4.117975000	-1.378778000	-4.369007000
6	6.531288000	-1.646351000	-4.200798000
6	6.938375000	1.510460000	-4.430414000
1	7.028595000	4.459503000	-1.856838000
6	7.739954000	3.714331000	-3.760747000
8	-3.238708000	1.783389000	-1.520525000
8	-3.881750000	-0.373393000	-2.755911000
1	-3.801882000	6.727263000	0.585313000
1	-0.198212000	4.902465000	1.886640000
6	1.304212000	6.440676000	1.748148000
6	2.580843000	6.560672000	-0.312521000
6	2.181269000	4.904991000	-2.111880000
6	1.947173000	3.206179000	-3.935144000
16	2.809348000	-3.627401000	-1.031091000
6	4.002839000	-2.395135000	-5.368064000
6	6.448196000	-2.673255000	-5.210003000
6	7.792766000	-1.399532000	-3.582647000
6	7.692811000	2.645300000	-4.694164000
1	6.904827000	0.696564000	-5.166467000
1	8.346634000	4.606419000	-3.977057000
1	1.105450000	6.815610000	2.763376000
6	2.312092000	7.050374000	0.955245000
1	3.371349000	7.011919000	-0.932052000
1	2.985480000	5.376010000	-2.696665000
6	1.035785000	2.729402000	-4.900367000
6	3.320563000	3.147768000	-4.260937000
8	2.836970000	-2.954081000	0.285360000
8	2.205456000	-4.985681000	-1.074024000
6	5.188264000	-3.004082000	-5.778511000
6	2.708453000	-2.737493000	-6.008131000
6	7.639930000	-3.349101000	-5.610308000
6	8.928402000	-2.089758000	-3.980669000
1	7.853991000	-0.663130000	-2.769680000
1	8.254958000	2.717018000	-5.637452000
1	2.883714000	7.902106000	1.353225000
6	1.474058000	2.250735000	-6.142965000
1	-0.037677000	2.753653000	-4.685157000
6	3.751287000	2.666333000	-5.499910000
1	4.068587000	3.464853000	-3.520739000
1	5.152360000	-3.763082000	-6.573760000
6	2.438028000	-4.060351000	-6.411868000

6	1.747291000	-1.747831000	-6.328084000
1	7.566172000	-4.125848000	-6.386280000
6	8.857511000	-3.056634000	-5.018411000
1	9.887775000	-1.895658000	-3.478644000
6	2.841684000	2.216608000	-6.481566000
1	0.722225000	1.913008000	-6.869055000
1	4.832539000	2.641583000	-5.697266000
6	1.305686000	-4.368050000	-7.179261000
1	3.107652000	-4.872996000	-6.097601000
6	0.619296000	-2.066065000	-7.088443000
1	1.894320000	-0.710211000	-6.000614000
1	9.765435000	-3.595429000	-5.327203000
6	3.356230000	1.765283000	-7.858285000
6	0.385320000	-3.374700000	-7.567243000
1	1.147607000	-5.417487000	-7.459381000
1	-0.088759000	-1.260675000	-7.328751000
6	-1.999922000	1.311903000	-6.447266000
6	4.018983000	2.981711000	-8.547430000
6	2.222555000	1.247788000	-8.761350000
6	4.405267000	0.642977000	-7.688522000
6	-0.801945000	-3.644309000	-8.508931000
6	-2.117778000	2.701516000	-6.616421000
6	-2.251193000	0.737836000	-5.194432000
1	4.405486000	2.695221000	-9.547447000
1	3.291389000	3.807679000	-8.681433000
1	4.869211000	3.372394000	-7.953386000
1	2.637338000	0.938212000	-9.741477000
1	1.716595000	0.364729000	-8.323797000
1	1.458944000	2.028006000	-8.956171000
1	5.263511000	0.971220000	-7.069700000
1	3.965936000	-0.252175000	-7.208147000
1	4.802018000	0.337496000	-8.678360000
6	-0.603632000	-2.793637000	-9.786433000
6	-2.134254000	-3.240057000	-7.838547000
6	-0.893457000	-5.125068000	-8.914398000
6	-2.482892000	3.511907000	-5.529725000
6	-2.613364000	1.539947000	-4.093797000
1	-1.426349000	-2.979759000	-10.507857000
1	-0.593429000	-1.709656000	-9.555628000
1	0.355681000	-3.043264000	-10.282938000
1	-2.973697000	-3.352240000	-8.555414000
1	-2.351206000	-3.887797000	-6.966339000
1	-2.124924000	-2.187540000	-7.493757000
1	0.015747000	-5.461978000	-9.451214000
1	-1.035870000	-5.784345000	-8.034299000
1	-1.758449000	-5.278574000	-9.590643000
6	-2.730413000	2.933564000	-4.269732000
6	4.739242000	-4.691181000	-2.715143000
6	4.526082000	-3.891544000	-1.573831000
6	6.034763000	-5.051985000	-3.111860000
6	5.642020000	-3.411209000	-0.864231000
6	7.138609000	-4.603729000	-2.369570000
6	6.944021000	-3.752904000	-1.271334000

1	0.921833000	-5.673224000	-2.008991000
1	-1.938613000	-5.590940000	-4.218223000
1	-0.062800000	-3.154430000	-4.080521000
8	1.178488000	-6.147743000	-4.336998000
9	-1.846876000	3.245712000	-7.798396000
9	-1.621102000	0.555576000	-7.484983000
9	-2.104974000	-0.582174000	-5.063019000
9	-3.036009000	3.757437000	-3.275328000
9	-2.578653000	4.830566000	-5.688833000
9	5.531217000	-2.613549000	0.198680000
9	8.001918000	-3.276508000	-0.608900000
9	8.373218000	-4.957694000	-2.723144000
9	6.219966000	-5.806155000	-4.199000000
9	3.718658000	-5.118648000	-3.453310000

Product complex for the concerted cycloaddition

6	-1.404335058	-3.981491901	-2.558957126
6	-1.465427341	-3.177534928	-3.869878387
6	-1.078711843	-4.215595807	-4.917432601
6	-0.074192697	-5.086752145	-4.206134560
8	-0.245519830	-4.886499062	-2.825419973
1	-2.447109059	-2.699037241	-4.019599274
1	-0.625126513	-3.810474630	-5.838766225
6	-2.645329841	-4.843032768	-2.336202732
6	-3.894030917	-4.197330471	-2.210181169
6	-2.575294657	-6.239458770	-2.182999880
6	-5.047405437	-4.943096776	-1.931430267
1	-3.960621189	-3.103325538	-2.328029963
6	-3.735275902	-6.983015283	-1.908102746
1	-1.603954681	-6.747370510	-2.266080105
6	-4.973206443	-6.337986560	-1.777933604
1	-6.014932213	-4.427329199	-1.833869960
1	-3.665209896	-8.075113134	-1.789425949
1	-5.880949079	-6.920339889	-1.558185737
6	-1.090357169	-3.183958392	-1.306254786
1	-0.936588463	-3.860377116	-0.446516940
1	-0.206470396	-2.541957980	-1.447680655
1	-0.376427501	2.329470525	4.866032932
6	0.156372008	2.204435865	3.900983764
6	1.522129333	1.520878789	4.132113815
1	0.266222326	3.209293404	3.447612829
1	-0.488197908	1.603873710	3.231312549
6	2.280133631	1.381109855	2.800692648
6	1.277442053	0.160988443	4.809395740
6	2.376493311	2.397726072	5.078388087
6	-0.678726632	-0.920614117	1.650721553
6	2.823724681	0.162096672	2.346155544
6	2.499234519	2.518826311	1.993405629
1	2.227202873	-0.353866166	5.058481850
1	0.723599054	0.310603575	5.758068393
1	0.671064252	-0.512974622	4.171780607

1	1.864202687	2.526145161	6.054679914
1	3.365928509	1.933143938	5.265131205
1	2.551301125	3.404949419	4.649749937
6	-1.904742693	-0.281888922	1.361773024
6	-0.631524208	-2.281768022	1.975728426
1	0.258404124	-0.350022264	1.621713780
6	3.576590961	0.081981085	1.165593343
1	2.674288855	-0.760186007	2.924016798
6	3.249892995	2.450419330	0.817882830
1	2.074486950	3.491317252	2.281031638
6	-1.990107161	1.169250095	1.070233426
6	-3.073277542	-1.075333939	1.382795580
6	-1.797362860	-3.073903527	2.025608643
1	0.353375103	-2.727641283	2.171320370
6	3.824338333	1.233322193	0.388206534
1	3.987162800	-0.889533507	0.864248612
1	3.374676671	3.356488256	0.207694113
6	-1.011898354	1.830861360	0.266090347
6	-3.041445399	1.947088029	1.546324938
6	-3.017707906	-2.434532490	1.714269268
1	-4.034210210	-0.621554952	1.097837731
6	-1.759036557	-4.558724757	2.428457027
6	4.698349456	1.221412664	-0.809997433
8	0.018550009	1.058677705	-0.264052510
6	-1.038453013	3.195600317	-0.028835707
1	-3.802812601	1.482667639	2.190898735
6	-3.185933871	3.318273661	1.201725794
1	-3.953098214	-3.012809642	1.707951676
6	-2.727999257	-5.395949295	1.565872888
6	-2.190311171	-4.656714001	3.911356199
6	-0.343400973	-5.152492059	2.284261630
6	4.686013645	0.160240290	-1.767209519
6	5.579470413	2.272060997	-1.065327317
15	-0.031649813	0.710521866	-1.874533823
6	-2.184893141	3.958571786	0.384955646
6	0.111748390	3.809193610	-0.750274179
6	-4.316176601	4.075766459	1.630148034
1	-2.493010334	-5.320090058	0.486359490
1	-2.662392951	-6.465267233	1.852867094
1	-3.783549596	-5.088358781	1.698278846
1	-2.178092458	-5.712915976	4.253481654
1	-1.508007018	-4.073188065	4.562142370
1	-3.215603051	-4.259997619	4.054888699
1	-0.366508703	-6.237024821	2.512946892
1	0.060815429	-5.030271004	1.258519920
1	0.377415438	-4.685499913	2.984341323
8	3.813347829	-0.905799665	-1.561624473
6	5.484290155	0.120713514	-2.910914714
1	5.642677477	3.100005618	-0.343355925
6	6.399241223	2.318557980	-2.225924618
8	-0.184319196	2.224532286	-2.521127643
7	1.415688419	0.128544546	-2.147895235
7	-1.232330776	-0.181794876	-2.428552886

6	-2.392642408	5.310689734	-0.014719868
6	0.875824194	4.883703102	-0.168066399
6	0.532816244	3.282825760	-1.974067874
1	-5.069747728	3.580623820	2.262120507
6	-4.478209058	5.397231897	1.244339700
15	2.439772422	-0.988419769	-2.470705145
6	5.368734943	-1.020818533	-3.862718764
6	6.352938119	1.237724822	-3.181185764
6	7.245828310	3.438346126	-2.484639633
16	-2.781284291	0.284849231	-2.655171296
1	-1.943016073	-2.524810752	-1.066786464
6	-3.513897997	6.013104190	0.403267468
1	-1.656757147	5.789345518	-0.675827483
6	0.592523873	5.428270787	1.120233077
6	2.014220651	5.398409849	-0.890137258
6	1.667537363	3.762111498	-2.701637825
1	-5.360578056	5.965837633	1.574642013
8	2.998825716	-0.670100138	-3.980136554
7	2.023194503	-2.573220727	-2.369689329
6	4.130534336	-1.343258980	-4.431055697
6	6.518625456	-1.798403594	-4.240144450
6	7.127132259	1.353887038	-4.373800233
1	7.277956713	4.252028247	-1.743503597
6	7.997620432	3.512680830	-3.646353180
8	-3.346398335	1.090254859	-1.555268722
8	-3.545254711	-0.894666028	-3.118839242
1	-3.660355343	7.052896128	0.073923627
1	-0.251376333	5.025607408	1.695713745
6	1.371637538	6.443915187	1.656669885
6	2.785790851	6.453096810	-0.315998289
6	2.372925465	4.824741927	-2.140188872
6	2.098884686	3.169908240	-3.992475448
16	2.595623274	-3.517912725	-1.242491729
6	3.964113590	-2.298821618	-5.484988773
6	6.380992012	-2.772693449	-5.293203884
6	7.781919986	-1.668326683	-3.590648975
6	7.927872272	2.464484521	-4.601693714
1	7.074568011	0.556213265	-5.126753080
1	8.639801543	4.385843178	-3.835577057
1	1.134505581	6.840922736	2.655239835
6	2.473406585	6.969650982	0.931324982
1	3.645927487	6.840921334	-0.883917692
1	3.236781484	5.238690559	-2.682023108
6	1.189531692	2.821760790	-5.012662555
6	3.473843392	2.997273997	-4.262135502
8	2.602810577	-3.072446654	0.155947293
8	1.826527381	-4.887682777	-1.399041287
6	5.113187436	-2.973647897	-5.901154485
6	2.674666484	-2.510003161	-6.190289721
6	7.526697793	-3.521557259	-5.698492411
6	8.870295854	-2.428698489	-3.993529900
1	7.881106672	-0.963042298	-2.753827451
1	8.508695737	2.534163949	-5.533692856

1	3.080794601	7.778479139	1.364541836
6	1.637353930	2.371623907	-6.263502880
1	0.112967825	2.930500880	-4.834376302
6	3.914069185	2.541346144	-5.507344566
1	4.211354591	3.211455060	-3.475577518
1	5.043570711	-3.679670191	-6.741308353
6	2.374304555	-3.762951146	-6.767023343
6	1.763395062	-1.451787447	-6.426513568
1	7.413389584	-4.255906321	-6.510280353
6	8.748829210	-3.347685129	-5.069942250
1	9.832216925	-2.324042763	-3.470102344
6	3.012026211	2.235641552	-6.549756309
1	0.886379881	2.146074617	-7.033493213
1	4.996710311	2.430050697	-5.664606102
6	1.267856506	-3.930576397	-7.610977319
1	3.008122323	-4.632035119	-6.538538684
6	0.656236680	-1.631529009	-7.260192894
1	1.936494012	-0.462594102	-5.984133526
1	9.620674997	-3.940395344	-5.383990192
6	3.551706269	1.838961282	-7.934799690
6	0.394263065	-2.861917193	-7.902613834
1	1.084833838	-4.928082490	-8.031750208
1	-0.014200405	-0.776574670	-7.422368320
6	-1.981098103	1.641449215	-6.461946712
6	4.304088077	3.057522516	-8.520937669
6	2.426623663	1.444989548	-8.908078450
6	4.531889372	0.650833553	-7.808387124
6	-0.780130095	-2.975571791	-8.889523404
6	-2.174410488	3.029098772	-6.374482251
6	-2.196280705	0.833263698	-5.337708189
1	4.709566440	2.815519759	-9.525433375
1	3.627284605	3.929949675	-8.622020051
1	5.151955785	3.360944042	-7.874625528
1	2.861014612	1.167548527	-9.889584180
1	1.850566441	0.572786885	-8.541429664
1	1.720137589	2.281196715	-9.083130174
1	5.382508636	0.883730803	-7.137295008
1	4.024376491	-0.248862774	-7.411130547
1	4.950851197	0.393599991	-8.802950308
6	-0.515565831	-2.002270685	-10.063289992
6	-2.106392165	-2.582368824	-8.199594669
6	-0.925106052	-4.399863561	-9.451243412
6	-2.591801737	3.596527854	-5.160321828
6	-2.620133390	1.385011407	-4.114538684
1	-1.332195814	-2.069360765	-10.811704017
1	-0.462009413	-0.951721254	-9.714551532
1	0.440656487	-2.241875122	-10.570935300
1	-2.939039470	-2.617560779	-8.931910491
1	-2.353899787	-3.278728922	-7.374156677
1	-2.070878733	-1.560298930	-7.778492886
1	-0.020234992	-4.720562094	-10.005745869
1	-1.118104651	-5.140396552	-8.648541153
1	-1.780004861	-4.440989522	-10.155651855

6	-2.802248827	2.778248968	-4.033183691
6	4.416975107	-4.754043651	-2.921126393
6	4.254118337	-4.027523217	-1.722422730
6	5.683910163	-5.200771152	-3.320369825
6	5.391900303	-3.687089034	-0.963767907
6	6.807728163	-4.893222332	-2.535852216
6	6.666302947	-4.110339870	-1.379073135
1	0.968226660	-4.842572973	-2.008578336
1	-1.936368328	-4.859259277	-5.212124707
1	-0.715495715	-2.364649076	-3.827254053
8	0.768030975	-5.841248009	-4.616019418
9	-1.942556645	3.800175957	-7.436024636
9	-1.546768730	1.107401212	-7.617092232
9	-1.957694210	-0.476361081	-5.464189078
9	-3.147964149	3.390714213	-2.905011055
9	-2.763949173	4.917332359	-5.071459844
9	5.318896817	-2.961558000	0.148306887
9	7.745204610	-3.777491592	-0.670720991
9	8.011114051	-5.326841482	-2.895294428
9	5.823818653	-5.897223771	-4.448699466
9	3.377195295	-5.034164263	-3.701129373

TS with Stacking leading to major isomer: (closed-TS-A)

TS-A1: Imaginary frequency = -128.08

6	-1.679215000	-4.056897000	-2.693101000
6	-1.318284000	-3.877081000	-4.136304000
6	-1.077757000	-5.217078000	-4.832081000
6	0.009674000	-5.915554000	-4.036593000
8	-0.227429000	-5.709828000	-2.699685000
1	-2.157735000	-3.299610000	-4.584424000
1	-0.737276000	-5.085550000	-5.872992000
6	-2.945452000	-4.747302000	-2.340072000
6	-3.989286000	-3.906733000	-1.878802000
6	-3.172453000	-6.137017000	-2.431838000
6	-5.233634000	-4.452627000	-1.540290000
1	-3.832199000	-2.820866000	-1.830412000
6	-4.413695000	-6.674477000	-2.064254000
1	-2.350470000	-6.803738000	-2.718894000
6	-5.449627000	-5.837046000	-1.623132000
1	-6.039196000	-3.782743000	-1.203552000
1	-4.565706000	-7.763081000	-2.114023000
1	-6.423366000	-6.264053000	-1.339677000
6	-1.109548000	-3.183568000	-1.701402000
1	-1.403608000	-3.396404000	-0.662756000
1	-0.017958000	-3.055177000	-1.844516000
1	-0.629618000	2.157756000	4.534973000
6	-0.040749000	2.005112000	3.606950000
6	1.345507000	1.414251000	3.944702000
1	0.042607000	2.983741000	3.095872000
1	-0.616653000	1.328492000	2.947027000
6	2.181259000	1.254823000	2.662554000

6	1.137564000	0.075605000	4.674492000
6	2.092742000	2.382768000	4.891673000
6	-0.677518000	-1.271673000	1.446111000
6	2.772930000	0.034319000	2.278756000
6	2.419739000	2.371569000	1.830614000
1	2.098814000	-0.374628000	4.994203000
1	0.526904000	0.237393000	5.585649000
1	0.602813000	-0.660131000	4.040696000
1	1.521988000	2.520920000	5.833671000
1	3.096767000	1.989354000	5.149827000
1	2.229215000	3.379786000	4.426900000
6	-1.961295000	-0.759001000	1.150219000
6	-0.488548000	-2.622962000	1.763495000
1	0.200511000	-0.614375000	1.425734000
6	3.586453000	-0.068896000	1.141436000
1	2.612711000	-0.872472000	2.877661000
6	3.230472000	2.279536000	0.696094000
1	1.961389000	3.343417000	2.065808000
6	-2.183168000	0.677506000	0.841913000
6	-3.046106000	-1.665508000	1.191861000
6	-1.565850000	-3.530489000	1.806478000
1	0.538617000	-2.965466000	1.944546000
6	3.847924000	1.060216000	0.336593000
1	4.024312000	-1.043016000	0.890753000
1	3.366360000	3.166719000	0.059930000
6	-1.221117000	1.442508000	0.106740000
6	-3.316264000	1.359454000	1.283222000
6	-2.850576000	-3.012672000	1.517875000
1	-4.054130000	-1.312207000	0.927567000
6	-1.378079000	-5.023207000	2.126273000
6	4.758109000	1.012135000	-0.834493000
8	-0.093283000	0.781246000	-0.352167000
6	-1.336140000	2.812638000	-0.151721000
1	-4.065047000	0.826053000	1.887528000
6	-3.539228000	2.732082000	0.984140000
1	-3.722836000	-3.684060000	1.516473000
6	-1.881832000	-5.877195000	0.939910000
6	-2.195731000	-5.367134000	3.391964000
6	0.099507000	-5.375407000	2.375672000
6	4.749720000	-0.078763000	-1.758655000
6	5.646860000	2.050087000	-1.106068000
15	0.018950000	0.486233000	-1.966411000
6	-2.554941000	3.472607000	0.232242000
6	-0.192560000	3.540137000	-0.779367000
6	-4.732771000	3.395293000	1.399562000
1	-1.265180000	-5.705353000	0.035867000
1	-1.814483000	-6.956196000	1.190031000
1	-2.935945000	-5.657103000	0.678408000
1	-2.073605000	-6.439328000	3.650770000
1	-1.857863000	-4.762488000	4.257863000
1	-3.278026000	-5.176131000	3.247376000
1	0.191889000	-6.458607000	2.593451000
1	0.732998000	-5.154713000	1.493476000

1	0.513239000	-4.824487000	3.244255000
8	3.862251000	-1.120920000	-1.518069000
6	5.554852000	-0.156090000	-2.897435000
1	5.703523000	2.900509000	-0.409754000
6	6.482100000	2.054680000	-2.257081000
8	-0.239470000	1.985461000	-2.591815000
7	1.501840000	-0.010855000	-2.125733000
7	-1.218074000	-0.404565000	-2.490343000
6	-2.845596000	4.820638000	-0.131397000
6	0.451850000	4.647853000	-0.118238000
6	0.351655000	3.085023000	-1.982989000
1	-5.474182000	2.825338000	1.979773000
6	-4.968638000	4.717749000	1.061154000
15	2.481050000	-1.206290000	-2.410925000
6	5.397552000	-1.294946000	-3.850071000
6	6.440331000	0.946366000	-3.181415000
6	7.338222000	3.161949000	-2.536450000
16	-2.193013000	-0.084009000	-3.753380000
6	-4.025898000	5.428232000	0.271582000
1	-2.122957000	5.372241000	-0.748880000
6	0.048753000	5.134056000	1.161084000
6	1.597678000	5.258632000	-0.749937000
6	1.490434000	3.662143000	-2.626466000
1	-5.899328000	5.211298000	1.377878000
8	3.048521000	-0.858878000	-3.912294000
7	1.991002000	-2.747439000	-2.291616000
6	4.137130000	-1.568514000	-4.393031000
6	6.506563000	-2.105503000	-4.274549000
6	7.236141000	1.024759000	-4.362978000
1	7.364546000	3.996127000	-1.818095000
6	8.107035000	3.200438000	-3.688820000
1	-4.237175000	6.464415000	-0.031620000
1	-0.802288000	4.662786000	1.669210000
6	0.722455000	6.179635000	1.777378000
6	2.257124000	6.342007000	-0.095370000
6	2.076470000	4.754671000	-1.990937000
6	2.042578000	3.107882000	-3.887655000
16	2.537242000	-3.762313000	-1.152623000
6	3.900445000	-2.501815000	-5.452577000
6	6.301147000	-3.060170000	-5.336622000
6	7.793180000	-2.027123000	-3.664278000
6	8.046751000	2.123794000	-4.612004000
1	7.195304000	0.205466000	-5.091803000
1	8.756022000	4.064991000	-3.893858000
1	0.394740000	6.527694000	2.768520000
6	1.831983000	6.797035000	1.142683000
1	3.124744000	6.802686000	-0.592936000
1	2.942761000	5.244911000	-2.460518000
6	1.220984000	2.675585000	-4.950884000
6	3.442015000	3.022174000	-4.063763000
8	2.612120000	-3.199338000	0.209442000
8	1.757083000	-5.042538000	-1.269392000
6	5.009217000	-3.213837000	-5.910584000

6	2.564127000	-2.645425000	-6.086294000
6	7.408084000	-3.842072000	-5.784762000
6	8.842283000	-2.816785000	-4.111835000
1	7.940551000	-1.337782000	-2.821073000
1	8.642663000	2.160573000	-5.536367000
1	2.356355000	7.627956000	1.637929000
6	1.783329000	2.213882000	-6.149260000
1	0.128371000	2.701666000	-4.855298000
6	3.992751000	2.557315000	-5.261262000
1	4.112327000	3.298764000	-3.236596000
1	4.890950000	-3.912527000	-6.751728000
6	2.161752000	-3.872178000	-6.656390000
6	1.675207000	-1.548897000	-6.217556000
1	7.244335000	-4.563165000	-6.599829000
6	8.654680000	-3.717916000	-5.193673000
1	9.823061000	-2.752442000	-3.617695000
6	3.178206000	2.154453000	-6.343250000
1	1.098879000	1.900445000	-6.949153000
1	5.088373000	2.506125000	-5.344997000
6	0.963952000	-3.979118000	-7.380524000
1	2.784405000	-4.768409000	-6.518342000
6	0.466328000	-1.675569000	-6.904883000
1	1.928294000	-0.571732000	-5.790404000
1	9.494733000	-4.337136000	-5.541492000
6	3.820810000	1.715477000	-7.670604000
6	0.088643000	-2.882992000	-7.531775000
1	0.717114000	-4.952780000	-7.826272000
1	-0.191877000	-0.794925000	-6.947991000
6	-4.217151000	3.470143000	-3.049627000
6	4.594975000	2.919545000	-8.256738000
6	2.774562000	1.255350000	-8.701135000
6	4.804769000	0.551151000	-7.423140000
6	-1.209869000	-2.935367000	-8.354104000
6	-5.177868000	3.075894000	-2.106563000
6	-3.295603000	2.534585000	-3.555221000
1	5.072320000	2.641300000	-9.219487000
1	3.915483000	3.775937000	-8.442337000
1	5.392874000	3.263656000	-7.568559000
1	3.283102000	0.946183000	-9.636614000
1	2.191074000	0.386364000	-8.336159000
1	2.065506000	2.067020000	-8.961122000
1	5.600886000	0.830955000	-6.706398000
1	4.282387000	-0.336284000	-7.017742000
1	5.294798000	0.253374000	-8.373142000
6	-1.101293000	-1.897072000	-9.496424000
6	-2.420133000	-2.581551000	-7.458959000
6	-1.447639000	-4.320980000	-8.977816000
6	-5.174721000	1.760649000	-1.615197000
6	-3.308229000	1.203127000	-3.100176000
1	-2.014354000	-1.919114000	-10.126807000
1	-0.986993000	-0.868354000	-9.101858000
1	-0.227136000	-2.110761000	-10.144317000
1	-3.338163000	-2.488925000	-8.075166000

1	-2.604228000	-3.377264000	-6.709936000
1	-2.281763000	-1.631736000	-6.906594000
1	-0.630579000	-4.609206000	-9.669575000
1	-1.538447000	-5.111840000	-8.205340000
1	-2.392134000	-4.316194000	-9.558207000
6	-4.238414000	0.841681000	-2.106522000
6	4.346276000	-4.953494000	-2.882669000
6	4.209162000	-4.221679000	-1.686039000
6	5.598106000	-5.425044000	-3.301198000
6	5.362776000	-3.909886000	-0.942397000
6	6.737383000	-5.144099000	-2.530084000
6	6.624483000	-4.357101000	-1.373878000
1	0.632815000	-5.534323000	-2.146056000
1	-1.994951000	-5.843557000	-4.843199000
1	-0.417101000	-3.236813000	-4.222009000
8	0.956868000	-6.531859000	-4.465605000
9	-6.075278000	3.954695000	-1.664688000
9	-4.189898000	4.735621000	-3.474056000
9	-2.425286000	2.974423000	-4.462789000
9	-4.253699000	-0.389635000	-1.580382000
9	-6.048891000	1.391975000	-0.673237000
9	5.324488000	-3.184582000	0.173794000
9	7.720442000	-4.045154000	-0.678112000
9	7.929907000	-5.600513000	-2.906161000
9	5.709196000	-6.125554000	-4.432375000
9	3.291879000	-5.210282000	-3.654218000
1	-1.433317000	-2.120402000	-1.976549000
8	-1.489814000	0.468277000	-4.931138000
8	-3.051362000	-1.278925000	-3.947141000

Pathway B: Stepwise Pathway (results for the Major *S* isomer only)

Protonation- via N-H (TS-B1)

TS-A1: Imaginary frequency = -1214.94

6	-1.707128000	-4.025146000	-2.876235000
6	-1.243190000	-3.925874000	-4.295572000
6	0.167948000	-5.890641000	-3.763925000
8	1.416769000	-5.477193000	-3.973297000
1	-2.051159000	-3.541374000	-4.946666000
6	-2.998538000	-4.621839000	-2.575160000
6	-3.191480000	-5.292214000	-1.340216000
6	-4.071176000	-4.582664000	-3.503893000
6	-4.411012000	-5.905169000	-1.045881000
1	-2.353842000	-5.366024000	-0.635911000
6	-5.294926000	-5.181300000	-3.197947000
1	-3.954449000	-4.030380000	-4.445763000
6	-5.467701000	-5.846263000	-1.970550000
1	-4.537141000	-6.439674000	-0.092200000
1	-6.126932000	-5.123900000	-3.915225000
1	-6.431830000	-6.322576000	-1.735608000

6	-0.829718000	-3.551202000	-1.863729000
1	0.250280000	-3.604300000	-2.109399000
1	-1.078262000	-3.768787000	-0.812892000
6	2.248192000	0.910344000	2.951575000
6	-0.175918000	-1.855295000	1.519928000
6	2.946498000	-0.183730000	2.401854000
6	2.297359000	2.125472000	2.233295000
6	-1.523630000	-1.430521000	1.415151000
6	0.147704000	-3.204105000	1.688487000
1	0.640007000	-1.124549000	1.471963000
6	3.681230000	-0.072819000	1.213038000
1	2.935413000	-1.159135000	2.906788000
6	3.028393000	2.247542000	1.048817000
1	1.749858000	3.006478000	2.598669000
6	-1.902498000	-0.001754000	1.270583000
6	-2.518681000	-2.430324000	1.480732000
6	-0.846018000	-4.203961000	1.790021000
1	1.213362000	-3.474416000	1.723240000
6	3.754122000	1.155515000	0.521379000
1	4.209638000	-0.956195000	0.835748000
1	3.016097000	3.205482000	0.508456000
6	-1.090561000	0.930459000	0.550960000
6	-3.080547000	0.501826000	1.818789000
6	-2.187049000	-3.779388000	1.682568000
1	-3.574970000	-2.151455000	1.349884000
6	4.595467000	1.344983000	-0.686108000
8	0.073666000	0.451692000	-0.044532000
6	-1.397784000	2.283443000	0.388720000
1	-3.723036000	-0.162404000	2.415758000
6	-3.501772000	1.843654000	1.616696000
1	-3.004804000	-4.510891000	1.740858000
6	4.717216000	0.346811000	-1.703855000
6	5.310199000	2.525404000	-0.885875000
15	0.037130000	0.239328000	-1.673158000
6	-2.666313000	2.753459000	0.872295000
6	-0.406007000	3.178866000	-0.274982000
6	-4.752576000	2.310376000	2.119954000
8	4.020745000	-0.841980000	-1.538750000
6	5.493173000	0.490706000	-2.856511000
1	5.269956000	3.309925000	-0.115260000
6	6.091946000	2.757284000	-2.050660000
8	-0.427247000	1.734603000	-2.184373000
7	1.507300000	-0.112256000	-2.070181000
7	-1.113875000	-0.757174000	-2.228907000
6	-3.149185000	4.068864000	0.612927000
6	0.150438000	4.323077000	0.401533000
6	0.085670000	2.859848000	-1.542917000
1	-5.378586000	1.612509000	2.697468000
6	-5.184406000	3.603694000	1.871592000
15	2.660616000	-1.116789000	-2.430872000
6	5.528501000	-0.602905000	-3.869880000
6	6.184871000	1.736937000	-3.067023000
6	6.762778000	4.001249000	-2.251218000

16	-2.695131000	-0.420263000	-2.525203000
6	-4.380581000	4.483785000	1.099243000
1	-2.536574000	4.750439000	0.006420000
6	-0.211749000	4.689458000	1.732092000
6	1.161562000	5.103973000	-0.270483000
6	1.102780000	3.600157000	-2.223230000
1	-6.156092000	3.946219000	2.258174000
8	3.133648000	-0.597828000	-3.923327000
7	2.422485000	-2.709219000	-2.408517000
6	4.334720000	-1.083404000	-4.421583000
6	6.768263000	-1.181774000	-4.314337000
6	6.916582000	2.032058000	-4.255867000
1	6.690018000	4.767305000	-1.463494000
6	7.475964000	4.250879000	-3.413006000
8	-3.400034000	0.255321000	-1.423860000
8	-3.267191000	-1.671543000	-3.077701000
1	-4.739666000	5.500519000	0.879346000
1	-0.956830000	4.086839000	2.267664000
6	0.370514000	5.782491000	2.358476000
6	1.729787000	6.229930000	0.397816000
6	1.605510000	4.721291000	-1.566057000
6	1.626547000	3.191096000	-3.550763000
16	3.141999000	-3.726424000	-1.374198000
6	4.278754000	-2.029732000	-5.493883000
6	6.742362000	-2.126707000	-5.403041000
6	8.020145000	-0.888349000	-3.696433000
6	7.544190000	3.258411000	-4.426181000
1	6.972043000	1.276946000	-5.050973000
1	7.981646000	5.217602000	-3.555997000
1	0.077553000	6.038613000	3.387795000
6	1.344008000	6.566946000	1.685371000
1	2.494384000	6.820670000	-0.130416000
1	2.373877000	5.332697000	-2.063095000
6	0.788191000	2.747847000	-4.594065000
6	3.009622000	3.273509000	-3.821703000
8	3.119166000	-3.291604000	0.038389000
8	2.609277000	-5.094815000	-1.651874000
6	5.498036000	-2.508460000	-5.973212000
6	3.002890000	-2.436243000	-6.135463000
6	7.972994000	-2.675038000	-5.874289000
6	9.195889000	-1.455945000	-4.165224000
1	8.041529000	-0.213802000	-2.829409000
1	8.095053000	3.462727000	-5.356644000
1	1.796003000	7.433798000	2.190354000
6	1.303455000	2.435644000	-5.859789000
1	-0.290274000	2.665410000	-4.418529000
6	3.518817000	2.956825000	-5.084285000
1	3.702730000	3.568300000	-3.020887000
1	5.502985000	-3.202010000	-6.826999000
6	2.838692000	-3.744623000	-6.635074000
6	1.954765000	-1.514304000	-6.369946000
1	7.942302000	-3.389304000	-6.710917000
6	9.176754000	-2.340146000	-5.276636000

1	10.148529000	-1.228130000	-3.664409000
6	2.680464000	2.542592000	-6.142456000
1	0.603140000	2.118597000	-6.644621000
1	4.604493000	3.036068000	-5.239630000
6	1.719745000	-4.095617000	-7.403771000
1	3.596824000	-4.507564000	-6.407690000
6	0.843328000	-1.872900000	-7.138177000
1	2.020947000	-0.493217000	-5.971447000
1	10.116276000	-2.781203000	-5.641135000
6	0.707450000	-3.160990000	-7.702315000
1	1.652591000	-5.125664000	-7.779761000
1	0.071939000	-1.112036000	-7.319399000
6	-2.043644000	1.151447000	-6.265633000
6	-2.449983000	2.489865000	-6.144877000
6	-2.119687000	0.297752000	-5.157619000
6	-2.936479000	2.962671000	-4.915098000
6	-2.613778000	0.752838000	-3.921979000
6	-3.008024000	2.099591000	-3.804575000
6	5.196409000	-4.474809000	-3.103530000
6	4.889047000	-3.814889000	-1.897777000
6	6.522990000	-4.684563000	-3.503606000
6	5.949953000	-3.313448000	-1.120316000
6	7.571969000	-4.208245000	-2.702208000
6	7.283561000	-3.494257000	-1.529760000
1	-0.389494000	-3.225442000	-4.367574000
8	-0.215061000	-6.525323000	-2.790344000
6	-0.794245000	-5.329444000	-4.799686000
1	-0.304843000	-5.204620000	-5.784606000
1	-1.667661000	-6.003621000	-4.883325000
9	5.756779000	-2.648198000	0.017575000
9	8.283056000	-2.996806000	-0.797150000
9	8.837855000	-4.412106000	-3.061849000
9	6.786418000	-5.319443000	-4.649850000
9	4.232335000	-4.924378000	-3.911920000
9	-2.348504000	3.309668000	-7.188350000
9	-3.428991000	2.624080000	-2.659112000
9	-1.688223000	-0.958494000	-5.309652000
9	-3.305964000	4.238784000	-4.796978000
9	-1.547110000	0.714339000	-7.433807000
6	-0.430917000	-5.668919000	2.002658000
6	-1.643725000	-6.604955000	2.142941000
1	-2.267525000	-6.620592000	1.226205000
1	-1.296188000	-7.643322000	2.314385000
1	-2.291286000	-6.321059000	2.997485000
6	0.433655000	-6.140458000	0.810493000
1	1.324294000	-5.501804000	0.658478000
1	0.790190000	-7.176694000	0.985361000
1	-0.125621000	-6.145228000	-0.146838000
6	0.401125000	-5.760767000	3.303745000
1	1.319949000	-5.145335000	3.243301000
1	-0.185440000	-5.413260000	4.178674000
1	0.710666000	-6.810272000	3.487900000
6	3.272928000	2.283544000	-7.538060000

6	3.829007000	3.621861000	-8.080010000
1	4.266802000	3.478704000	-9.089857000
1	3.026865000	4.383713000	-8.155350000
1	4.620944000	4.028496000	-7.419678000
6	2.220158000	1.753210000	-8.527556000
1	2.690467000	1.578816000	-9.516126000
1	1.786490000	0.790124000	-8.192499000
1	1.391486000	2.474420000	-8.677342000
6	4.424000000	1.256986000	-7.447056000
1	5.230250000	1.600833000	-6.770013000
1	4.065273000	0.278709000	-7.073843000
1	4.872573000	1.094985000	-8.448731000
6	-0.463821000	-3.462136000	-8.654486000
6	-0.475153000	-4.929588000	-9.115109000
1	-0.572801000	-5.628651000	-8.259450000
1	-1.336363000	-5.106286000	-9.790438000
1	0.445093000	-5.195795000	-9.672492000
6	-0.309899000	-2.554549000	-9.898945000
1	-0.352498000	-1.481828000	-9.624044000
1	0.659833000	-2.736760000	-10.404376000
1	-1.123735000	-2.752562000	-10.627282000
6	-1.816884000	-3.153012000	-7.976314000
1	-1.991273000	-3.824788000	-7.112426000
1	-1.872384000	-2.109904000	-7.612168000
1	-2.647857000	-3.306159000	-8.695316000
6	1.496707000	0.835863000	4.291818000
6	2.236962000	1.737358000	5.308056000
1	1.725435000	1.713110000	6.292989000
1	3.282365000	1.397014000	5.452416000
1	2.268898000	2.791274000	4.965743000
6	1.444148000	-0.597409000	4.849845000
1	0.932467000	-1.291149000	4.152760000
1	2.456525000	-0.998232000	5.057926000
1	0.881836000	-0.606324000	5.805363000
6	0.048372000	1.344206000	4.122553000
1	-0.516101000	0.720116000	3.403585000
1	-0.484395000	1.312304000	5.095274000
1	0.015939000	2.389605000	3.757879000
1	1.937459000	-5.532388000	-3.109335000
1	-0.968477000	-2.374414000	-1.999281000

Protonation-via O-H: (TS-B2)

TS-A1: Imaginary frequency = - 894.2

6	-2.702347000	-4.093630000	-2.774094000
6	-1.686563000	-3.823140000	-3.849106000
6	-0.147473000	-5.563847000	-3.056831000
8	-0.719989000	-5.747821000	-1.986594000
1	-2.218937000	-3.410957000	-4.731315000
6	-3.805677000	-5.029785000	-3.065240000
6	-4.324972000	-5.177993000	-4.373805000
6	-4.359669000	-5.811387000	-2.024049000

6	-5.379410000	-6.063343000	-4.626682000
1	-3.918686000	-4.572981000	-5.197330000
6	-5.402565000	-6.707852000	-2.283025000
1	-3.927239000	-5.740244000	-1.016685000
6	-5.919859000	-6.832552000	-3.582950000
1	-5.783361000	-6.153919000	-5.646197000
1	-5.809710000	-7.321096000	-1.465180000
1	-6.742482000	-7.535203000	-3.785083000
6	-2.693715000	-3.343095000	-1.606024000
1	-1.754673000	-2.856869000	-1.291281000
1	-3.432790000	-3.544549000	-0.815650000
6	2.208782000	1.428231000	2.786150000
6	-0.372304000	-1.420506000	1.446244000
6	2.779750000	0.219514000	2.337548000
6	2.420702000	2.568279000	1.981435000
6	-1.637749000	-0.788858000	1.450212000
6	-0.244367000	-2.804499000	1.625697000
1	0.541230000	-0.831811000	1.306461000
6	3.535588000	0.147005000	1.159491000
1	2.646456000	-0.705546000	2.914734000
6	3.165326000	2.505683000	0.800545000
1	1.989064000	3.536402000	2.269614000
6	-1.821978000	0.670852000	1.243880000
6	-2.766220000	-1.619412000	1.656115000
6	-1.363984000	-3.629429000	1.848503000
1	0.767014000	-3.228772000	1.575293000
6	3.752898000	1.296011000	0.367053000
1	3.958835000	-0.821180000	0.869170000
1	3.270100000	3.413210000	0.188382000
6	-0.948845000	1.443216000	0.412820000
6	-2.895956000	1.358586000	1.808372000
6	-2.627796000	-2.995666000	1.864689000
1	-3.774840000	-1.180593000	1.623753000
6	4.601089000	1.288307000	-0.851015000
8	0.132170000	0.788457000	-0.172678000
6	-1.122826000	2.800795000	0.126167000
1	-3.571548000	0.829435000	2.496528000
6	-3.181679000	2.715085000	1.498780000
1	-3.537451000	-3.594388000	2.028757000
6	4.639809000	0.183466000	-1.761371000
6	5.407985000	2.380695000	-1.168068000
15	0.003655000	0.409542000	-1.761928000
6	-2.309258000	3.448451000	0.615464000
6	-0.087760000	3.521638000	-0.671315000
6	-4.341325000	3.363550000	2.019696000
8	3.858990000	-0.923879000	-1.470712000
6	5.430431000	0.142988000	-2.913489000
1	5.437677000	3.239295000	-0.480691000
6	6.195841000	2.433047000	-2.349700000
8	-0.323793000	1.891504000	-2.412331000
7	1.389604000	-0.150749000	-2.194666000
7	-1.286961000	-0.493493000	-2.162231000
6	-2.679602000	4.770743000	0.233599000

6	0.585665000	4.688341000	-0.156639000
6	0.319401000	3.020860000	-1.911390000
1	-4.992761000	2.800578000	2.706066000
6	-4.658440000	4.661744000	1.653115000
15	2.500967000	-1.254495000	-2.348867000
6	5.414995000	-1.059014000	-3.796370000
6	6.205838000	1.309216000	-3.254529000
6	6.955127000	3.596910000	-2.676637000
16	-2.753574000	0.067455000	-2.439421000
6	-3.828181000	5.362858000	0.738479000
1	-2.046460000	5.315364000	-0.480468000
6	0.311322000	5.239896000	1.130321000
6	1.622511000	5.301071000	-0.952163000
6	1.368767000	3.584776000	-2.702647000
1	-5.561722000	5.145093000	2.054860000
8	3.025452000	-0.954954000	-3.883672000
7	2.169783000	-2.806719000	-2.094796000
6	4.205519000	-1.542544000	-4.309040000
6	6.630206000	-1.737378000	-4.163445000
6	6.944632000	1.428081000	-4.469579000
1	6.945494000	4.442303000	-1.971031000
6	7.674084000	3.673574000	-3.858846000
8	-3.382937000	0.946772000	-1.445901000
8	-3.595165000	-1.170424000	-2.814439000
1	-4.100544000	6.381472000	0.423410000
1	-0.445506000	4.762383000	1.765315000
6	0.996346000	6.353608000	1.595105000
6	2.299510000	6.452814000	-0.449315000
6	1.985071000	4.729498000	-2.201982000
6	1.816176000	2.977428000	-3.981543000
16	2.874494000	-3.691030000	-0.934296000
6	4.114126000	-2.582529000	-5.288739000
6	6.569106000	-2.784588000	-5.152474000
6	7.889575000	-1.440929000	-3.562490000
6	7.658045000	2.581345000	-4.766026000
1	6.934960000	0.595380000	-5.185194000
1	8.248503000	4.580383000	-4.100666000
1	0.767753000	6.753342000	2.594577000
6	1.993507000	6.974250000	0.797323000
1	3.082318000	6.912664000	-1.072476000
1	2.780868000	5.209718000	-2.791234000
6	0.917893000	2.486335000	-4.951064000
6	3.193914000	2.910185000	-4.282529000
8	2.884569000	-3.057840000	0.401392000
8	2.315941000	-5.075384000	-0.999002000
6	5.314077000	-3.161120000	-5.702165000
6	2.826587000	-2.976810000	-5.915381000
6	7.776278000	-3.434581000	-5.549436000
6	9.041093000	-2.106286000	-3.956656000
1	7.935620000	-0.685559000	-2.766069000
1	8.211664000	2.649357000	-5.714644000
1	2.527367000	7.859340000	1.174609000
6	1.373457000	1.984343000	-6.178331000

1	-0.158749000	2.516438000	-4.748798000
6	3.642763000	2.405949000	-5.506113000
1	3.929449000	3.237721000	-3.534033000
1	5.297061000	-3.930694000	-6.487790000
6	2.618140000	-4.302442000	-6.350655000
6	1.810824000	-2.033242000	-6.203400000
1	7.718659000	-4.227898000	-6.310025000
6	8.989532000	-3.097013000	-4.973106000
1	9.998900000	-1.873767000	-3.468053000
6	2.747093000	1.941521000	-6.493660000
1	0.630653000	1.636236000	-6.909299000
1	4.727022000	2.375640000	-5.686163000
6	1.493440000	-4.651528000	-7.112240000
1	3.345830000	-5.080340000	-6.077257000
6	0.688026000	-2.392376000	-6.954075000
1	1.910466000	-0.995701000	-5.859999000
1	9.910055000	-3.616068000	-5.278583000
6	0.512468000	-3.699410000	-7.458560000
1	1.390632000	-5.695905000	-7.436799000
1	-0.061263000	-1.616932000	-7.167437000
6	-2.081193000	0.939052000	-6.395241000
6	-2.348423000	2.315182000	-6.477674000
6	-2.232697000	0.271945000	-5.173346000
6	-2.777995000	3.012467000	-5.336755000
6	-2.667901000	0.955447000	-4.021949000
6	-2.926714000	2.338165000	-4.109252000
6	4.882833000	-4.717589000	-2.575404000
6	4.615171000	-3.908172000	-1.454230000
6	6.194821000	-5.046889000	-2.942191000
6	5.704198000	-3.383704000	-0.733484000
6	7.270329000	-4.547116000	-2.192096000
6	7.024831000	-3.685936000	-1.112675000
1	-0.984273000	-3.036133000	-3.512221000
8	1.156201000	-5.689991000	-3.257297000
6	-0.904004000	-5.075724000	-4.277541000
1	-0.207794000	-4.835068000	-5.101054000
1	-1.599235000	-5.871203000	-4.618606000
9	5.551828000	-2.578137000	0.316819000
9	8.052527000	-3.163363000	-0.438249000
9	8.522376000	-4.862954000	-2.519383000
9	6.420161000	-5.822956000	-4.007206000
9	3.893692000	-5.197879000	-3.333178000
9	-2.172612000	2.955093000	-7.629761000
9	-3.285983000	3.065059000	-3.058948000
9	-1.933057000	-1.028967000	-5.135325000
9	-3.018844000	4.320701000	-5.412611000
9	-1.641247000	0.283418000	-7.478646000
6	-1.238699000	-5.140004000	2.103744000
6	-1.669602000	-5.417229000	3.563166000
1	-2.718545000	-5.107875000	3.747400000
1	-1.589161000	-6.500793000	3.790074000
1	-1.024532000	-4.866506000	4.277534000
6	-2.153470000	-5.924010000	1.136360000

1	-1.832318000	-5.777033000	0.086564000
1	-2.094623000	-7.009723000	1.358501000
1	-3.217212000	-5.625600000	1.241000000
6	0.202992000	-5.639641000	1.902829000
1	0.567881000	-5.456063000	0.873325000
1	0.909485000	-5.160851000	2.610110000
1	0.244100000	-6.732936000	2.083515000
6	3.282725000	1.473030000	-7.857099000
6	3.905137000	2.695367000	-8.572943000
1	4.307216000	2.400316000	-9.564528000
1	3.149075000	3.491397000	-8.728805000
1	4.736825000	3.129872000	-7.982942000
6	2.171466000	0.894028000	-8.750698000
1	2.602216000	0.565276000	-9.717778000
1	1.687618000	0.012156000	-8.286384000
1	1.387030000	1.644542000	-8.976141000
6	4.369508000	0.392246000	-7.661189000
1	5.218553000	0.764457000	-7.054817000
1	3.961893000	-0.503669000	-7.155315000
1	4.773916000	0.073956000	-8.643965000
6	-0.680308000	-4.010402000	-8.379227000
6	-0.717887000	-5.489233000	-8.799406000
1	-0.811390000	-6.162648000	-7.923191000
1	-1.590899000	-5.672697000	-9.457702000
1	0.191084000	-5.781330000	-9.362441000
6	-0.550857000	-3.135770000	-9.649166000
1	-0.580067000	-2.055737000	-9.402510000
1	0.405002000	-3.337831000	-10.173082000
1	-1.383278000	-3.346773000	-10.352362000
6	-2.011228000	-3.672311000	-7.670537000
1	-2.161107000	-4.327829000	-6.789806000
1	-2.045086000	-2.620209000	-7.327338000
1	-2.865225000	-3.831010000	-8.361009000
6	1.447316000	1.541825000	4.118166000
6	2.408851000	2.167134000	5.157037000
1	1.903991000	2.275976000	6.139943000
1	3.307403000	1.532934000	5.297342000
1	2.750775000	3.170563000	4.831780000
6	0.979684000	0.168153000	4.634136000
1	0.330152000	-0.346513000	3.898194000
1	1.831010000	-0.501573000	4.867843000
1	0.399207000	0.297088000	5.570008000
6	0.207450000	2.450192000	3.969859000
1	0.481484000	3.489628000	3.702905000
1	-0.483263000	2.067653000	3.193353000
1	-0.346517000	2.494143000	4.929769000
1	1.620467000	-5.666232000	-2.358685000
1	-3.192788000	-2.244990000	-2.268919000

Pre-reaction-complex before stepwise protonation

6	-1.906129000	-3.504958000	-2.907303000
6	-1.010716000	-3.938250000	-4.044087000
6	-0.173473000	-5.956125000	-2.883390000
8	1.152577000	-5.797585000	-3.030030000
1	-1.378351000	-3.553018000	-5.012714000
6	-3.375485000	-3.651193000	-3.070648000
6	-3.985833000	-3.587819000	-4.344102000
6	-4.210634000	-3.849608000	-1.946589000
6	-5.376024000	-3.690604000	-4.485888000
1	-3.369835000	-3.426307000	-5.239116000
6	-5.598348000	-3.955165000	-2.087204000
1	-3.755109000	-3.947458000	-0.952210000
6	-6.190472000	-3.871748000	-3.358219000
1	-5.827044000	-3.626064000	-5.488063000
1	-6.224613000	-4.115829000	-1.196140000
1	-7.282269000	-3.956133000	-3.469372000
6	-1.352895000	-3.066529000	-1.743569000
1	-0.258261000	-3.044361000	-1.622897000
1	-1.967641000	-2.799638000	-0.871893000
6	1.872692000	1.731012000	2.816831000
6	-0.638009000	-1.149359000	1.411068000
6	2.429551000	0.497812000	2.421598000
6	2.156175000	2.846420000	1.999758000
6	-1.883054000	-0.500598000	1.241903000
6	-0.559702000	-2.527423000	1.655634000
1	0.295770000	-0.577205000	1.356471000
6	3.228202000	0.376309000	1.276077000
1	2.250542000	-0.408509000	3.015788000
6	2.945515000	2.736133000	0.852194000
1	1.747204000	3.833918000	2.253067000
6	-2.011929000	0.956869000	0.983381000
6	-3.044821000	-1.305550000	1.328524000
6	-1.714137000	-3.328862000	1.759846000
1	0.440685000	-2.972018000	1.739503000
6	3.507221000	1.498647000	0.464438000
1	3.634843000	-0.610343000	1.027865000
1	3.109251000	3.629088000	0.231962000
6	-1.065697000	1.682288000	0.193728000
6	-3.098284000	1.690711000	1.459183000
6	-2.958423000	-2.676581000	1.594034000
1	-4.032530000	-0.853744000	1.151420000
6	4.402816000	1.435607000	-0.718290000
8	0.019786000	0.962469000	-0.322596000
6	-1.178152000	3.032088000	-0.148701000
1	-3.833907000	1.196935000	2.111265000
6	-3.313755000	3.049852000	1.106084000
1	-3.891315000	-3.257933000	1.651349000
6	4.463177000	0.297105000	-1.586012000
6	5.248133000	2.499814000	-1.031167000
15	-0.090706000	0.536704000	-1.889629000
6	-2.366162000	3.732438000	0.259788000

6	-0.097324000	3.684020000	-0.944643000
6	-4.479715000	3.747378000	1.542273000
8	3.633375000	-0.773989000	-1.290372000
6	5.337398000	0.185178000	-2.672335000
1	5.259583000	3.382841000	-0.375098000
6	6.104150000	2.489594000	-2.164928000
8	-0.318934000	1.976455000	-2.624556000
7	1.146657000	-0.292948000	-2.286765000
7	-1.529696000	-0.196039000	-2.296963000
6	-2.672992000	5.053232000	-0.178558000
6	0.587219000	4.860971000	-0.468564000
6	0.338055000	3.116867000	-2.145858000
1	-5.188304000	3.222752000	2.201665000
6	-4.732115000	5.044684000	1.126335000
15	2.403030000	-1.256389000	-2.271659000
6	5.393329000	-1.066993000	-3.481180000
6	6.153574000	1.323604000	-3.012763000
6	6.901761000	3.626428000	-2.495950000
16	-3.016126000	0.516380000	-2.768597000
6	-3.828838000	5.693852000	0.243511000
1	-1.986153000	5.559009000	-0.870830000
6	0.289015000	5.479838000	0.781669000
6	1.657302000	5.413766000	-1.263473000
6	1.427554000	3.610538000	-2.929095000
1	-5.640701000	5.566537000	1.462370000
8	3.020320000	-0.972822000	-3.766740000
7	2.150234000	-2.808231000	-1.975565000
6	4.232735000	-1.587890000	-4.060375000
6	6.632957000	-1.767383000	-3.697465000
6	6.973401000	1.372341000	-4.179646000
1	6.860947000	4.504668000	-1.832894000
6	7.696597000	3.635958000	-3.631018000
8	-3.328632000	1.658077000	-1.907235000
8	-3.932100000	-0.614803000	-2.941437000
1	-4.050239000	6.710578000	-0.114428000
1	-0.492986000	5.047317000	1.418494000
6	0.981631000	6.603677000	1.208890000
6	2.341830000	6.577902000	-0.800596000
6	2.048112000	4.770182000	-2.468178000
6	1.922147000	2.919767000	-4.147929000
16	2.738481000	-3.598453000	-0.680899000
6	4.209405000	-2.708033000	-4.949391000
6	6.645794000	-2.887608000	-4.605643000
6	7.842088000	-1.423947000	-3.023200000
6	7.722638000	2.500538000	-4.483477000
1	6.997374000	0.505197000	-4.852840000
1	8.300237000	4.522156000	-3.877857000
1	0.734087000	7.057079000	2.180488000
6	2.011086000	7.166510000	0.409075000
1	3.149975000	6.991797000	-1.423457000
1	2.873159000	5.203024000	-3.053461000
6	1.068043000	2.357420000	-5.119299000
6	3.312159000	2.840700000	-4.387517000

8	2.628576000	-2.842499000	0.584969000
8	2.177506000	-4.975874000	-0.691048000
6	5.436130000	-3.315242000	-5.217164000
6	2.968195000	-3.167285000	-5.622303000
6	7.879281000	-3.559652000	-4.859574000
6	9.019738000	-2.111611000	-3.277364000
1	7.828591000	-0.612867000	-2.282492000
1	8.338800000	2.514714000	-5.395105000
1	2.550214000	8.060470000	0.756623000
6	1.578460000	1.776514000	-6.288716000
1	-0.016417000	2.393941000	-4.968917000
6	3.814439000	2.258617000	-5.554250000
1	4.015804000	3.223063000	-3.634574000
1	5.476045000	-4.151301000	-5.930834000
6	2.775334000	-4.534166000	-5.910969000
6	1.987293000	-2.260205000	-6.090426000
1	7.879227000	-4.407914000	-5.560562000
6	9.045379000	-3.173986000	-4.219492000
1	9.936957000	-1.840381000	-2.734033000
6	2.963362000	1.722347000	-6.544712000
1	0.870768000	1.373011000	-7.025295000
1	4.905571000	2.224353000	-5.685159000
6	1.706351000	-4.965225000	-6.709235000
1	3.468600000	-5.277544000	-5.492079000
6	0.919953000	-2.701099000	-6.877428000
1	2.071415000	-1.190401000	-5.859089000
1	9.986052000	-3.709678000	-4.414750000
6	0.768863000	-4.057121000	-7.241728000
1	1.616313000	-6.040032000	-6.916774000
1	0.196891000	-1.954224000	-7.234284000
6	-1.820972000	0.689818000	-6.685456000
6	-1.975218000	2.045891000	-7.017700000
6	-2.138692000	0.247081000	-5.395214000
6	-2.442628000	2.954597000	-6.055176000
6	-2.600103000	1.149660000	-4.416246000
6	-2.758253000	2.507895000	-4.757208000
6	4.874372000	-4.728517000	-2.064264000
6	4.513221000	-3.831950000	-1.039141000
6	6.214506000	-5.063673000	-2.300725000
6	5.537227000	-3.225811000	-0.287592000
6	7.223032000	-4.482671000	-1.516440000
6	6.886156000	-3.537680000	-0.536272000
1	0.008739000	-3.526945000	-3.907350000
8	-0.714327000	-6.324770000	-1.853202000
6	-0.910851000	-5.484063000	-4.121395000
1	-0.342613000	-5.760207000	-5.031104000
1	-1.917352000	-5.943684000	-4.149431000
9	5.294529000	-2.333280000	0.672152000
9	7.852928000	-2.940981000	0.165712000
9	8.499741000	-4.803611000	-1.718649000
9	6.531320000	-5.918337000	-3.277860000
9	3.951100000	-5.287497000	-2.849603000
9	-1.641819000	2.467506000	-8.233627000

9	-3.170075000	3.420418000	-3.886430000
9	-1.957060000	-1.043698000	-5.108614000
9	-2.576436000	4.242000000	-6.368406000
9	-1.344826000	-0.159910000	-7.604449000
6	-1.652265000	-4.835086000	2.060386000
6	-2.238830000	-5.058056000	3.474743000
1	-3.292894000	-4.719031000	3.538442000
1	-2.211761000	-6.136282000	3.736102000
1	-1.657574000	-4.502649000	4.238846000
6	-2.483019000	-5.629675000	1.027798000
1	-2.064249000	-5.538472000	0.006162000
1	-2.469059000	-6.707602000	1.289115000
1	-3.545524000	-5.312775000	1.013385000
6	-0.209856000	-5.370488000	2.025725000
1	0.264058000	-5.231281000	1.034407000
1	0.431597000	-4.887473000	2.790249000
1	-0.215979000	-6.458425000	2.238411000
6	3.554343000	1.166662000	-7.850918000
6	4.217161000	2.338530000	-8.613521000
1	4.659864000	1.978682000	-9.565426000
1	3.474831000	3.126526000	-8.853366000
1	5.025873000	2.805961000	-8.016818000
6	2.477992000	0.539919000	-8.755371000
1	2.946971000	0.155845000	-9.683316000
1	1.971650000	-0.313948000	-8.263536000
1	1.706609000	1.277903000	-9.055292000
6	4.622458000	0.094316000	-7.539057000
1	5.446238000	0.498774000	-6.918437000
1	4.185199000	-0.767414000	-6.999481000
1	5.068677000	-0.285455000	-8.480957000
6	-0.335978000	-4.468433000	-8.231235000
6	-0.370344000	-5.988791000	-8.461480000
1	-0.563658000	-6.540922000	-7.519406000
1	-1.182547000	-6.244697000	-9.171213000
1	0.579387000	-6.363406000	-8.892981000
6	-0.045684000	-3.768969000	-9.581275000
1	-0.071154000	-2.665540000	-9.479706000
1	0.955222000	-4.050324000	-9.966226000
1	-0.803051000	-4.059001000	-10.339023000
6	-1.726794000	-4.024352000	-7.725474000
1	-2.012486000	-4.578911000	-6.810173000
1	-1.763796000	-2.940465000	-7.499445000
1	-2.496729000	-4.232625000	-8.496559000
6	1.042382000	1.898255000	4.100560000
6	1.938779000	2.581719000	5.160359000
1	1.378236000	2.731208000	6.106904000
1	2.832586000	1.964045000	5.381301000
1	2.291197000	3.572597000	4.808955000
6	0.557032000	0.545897000	4.653991000
1	-0.054266000	-0.005324000	3.911608000
1	1.399621000	-0.106503000	4.957912000
1	-0.069062000	0.712162000	5.553730000
6	-0.194809000	2.784813000	3.836797000

1	0.085320000	3.812644000	3.532842000
1	-0.836480000	2.356061000	3.042508000
1	-0.803456000	2.871231000	4.759961000
1	1.566042000	-5.750358000	-2.121513000
1	-1.545701000	-1.259213000	-2.300762000

Product-complex after stepwise protonation

C	-2.18149651644312	-3.59807713334799	-2.79356924413134
C	-1.49643184036726	-3.82248604438550	-4.10771183602886
C	-0.44439652318781	-5.80275876445214	-3.07516704490569
O	0.86605163443554	-5.58938942701826	-3.18365644754464
H	-2.11997142413347	-3.45165121175829	-4.94227585974978
C	-3.59326934768473	-3.91419523953078	-2.64610072398885
C	-4.42409925338282	-4.12973139159220	-3.77820681606137
C	-4.17669235927819	-4.01493072511615	-1.35688360327550
C	-5.77710216186738	-4.43560979489837	-3.62415066019246
H	-4.01032056136380	-4.02439918488152	-4.78995793875929
C	-5.52943558915391	-4.32339822293757	-1.20482022068637
H	-3.55039315582475	-3.87229612648501	-0.46869744416393
C	-6.33477365781772	-4.53426395907308	-2.33698776497002
H	-6.40838975952895	-4.58895315183422	-4.51178682363764
H	-5.96028924638378	-4.40645644075174	-0.19579646246751
H	-7.40195756854925	-4.77598215069845	-2.21750819227799
C	-1.38918666931577	-3.06066770754475	-1.74332559883092
H	-0.30568895024048	-3.28227463244734	-1.80907954357254
H	-1.79156280443458	-3.05708835786411	-0.71843820238984
C	1.89930227427423	1.74777415235750	2.66456119829047
C	-0.66360289796036	-1.03283482718249	1.40599877855040
C	2.50228358499388	0.51323238189334	2.34987499177926
C	2.15069224983086	2.82381432511454	1.78477419194255
C	-1.95028204290962	-0.49310574140769	1.17583054497270
C	-0.48545696872112	-2.37527125699649	1.76659239160468
H	0.22721929860130	-0.40209466173861	1.30478309637957
C	3.33329811994730	0.35743999648606	1.23201600822772
H	2.33612097327030	-0.36437027138059	2.98901023311359
C	2.97699401534516	2.67905047015665	0.66692354119576
H	1.68894325677786	3.80520438113403	1.96564488986092
C	-2.17080174143623	0.92866678814473	0.80373571619191
C	-3.04999096019222	-1.36968456920495	1.34104687560184
C	-1.57727057874563	-3.24792205697899	1.94302678545849
H	0.54415885938433	-2.73567524406318	1.88501096329504
C	3.60191473906747	1.44580724561797	0.37411423697483
H	3.77951361557240	-0.62548650651813	1.04126199751600
H	3.12044977015767	3.53625502077520	-0.00726119022724
C	-1.22388860414417	1.67202826202739	0.02857317701607
C	-3.32501635951522	1.61246232065029	1.18266636890971
C	-2.86547163531248	-2.70150618593628	1.73287270888055
H	-4.06813465851567	-1.00662570401022	1.13522443215536
C	4.54358523534775	1.34619785202985	-0.76826621551974
O	-0.07738172029741	1.01101027836043	-0.40521903254409
C	-1.38181966286669	3.01103174642915	-0.33936680618019

H	-4.06834818639982	1.10525624492528	1.81544049648778
C	-3.59677022214040	2.94230148550460	0.76326612047890
H	-3.75533285932225	-3.33657426622505	1.86797462964353
C	4.61611950160081	0.18710051597931	-1.60409795132845
C	5.40923586194932	2.39271714207054	-1.08299247061325
P	-0.03348905140706	0.57755065322571	-1.98933051049880
C	-2.62771861551675	3.65612643713372	-0.03074825120213
C	-0.26163109312306	3.71401866620926	-1.03128905895538
C	-4.82607656852276	3.58600615287775	1.09515110743545
O	3.76595086856460	-0.87239713186132	-1.32337995214768
C	5.49159291987108	0.04939394218151	-2.68367063897551
H	5.40843780062617	3.29370882297770	-0.45112809340878
C	6.29753113500463	2.34164970125987	-2.19220696641983
O	-0.28449218006158	2.02610568606770	-2.72910753960616
N	1.40603040731473	0.01544945256651	-2.22534342642856
N	-1.26185974725547	-0.35025389509083	-2.49862724709495
C	-2.96138233177595	4.95537090990479	-0.51162171832335
C	0.34951810344293	4.89382085195818	-0.47230923164611
C	0.29110240138755	3.17673992877019	-2.19629123949226
H	-5.55470206026278	3.03840499196655	1.71296791243434
C	-5.11147096189429	4.86114268414696	0.63337782710622
P	2.45803018560505	-1.15088947467146	-2.28861866241198
C	5.47435011713348	-1.19826958771918	-3.50075820614867
C	6.34521987780630	1.16043169545448	-3.02008751473661
C	7.12580933134321	3.45607332088820	-2.52313936380454
S	-2.75559395416736	0.13503561671827	-2.99444066865324
C	-4.17598676412403	5.54333948468273	-0.18928759650054
H	-2.24640148213406	5.48311428364951	-1.15807712123637
C	-0.07856670136686	5.48319353118111	0.75454136755773
C	1.48059586530077	5.47956070920398	-1.15169283821875
C	1.42773488191171	3.71816435218753	-2.87517637641003
H	-6.06829952340306	5.34077317249040	0.88905414192013
O	3.10874861853378	-0.95333226160408	-3.79112850572308
N	2.02448468222844	-2.67953481216255	-2.02137446986017
C	4.27996188424029	-1.63745636033885	-4.08446031576591
C	6.66724847538275	-1.97141689689141	-3.72289306692261
C	7.19949681305677	1.17029191430444	-4.16284438338170
H	7.08464839876135	4.34771304239997	-1.87821524858089
C	7.95176674306313	3.42916830110899	-3.63576094237748
O	-3.42835285880202	1.07419908706560	-2.08280700988462
O	-3.45989168088321	-1.10405145382886	-3.39762639766222
H	-4.41876294883459	6.54329116314710	-0.57939104816124
H	-0.91853881695942	5.03282008322590	1.29861631828669
C	0.55541962226279	6.60392418612150	1.27225709861770
C	2.10131316612258	6.63961308084175	-0.59817500707345
C	1.98575436216323	4.87499064611488	-2.33541279345497
C	2.01497369423064	3.06972046198614	-4.07482774336176
S	2.54936426023103	-3.56193324595516	-0.76929035235872
C	4.19830007890571	-2.73703428928129	-4.99724006723302
C	6.61659708272438	-3.07659017131170	-4.64748595145710
C	7.89055381847982	-1.71327372216690	-3.03609763707410
C	7.98074517093376	2.27716890429777	-4.46526863513388
H	7.22746878153173	0.28931328780553	-4.81730328267103

H	8.57851624810087	4.29939698740157	-3.88190914224192
H	0.20784757805620	7.03337243889163	2.22393561585914
C	1.65040388754538	7.19499559349064	0.58852689356447
H	2.95827505478415	7.07926724374991	-1.13196914242427
H	2.84757512382569	5.33858019516936	-2.83899745001990
C	1.22550103100038	2.54675545214033	-5.11917639044734
C	3.41705362442519	2.98477662436188	-4.21817338230114
O	2.51121146152134	-2.87064446441069	0.53607490137337
O	1.85623392300317	-4.88573584530932	-0.83568705377481
C	5.38617552283296	-3.41399785387107	-5.27303038609786
C	2.93997657649556	-3.10650925764528	-5.69367787513757
C	7.80628307930630	-3.82303555072104	-4.90149409210095
C	9.02343609426013	-2.47267735989063	-3.28973489230149
H	7.92195341584025	-0.90981668548010	-2.28732531318052
H	8.62382131519644	2.26117204669600	-5.35809753136048
H	2.14331085763235	8.08562525330686	1.00631748173696
C	1.80958887323016	1.99099529441641	-6.26558736042104
H	0.13366959304746	2.59185125158715	-5.04189250907559
C	3.99476049800768	2.42565720821080	-5.36174691726525
H	4.06809156168851	3.33970980466395	-3.40651042520004
H	5.37772802255213	-4.23523352490542	-6.00475608662390
C	2.66472231850237	-4.45385170509036	-6.00652372901998
C	2.03030170369387	-2.13063354993603	-6.16678304734740
H	7.75813071353287	-4.65947947267314	-5.61503523956210
C	8.98879586647302	-3.52235527035966	-4.24590057769762
H	9.95235246496311	-2.26679562523135	-2.73750847951902
C	3.20871336281974	1.92297374883530	-6.42197817692808
H	1.14858635501588	1.61822309562364	-7.05978257805100
H	5.09204145720853	2.38015358124408	-5.41879040304824
C	1.58136759269715	-4.80468904440026	-6.82496933364054
H	3.30546549677092	-5.24519086121682	-5.59181724199312
C	0.95170311482432	-2.48975170288324	-6.98001799503805
H	2.18204330079662	-1.07173450035356	-5.91957878637982
H	9.89476690912566	-4.11509664758285	-4.44069420447214
C	0.71389934454250	-3.82970941467505	-7.35865520122244
H	1.42665215729357	-5.86862166955467	-7.05109224319708
H	0.29216702558905	-1.69130279976715	-7.34652781600988
C	-1.58764421993188	0.95029334533912	-6.84853778487900
C	-1.83609780323775	2.32448374475036	-6.99338003243440
C	-1.86500536264181	0.31631177947532	-5.63067061917470
C	-2.36498204759187	3.05467232375174	-5.91672328348901
C	-2.40103082827413	1.03204278099125	-4.54493661409560
C	-2.63773930260481	2.41204270260424	-4.69344800541728
C	4.59606778809000	-4.83199638509640	-2.17853034823819
C	4.29926436408659	-3.94631672587398	-1.12478100933244
C	5.90699084265260	-5.26061386040667	-2.42654842493067
C	5.36481064230581	-3.44865167248583	-0.35023909868584
C	6.95487587742778	-4.78544775249243	-1.62315404720222
C	6.68681026817700	-3.85387087704490	-0.60951844526146
H	-0.54167482691348	-3.26314496536154	-4.13344147110611
O	-1.01846263105595	-6.19132770425513	-2.06750680612707
C	-1.19346390710980	-5.33482871205587	-4.31222447818251
H	-0.57941377794595	-5.44601627645783	-5.22632561540379

H	-2.13441264787040	-5.90993772070287	-4.40474074609675
F	5.19025188867758	-2.57930970482747	0.64374052532905
F	7.69312809600503	-3.36081511724310	0.11663187202608
F	8.20363120687158	-5.19615277612915	-1.83554013069008
F	6.15946389387216	-6.10241509972073	-3.43368470272222
F	3.63519706477660	-5.29397671865238	-2.98329079407930
F	-1.54221583442160	2.93321228855296	-8.13998832833525
F	-3.08864332215613	3.17094894244927	-3.70079239105846
F	-1.58196273125711	-0.98677338857832	-5.52918670039423
F	-2.58372186593463	4.36363788053432	-6.04939011617930
F	-1.04940162860618	0.26536106904399	-7.87007706254849
C	-1.40167145782122	-4.71834402574345	2.35796504066806
C	-2.05365198694071	-4.91191960415086	3.74678842039349
H	-3.13674204244173	-4.67446344170388	3.72956880565647
H	-1.94314921880458	-5.96436124927268	4.08106716954116
H	-1.57597928043528	-4.25727365128148	4.50378180591614
C	-2.08847920962202	-5.64918319275316	1.33277144690370
H	-1.63264295168649	-5.56757597097732	0.32570670443914
H	-1.98416962592366	-6.70568010987999	1.65505438912114
H	-3.17492716152777	-5.44343061785249	1.24745017873067
C	0.08278243015958	-5.11611349475383	2.44448933380886
H	0.60360192719904	-4.99506212200411	1.47437682246941
H	0.62594028276778	-4.52053211735267	3.20554521789909
H	0.16266634535178	-6.18236967391795	2.73768123746148
C	3.88363041482831	1.37773748159095	-7.69181911807718
C	4.65611954695431	2.53650002279168	-8.36545890259503
H	5.15775364116906	2.18163505834371	-9.28975190707948
H	3.97093583982201	3.36413775692425	-8.63945098422993
H	5.43449657512171	2.94977950006999	-7.69333423119919
C	2.86207952432393	0.81898518510276	-8.69829182803065
H	3.39082790115068	0.43737384836005	-9.59474622827077
H	2.28091159425571	-0.02347387422873	-8.27314813739054
H	2.14838105473200	1.59610041105418	-9.03897095890376
C	4.87649730374128	0.25295412241966	-7.32334522416258
H	5.65309000021323	0.60496181027965	-6.61684911511188
H	4.35836017761759	-0.60500333189184	-6.85385894537309
H	5.39114049468374	-0.11784081762935	-8.23363464755242
C	-0.40257065108301	-4.15516172616387	-8.36702167221556
C	-0.54315792518961	-5.66745020801580	-8.60959389754562
H	-0.78843112248072	-6.21263316870631	-7.67523231190479
H	-1.36128868184314	-5.85915518872346	-9.33271765565704
H	0.38308601998768	-6.10691759222265	-9.03070864941846
C	-0.04674821921250	-3.46995003477335	-9.70862263110698
H	0.00821926458354	-2.36870822400869	-9.59792341096492
H	0.93478282746022	-3.82245157755071	-10.08453601862384
H	-0.81488156452547	-3.69759115478191	-10.47681527825349
C	-1.76236328661015	-3.61217337400952	-7.87453066407081
H	-2.08456111331091	-4.13397064428941	-6.95133472372388
H	-1.72594650648328	-2.52769615573801	-7.65800543312077
H	-2.54271211134395	-3.77886250808002	-8.64528896741711
C	1.04566710450503	1.96135642352944	3.92632159691549
C	1.83542173286117	2.88095271415985	4.88764101193923
H	1.25386168722004	3.06444511218044	5.81531499689363

H	2.80344924491723	2.42048469779788	5.17117227320072
H	2.05203753588388	3.86239279922327	4.41969799650934
C	0.73629533827252	0.63798790328945	4.64866407741922
H	0.18995190219451	-0.06902234152025	3.99233312996330
H	1.65684361863703	0.13612069768685	5.00840565329228
H	0.10019446216298	0.83575205870375	5.53497192700746
C	-0.29333511191177	2.63881703732241	3.56262496916316
H	-0.14134546820618	3.62703631165819	3.08662134902159
H	-0.88442137506417	2.01504323546290	2.86477545259086
H	-0.90072910654922	2.79931390981951	4.47710555090572
H	1.27615119455778	-5.53695765032093	-2.26322686376846
H	-1.35146664769817	-1.90942966304704	-2.07965364216583

Stepwise Cyclization: (*TS-C1*)

Imaginary frequency = -59.25

1	-2.152731000	2.825125000	6.727409000
6	-1.493927000	2.639447000	5.853772000
6	-0.142091000	2.053245000	6.314806000
1	-1.361023000	3.599044000	5.319185000
1	-2.022039000	1.945461000	5.172190000
6	0.782221000	1.853015000	5.101042000
6	-0.413646000	0.736893000	7.063818000
6	0.535455000	3.044767000	7.289424000
6	-2.239390000	-0.645911000	3.779998000
6	1.339910000	0.604057000	4.762180000
6	1.131445000	2.953786000	4.285747000
1	0.519144000	0.284012000	7.456166000
1	-1.080600000	0.930148000	7.928238000
1	-0.916632000	-0.009290000	6.416332000
1	-0.104089000	3.207783000	8.181933000
1	1.516007000	2.656991000	7.632562000
1	0.708606000	4.029952000	6.812013000
6	-3.506622000	-0.111751000	3.449381000
6	-2.086196000	-1.992434000	4.136683000
1	-1.343831000	-0.012693000	3.747123000
6	2.218774000	0.455022000	3.680555000
1	1.096886000	-0.291283000	5.350022000
6	2.010251000	2.815122000	3.207411000
1	0.705994000	3.947970000	4.488537000
6	-3.693570000	1.314893000	3.080149000
6	-4.613354000	-0.993711000	3.509055000
6	-3.185396000	-2.875780000	4.186169000
1	-1.068380000	-2.352480000	4.336241000
6	2.582808000	1.563352000	2.888137000
1	2.620990000	-0.540833000	3.460408000
1	2.232087000	3.690460000	2.579239000
6	-2.699648000	2.028775000	2.333053000
6	-4.823634000	2.030600000	3.464143000
6	-4.455348000	-2.333997000	3.876250000
1	-5.609497000	-0.624308000	3.223443000
6	-3.039325000	-4.363312000	4.548104000

6	3.537714000	1.449947000	1.757846000
8	-1.607211000	1.305966000	1.896509000
6	-2.745702000	3.408656000	2.099480000
1	-5.602744000	1.530558000	4.058905000
6	-5.011924000	3.395825000	3.112356000
1	-5.344643000	-2.982601000	3.888085000
6	-3.597268000	-5.240139000	3.402202000
6	-3.844140000	-4.637681000	5.839464000
6	-1.569383000	-4.756179000	4.782059000
6	3.478464000	0.360812000	0.832449000
6	4.509684000	2.418199000	1.518535000
15	-1.251869000	1.090947000	0.294443000
6	-3.965129000	4.104401000	2.417718000
6	-1.533178000	4.116726000	1.591514000
6	-6.221214000	4.083128000	3.424558000
1	-2.990776000	-5.133562000	2.480057000
1	-3.567658000	-6.310732000	3.691915000
1	-4.646637000	-4.988426000	3.149664000
1	-3.751924000	-5.703957000	6.132724000
1	-3.469848000	-4.015723000	6.677468000
1	-4.921949000	-4.414580000	5.707578000
1	-1.506903000	-5.836779000	5.022215000
1	-0.941363000	-4.572645000	3.887221000
1	-1.126210000	-4.200535000	5.632659000
8	2.508780000	-0.607088000	1.044230000
6	4.317721000	0.216128000	-0.275006000
1	4.604515000	3.264841000	2.215496000
6	5.382243000	2.356449000	0.396268000
8	-1.462348000	2.625661000	-0.273595000
7	0.268516000	0.667940000	0.354171000
7	-2.198097000	0.174695000	-0.620589000
6	-4.194872000	5.457443000	2.034673000
6	-0.909712000	5.179224000	2.345834000
6	-0.896608000	3.682032000	0.428483000
1	-7.011611000	3.536172000	3.961783000
6	-6.411829000	5.403054000	3.044800000
15	1.154228000	-0.613571000	0.106671000
6	4.115797000	-0.920118000	-1.223805000
6	5.292782000	1.249045000	-0.526633000
6	6.324615000	3.397947000	0.143764000
16	-3.791151000	0.268834000	-0.869995000
6	-5.393245000	6.089790000	2.334276000
1	-3.412647000	5.992046000	1.477235000
6	-1.417673000	5.653567000	3.591581000
6	0.320731000	5.752359000	1.852140000
6	0.318035000	4.235231000	-0.085679000
1	-7.356059000	5.915865000	3.281806000
8	1.802806000	-0.334014000	-1.380067000
7	0.550260000	-2.111621000	0.237904000
6	2.859206000	-1.119485000	-1.807946000
6	5.181400000	-1.811808000	-1.595513000
6	6.133359000	1.264794000	-1.679625000
1	6.385825000	4.231563000	0.860616000

6	7.132797000	3.376721000	-0.981830000
8	-4.581013000	0.138322000	0.384822000
8	-4.132618000	-0.658371000	-1.978614000
1	-5.557580000	7.128278000	2.010072000
1	-2.341147000	5.218688000	3.993250000
6	-0.758835000	6.643386000	4.308387000
6	0.965042000	6.777083000	2.607472000
6	0.895064000	5.269908000	0.642797000
6	0.941225000	3.703821000	-1.323132000
16	0.975136000	-3.108293000	1.445722000
6	2.598688000	-2.056324000	-2.859417000
6	4.948823000	-2.772827000	-2.646427000
6	6.448270000	-1.809415000	-0.940157000
6	7.027566000	2.302801000	-1.903896000
1	6.062052000	0.444983000	-2.405697000
1	7.847860000	4.192319000	-1.166612000
1	-1.170393000	6.980379000	5.271688000
6	0.441277000	7.216311000	3.813199000
1	1.900469000	7.205219000	2.214760000
1	1.825776000	5.729348000	0.276035000
6	0.187400000	3.472073000	-2.490620000
6	2.324359000	3.427659000	-1.372607000
8	1.031233000	-2.477368000	2.781034000
8	0.133083000	-4.340915000	1.361708000
6	3.669591000	-2.852635000	-3.263245000
6	1.275357000	-2.120824000	-3.533444000
6	6.014282000	-3.637751000	-3.038762000
6	7.455840000	-2.677514000	-1.334637000
1	6.612967000	-1.115706000	-0.103854000
1	7.655966000	2.292329000	-2.807172000
1	0.954923000	8.001221000	4.388291000
6	0.789537000	2.994480000	-3.661521000
1	-0.887057000	3.687410000	-2.495391000
6	2.921589000	2.955601000	-2.545974000
1	2.937143000	3.556380000	-0.468162000
1	3.531324000	-3.562490000	-4.092116000
6	0.782680000	-3.337614000	-4.059487000
6	0.490715000	-0.962922000	-3.742441000
1	5.829423000	-4.363572000	-3.845104000
6	7.245046000	-3.586679000	-2.405264000
1	8.420432000	-2.669949000	-0.805682000
6	2.172892000	2.729975000	-3.722830000
1	0.149979000	2.842358000	-4.541474000
1	4.002614000	2.753884000	-2.534552000
6	-0.411101000	-3.376801000	-4.792339000
1	1.327121000	-4.274561000	-3.869424000
6	-0.715989000	-1.019222000	-4.448566000
1	0.819432000	0.008877000	-3.355393000
1	8.052035000	-4.269194000	-2.710347000
6	2.877193000	2.266759000	-5.009946000
6	-1.197166000	-2.220084000	-5.004502000
1	-0.735195000	-4.345687000	-5.203744000
1	-1.284580000	-0.088905000	-4.563414000

6	-3.472966000	3.673776000	-3.123611000
6	3.839717000	3.389469000	-5.463915000
6	1.878972000	1.980537000	-6.145629000
6	3.689165000	0.980923000	-4.742488000
6	-2.483858000	-2.271590000	-5.847784000
6	-4.308904000	4.593677000	-2.467945000
6	-3.376644000	2.362412000	-2.643197000
1	4.364116000	3.096178000	-6.397268000
1	3.287955000	4.331536000	-5.658041000
1	4.608216000	3.599374000	-4.693049000
1	2.425946000	1.636258000	-7.046431000
1	1.159234000	1.184162000	-5.867312000
1	1.304920000	2.884975000	-6.431247000
1	4.433763000	1.123204000	-3.936054000
1	3.029641000	0.142769000	-4.447999000
1	4.236963000	0.676127000	-5.657988000
6	-2.086745000	-2.572116000	-7.311868000
6	-3.254702000	-0.939002000	-5.799194000
6	-3.431011000	-3.379740000	-5.338881000
6	-5.040025000	4.191873000	-1.339471000
6	-4.083429000	1.950493000	-1.498819000
1	-2.987144000	-2.608919000	-7.960002000
1	-1.409287000	-1.787471000	-7.705074000
1	-1.562500000	-3.545021000	-7.399899000
1	-4.187216000	-1.026859000	-6.392572000
1	-3.534133000	-0.667136000	-4.761316000
1	-2.667097000	-0.104206000	-6.230779000
1	-2.954227000	-4.379899000	-5.347765000
1	-3.773660000	-3.163444000	-4.307252000
1	-4.330662000	-3.436868000	-5.985303000
6	-4.934080000	2.872344000	-0.860441000
6	2.785836000	-4.440888000	-0.178567000
6	2.643689000	-3.681216000	0.999115000
6	4.020643000	-4.998368000	-0.537799000
6	3.781053000	-3.439357000	1.790366000
6	5.141988000	-4.779039000	0.278150000
6	5.029686000	-3.970365000	1.419260000
9	3.741947000	-2.699952000	2.898469000
9	6.112197000	-3.721085000	2.160806000
9	6.318577000	-5.315041000	-0.040949000
9	4.132314000	-5.726873000	-1.651351000
9	1.750645000	-4.641075000	-0.993693000
9	-2.538855000	1.530831000	-3.269048000
9	-5.656863000	2.560857000	0.211438000
9	-5.848631000	5.062850000	-0.732042000
9	-4.406382000	5.843623000	-2.916909000
9	-2.755337000	4.060723000	-4.184183000
6	-3.176411000	-3.264174000	-0.172533000
6	-2.457662000	-3.307000000	-1.460120000
6	-2.353978000	-4.644495000	-2.190569000
6	-1.383781000	-5.507328000	-1.394960000
8	-1.690371000	-5.458444000	-0.069517000
1	-3.048388000	-2.546169000	-2.046957000

1	-1.930976000	-4.496117000	-3.196800000
6	-4.500160000	-3.860226000	0.029832000
6	-5.543735000	-2.993008000	0.456891000
6	-4.784288000	-5.237485000	-0.157647000
6	-6.836112000	-3.492865000	0.647911000
1	-5.349388000	-1.914564000	0.560134000
6	-6.071472000	-5.729059000	0.082647000
1	-3.968387000	-5.931413000	-0.387633000
6	-7.103912000	-4.859892000	0.473131000
1	-7.640849000	-2.802366000	0.941693000
1	-6.267138000	-6.806165000	-0.026457000
1	-8.117602000	-5.251616000	0.647152000
6	-2.648410000	-2.445778000	0.906082000
1	-2.485861000	-3.137126000	1.770518000
1	-1.700286000	-1.949709000	0.659522000
1	-3.407121000	-1.741268000	1.316222000
1	-0.858875000	-5.326470000	0.481374000
1	-3.336289000	-5.147321000	-2.304867000
1	-1.455350000	-2.846738000	-1.342608000
8	-0.438011000	-6.103929000	-1.862535000

Pre-reaction-complex before cyclization

1	-2.044388000	2.788452000	6.613572000
6	-1.356643000	2.590971000	5.764972000
6	0.025226000	2.137221000	6.282740000
1	-1.281523000	3.516242000	5.163430000
1	-1.818730000	1.812506000	5.128782000
6	0.982557000	1.952475000	5.092666000
6	-0.161964000	0.837572000	7.084706000
6	0.598367000	3.220830000	7.224789000
6	-2.284919000	-0.651695000	3.672552000
6	1.561417000	0.710433000	4.765712000
6	1.322642000	3.055448000	4.276034000
1	0.796598000	0.466109000	7.500097000
1	-0.849547000	1.019266000	7.935388000
1	-0.606888000	0.032849000	6.465236000
1	-0.070280000	3.364389000	8.099030000
1	1.601079000	2.931946000	7.600099000
1	0.696062000	4.198415000	6.711909000
6	-3.526213000	-0.042621000	3.376534000
6	-2.195186000	-2.019723000	3.971808000
1	-1.358377000	-0.064065000	3.662657000
6	2.443377000	0.568499000	3.686497000
1	1.325635000	-0.186597000	5.353323000
6	2.204953000	2.923420000	3.199608000
1	0.881358000	4.044209000	4.472184000
6	-3.670438000	1.405500000	3.074170000
6	-4.670251000	-0.875186000	3.398543000
6	-3.335025000	-2.846951000	4.001503000
1	-1.193871000	-2.432956000	4.152100000
6	2.787845000	1.675594000	2.884337000

1	2.856822000	-0.423718000	3.469578000
1	2.413676000	3.797630000	2.565083000
6	-2.649389000	2.150239000	2.397672000
6	-4.810657000	2.114127000	3.447358000
6	-4.576297000	-2.233881000	3.712544000
1	-5.647745000	-0.453630000	3.122753000
6	-3.269566000	-4.347077000	4.332559000
6	3.707259000	1.551500000	1.726955000
8	-1.516885000	1.461963000	2.001721000
6	-2.703137000	3.534130000	2.188390000
1	-5.609749000	1.595611000	3.997645000
6	-4.992475000	3.489090000	3.135753000
1	-5.497244000	-2.837050000	3.707847000
6	-3.979936000	-5.162551000	3.227779000
6	-3.989307000	-4.581322000	5.681091000
6	-1.818658000	-4.849221000	4.449819000
6	3.592438000	0.462034000	0.807441000
6	4.679543000	2.506379000	1.441462000
15	-1.124606000	1.257269000	0.409275000
6	-3.931823000	4.219388000	2.487134000
6	-1.489732000	4.255501000	1.704540000
6	-6.211107000	4.165258000	3.437767000
1	-3.490164000	-5.022108000	2.244838000
1	-3.949401000	-6.244876000	3.473442000
1	-5.045849000	-4.880502000	3.117671000
1	-3.966871000	-5.657570000	5.952368000
1	-3.497888000	-4.010210000	6.494373000
1	-5.050282000	-4.262591000	5.638182000
1	-1.818304000	-5.937200000	4.667590000
1	-1.238391000	-4.680806000	3.520081000
1	-1.277480000	-4.350351000	5.278457000
8	2.611819000	-0.487454000	1.053212000
6	4.377178000	0.303273000	-0.335807000
1	4.813793000	3.356036000	2.128355000
6	5.500002000	2.429279000	0.280640000
8	-1.404025000	2.778100000	-0.171389000
7	0.424194000	0.923952000	0.467517000
7	-2.006557000	0.269886000	-0.493768000
6	-4.154857000	5.582953000	2.136995000
6	-0.869303000	5.304258000	2.478527000
6	-0.853359000	3.843305000	0.533900000
1	-7.013242000	3.601519000	3.939094000
6	-6.395005000	5.495247000	3.092201000
15	1.215444000	-0.418217000	0.182980000
6	4.113124000	-0.834604000	-1.266536000
6	5.355785000	1.319881000	-0.633534000
6	6.441337000	3.458683000	-0.020094000
16	-3.584397000	0.249827000	-0.853103000
6	-5.360708000	6.204788000	2.427358000
1	-3.360118000	6.134944000	1.615395000
6	-1.379317000	5.751139000	3.733547000
6	0.361718000	5.887246000	1.997765000
6	0.355315000	4.414694000	0.026603000

1	-7.345681000	5.999694000	3.321508000
8	1.809521000	-0.199901000	-1.342494000
7	0.515598000	-1.861586000	0.351346000
6	2.828867000	-1.017871000	-1.796939000
6	5.151505000	-1.745304000	-1.666520000
6	6.143265000	1.320190000	-1.823380000
1	6.544907000	4.294788000	0.688931000
6	7.195760000	3.424163000	-1.182248000
8	-4.449949000	0.005881000	0.329245000
8	-3.761466000	-0.661579000	-2.010306000
1	-5.519132000	7.252829000	2.132005000
1	-2.306094000	5.310634000	4.122003000
6	-0.718048000	6.721363000	4.474520000
6	1.007665000	6.892041000	2.777751000
6	0.932845000	5.433995000	0.775354000
6	0.967973000	3.907040000	-1.225202000
16	0.886551000	-2.905756000	1.534369000
6	2.516696000	-1.969343000	-2.823803000
6	4.873315000	-2.698307000	-2.711971000
6	6.435410000	-1.767860000	-1.046111000
6	7.036929000	2.347765000	-2.093675000
1	6.029737000	0.498206000	-2.541838000
1	7.909494000	4.231667000	-1.403978000
1	-1.129767000	7.037459000	5.444797000
6	0.484325000	7.302253000	3.994011000
1	1.943890000	7.328229000	2.395977000
1	1.862413000	5.901875000	0.416201000
6	0.202406000	3.729181000	-2.393513000
6	2.340013000	3.583990000	-1.283962000
8	1.060520000	-2.301440000	2.872688000
8	-0.075544000	-4.044074000	1.429283000
6	3.571645000	-2.761166000	-3.278447000
6	1.153742000	-2.101708000	-3.404402000
6	5.909941000	-3.582376000	-3.136738000
6	7.413947000	-2.656183000	-1.468377000
1	6.636705000	-1.077379000	-0.215129000
1	7.623177000	2.326822000	-3.024692000
1	1.000123000	8.070901000	4.588823000
6	0.780468000	3.249651000	-3.575572000
1	-0.863231000	3.986083000	-2.386929000
6	2.913633000	3.110318000	-2.468231000
1	2.959234000	3.671262000	-0.378966000
1	3.398450000	-3.469539000	-4.101521000
6	0.716079000	-3.352531000	-3.902330000
6	0.258456000	-1.013923000	-3.531240000
1	5.688382000	-4.303194000	-3.938463000
6	7.157625000	-3.557731000	-2.535478000
1	8.392453000	-2.668724000	-0.965590000
6	2.151664000	2.931648000	-3.644526000
1	0.131774000	3.133983000	-4.454104000
1	3.985406000	2.864822000	-2.467215000
6	-0.538986000	-3.499730000	-4.503936000
1	1.365166000	-4.233837000	-3.788650000

6	-1.001561000	-1.173555000	-4.122181000
1	0.538148000	-0.018635000	-3.166262000
1	7.942596000	-4.255563000	-2.862664000
6	2.828859000	2.425089000	-4.929379000
6	-1.436929000	-2.413413000	-4.628234000
1	-0.829447000	-4.495578000	-4.873691000
1	-1.658494000	-0.299025000	-4.173370000
6	-3.430129000	3.754761000	-2.981093000
6	3.963192000	3.402258000	-5.315307000
6	1.839811000	2.326405000	-6.103472000
6	3.425862000	1.022749000	-4.680505000
6	-2.817021000	-2.600664000	-5.283230000
6	-4.312268000	4.604385000	-2.292252000
6	-3.259552000	2.434371000	-2.547762000
1	4.462408000	3.064718000	-6.247403000
1	3.567612000	4.424146000	-5.485401000
1	4.735466000	3.464512000	-4.522645000
1	2.366741000	1.961634000	-7.008206000
1	1.018293000	1.613530000	-5.887225000
1	1.390517000	3.309569000	-6.350311000
1	4.132601000	1.022909000	-3.828740000
1	2.629903000	0.287253000	-4.456746000
1	3.973148000	0.669137000	-5.578946000
6	-2.637644000	-3.241780000	-6.677504000
6	-3.559677000	-1.261654000	-5.445779000
6	-3.687678000	-3.524504000	-4.398943000
6	-5.026975000	4.119683000	-1.186337000
6	-3.956194000	1.936270000	-1.431882000
1	-3.622977000	-3.366066000	-7.172318000
1	-2.003965000	-2.605117000	-7.327456000
1	-2.164643000	-4.242529000	-6.620033000
1	-4.537622000	-1.436819000	-5.938422000
1	-3.753720000	-0.784256000	-4.465443000
1	-2.987735000	-0.553156000	-6.078626000
1	-3.198566000	-4.504061000	-4.219810000
1	-3.898754000	-3.046435000	-3.421640000
1	-4.662171000	-3.723732000	-4.891332000
6	-4.852741000	2.789608000	-0.760360000
6	2.539605000	-4.323880000	-0.198521000
6	2.488985000	-3.602094000	1.009080000
6	3.727269000	-4.913160000	-0.649936000
6	3.676525000	-3.445518000	1.746939000
6	4.899456000	-4.772996000	0.110107000
6	4.880166000	-4.012472000	1.288951000
9	3.729848000	-2.752040000	2.883489000
9	6.008989000	-3.840918000	1.981749000
9	6.033802000	-5.339384000	-0.296691000
9	3.750064000	-5.588664000	-1.803014000
9	1.458537000	-4.442180000	-0.975819000
9	-2.367269000	1.680657000	-3.197133000
9	-5.562911000	2.402257000	0.295071000
9	-5.876883000	4.922314000	-0.543227000
9	-4.474050000	5.863789000	-2.693476000

9	-2.729279000	4.223401000	-4.020259000
6	-2.947196000	-3.539318000	-0.420116000
6	-1.780703000	-3.874076000	-1.280261000
6	-1.437698000	-5.315075000	-1.678192000
6	-1.073023000	-6.203199000	-0.501050000
8	-1.982781000	-6.092426000	0.506272000
1	-1.984493000	-3.289099000	-2.214467000
1	-0.553596000	-5.284121000	-2.337275000
6	-4.305367000	-4.009852000	-0.636210000
6	-5.371907000	-3.093081000	-0.395514000
6	-4.627385000	-5.317270000	-1.096667000
6	-6.694041000	-3.466395000	-0.649473000
1	-5.153874000	-2.058991000	-0.090179000
6	-5.956023000	-5.690007000	-1.307144000
1	-3.839241000	-6.067591000	-1.206455000
6	-6.992841000	-4.763502000	-1.097208000
1	-7.499585000	-2.733618000	-0.494078000
1	-6.188618000	-6.715284000	-1.630738000
1	-8.037899000	-5.058200000	-1.278266000
6	-2.672441000	-2.599813000	0.641811000
1	-1.946194000	-3.163658000	1.296346000
1	-1.993730000	-1.781759000	0.295200000
1	-3.527469000	-2.229086000	1.223699000
1	-1.623024000	-6.595653000	1.268756000
1	-2.243954000	-5.803861000	-2.263127000
1	-0.881009000	-3.397817000	-0.848018000
8	-0.116711000	-6.946122000	-0.450040000

Product-complex for after cyclization

1	-2.217386000	2.925654000	6.593446000
6	-1.542444000	2.725686000	5.735682000
6	-0.231993000	2.072879000	6.227410000
1	-1.351249000	3.688175000	5.224483000
1	-2.081522000	2.068162000	5.026958000
6	0.717821000	1.837788000	5.039694000
6	-0.581309000	0.767372000	6.963020000
6	0.465221000	3.027896000	7.225060000
6	-2.545813000	-0.495950000	3.783154000
6	1.251029000	0.571909000	4.723026000
6	1.124763000	2.924646000	4.232925000
1	0.319779000	0.274272000	7.380330000
1	-1.262793000	0.987143000	7.809432000
1	-1.099251000	0.045292000	6.300812000
1	-0.190625000	3.218007000	8.100075000
1	1.416133000	2.592487000	7.593614000
1	0.698410000	4.005202000	6.757075000
6	-3.754354000	0.045202000	3.288685000
6	-2.484422000	-1.807800000	4.264671000
1	-1.631750000	0.111424000	3.794579000
6	2.162307000	0.394163000	3.672485000
1	0.963303000	-0.312358000	5.307738000

6	2.035925000	2.757451000	3.186480000
1	0.719879000	3.931086000	4.416437000
6	-3.851134000	1.463727000	2.861819000
6	-4.884861000	-0.799176000	3.270768000
6	-3.615322000	-2.650126000	4.271841000
1	-1.513294000	-2.177887000	4.621665000
6	2.584311000	1.489254000	2.890104000
1	2.549179000	-0.612327000	3.471161000
1	2.303222000	3.623907000	2.564059000
6	-2.778863000	2.104281000	2.161770000
6	-4.948308000	2.255715000	3.191195000
6	-4.814269000	-2.112229000	3.754017000
1	-5.824103000	-0.429296000	2.833548000
6	-3.554574000	-4.073198000	4.852922000
6	3.568178000	1.354923000	1.786830000
8	-1.696051000	1.317523000	1.819254000
6	-2.735736000	3.476638000	1.889170000
1	-5.778951000	1.811163000	3.759577000
6	-5.028872000	3.628411000	2.826895000
1	-5.717930000	-2.736150000	3.704216000
6	-4.465785000	-5.038173000	4.068337000
6	-4.035407000	-4.001730000	6.322078000
6	-2.118525000	-4.636384000	4.825800000
6	3.499384000	0.284489000	0.841963000
6	4.572085000	2.298684000	1.582607000
15	-1.311384000	1.002373000	0.242878000
6	-3.918358000	4.253996000	2.150183000
6	-1.463815000	4.094499000	1.416278000
6	-6.181884000	4.411774000	3.131085000
1	-4.194018000	-5.075119000	2.995934000
1	-4.373639000	-6.063899000	4.479708000
1	-5.534956000	-4.757472000	4.134359000
1	-4.008187000	-5.007291000	6.792092000
1	-3.392689000	-3.322609000	6.918162000
1	-5.075166000	-3.621248000	6.381015000
1	-2.122026000	-5.688529000	5.175169000
1	-1.682031000	-4.618695000	3.806035000
1	-1.434836000	-4.074544000	5.492391000
8	2.510157000	-0.673072000	1.032287000
6	4.341895000	0.143624000	-0.263802000
1	4.676978000	3.129954000	2.296210000
6	5.461551000	2.233531000	0.474235000
8	-1.373999000	2.527333000	-0.394198000
7	0.189905000	0.504456000	0.420490000
7	-2.241611000	0.094276000	-0.663378000
6	-4.050114000	5.612808000	1.737332000
6	-0.820660000	5.147491000	2.167574000
6	-0.802486000	3.588076000	0.295035000
1	-7.016415000	3.929820000	3.663363000
6	-6.265294000	5.740010000	2.745941000
15	1.164201000	-0.664099000	0.085653000
6	4.111354000	-0.953408000	-1.252471000
6	5.349257000	1.154585000	-0.479322000

6	6.441408000	3.249702000	0.263841000
16	-3.806828000	0.079424000	-1.097273000
6	-5.199021000	6.336474000	2.022332000
1	-3.228130000	6.080229000	1.176765000
6	-1.344936000	5.666071000	3.389027000
6	0.445040000	5.661613000	1.701519000
6	0.440122000	4.096689000	-0.198652000
1	-7.166760000	6.327079000	2.976469000
8	1.804531000	-0.319487000	-1.379227000
7	0.675116000	-2.233723000	0.156762000
6	2.853363000	-1.097867000	-1.848674000
6	5.152590000	-1.858632000	-1.661345000
6	6.201578000	1.178338000	-1.623690000
1	6.519733000	4.060491000	1.004646000
6	7.263632000	3.232922000	-0.851261000
8	-4.643769000	-0.563470000	-0.056732000
8	-3.899264000	-0.437069000	-2.480861000
1	-5.285596000	7.378244000	1.679299000
1	-2.294124000	5.273384000	3.773732000
6	-0.668685000	6.644279000	4.105506000
6	1.106660000	6.677708000	2.453498000
6	1.030745000	5.131720000	0.518846000
6	1.078264000	3.552141000	-1.422988000
16	1.184780000	-3.180140000	1.315550000
6	2.568208000	-1.973078000	-2.945267000
6	4.890457000	-2.777480000	-2.743496000
6	6.423188000	-1.908545000	-1.014795000
6	7.132620000	2.191530000	-1.806646000
1	6.110045000	0.386558000	-2.377881000
1	8.008178000	4.028545000	-1.003433000
1	-1.093591000	7.015419000	5.050287000
6	0.565809000	7.161916000	3.634163000
1	2.068503000	7.061758000	2.079362000
1	1.981136000	5.555861000	0.160162000
6	0.354389000	3.372036000	-2.619134000
6	2.457905000	3.255039000	-1.438010000
8	1.172481000	-2.711919000	2.706159000
8	0.378146000	-4.530106000	1.186523000
6	3.612406000	-2.793731000	-3.369303000
6	1.254423000	-1.930245000	-3.638466000
6	5.932752000	-3.655415000	-3.168996000
6	7.408483000	-2.785218000	-1.445198000
1	6.609790000	-1.247056000	-0.157222000
1	7.769486000	2.187428000	-2.703966000
1	1.091972000	7.939339000	4.208121000
6	0.992647000	2.934124000	-3.787802000
1	-0.718366000	3.598781000	-2.649652000
6	3.086727000	2.818618000	-2.608426000
1	3.043029000	3.348416000	-0.511012000
1	3.456853000	-3.462389000	-4.229041000
6	0.690563000	-3.082611000	-4.232642000
6	0.553330000	-0.712366000	-3.797206000
1	5.727456000	-4.347596000	-3.999590000

6	7.168153000	-3.655175000	-2.542044000
1	8.376630000	-2.816659000	-0.923869000
6	2.374156000	2.653384000	-3.816426000
1	0.386550000	2.822089000	-4.697130000
1	4.163981000	2.601843000	-2.571445000
6	-0.494252000	-3.000807000	-4.977572000
1	1.172147000	-4.062239000	-4.091286000
6	-0.644979000	-0.648222000	-4.514569000
1	0.945380000	0.216303000	-3.367069000
1	7.956943000	-4.345319000	-2.875972000
6	3.115524000	2.225347000	-5.095234000
6	-1.196356000	-1.782788000	-5.139245000
1	-0.881581000	-3.921135000	-5.442520000
1	-1.153026000	0.322411000	-4.576765000
6	-3.922922000	4.021855000	-2.202260000
6	4.145427000	3.323393000	-5.450185000
6	2.158422000	2.038437000	-6.285355000
6	3.856944000	0.891138000	-4.858248000
6	-2.483574000	-1.709827000	-5.978622000
6	-4.970620000	4.558909000	-1.435396000
6	-3.636410000	2.653641000	-2.116206000
1	4.695260000	3.053859000	-6.375966000
1	3.644995000	4.298831000	-5.616309000
1	4.889562000	3.458041000	-4.639605000
1	2.730776000	1.718620000	-7.179385000
1	1.397165000	1.258803000	-6.079106000
1	1.632988000	2.979183000	-6.546341000
1	4.558614000	0.954619000	-4.004293000
1	3.145088000	0.069977000	-4.649912000
1	4.443221000	0.612750000	-5.758246000
6	-2.153476000	-2.136734000	-7.426884000
6	-3.071870000	-0.286945000	-5.998672000
6	-3.554816000	-2.654373000	-5.389692000
6	-5.719563000	3.723044000	-0.594214000
6	-4.319828000	1.822442000	-1.210215000
1	-3.064259000	-2.091537000	-8.059340000
1	-1.390004000	-1.467705000	-7.873412000
1	-1.762537000	-3.173143000	-7.472056000
1	-3.996690000	-0.277210000	-6.610141000
1	-3.335803000	0.055052000	-4.978289000
1	-2.369023000	0.443421000	-6.448543000
1	-3.218155000	-3.710481000	-5.381347000
1	-3.813162000	-2.351049000	-4.356087000
1	-4.478782000	-2.608328000	-6.002254000
6	-5.387468000	2.361980000	-0.469583000
6	2.984218000	-4.550158000	-0.277956000
6	2.831831000	-3.772860000	0.889473000
6	4.224937000	-5.113995000	-0.606000000
6	3.960903000	-3.494428000	1.684433000
6	5.336622000	-4.866176000	0.215749000
6	5.212790000	-4.030757000	1.337280000
9	3.900290000	-2.725130000	2.767836000
9	6.287895000	-3.752757000	2.075393000

9	6.516147000	-5.404556000	-0.077452000
9	4.350694000	-5.872816000	-1.694020000
9	1.956445000	-4.774588000	-1.091343000
9	-2.660538000	2.168944000	-2.890030000
9	-6.119531000	1.635808000	0.373407000
9	-6.747577000	4.226270000	0.090765000
9	-5.253729000	5.859009000	-1.506126000
9	-3.207809000	4.813274000	-3.009980000
6	-2.817937000	-3.861638000	-0.137713000
6	-2.958451000	-3.260892000	-1.549397000
6	-2.527296000	-4.411056000	-2.452978000
6	-1.442604000	-5.092421000	-1.659185000
8	-1.619899000	-4.733636000	-0.312984000
1	-3.975708000	-2.883706000	-1.743639000
1	-2.127862000	-4.104867000	-3.433918000
6	-4.012956000	-4.736404000	0.228461000
6	-5.270890000	-4.112878000	0.376211000
6	-3.903201000	-6.120749000	0.449282000
6	-6.394768000	-4.867904000	0.736350000
1	-5.364535000	-3.026453000	0.216511000
6	-5.034007000	-6.874670000	0.807109000
1	-2.923880000	-6.611033000	0.354739000
6	-6.281395000	-6.251928000	0.952738000
1	-7.369334000	-4.369126000	0.850845000
1	-4.932917000	-7.957131000	0.978995000
1	-7.165612000	-6.842541000	1.236513000
6	-2.507620000	-2.868601000	0.967273000
1	-2.322983000	-3.391775000	1.923079000
1	-1.636853000	-2.247141000	0.699512000
1	-3.369392000	-2.192070000	1.109114000
1	-0.431773000	-4.542069000	0.517916000
1	-3.343145000	-5.146867000	-2.622636000
1	-2.261247000	-2.407776000	-1.657204000
8	-0.537839000	-5.812197000	-1.990274000
